# Analysis of metabolic dynamics during drought stress in Arabidopsis plants

**DOI:** 10.1038/s41597-022-01161-4

**Published:** 2022-03-21

**Authors:** Fidel Lozano-Elena, Norma Fàbregas, Veredas Coleto-Alcudia, Ana I. Caño-Delgado

**Affiliations:** 1 Department of Molecular Genetics, Centre for Research in Agricultural Genomics (CRAG) CSIC-IRTA-UAB-UB, Campus UAB (Cerdanyola del Vallès), 08193 Barcelona, Spain; 2 Present Address: Vetgenomics, Campus UAB - Cerdanyola del Vallès, Barcelona, 08193 Spain

**Keywords:** Transcriptomics, Molecular engineering in plants, Metabolomics

## Abstract

Drought is a major cause of agricultural losses worldwide. Climate change will intensify drought episodes threatening agricultural sustainability. Gaining insights into drought response mechanisms is vital for crop adaptation to climate emergency. To date, only few studies report comprehensive analyses of plant metabolic adaptation to drought. Here, we present a multifactorial metabolomic study of early-mid drought stages in the model plant *Arabidopsis thaliana*. We sampled root and shoot tissues of plants subjected to water withholding over a six-day time course, including brassinosteroids receptor mutants previously reported to show drought tolerance phenotypes. Furthermore, we sequenced the root transcriptome at basal and after 5 days drought, allowing direct correlation between metabolic and transcriptomic changes and the multi-omics integration. Significant abiotic stress signatures were already activated at basal conditions in a vascular-specific receptor overexpression (*BRL3ox)*. These were also rapidly mobilized under drought, revealing a systemic adaptation strategy driven from inner tissues of the plant. Overall, this dataset provides a significant asset to study drought metabolic adaptation and allows its analysis from multiple perspectives.

## Abstract (summary)

**Table:** 

Measurement(s)	gene expression • metabolite
Technology Type(s)	gene expression detection method • gas chromatography time-of-flight mass spectrometry
Factor Type(s)	time • drought • genotype • plant tissue
Sample Characteristic - Organism	Arabidopsis thaliana
Sample Characteristic - Environment	greenhouse soil

Machine-accessible metadata file describing the reported data: 10.6084/m9.figshare.17705021

## Background & Summary

Drought is a major cause of agricultural yield loss^1^. Furthermore, predictions for the current global warming and climate change scenario forecast an increase in the frequency and acuteness of drought and heat stress waves^2^. In this context, the study of plant adaptation to stress becomes of great importance as first step to eventually modify these mechanisms and develop better adapted crops.

Metabolic adaptation to drought is a well-known phenomenon previously described^3–5^, in which stands out the accumulation of sugars and other metabolites known as osmoprotectants^5–7^. However, many of these metabolomic studies have been focused to well-known osmoprotectants or limited to very specific phases of drought or using dehydration and osmosis rather than withholding water treatments^3,4,8,9^. The engineering of key enzymes involved in the synthesis of drought-regulated metabolites have been proven successfully in producing drought tolerant plants^10–13^. This strategy is an important biotechnological approach. Nevertheless, targeting specific enzymes has a limited impact in the metabolism. In this context, targeting signaling components, such as hormones signaling pathways, seems to be more promising when comes to systemically push plant metabolism towards a resilience status to overcome stress^14–16^.

During the last two decades, field studies have pointed towards the plant hormone Brassinosteroids (BRs) as a biotechnological target enabling better yields and plant adaptation^17,18^. However, the mechanisms lying behind the capacity of BRs have remained controversial. Despite the beneficial effects of exogenous hormone application in plant stress tolerance^17^, the modification of the canonical signaling pathway have unveiled a non-linear picture of the action of BRs: The overexpression of the main receptor Brassinosteroid Insensitive 1 (BRI1) in tomato yielded drought sensitive plants^19^. Similarly, the activation of downstream players in Arabidopsis also yield drought-sensitive plants^20^. Recently, we published that enhanced expression of the non-canonical BR receptor Brassinosteroid Insensitive-Like 3 (BRL3), especially in the vascular tissues of the plant, yielded drought resistant plants without major growth penalization^21^. The reasons for studying BRL3 instead of the canonical BR receptor Brassinosteroid Insensitive 1 (BRI1), were supported by, (*i*) the lack growth arrest phenotypes upon BRL3 mutations (oppositely to BRI1), which point to specialized functions^22^; (*ii*) the presence of stress-responsive proteins complexed together with BRL3 in the plasma membrane^23^; and, (*iii*) the restricted BRL3 native expression pattern to the phloem cells in the vasculature of Arabidopsis plants^22,23^.

**Fig. 1:**
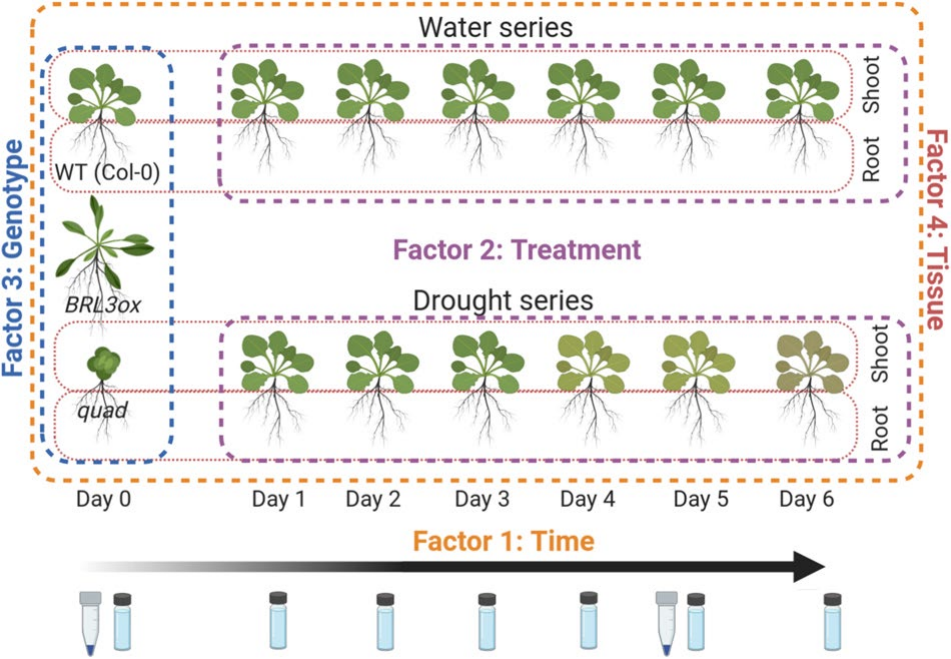
Experimental design and sampling procedure. Scintillation vials represent metabolite sampling points whereas microtubes represent RNA sampling points (Days 1 and 5).

This last point and its potential implications in the systemic distribution of metabolites through the phloem of the plant, prompted us to investigate the metabolic adaptation to drought of BRL3 overexpressing plants (*BRL3ox*). Here, we report a time-course metabolomic study along the first six days of a controlled withholding water experiment in the model plant *Arabidopsis thaliana*. Aerial tissues (shoots) were separated from underground tissues (roots) and analyzed separately, also providing insights on the shoot-to-root metabolite transport. Furthermore, a third factor was considered. Apart from the wild-type (WT, ecotype Col-0), two mutants, one overexpressing BRL3 receptor (*BRL3ox*) and other one lacking all BR receptors and the canonical coreceptor BAK1 (quadruple mutant, named *quad*), were included in the study. We also sequenced messenger RNA from WT and *BRL3ox* roots at basal conditions and after 5 days of drought^21^. A graphical overview of the experimental design and the sampling is depicted in Fig. 1.

Several studies have previously addressed plant metabolic adaptation to drought in model organisms as Arabidopsis or rice^7^. Many of these present interesting integrative *omics* approaches, however, most of them only involve pairwise comparisons (normally involving a defined drought condition and different genotypes)^24–26^ and only a few approach drought adaptation with a time course design, although not involving other factors^8,27^. Our study proposes a multifactorial design including a temporal dimension (that can be treated as a continuous variable) and several factors such as tissue or genotype with multiple levels (that can be treated as discrete variables or factors). Overall, our dataset provides a significant asset to metabolic analyses of plant adaptation to drought, allows its analysis from multiple perspectives and opens possibilities for future crop improvement through metabolic engineering and biotechnology approaches. The major findings of our study are (i) a remarkable increase in osmoprotectant metabolites in the roots of *BRL3ox* plants already at basal conditions, which followed steep accumulation dynamics towards later stages of drought. (ii) Enrichment of response to stress and water deprivation Gene Ontology (GO) categories among Differentially Expressed Genes of RNAseq, already at basal conditions. (iii) Over-representation of vascular-specific genes among the deregulated genes and (iv) direct transcriptional control of sugar metabolic pathways by BRL3, as suggested by the integration of metabolomics and transcriptomics^21^. The overall plant effects observed with the overexpression of a vascular receptor encourage the study of hormone signaling from a tissue-specific perspective and emerges as a promising approach to boost agriculture adaptation to coming challenges^28^.

## Methods

### Plant growth and sample collection

Arabidopsis seeds of WT (Col-0 ecotype) and mutants, *BRL3ox* and *quad*^21^, were sterilized with 35% bleach and vernalized for two days in dark and at 4 °C. Then seeds were sowed in *in vitro* plates filled with half-strength Murashige and Skoog medium (MS) and grown for 7 days in long-day conditions (16 h light/8 h dark) at 22 °C. Then, 7-day-old seedlings were transferred to pots containing approx. 30 g of universal substrate supplemented with perlite and vermiculite. Plants were let growth in a chamber with long day conditions at 22 °C and a relative humidity of 60% for two weeks before the drought start. Before starting the drought time course, 3-week-old plants were watered until field capacity (maximum water absorption by the soil), and the excess water was retired. Next day was considered as day 1. For a period of 6 days, pools of five plants per genotype and conditions were collected every day. The position of the different genotypes in the trays and position of replicates within the growth chambers were randomly distributed to avoid positional effects or biases. Shoots were directly cut with a razor blade, softly dried with a tissue paper and flash-frozen in liquid nitrogen. Roots were gently washed in water several times in order to clean a detach the soil leftovers, keeping the process under 2 min to minimize the induction of metabolic and transcriptomic changes. Then, roots were gently dried and flash-frozen in liquid nitrogen. Samples were kept at −80 °C until metabolite or RNA extraction. Four independent plants were bulked in each biological replicate. A total of 5 independent biological replicates were collected.

### Metabolomic data

#### Metabolite extraction

Four entire shoots were grinded using the Frosty Cryogenic grinder system (Labman). Four entire root samples were grinded in the Tissue Lyser Mixer-Mill (Qiagen). Roots were aliquoted into 30 mg (+/− 5) samples and shoots into 55 mg (+/− 5) samples. Before starting the extraction, the exact weight for each sample was wrote down. Grinding and aliquoting processes must be carried in liquid nitrogen avoiding defrosting of samples. A sample list file with sample name and exact weight was prepared. This is essential for data normalization. The Ribitol stock (0.2 mg/ml in water) was prepared. The extraction buffer (20 ml of 100% Methanol pre-mixed with 1 ml of Ribitol stock) was prepared. One zirconia bead and 500 μl of 100% methanol premixed with ribitol (20:1) were carefully added to the Eppendorf containing the frozen aliquoted samples and vortexed for 15 sec. Samples were then homogenized in the Tissue Lyser (Qiagen) 3 min at 25 Hz. Samples were centrifuged 10 min at 14,000 rpm (10 °C) and resulting supernatant was transferred into fresh Eppendorf tubes (1.5 ml). Next, 200 μl of CHCl_3_ were added to the samples and vortexed 30 sec. Note that this is a critical step: make sure sample is well mixed in one single phase. Then, 600 μl of H_2_O were added to the samples and carefully vortexed 15 sec. Samples were centrifuged 10 min at 14,000 rpm (10 °C). Note that this is a critical step: samples will be distributed in two phases, do not disrupt them. 100 μl from the upper phase (polar phase) were transferred into fresh Eppendorf tubes (1.5 ml) and dried in the speed vacuum for at least 3 h without heating. Dry aliquots were kept at −80 °C until the following day. Next day, 100 μl aliquot samples were taken out of −80 °C and dried again in the speed vacuum for 30 min. This is a critical step: extracts should be completely dried without any water drops inside of the tubes. Contamination of water will disturb derivatization and interfere with the analysis. Make sure there are no water drops in your sample before proceeding to derivatization. Next, 40 μl of derivatization reagent Methoxyaminhydrochlorid (20 mg/ml in Pyridin) were added to each sample. One extra sample vial (blank) with only Methoxyaminhydrochlorid (20 mg/ml in Pyridin) was prepared. Samples were shaken for 2 h at 900 rpm at 37 °C. Drops on the cover were shortly spun down. One sample vial with 1 ml MSTFA mix [*N*-Methyl-*N*-(trimethylsilyl) trifluoroacetamide) +20 μl FAME mix (fatty acid methyl ester)] was prepared. 70 μl MSTFA + FAMEs were added to each sample followed by shaking 30 min at 900 rpm at 37 °C. Drops on the cover were shortly spun down.

#### Metabolite chromatography and detection

Samples were transferred into glass vials specific for injection in a gas chromatography (GC) time-of-flight (TOF) mass spectrometry (MS) system. Samples were injected in the chromatography in four separate batches (See supplementary Table 1). Root samples were divided in two batches (set 1 and set 2). Set 1 contained replicates 1 and 2 for each genotype and condition in roots (n = 74 samples). Set 2 contained replicates 3, 4 and 5 of each genotype and condition in roots (n = 117 samples). Shoot samples were divided in two batches (set 3 and set 4). Set 3 contained replicates 1 and 2 for each genotype and condition in shoots (n = 78 samples). Set 4 contained replicates 3, 4 and 5 for each genotype and condition in shoots (n = 117 samples). This experimental design allowed that each sample set included representative replicates of each genotype and condition and ran in the GC/MS/MS machines for approximately 24 hours. The GC–TOF–MS system comprised of a CTC CombiPAL autosampler (Agilent), a 6890 N gas chromatograph (Agilent), and a LECO Pegasus III TOF–MS (LECO Inc.) running in electron impact ionization (EI^+^) mode. Chromatograms were evaluated and converted to CDF formatted file using Chroma TOF 1.0 (Leco) Pegasus software.

#### Metabolite identification and annotation

GC-MS-based metabolite profiling derived chromatogram files contain the mass spectral tags (MSTs), which are the characteristic patterns of fragment ions generated by electron impact ionization (EI) of the separated molecules. These fragment ions are subsequently detected by TOF-MS. MSTs are reported as a list of ions, which are characterized by mass of fragment peaks, chromatographic retention time index (RI) determined by RI of FAMES detected in the same analytical batch, and arbitrary abundance. Fragment masses and their RI allow the peak identification, while the abundance allows the quantification of the metabolic compounds. Mass spectral tags of identified peaks were evaluated with TagFinder 4.0^29^ and Xcalibur (ThermoFisher) softwares. Xcalibur was used to pick the metabolite peak area. TagFinder was used for peak annotation and quantification of metabolic data. TagFinder is a Java based program which supports both non-targeted and targeted metabolite profiling analyses. TagFinder is freely available for academic use in the following link: https://www.mpimp-golm.mpg.de/10871/Supplementary_Materials. For a detailed description of TagFinder please see^29^. Xcalibur was used to pick the metabolite peak area.

Below we describe a detailed step-by-step of the TagFinder workflow for our data analysis:

1. Import of fragment ion data, namely mass, time and arbitrary abundance (intensity), from a chromatography file (.cdf). Within the peak finder settings, samples with lower intensity than 150 were removed. The.cdf raw files for all samples analyzed within this study are available at MetaboLights database with the identifier MTBLS2289^30^.
2. Retention index (RI) calculation (retention time calibration):
  2.1. Load time standard list: Within the time standard Finder Panel select open file and load the time standard list. Metabolites were identified by comparing to this database of authentic standards^31^.
  2.2. Find the FAMEs peaks: Adjust search parameters, run the time standard finder, check the results. Search parameters were adjusted in order to detect all FAMES within all samples.
  2.3. Fill the Retention Index (RI) list: Import the retention time information results for each FAME and each sample. Save the list as a.txt file.
  2.4. Time Index calculation: run time index calculation using the RI list (.txt file) generated in the previous step.
3. Create a sample annotation file containing raw name, sample name, condition and fresh weight for each sample. Save it as Sample Annotation.txt file
4. Annotating sample groups:
  4.1. Open Sample Annotator panel.
  4.2. Assign groups from the Sample Annotation.txt file: sample name is assigned to raw name column and group is assigned to condition. Save file and refresh.
5. Set up TagFinder parameters. Within TagFinder settings, click edit settings and indicate the number of replicates of your experiment.
6. Run TagFinder to compare peaks among all samples. A tags.tab file will be generated. This file includes information of all detected mass fragments:
  6.1. **Tag_Time_Index**: RI in which the mass fragment was detected.
  6.2. **Tag_Mass**: molecular mass of the fragment.
  6.3. **Time_Group**: Time group (peaks) in which the mass fragment was involved and the intensities in each sample.
7. Peak annotation using TargetFinderPanel. Open External Tools within the tools tab. Select Jar File and select tagtools.jar, pbuilder.TargetFinderPanel and click Run.
8. Load FAMEs libraries including information about standard compounds. Updated libraries can be downloaded from GMD: http://gmd.mpimp-golm.mpg.de/download/GMD_20111121_MDN35_FAME_TFLIB.txt
  8.Load tags.tab excel file and set up target finder with the following parameters:
  8.1. Select Group by TIME_GROUP
  8.2. Set Min matching probability to 0
  8.3. Set Max fragment ratio deviation to 10
9. Select all **metabolites**, click find targes and go to results tab.
10. Within results panel, candidate metabolites appear in the left side, mass spectrum in the upper right side and the detail of mass spectrum in the down right side.
11. **Annotate the peaks** following these basic criteria:
  11.1. Time deviation should be small (less than 0.1)
  11.2. Mass spectrum is similar to standard compound
  11.3. At least 3 major mass fragments should be detected in sample mass spectrum
  11.4. Intensities of most masses should be linearly correlated
  11.5. Take one metabolite from one-time group
12. Export target match results into target_results.txt file containing the data matrix.
13. Extraction of selective/representative mass tags:
  13.1. Representing the behavior of all other masses
  13.2. Stably detected
  13.3. Low background
  13.4. Most prominent

#### Metabolomic data analysis

The resulting matrix was normalized against the internal standard, Ribitol, to obtain the abundance of each metabolite per sample. Then, this matrix was normalized again with the fresh weight of each sample to obtain the abundance per sample weight. The matrix was log-transformed and the distribution per sample plotted as boxplots in order to scan for outliers, that is, samples showing unusual distribution (shifted one or more order of magnitude respect the overall sample median), which are likely artifacts. These samples were deleted. No further statistical normalization was applied to resulting data set.

For statistical inference of the metabolomic dataset, we used pairwise comparisons between genotypes at a given time point using a *Student’s t-test*. For shoot-to-root partition, we generated a new variable (ratio shoot-root) based on paired samples. Pairwise comparisons between ratios were also with *Student’s t-test*. For the analysis of differential dynamics along the time course we used the maSigPro^32^ package in R, which is based on fitting the data to polynomials curves and compare their coefficients (See Supplemental Material). For the identification of Dynamical Network Biomarkers (DNBs), we followed the method proposed by Chen *et al*.^33^: In order to detect early-warning signals of drought stress, marked and nonlinear transitions known as “critical points” are sought. These are characterized by a dominant group of molecules that, once clustered according their profiles: (i) drastically increase their average Standard Deviation (SDin); (ii) Drastically increase their intra-correlation (measured by absolute Pearson correlation coefficient, PCCin) and (iii) Drastically decrease their correlation with other molecules (PCCout). For the clustering of metabolites, we used hierarchical clustering (complete-linkage). These three signals were then merged in a per cluster Composite Index (CI), according the formula: $CI=\frac{SDin\times PCCin}{PCCout}$. The CI maxima along the time-course provided a significant early-warning of the process under study, in our case the progression of the drought stress in Arabidopsis.

### RNAseq data

#### RNA extraction, library preparation and sequencing

RNA was extracted with Plant Easy Mini Kit (Qiagen) according to manufacturer instructions. The quality of the RNA was checked with Bioanalyzer (Agilent). Stranded cDNA libraries were prepared with TruSeq Stranded mRNA kit (Illumina) according to manufacturer instructions. Single-end sequencing, with 50-bp reads, was performed in an Illumina HiSeq2000 sequencer, at a minimum sequencing depth of 21 M. One sample of *BRL3ox* at drought conditions was removed due to bad RNA quality.

#### RNAseq analysis and functional annotation

Quality of raw reads (fastq files) were assessed using FastQC v0.11.5. Reads were trimmed 5 bp at their 3′ end and quality filtered, keeping only reads with a minimum quality of 28 (Phred) in 80% of the bases. Reads were mapped against TAIR10 genome using HISAT2 v2.1.0^34^. Mapped reads were quantified using only gene features with HtSeq v0.9.1 based on Araport11 genome annotation (retrieved from Phytozome). Diagnosis plots, including PCA, saturation and sensitivity were generated with NOISeq package in RStudio. For differential expression analysis, raw counts were normalized by Trimmed-Mean of M values (TMM) method using edgeR v3.14.0^35^ package in RStudio. Pairwise comparisons and a linear model accounting for both factor interaction (Drought*Genotype) were used to obtain differentially expressed genes.

## Data Records

Metabolomic data is provided in raw peak areas (Supplementary Table 2) and normalized by internal standard and sample fresh weight (Supplementary Table 3). Metabolites having *NA* values in all the samples for a particular tissue were not identified in that tissue. Raw metabolomic data is available at MetaboLight, with accession number MTBLS2289^30^. RNAseq data, in form of raw reads (fastq files) and gene raw counts (once mapped to TAIR10 genome and gene features counted), has been deposited and can be accessed at Gene Expression Omnibus (GEO)^36^ with accession code GSE119382.

## Technical Validation

### Experimental design

Drought is a complex trait, in which many environmental factors have influence^37^. To ensure the quality of the samples and the isolation of drought as the only source of environmental variation, we grew the plants in controlled conditions growth chamber, where light, temperature and humidity were monitored. We designed a multifactorial experiment to investigate metabolite dynamics over early stages of drought and to explore differences in genotypes and source/sink tissues metabolite transport. In order to deal with the typical variability associated to metabolomic experiments^38^, we repeated the drought time course and sampled five bulked plants five times, constituting five biological replicates to be analyzed.

Four factors were involved in the generation of the data set. The first factor was the time, for which we collected samples every day for a time period of six days (0, 1, 2, 3, 4, 5, 6), both in a well-watered conditions regime and after withholding water regime started (we also collected sample at time 0, that is basal conditions). The second factor was the treatment (water/drought), as we kept a watered control along the entire time course to control for any changes due to developmental plant growth. The third factor was the genotype, as we collected samples from WT (Col-0), *BRL3ox* and *quad* mutants. The fourth factor was the tissue, as we collected the aerial parts (shoots) and underground parts (roots) of the plant separately. In total, we collected 390 samples. A summary of the experimental design and the sampling process is depicted in Fig. 1.

### Metabolomic samples distribution

**Fig. 2:**
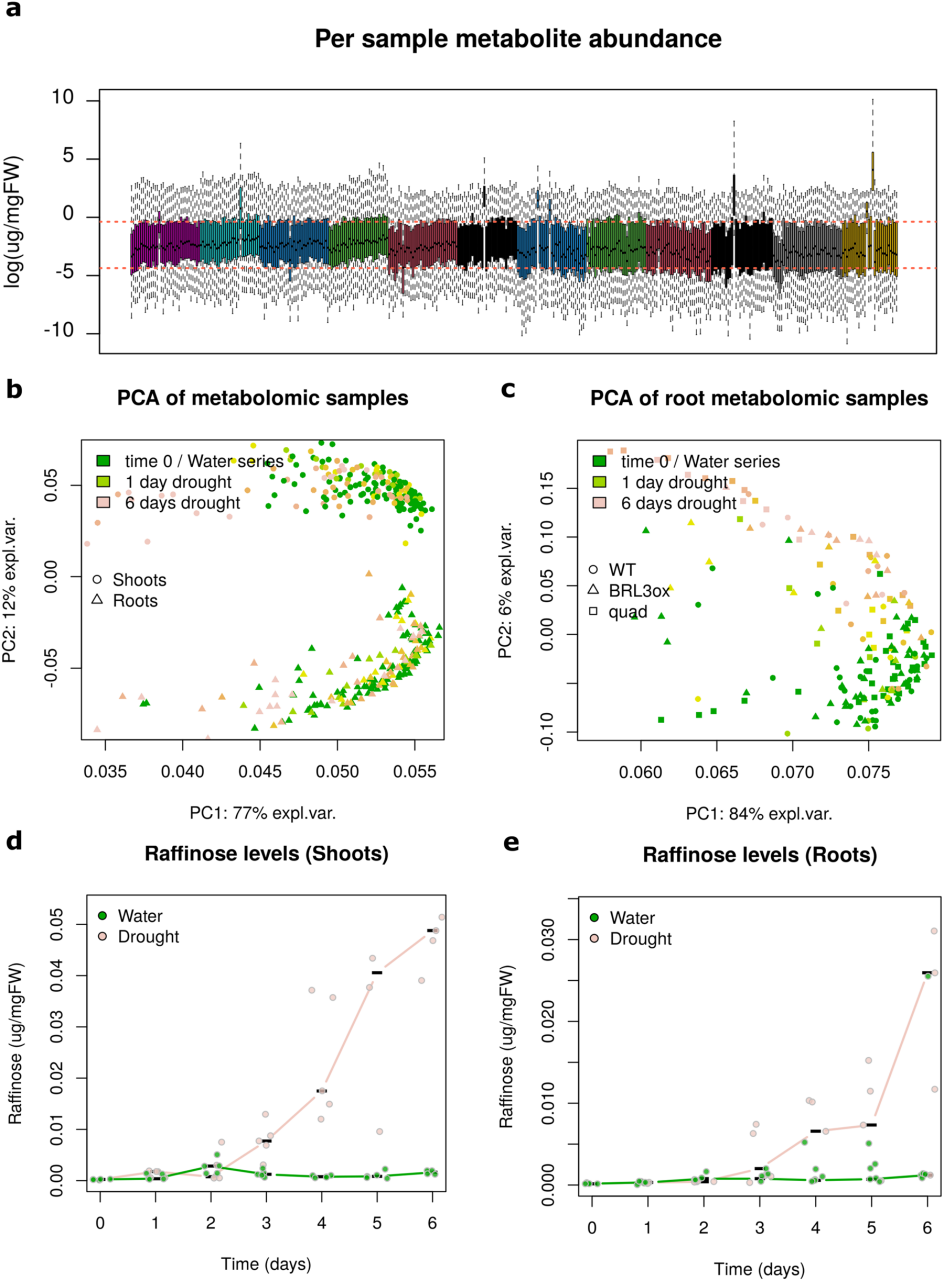
Technical validation. (**a**) Boxplot with the distribution of metabolomic measures (log-scale) per sample. Note the few samples showing an evident outlier distribution. These are likely artifacts and were deleted for subsequent analyses. Red dashed lines denote the Q1 and Q3 of the whole dataset. (**b**) Graphical representation of Principal Component Analysis (PCA). Axis represent the two first components explaining most of the dataset variability. Data matrix was centered and scaled to unit variance with R *prcomp* function. (**c**) Same than in (b) but limited to root samples only. (**d**) Profile of the osmoprotectant sugar raffinose along the drought time course in WT shoots. Points denote individual measurements and lines denote the medians. Note the large accumulation in later stages of drought, whereas the watered control remains unchanged. (**e**) Same than in (d) but in WT roots.

Upon metabolite extraction and analysis, peak areas (chromatograms) corresponding to identified metabolites were quantified (Supplementary Table 2). The resulting data matrix was subjected to two normalization steps. First, normalized by the peak area of an internal standard (Ribitol) and second, by the fresh weight of each sample. The resulting data matrix with metabolite abundance per sample fresh weight (µg/mgFW) was the source for further analyses (Supplementary Table 3). Overall metabolite abundances ranged several orders of magnitude within samples but they showed a consistent homogeneous distribution across samples (Fig. 2a), with the exception of few samples clearly identified as artifacts, given that their overall metabolite distributions were shifted several orders of magnitude respects most of the samples. Although these samples are easily spotted visually (Fig. 2a), as an arbitrary criterion, we removed samples which had a (log-transformed) median over or below the percentiles 0.75 and 0.25 of all (log-transformed) measurements. Removed samples are summarized in Table 1. Due to the good distribution of the samples, no further normalization was applied to the data.

**Table 1:** Deleted samples, identified as outliers.

Sample ID (internal)	log(median)	Sample log(Q1)	Sample log(Q3)
16076oA_23	−8.35	−10.55	−6.40
16075oA_75	−3.24	−4.97	−1.45
16076oA_75	−2.00	−3.10	−1.34
15316oA_27	−2.13	−2.77	−1.30
15316oA_44	−2.93	−3.93	−2.03
15316oA_49	−1.96	−3.40	0.07
15319oA_30	−2.98	−3.53	−2.20
15319oA_52	0.57	−1.20	2.08

The overall median for all measurements in the data set (log-scale) is −6.30 and first quartile (Q1, percentil 0.25) and the third quartile (Q3, percentil 0.75) are −8.01 and −4.13 respectively.

Principal Component Analysis (PCA) of the whole data clearly separated samples according the tissue of origin (Fig. 2b), which reveals the tissue as a major factor explaining the variability in the data. Indeed, substantial differences in metabolism between shoots (source tissue) and roots (sink tissue) are well known, being this partitioning of great importance for plants^5^. However, the component explaining most of the variability correlated with drought time and/or developmental time rather than the tissue (Fig. 2b,c x-axis). This is more evident if PCA is applied only to one tissue, for example roots (Fig. 2c). No clear separation according genotypes is observed in PCA plots. These results indicate that the metabolic changes that occur between the different genotypes only occur in few key metabolites (which would not influence very much the sample distribution in the PCA plots). In addition, we did not detect in the PCAs (nor with other methods) any bias associated with replicates nor with the date-time of chromatography analysis, therefore batch effects were not detected in our dataset. Overall, PCA support the coherence and good quality of the data.

### Sugars accumulation in later drought stages

The accumulation of soluble sugars in plants under drought stress is well described^3–5,7^. In order to confirm that plants were actually perceiving the drought and triggering stress responses, we checked several metabolites as benchmark. Such is the case of the accumulation of raffinose, a sugar known to act as osmoprotectant, being synthesized as defense mechanisms and that can provide plant with drought tolerance^39^. Raffinose clearly accumulated in WT samples at later stages of drought, while remained unchanged in the watered series, both in shoots and roots (Fig. 2d,e). The accumulation profile of raffinose and other metabolites, as sucrose or proline, confirmed that samples actually displayed drought-stress features, biologically validating the experimental design^21^.

### Transcriptomic fingerprints

**Fig. 3:**
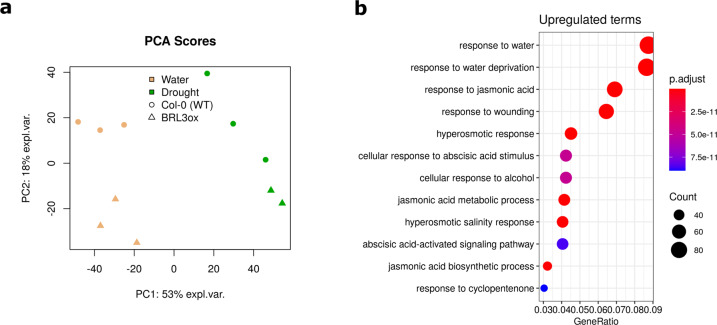
Validation of RNAseq. (**a**) PCA plot of samples based on gene counts (NOISeq R package^42^). PC1 roughly coincide with drought, which clearly separates samples. PC2 roughly corresponds with the genotype, which also separates well the samples. Data matrix was centered and scaled to unit-variance. (**b**) GO enrichment analysis of differentially upregulated genes on the pairwise comparison WT drought vs. WT control. The great enrichment values obtained for response to water (GO:0009415), response to water deprivation (GO:0009414) and other stress-related categories validate the effect of drought on transcriptomics, thus supporting the quality of the dataset. The p.adjust parameter is the FDR-adjusted p-value of the enrichment test. Count is the number of deregulated genes annotated in a particular GO category and GeneRatio is the Count number divided by number of deregulated genes that are not annotated in such category. GO enrichment analysis performed with ClusterProfiler package, based on *org.At.tair.db* annotation.

To support the metabolomic data with gene expression changes, RNA from samples of WT and *BRL3ox* roots at extreme time points of the drought time course (time 0- and 5-days drought) was extracted and sequenced. In this tissue and genotype, we originally found the most relevant differences in osmoprotectant accumulation^21^. Quality control graphs of RNA samples before sequencing (Bioanalyzer) and quality control plots of raw reads from RNAseq are provided in Supplementary Data 1 and 2. These confirmed the good quality of the sequencing. Upon mapping against TAIR10 genome and gene features quantification (Araport11 annotation), PCA plots showed a coherent distribution of the samples (Fig. 3b). The first component corresponded with drought treatment and the second component with genotype (Fig. 3b). Further analyses based on Differentially Expressed Genes (DEG) revealed that a proportion of DEG (~10%) were annotated in “response to water deprivation” and/or other drought-related GO categories^21^. These transcriptomic hallmarks of stress were especially visible in the pairwise comparison of drought-stressed roots versus control conditions. These results further validated, from a biological perspective, the quality of the samples.

## Usage Notes

With the following examples, we aim to illustrate how this data set, with such multifactorial design, can be exploited through different approaches.

### Comparison between genotypes at a given time point

**Fig. 4:**
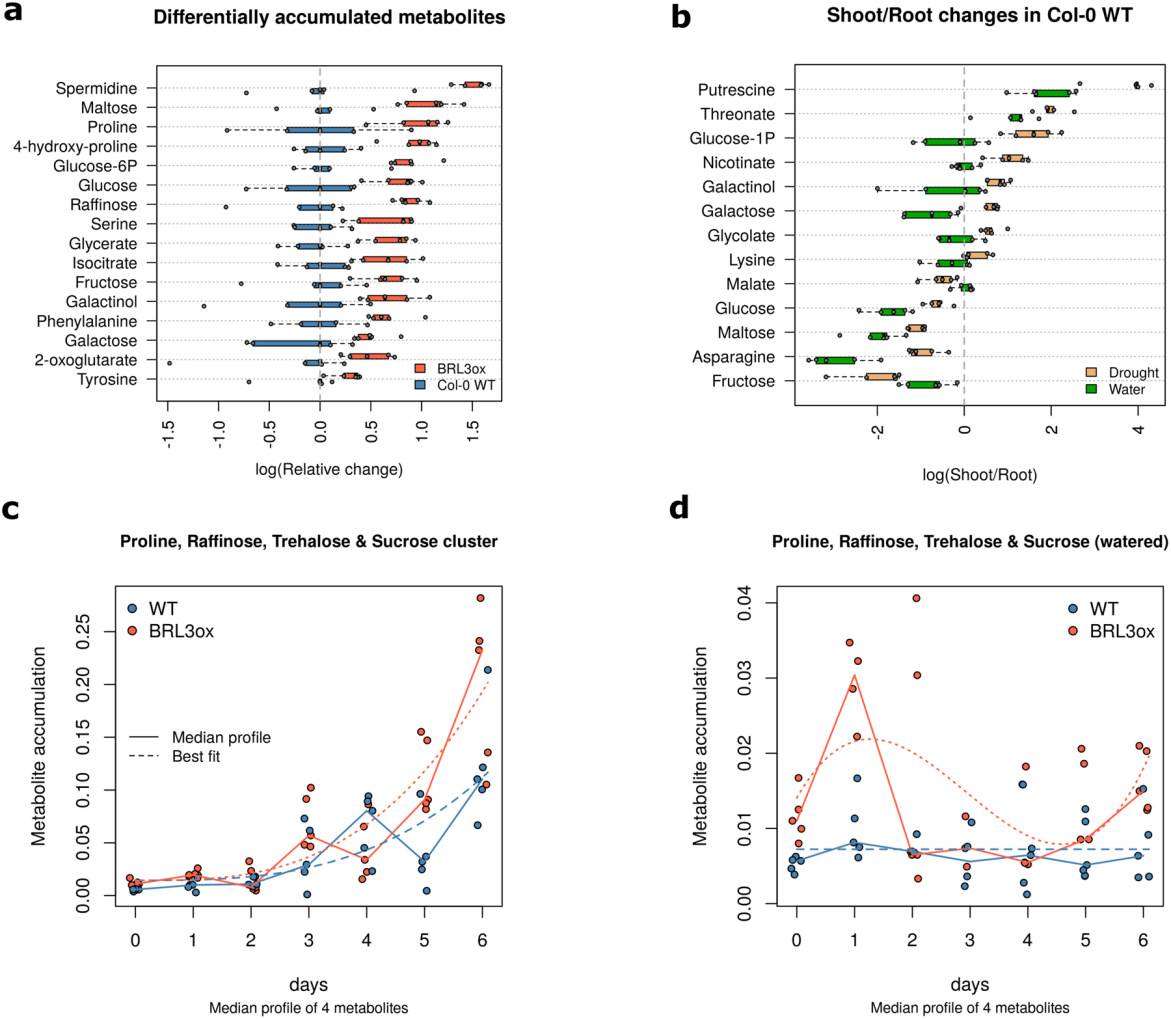
Usage examples: (**a**) Time 0 comparison between WT and *BRL3ox*. Boxplots represents the (log) fold-change relative to the WT median. Points are the particular relativized values of each replicate. Important osmoprotectant metabolites appeared accumulated already in basal conditions in *BRL3ox* roots, supporting the priming hypothesis of these plants. (**b**) Metabolites whose ratio shoot/root is significantly altered after six days of drought in WT plants. Boxplot represent the (log) ratio between shoot and roots. Points represent the particular values of each replicate. (**c**) Median profile of a cluster of metabolites that follow differential dynamics between *BRL3ox* and WT along drought. Note how both genotypes exponentially accumulate these osmoprotectant metabolites along drought, however in *BRL3ox* this accumulation is way steeper. (**d**) Median profiles of the same metabolites than in (c) but in the watered series. Any metabolite was identified as significantly affected by time in the watered series. Note the y-axis scale, despite the apparent fluctuations in *BRL3ox*, these changes are very small compared to drought.

A straight-forward approach, still very informative, is a pairwise comparison between levels of a single factor (e.g. treatment vs. control). For example, comparison at basal conditions (time 0) between *BRL3ox* and WT reveals that *BRL3ox* plants accumulate some metabolites (*t-test*, p-value < 0.025). Interestingly, among these we found well known osmoprotectants, such as proline, raffinose or galactinol (Fig. 4a). This accumulation resulted in better prepared plants to front drought, phenomena referred as priming, which was in accordance with the drought tolerance phenotypes found in *BRL3ox* plants^21^.

### Shoot-to-root metabolite mobilization upon drought

A distinct approach involves the integration of the levels of a factor in a new parameter. This new parameter may offer more relevant information while discard one factor in further comparisons. In our case, calculation of the ratio shoot-to-root would reflect the accumulation balance of a particular metabolite and suppress the *tissue* factor in further analysis. Comparing these ratios allow the investigation on how other factors (i.e. genotype or drought) can affect the accumulation and/or transport of metabolites from source to sink tissues. Unfortunately, as this analysis requires paired samples, if a replicate in shoot has been discarded because likely containing outliers (See Table 1 and Fig. 2a), its paired counterpart in root has to be discarded as well and vice versa. Still, any condition resulted in less than four paired samples to calculate shoot/root ratio. This analysis reveals some metabolites that tend to accumulate in shoots after 6 days of drought in WT plants (*t-test*, p-value < 0.025; Fig. 4b). Interestingly, the plant distribution of sugars as galactinol and galactose, together with glucose (phospho- and unphosphorylated) and maltose is affected upon drought (Fig. 4b). Similarly, further comparisons are possible following this approach. For example, shoot/root ratios of WT vs. *BRL3ox* at a given time point.

### Differential metabolite dynamics upon drought

Other approaches might be involved considering the whole temporal dimension. To find out which metabolites are following differential accumulation dynamics along drought, a method that involves the fitting of the metabolite profiles to polynomial curves was applied (Using maSigPro R package^32^). By comparing coefficients of these curves, a set of metabolites with differential dynamics between WT and *BRL3ox* under drought were identified. Further, based on their accumulation profiles, these metabolites can be clustered according to their stereotypical profile. Interestingly, among the metabolites following differential dynamics we found well known osmoprotectants^21^ (Fig. 4c). The same approach was applied to the control (watered) time-series, yielding no significant metabolites (that is, their accumulation does not depend on time). The profiles of the same osmoprotectants but in watered conditions (fitted to their respective curves) are shown in Fig. 4d.

### Identification of Dynamical Network Biomarkers (DNBs) along drought time course

Another approach would make use of the metabolite variances along time series and the correlations between them in order to identify important metabolite clusters involved in drought responses. Such is the case of the Dynamic Network Biomarkers (DNBs)^33^. Compared with traditional biomarkers, DNBs allow to detect critical points by exploiting network information and its dynamics in time-course data. We applied the DNB method (see Supplementary Material) to identify early-warning signals of drought stress, separating the data by organ and genotype. We used samples at time 0 as controls and considered samples in 1–6 drought time points as case samples.

**Fig. 5:**
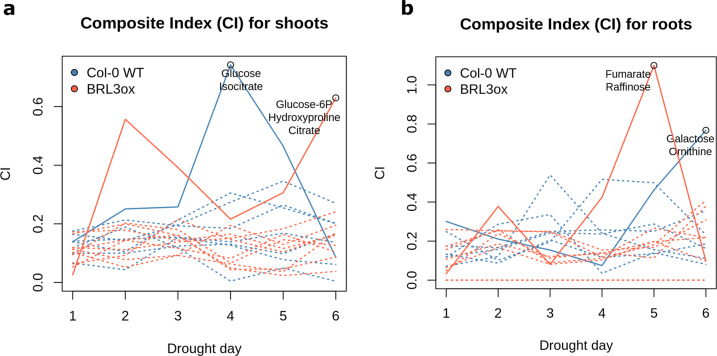
The dynamic evolution of the calculated Composite Index (CI) for each cluster in Col-0 and *BRL3ox* genotypes. Each line represents a metabolite cluster. Dashed lines are CI of clusters that are not yielding DNBs. Solid lines are the cluster in which a DNB is identified at the critical point (encircled). Metabolite names of the DNBs are denoted next to the critical point for each genotype. (**a**) Analysis of CI evolution in shoots. (**b**) Analysis of CI evolution in roots.

The identified DNBs with higher Composite Indexes (Fig. 5, see methods) included osmoprotectant sugars as galactose and raffinose in later drought stages. This finding is consistent with the approaches disclosed above. Additional metabolites such as glucose and hydroxyproline also appeared in DNBs with high CI, pointing at a predominant role of sugar metabolism in the early response to drought stress (Table 2). This inference method, and analogues one, use the global relationships present in the data and have been developed and successfully applied to genomics studies. Although the statistical framework is directly transferable to metabolomic data, major limitations for our dataset were noticed as in somewhat similar approaches when applied to metabolomics^40^. For example, it is extremely dependent on clustering methods and the correlations between metabolites are generally weak, which can potentially lead to CI that do not clearly identify markers and are ineffective providing biological insights. Despite these limitations, we anticipate that the use of this method with large enough data sets (including many more time points and metabolites than the data here presented), can potentially increase the power of the method and unequivocally yield DNBs that are relevant for the biological process under study.

**Table 2:** Identification of DNBs based on metabolites correlation and variance.

Sample	DNB metabolites	Critical point (days)	PCCin	PCCout	SDin	CI
BRL3ox root	Fumarate, Raffinose	5	0.45	0.38	0.93	1.10
*quad* shoot	Urea, Glutamine, Raffinose	3	0.47	0.33	0.58	0.81
Col-0 WT root	Galactose, Ornithine	6	0.31	0.38	0.92	0.76
Col-0 WT shoot	Glucose, Isocitrate	4	0.45	0.64	1.05	0.74
*quad* root	Ornithine, Glutamine	6	0.36	0.37	0.67	0.65
BRL3ox shoot	4-hydroxyproline, Glucose-6-Phosphate, Citrate	6	0.34	0.48	0.89	0.63

### Integration of metabolomics and transcriptomics

Having several *omics* data layers open the possibility of their integration. This can provide a complementary approach for the analysis of the experiment, and eventually, underpinning the results. In our case, we found a prominent osmoprotectant accumulation in drought-stressed roots, especially in the *BRL3ox* line^21^. Accordingly, we sequenced mRNA of these samples and we looked forward combining both *omics*. Metabolomic-transcriptomic integration was based on the principle that changes in metabolites must be driven by changes in the levels and/or the activity of the enzymes involved in their synthesis/transformation/degradation reactions. Using the KEGG pathways atlas, we search for metabolic pathways that had an overrepresentation of DEG and metabolites that were identified to follow differential dynamics upon drought. For that we used PaintOmics web server^41^, but analogous approaches should also yield coherent results. We search for pathways with an overrepresentation of DEG (based on differential drought response between *BRL3ox* and WT) and metabolites identified to follow differential dynamics upon drought (*BRL3ox* vs. WT). The most significant affected pathways by drought stress were the phenylpropanoid biosynthesis, plant hormone signal transduction and in accordance with metabolomic analysis, carbon metabolism: starch and sucrose metabolism and galactose metabolism (Table 3).

**Table 3:** Integration of drought metabolomics and transcriptomics.

Pathway name	Unique genes	Unique metabol.	P-val. Genes	P-val. Met.	combined p-val.
Phenylpropanoid biosynthesis	120	2	6.73e-9	1	1.33e-7
Plan hormone signal transduction	203	1	2.30e-6	1	3.22e-5
Starch and sucrose metabolism	164	10	6.98e-4	1.93e-2	1.64e-4
Galactose metabolism	48	11	4.67e-2	2.27e-3	1.08e-3
Pentose and glucoronate interconversions	58	3	1.13e-3	1	8.85e-3

KEGG pathways affected by the differential drought response between *BRL3ox* and WT roots. Identification of pathways based on the overrepresentation of DEG and differentially accumulated metabolites. Results as obtained from Paintomics3^41^. Unique genes and unique metabolites columns refer to the number of deregulated genes or metabolites mapping in a particular pathway. P-val. Genes/Metabolites columns refer to enrichment values for genes and metabolites independently (Fisher’s exact test). Combined p-val. column is the enrichment value combined for genes and metabolites (Fisher combined probability test).

## Supplementary information

Supplementary Table 1

Supplementary Table 2

Supplementary Table 3

Supplementary Data 1

Supplementary Data 2

## Data Availability

The code used to normalize and analyze transcriptomic and metabolomic data similarly as disclosed in the Usage Notes section have been deposited in Github repository (https://github.com/fle1/Scientific_Data).
