# Supplementary material for: Analysis of metabolic dynamics during drought stress in Arabidopsis plants
Source: Sci Data. 2022 Mar 21;9:90. doi: 10.1038/s41597-022-01161-4 (PMC8938536; doi:10.1038/s41597-022-01161-4)
Supplement: Supplementary file 2 — Supplementary Table 2 [file 41597_2022_1161_MOESM2_ESM.pdf]

Supplementary table 2: Raw peaks areas. Note that genotypes bri1-301bak1brl1brl3 and 35S:

Filename	Genotype	Tissue	Condition	Time (day)	Replicate
16075oA_1	Col-0 WT	shoot	water	0	1
16075oA_2	Col-0 WT	shoot	water	0	2
16076oA_5	Col-0 WT	shoot	water	0	3
16076oA_6	Col-0 WT	shoot	water	0	4
16076oA_7	Col-0 WT	shoot	water	0	5
16075oA_9	Col-0 WT	shoot	water	1	1
16075oA_10	Col-0 WT	shoot	water	1	2
16076oA_17	Col-0 WT	shoot	water	1	3
16076oA_18	Col-0 WT	shoot	water	1	4
16076oA_19	Col-0 WT	shoot	water	1	5
16075oA_25	Col-0 WT	shoot	water	2	1
16075oA_26	Col-0 WT	shoot	water	2	2
16076oA_41	Col-0 WT	shoot	water	2	3
16076oA_42	Col-0 WT	shoot	water	2	4
16076oA_43	Col-0 WT	shoot	water	2	5
16075oA_41	Col-0 WT	shoot	water	3	1
16075oA_42	Col-0 WT	shoot	water	3	2
16076oA_65	Col-0 WT	shoot	water	3	3
16076oA_66	Col-0 WT	shoot	water	3	4
16076oA_67	Col-0 WT	shoot	water	3	5
16075oA_57	Col-0 WT	shoot	water	4	1
16075oA_58	Col-0 WT	shoot	water	4	2
16076oA_89	Col-0 WT	shoot	water	4	3
16076oA_90	Col-0 WT	shoot	water	4	4
16076oA_91	Col-0 WT	shoot	water	4	5
16075oA_73	Col-0 WT	shoot	water	5	1
16075oA_74	Col-0 WT	shoot	water	5	2
16076oA_113	Col-0 WT	shoot	water	5	3
16076oA_114	Col-0 WT	shoot	water	5	4
16076oA_115	Col-0 WT	shoot	water	5	5
16075oA_89	Col-0 WT	shoot	water	6	1
16075oA_90	Col-0 WT	shoot	water	6	2
16076oA_137	Col-0 WT	shoot	water	6	3
16076oA_138	Col-0 WT	shoot	water	6	4
16076oA_139	Col-0 WT	shoot	water	6	5
16075oA_11	Col-0 WT	shoot	drought	1	1
16075oA_12	Col-0 WT	shoot	drought	1	2
16076oA_20	Col-0 WT	shoot	drought	1	3
16076oA_21	Col-0 WT	shoot	drought	1	4
16076oA_22	Col-0 WT	shoot	drought	1	5
16075oA_27	Col-0 WT	shoot	drought	2	1
16075oA_28	Col-0 WT	shoot	drought	2	2
16076oA_44	Col-0 WT	shoot	drought	2	3
16076oA_45	Col-0 WT	shoot	drought	2	4
16076oA_46	Col-0 WT	shoot	drought	2	5
16075oA_43	Col-0 WT	shoot	drought	3	1
16075oA_44	Col-0 WT	shoot	drought	3	2
16076oA_68	Col-0 WT	shoot	drought	3	3
16076oA_69	Col-0 WT	shoot	drought	3	4
16076oA_70	Col-0 WT	shoot	drought	3	5
16075oA_59	Col-0 WT	shoot	drought	4	1

16075oA_60	Col-0 WT	shoot	drought	4	2
16076oA_92	Col-0 WT	shoot	drought	4	3
16076oA_93	Col-0 WT	shoot	drought	4	4
16076oA_94	Col-0 WT	shoot	drought	4	5
16075oA_75	Col-0 WT	shoot	drought	5	1
16075oA_76	Col-0 WT	shoot	drought	5	2
16076oA_116	Col-0 WT	shoot	drought	5	3
16076oA_117	Col-0 WT	shoot	drought	5	4
16076oA_118	Col-0 WT	shoot	drought	5	5
16075oA_91	Col-0 WT	shoot	drought	6	1
16075oA_92	Col-0 WT	shoot	drought	6	2
16076oA_140	Col-0 WT	shoot	drought	6	3
16076oA_141	Col-0 WT	shoot	drought	6	4
16076oA_142	Col-0 WT	shoot	drought	6	5
16075oA_5	35S:BRL3-GFP	shoot	water	0	1
16075oA_6	35S:BRL3-GFP	shoot	water	0	2
16076oA_11	35S:BRL3-GFP	shoot	water	0	3
16076oA_12	35S:BRL3-GFP	shoot	water	0	4
16076oA_13	35S:BRL3-GFP	shoot	water	0	5
16075oA_17	35S:BRL3-GFP	shoot	water	1	1
16075oA_18	35S:BRL3-GFP	shoot	water	1	2
16076oA_29	35S:BRL3-GFP	shoot	water	1	3
16076oA_30	35S:BRL3-GFP	shoot	water	1	4
16076oA_31	35S:BRL3-GFP	shoot	water	1	5
16075oA_33	35S:BRL3-GFP	shoot	water	2	1
16075oA_34	35S:BRL3-GFP	shoot	water	2	2
16076oA_53	35S:BRL3-GFP	shoot	water	2	3
16076oA_54	35S:BRL3-GFP	shoot	water	2	4
16076oA_55	35S:BRL3-GFP	shoot	water	2	5
16075oA_49	35S:BRL3-GFP	shoot	water	3	1
16075oA_50	35S:BRL3-GFP	shoot	water	3	2
16076oA_77	35S:BRL3-GFP	shoot	water	3	3
16076oA_78	35S:BRL3-GFP	shoot	water	3	4
16076oA_79	35S:BRL3-GFP	shoot	water	3	5
16075oA_65	35S:BRL3-GFP	shoot	water	4	1
16075oA_66	35S:BRL3-GFP	shoot	water	4	2
16076oA_101	35S:BRL3-GFP	shoot	water	4	3
16076oA_102	35S:BRL3-GFP	shoot	water	4	4
16076oA_103	35S:BRL3-GFP	shoot	water	4	5
16075oA_81	35S:BRL3-GFP	shoot	water	5	1
16075oA_82	35S:BRL3-GFP	shoot	water	5	2
16076oA_125	35S:BRL3-GFP	shoot	water	5	3
16076oA_126	35S:BRL3-GFP	shoot	water	5	4
16076oA_127	35S:BRL3-GFP	shoot	water	5	5
16075oA_97	35S:BRL3-GFP	shoot	water	6	1
16075oA_98	35S:BRL3-GFP	shoot	water	6	2
16076oA_149	35S:BRL3-GFP	shoot	water	6	3
16076oA_150	35S:BRL3-GFP	shoot	water	6	4
16076oA_151	35S:BRL3-GFP	shoot	water	6	5
16075oA_19	35S:BRL3-GFP	shoot	drought	1	1
16075oA_20	35S:BRL3-GFP	shoot	drought	1	2
16076oA_32	35S:BRL3-GFP	shoot	drought	1	3
16076oA_33	35S:BRL3-GFP	shoot	drought	1	4

16076oA_34	35S:BRL3-GFP	shoot	drought	1	5
16075oA_35	35S:BRL3-GFP	shoot	drought	2	1
16075oA_36	35S:BRL3-GFP	shoot	drought	2	2
16076oA_56	35S:BRL3-GFP	shoot	drought	2	3
16076oA_57	35S:BRL3-GFP	shoot	drought	2	4
16076oA_58	35S:BRL3-GFP	shoot	drought	2	5
16075oA_51	35S:BRL3-GFP	shoot	drought	3	1
16075oA_52	35S:BRL3-GFP	shoot	drought	3	2
16076oA_80	35S:BRL3-GFP	shoot	drought	3	3
16076oA_81	35S:BRL3-GFP	shoot	drought	3	4
16076oA_82	35S:BRL3-GFP	shoot	drought	3	5
16075oA_67	35S:BRL3-GFP	shoot	drought	4	1
16075oA_68	35S:BRL3-GFP	shoot	drought	4	2
16076oA_104	35S:BRL3-GFP	shoot	drought	4	3
16076oA_105	35S:BRL3-GFP	shoot	drought	4	4
16076oA_106	35S:BRL3-GFP	shoot	drought	4	5
16075oA_83	35S:BRL3-GFP	shoot	drought	5	1
16075oA_84	35S:BRL3-GFP	shoot	drought	5	2
16076oA_128	35S:BRL3-GFP	shoot	drought	5	3
16076oA_129	35S:BRL3-GFP	shoot	drought	5	4
16076oA_130	35S:BRL3-GFP	shoot	drought	5	5
16075oA_99	35S:BRL3-GFP	shoot	drought	6	1
16075oA_100	35S:BRL3-GFP	shoot	drought	6	2
16076oA_152	35S:BRL3-GFP	shoot	drought	6	3
16076oA_153	35S:BRL3-GFP	shoot	drought	6	4
16076oA_154	35S:BRL3-GFP	shoot	drought	6	5
16075oA_3	bri1-301bak1brl1brl3	shoot	water	0	1
16075oA_4	bri1-301bak1brl1brl3	shoot	water	0	2
16076oA_8	bri1-301bak1brl1brl3	shoot	water	0	3
16076oA_9	bri1-301bak1brl1brl3	shoot	water	0	4
16076oA_10	bri1-301bak1brl1brl3	shoot	water	0	5
16075oA_13	bri1-301bak1brl1brl3	shoot	water	1	1
16075oA_14	bri1-301bak1brl1brl3	shoot	water	1	2
16076oA_23	bri1-301bak1brl1brl3	shoot	water	1	3
16076oA_24	bri1-301bak1brl1brl3	shoot	water	1	4
16076oA_25	bri1-301bak1brl1brl3	shoot	water	1	5
16075oA_29	bri1-301bak1brl1brl3	shoot	water	2	1
16075oA_30	bri1-301bak1brl1brl3	shoot	water	2	2
16076oA_47	bri1-301bak1brl1brl3	shoot	water	2	3
16076oA_48	bri1-301bak1brl1brl3	shoot	water	2	4
16076oA_49	bri1-301bak1brl1brl3	shoot	water	2	5
16075oA_45	bri1-301bak1brl1brl3	shoot	water	3	1
16075oA_46	bri1-301bak1brl1brl3	shoot	water	3	2
16076oA_71	bri1-301bak1brl1brl3	shoot	water	3	3
16076oA_72	bri1-301bak1brl1brl3	shoot	water	3	4
16076oA_73	bri1-301bak1brl1brl3	shoot	water	3	5
16075oA_61	bri1-301bak1brl1brl3	shoot	water	4	1
16075oA_62	bri1-301bak1brl1brl3	shoot	water	4	2
16076oA_95	bri1-301bak1brl1brl3	shoot	water	4	3
16076oA_96	bri1-301bak1brl1brl3	shoot	water	4	4
16076oA_97	bri1-301bak1brl1brl3	shoot	water	4	5
16075oA_77	bri1-301bak1brl1brl3	shoot	water	5	1
16075oA_78	bri1-301bak1brl1brl3	shoot	water	5	2

16076oA_119	bri1-301bak1brl1brl3	shoot	water	5	3
16076oA_120	bri1-301bak1brl1brl3	shoot	water	5	4
16076oA_121	bri1-301bak1brl1brl3	shoot	water	5	5
16075oA_93	bri1-301bak1brl1brl3	shoot	water	6	1
16075oA_94	bri1-301bak1brl1brl3	shoot	water	6	2
16076oA_143	bri1-301bak1brl1brl3	shoot	water	6	3
16076oA_144	bri1-301bak1brl1brl3	shoot	water	6	4
16076oA_145	bri1-301bak1brl1brl3	shoot	water	6	5
16075oA_15	bri1-301bak1brl1brl3	shoot	drought	1	1
16075oA_16	bri1-301bak1brl1brl3	shoot	drought	1	2
16076oA_26	bri1-301bak1brl1brl3	shoot	drought	1	3
16076oA_27	bri1-301bak1brl1brl3	shoot	drought	1	4
16076oA_28	bri1-301bak1brl1brl3	shoot	drought	1	5
16075oA_31	bri1-301bak1brl1brl3	shoot	drought	2	1
16075oA_32	bri1-301bak1brl1brl3	shoot	drought	2	2
16076oA_50	bri1-301bak1brl1brl3	shoot	drought	2	3
16076oA_51	bri1-301bak1brl1brl3	shoot	drought	2	4
16076oA_52	bri1-301bak1brl1brl3	shoot	drought	2	5
16075oA_47	bri1-301bak1brl1brl3	shoot	drought	3	1
16075oA_48	bri1-301bak1brl1brl3	shoot	drought	3	2
16076oA_74	bri1-301bak1brl1brl3	shoot	drought	3	3
16076oA_75	bri1-301bak1brl1brl3	shoot	drought	3	4
16076oA_76	bri1-301bak1brl1brl3	shoot	drought	3	5
16075oA_63	bri1-301bak1brl1brl3	shoot	drought	4	1
16075oA_64	bri1-301bak1brl1brl3	shoot	drought	4	2
16076oA_98	bri1-301bak1brl1brl3	shoot	drought	4	3
16076oA_99	bri1-301bak1brl1brl3	shoot	drought	4	4
16076oA_100	bri1-301bak1brl1brl3	shoot	drought	4	5
16075oA_79	bri1-301bak1brl1brl3	shoot	drought	5	1
16075oA_80	bri1-301bak1brl1brl3	shoot	drought	5	2
16076oA_122	bri1-301bak1brl1brl3	shoot	drought	5	3
16076oA_123	bri1-301bak1brl1brl3	shoot	drought	5	4
16076oA_124	bri1-301bak1brl1brl3	shoot	drought	5	5
16075oA_95	bri1-301bak1brl1brl3	shoot	drought	6	1
16075oA_96	bri1-301bak1brl1brl3	shoot	drought	6	2
16076oA_146	bri1-301bak1brl1brl3	shoot	drought	6	3
16076oA_147	bri1-301bak1brl1brl3	shoot	drought	6	4
16076oA_148	bri1-301bak1brl1brl3	shoot	drought	6	5
15316oA_3	Col-0 WT	root	water	0	1
15316oA_4	Col-0 WT	root	water	0	2
15317oA_1	Col-0 WT	root	water	0	3
15317oA_2	Col-0 WT	root	water	0	4
15317oA_3	Col-0 WT	root	water	0	5
15316oA_11	Col-0 WT	root	water	1	1
15316oA_12	Col-0 WT	root	water	1	2
15317oA_13	Col-0 WT	root	water	1	3
15317oA_14	Col-0 WT	root	water	1	4
15317oA_15	Col-0 WT	root	water	1	5
15316oA_27	Col-0 WT	root	water	2	1
15316oA_28	Col-0 WT	root	water	2	2
15317oA_37	Col-0 WT	root	water	2	3
15317oA_38	Col-0 WT	root	water	2	4
15317oA_39	Col-0 WT	root	water	2	5

15316oA_43	Col-0 WT	root	water	3	1
15316oA_44	Col-0 WT	root	water	3	2
15319oA_19	Col-0 WT	root	water	3	3
15319oA_20	Col-0 WT	root	water	3	4
15319oA_21	Col-0 WT	root	water	3	5
15316oA_56	Col-0 WT	root	water	4	1
15316oA_57	Col-0 WT	root	water	4	2
15319oA_43	Col-0 WT	root	water	4	3
15319oA_44	Col-0 WT	root	water	4	4
15319oA_45	Col-0 WT	root	water	4	5
15316oA_72	Col-0 WT	root	water	5	1
15316oA_73	Col-0 WT	root	water	5	2
15319oA_67	Col-0 WT	root	water	5	3
15319oA_68	Col-0 WT	root	water	5	4
15319oA_69	Col-0 WT	root	water	5	5
15316oA_87	Col-0 WT	root	water	6	1
15316oA_88	Col-0 WT	root	water	6	2
15319oA_91	Col-0 WT	root	water	6	3
15319oA_92	Col-0 WT	root	water	6	4
15319oA_93	Col-0 WT	root	water	6	5
15316oA_13	Col-0 WT	root	drought	1	1
15316oA_14	Col-0 WT	root	drought	1	2
15317oA_16	Col-0 WT	root	drought	1	3
15317oA_17	Col-0 WT	root	drought	1	4
15317oA_18	Col-0 WT	root	drought	1	5
15316oA_29	Col-0 WT	root	drought	2	1
15316oA_30	Col-0 WT	root	drought	2	2
15317oA_40	Col-0 WT	root	drought	2	3
15317oA_41	Col-0 WT	root	drought	2	4
15317oA_42	Col-0 WT	root	drought	2	5
15316oA_45	Col-0 WT	root	drought	3	1
15316oA_46	Col-0 WT	root	drought	3	2
15319oA_22	Col-0 WT	root	drought	3	3
15319oA_23	Col-0 WT	root	drought	3	4
15319oA_24	Col-0 WT	root	drought	3	5
15316oA_58	Col-0 WT	root	drought	4	1
15316oA_59	Col-0 WT	root	drought	4	2
15319oA_46	Col-0 WT	root	drought	4	3
15319oA_47	Col-0 WT	root	drought	4	4
15319oA_48	Col-0 WT	root	drought	4	5
15316oA_74	Col-0 WT	root	drought	5	1
15316oA_75	Col-0 WT	root	drought	5	2
15319oA_70	Col-0 WT	root	drought	5	3
15319oA_71	Col-0 WT	root	drought	5	4
15319oA_72	Col-0 WT	root	drought	5	5
15316oA_89	Col-0 WT	root	drought	6	1
15316oA_90	Col-0 WT	root	drought	6	2
15319oA_94	Col-0 WT	root	drought	6	3
15319oA_95	Col-0 WT	root	drought	6	4
15319oA_96	Col-0 WT	root	drought	6	5
15316oA_7	35S:BRL3-GFP	root	water	0	1
15316oA_8	35S:BRL3-GFP	root	water	0	2
15317oA_7	35S:BRL3-GFP	root	water	0	3

15317oA_8	35S:BRL3-GFP	root	water	0	4
15317oA_9	35S:BRL3-GFP	root	water	0	5
15316oA_19	35S:BRL3-GFP	root	water	1	1
15316oA_20	35S:BRL3-GFP	root	water	1	2
15317oA_25	35S:BRL3-GFP	root	water	1	3
15317oA_26	35S:BRL3-GFP	root	water	1	4
15317oA_27	35S:BRL3-GFP	root	water	1	5
15316oA_35	35S:BRL3-GFP	root	water	2	1
15316oA_36	35S:BRL3-GFP	root	water	2	2
15319oA_7	35S:BRL3-GFP	root	water	2	3
15319oA_8	35S:BRL3-GFP	root	water	2	4
15319oA_9	35S:BRL3-GFP	root	water	2	5
15319oA_31	35S:BRL3-GFP	root	water	3	3
15319oA_32	35S:BRL3-GFP	root	water	3	4
15319oA_33	35S:BRL3-GFP	root	water	3	5
15316oA_64	35S:BRL3-GFP	root	water	4	1
15316oA_65	35S:BRL3-GFP	root	water	4	2
15319oA_55	35S:BRL3-GFP	root	water	4	3
15319oA_56	35S:BRL3-GFP	root	water	4	4
15319oA_57	35S:BRL3-GFP	root	water	4	5
15316oA_80	35S:BRL3-GFP	root	water	5	1
15316oA_81	35S:BRL3-GFP	root	water	5	2
15319oA_79	35S:BRL3-GFP	root	water	5	3
15319oA_80	35S:BRL3-GFP	root	water	5	4
15319oA_81	35S:BRL3-GFP	root	water	5	5
15316oA_95	35S:BRL3-GFP	root	water	6	1
15316oA_96	35S:BRL3-GFP	root	water	6	2
15319oA_103	35S:BRL3-GFP	root	water	6	3
15319oA_104	35S:BRL3-GFP	root	water	6	4
15319oA_105	35S:BRL3-GFP	root	water	6	5
15316oA_21	35S:BRL3-GFP	root	drought	1	1
15316oA_22	35S:BRL3-GFP	root	drought	1	2
15317oA_28	35S:BRL3-GFP	root	drought	1	3
15317oA_29	35S:BRL3-GFP	root	drought	1	4
15317oA_30	35S:BRL3-GFP	root	drought	1	5
15316oA_37	35S:BRL3-GFP	root	drought	2	1
15316oA_38	35S:BRL3-GFP	root	drought	2	2
15319oA_10	35S:BRL3-GFP	root	drought	2	3
15319oA_11	35S:BRL3-GFP	root	drought	2	4
15319oA_12	35S:BRL3-GFP	root	drought	2	5
15316oA_48	35S:BRL3-GFP	root	drought	3	1
15316oA_49	35S:BRL3-GFP	root	drought	3	1
15316oA_50	35S:BRL3-GFP	root	drought	3	2
15319oA_34	35S:BRL3-GFP	root	drought	3	3
15319oA_35	35S:BRL3-GFP	root	drought	3	4
15319oA_36	35S:BRL3-GFP	root	drought	3	5
15316oA_66	35S:BRL3-GFP	root	drought	4	1
15316oA_67	35S:BRL3-GFP	root	drought	4	2
15319oA_58	35S:BRL3-GFP	root	drought	4	3
15319oA_59	35S:BRL3-GFP	root	drought	4	4
15319oA_60	35S:BRL3-GFP	root	drought	4	5
15316oA_82	35S:BRL3-GFP	root	drought	5	1
15316oA_83	35S:BRL3-GFP	root	drought	5	2

15319oA_82	35S:BRL3-GFP	root	drought	5	3
15319oA_83	35S:BRL3-GFP	root	drought	5	4
15319oA_84	35S:BRL3-GFP	root	drought	5	5
15316oA_97	35S:BRL3-GFP	root	drought	6	1
15316oA_98	35S:BRL3-GFP	root	drought	6	2
15319oA_106	35S:BRL3-GFP	root	drought	6	3
15319oA_107	35S:BRL3-GFP	root	drought	6	4
15319oA_108	35S:BRL3-GFP	root	drought	6	5
15316oA_5	bri1-301bak1brl1brl3	root	water	0	1
15316oA_6	bri1-301bak1brl1brl3	root	water	0	2
15317oA_4	bri1-301bak1brl1brl3	root	water	0	3
15317oA_5	bri1-301bak1brl1brl3	root	water	0	4
15317oA_6	bri1-301bak1brl1brl3	root	water	0	5
15316oA_15	bri1-301bak1brl1brl3	root	water	1	1
15316oA_16	bri1-301bak1brl1brl3	root	water	1	2
15317oA_19	bri1-301bak1brl1brl3	root	water	1	3
15317oA_20	bri1-301bak1brl1brl3	root	water	1	4
15317oA_21	bri1-301bak1brl1brl3	root	water	1	5
15316oA_31	bri1-301bak1brl1brl3	root	water	2	1
15316oA_32	bri1-301bak1brl1brl3	root	water	2	2
15317oA_43	bri1-301bak1brl1brl3	root	water	2	3
15319oA_1	bri1-301bak1brl1brl3	root	water	2	3
15319oA_2	bri1-301bak1brl1brl3	root	water	2	4
15319oA_3	bri1-301bak1brl1brl3	root	water	2	5
15316oA_47	bri1-301bak1brl1brl3	root	water	3	1
15319oA_25	bri1-301bak1brl1brl3	root	water	3	3
15319oA_26	bri1-301bak1brl1brl3	root	water	3	4
15319oA_27	bri1-301bak1brl1brl3	root	water	3	5
15316oA_60	bri1-301bak1brl1brl3	root	water	4	1
15316oA_61	bri1-301bak1brl1brl3	root	water	4	2
15319oA_49	bri1-301bak1brl1brl3	root	water	4	3
15319oA_50	bri1-301bak1brl1brl3	root	water	4	4
15319oA_51	bri1-301bak1brl1brl3	root	water	4	5
15316oA_76	bri1-301bak1brl1brl3	root	water	5	1
15316oA_77	bri1-301bak1brl1brl3	root	water	5	2
15319oA_73	bri1-301bak1brl1brl3	root	water	5	3
15319oA_74	bri1-301bak1brl1brl3	root	water	5	4
15319oA_75	bri1-301bak1brl1brl3	root	water	5	5
15316oA_91	bri1-301bak1brl1brl3	root	water	6	1
15316oA_92	bri1-301bak1brl1brl3	root	water	6	2
15319oA_97	bri1-301bak1brl1brl3	root	water	6	3
15319oA_98	bri1-301bak1brl1brl3	root	water	6	4
15319oA_99	bri1-301bak1brl1brl3	root	water	6	5
15316oA_17	bri1-301bak1brl1brl3	root	drought	1	1
15316oA_18	bri1-301bak1brl1brl3	root	drought	1	2
15317oA_22	bri1-301bak1brl1brl3	root	drought	1	3
15317oA_23	bri1-301bak1brl1brl3	root	drought	1	4
15317oA_24	bri1-301bak1brl1brl3	root	drought	1	5
15316oA_33	bri1-301bak1brl1brl3	root	drought	2	1
15316oA_34	bri1-301bak1brl1brl3	root	drought	2	2
15319oA_4	bri1-301bak1brl1brl3	root	drought	2	3
15319oA_5	bri1-301bak1brl1brl3	root	drought	2	4
15319oA_6	bri1-301bak1brl1brl3	root	drought	2	5

15319oA_28	bri1-301bak1brl1brl3	root	drought	3	3
15319oA_29	bri1-301bak1brl1brl3	root	drought	3	4
15319oA_30	bri1-301bak1brl1brl3	root	drought	3	5
15316oA_62	bri1-301bak1brl1brl3	root	drought	4	1
15316oA_63	bri1-301bak1brl1brl3	root	drought	4	2
15319oA_52	bri1-301bak1brl1brl3	root	drought	4	3
15319oA_53	bri1-301bak1brl1brl3	root	drought	4	4
15319oA_54	bri1-301bak1brl1brl3	root	drought	4	5
15316oA_78	bri1-301bak1brl1brl3	root	drought	5	1
15316oA_79	bri1-301bak1brl1brl3	root	drought	5	2
15319oA_76	bri1-301bak1brl1brl3	root	drought	5	3
15319oA_77	bri1-301bak1brl1brl3	root	drought	5	4
15319oA_78	bri1-301bak1brl1brl3	root	drought	5	5
15316oA_93	bri1-301bak1brl1brl3	root	drought	6	1
15316oA_94	bri1-301bak1brl1brl3	root	drought	6	2
15319oA_100	bri1-301bak1brl1brl3	root	drought	6	3
15319oA_101	bri1-301bak1brl1brl3	root	drought	6	4
15319oA_102	bri1-301bak1brl1brl3	root	drought	6	5
15316oA_102	Arabidopsis leaf control	NA	NA		
15319oA_115	Arabidopsis leaf control	NA	NA		
15316oA_103	Blank	NA	NA		
15319oA_116	Blank	NA	NA		

Supplementary_table2

BRL3-GFP are referred in the manuscript as quad and BRL3ox respectively

Weight (mg)	1,6-anhydroglucose	2-methylmalate	2-oxoglutarate	4-hydroxybenzoic_acid
52	5240	195	22	79
51	3054	81	47	53
55	7679	152	88	313
53.3	7880	150	99	327
56	15041	160	94	475
54.3	11212	84	85	305
50.2	12806	157	98	320
55.8	35668	472	144	1259
55.4	16186	216	63	760
55.5	11384	84	43	433
54.1	12813	141	62	762
53.2	13606	228	132	1431
53.5	31793	248	86	1487
55.7	23669	166	52	1128
55.8	5880	71	14	1
51.9	22445	218	69	1459
54	9450	241	10	1071
55.5	23452	181	39	1113
51.5	34274	245	67	1363
56.1	40334	245	55	1357
55.3	16749	175	84	1393
57.4	16801	160	20	1233
55	37250	194	60	1905
55.3	35336	243	66	1838
54.4	38614	252	56	1764
55	32796	277	146	2019
54.1	27919	234	107	1763
53.9	43499	336	191	2150
53.6	47408	174	173	2086
56	84990	334	132	2629
58.3	46898	254	40	2231
54.5	33170	276	79	2070
55.3	58305	173	83	1705
53.8	34698	219	59	1862
54.2	34580	105	69	1738
52	12251	170	46	364
56	8456	134	47	350
53.8	24891	278	116	972
54.5	24597	292	77	906
55.5	27475	409	141	1249
51.1	7776	168	46	1231
56	15564	193	48	988
53.5	59277	463	56	3573
57.2	30135	409	159	2498
50.4	20109	186	54	1091
57	19524	205	21	1171
52.2	38413	252	57	1437
53.6	53717	399	64	2140
56.8	38349	275	44	1857
56.9	56680	287	52	2376
53.3	22300	286	23	1245

57.8	31855	288	10	1375
55.4	49814	349	51	1402
57.5	52024	281	83	2348
56.3	35516	346	65	1590
54.9	558	22	4	777
55	42513	448	86	1870
59	80861	470	99	2594
54.6	45894	392	160	2442
57.4	55037	784	409	2686
54.2	34708	322	118	2166
58.1	43500	496	93	2639
52.6	33720	421	72	2358
54.9	42042	410	165	1813
56.7	35856	493	369	2064
54.6	4037	73	95	135
52.3	7516	72	70	208
54.2	20342	231	224	693
50.9	12669	210	141	645
54.7	18297	206	178	718
52	12700	133	164	406
52	13362	100	45	566
57	19872	169	64	916
55.2	29126	223	98	1067
55.2	25130	263	127	1155
55.9	11521	317	111	993
57.5	28669	426	168	2216
56.5	32516	252	133	1168
53.9	26061	278	147	1335
56.2	17733	189	42	1039
57.8	2630	63	8	789
57.4	6221	124	23	1055
57.7	40334	569	86	4357
55.7	2609	99	32	396
56.6	5840	73	2	625
55.9	7347	46	41	892
52.9	8816	171	72	1107
54.2	33040	244	94	1876
55	30919	261	147	1876
55	42984	469	208	2159
57.5	31172	338	283	2024
53.2	26584	256	135	1848
51.5	28590	249	149	1865
56.3	29509	248	171	1677
56.9	44510	227	168	2054
55.9	31960	172	215	1944
57.9	26971	123	130	2079
56.1	30059	151	42	1541
57.7	27634	174	29	1487
54.1	15863	224	60	1384
55.4	6784	53	33	499
54.8	14034	117	85	715
52.8	24112	126	106	1019
56.6	28293	86	107	1096

56.5	34155	209	174	1249
56.9	22542	146	177	1889
57.2	33400	289	212	2033
55.6	13667	295	168	1065
57.5	25601	288	208	1180
53.5	18545	160	93	1290
54.5	17695	161	46	1186
56.5	9765	139	41	976
52.9	9925	69	13	740
56.1	28621	199	73	1007
56.7	39988	358	80	1660
55.6	11540	93	111	1144
58.2	20107	260	149	1496
57.9	42928	285	135	1829
55.3	49168	448	162	2128
58.4	28418	252	122	1706
58	48900	251	126	1956
52.9	46426	311	131	2126
55.3	60408	174	203	2097
54.9	37583	297	200	2018
58	32019	177	111	1554
57.2	29582	170	68	1924
53.9	55893	249	83	2376
57	59342	488	117	2180
58	49246	327	60	1783
54.8	52879	330	125	2043
55.5	5482	75	97	146
55.5	5111	74	32	120
56	7275	112	69	424
50	7459	102	47	522
55.9	9016	119	92	542
54.5	28886	120	76	709
54.1	14766	101	86	630
56	1330	51	0	667
52.7	19199	224	98	912
52.1	29143	176	78	1069
53.8	8705	109	65	854
54	5258	90	44	769
55.3	9908	110	55	835
56.6	24549	164	128	1052
55.1	20068	129	85	807
56	31290	248	51	1543
54.8	21376	246	62	1440
53.9	12764	200	49	1087
57.8	21425	220	76	1183
55.4	32417	205	52	1699
54.9	14048	126	51	1133
57.2	7871	148	75	1025
56.1	32627	152	22	1292
58	37424	368	42	2337
54.3	29934	308	20	2148
57.8	31188	176	187	2033
53.9	19758	129	96	1567

56.4	53311	205	177	2477
54	39750	198	86	1869
53.5	9583	82	75	1512
57	39362	145	139	2270
54.1	23516	199	170	2051
57.1	38276	175	86	1835
55.4	37419	151	85	1624
51.5	31732	179	135	1670
55	9213	144	44	433
50.8	20685	100	98	484
52	27303	120	23	955
54.6	28659	197	60	949
55.9	32628	136	97	990
58	7452	75	25	657
54.6	17130	301	36	894
52.4	14347	92	20	923
56	17499	97	30	781
54	22626	170	52	940
56.8	22358	196	2	1200
56.4	13833	160	34	1072
54.2	17291	117	73	1627
55.5	1158	12	28	5
56.2	25981	139	35	2930
53.9	30199	191	78	1291
56.1	23702	183	93	1191
58.5	40289	288	48	2404
57.2	38898	398	45	2079
56.1	31321	221	58	1952
57.2	33938	243	89	2007
57.1	27366	270	136	1810
57.4	65937	349	106	2212
53.6	61110	284	140	2141
56.8	38205	160	119	1711
54	65517	151	90	2641
56.2	48250	190	74	2122
55.2	55629	202	57	1934
52.2	59291	205	41	2425
54.1	36785	255	117	1830
34.6	35833	NA	1445	NA
30.6	14743	NA	894	NA
35.5	14299	NA	628	NA
34.1	8959	NA	517	NA
32.9	8027	NA	587	NA
31.3	13705	NA	562	NA
31.4	6132	NA	351	NA
36	13968	NA	2583	NA
32	14066	NA	1953	NA
35.8	10080	NA	2136	NA
35.9	329	NA	5	NA
36.5	32634	NA	1576	NA
34.8	15387	NA	2627	NA
35	9838	NA	4635	NA
36	10625	NA	2768	NA

36	13936	NA	2260	NA
35.1	460	NA	6	NA
31.9	4009	NA	1814	NA
29	1802	NA	1007	NA
27.9	970	NA	266	NA
31.7	15737	NA	2157	NA
26.5	21919	NA	2804	NA
26	5228	NA	2026	NA
33	2914	NA	889	NA
34.1	4419	NA	14	NA
25.5	15796	NA	3264	NA
26.7	26078	NA	2785	NA
16	4857	NA	2813	NA
26	3910	NA	3373	NA
26.1	2907	NA	2102	NA
23	12737	NA	1992	NA
8.1	3533	NA	533	NA
35.2	3439	NA	2029	NA
16.8	2182	NA	1659	NA
29.5	3667	NA	2012	NA
36.9	577	NA	50	NA
34.3	183	NA	42	NA
36	4763	NA	1837	NA
44	11833	NA	2870	NA
36	2688	NA	968	NA
33.3	22343	NA	1856	NA
35.1	16646	NA	1777	NA
32.2	8421	NA	1975	NA
35.3	11091	NA	2066	NA
30.1	9596	NA	2292	NA
34.7	31417	NA	3576	NA
35.7	32031	NA	4719	NA
35	2506	NA	1940	NA
30	3392	NA	2620	NA
35.5	3398	NA	2160	NA
35.1	13461	NA	2984	NA
35	20472	NA	3364	NA
32.6	2692	NA	1876	NA
31.8	3022	NA	2621	NA
32.8	2411	NA	3302	NA
34.2	15295	NA	4353	NA
32.4	13439	NA	2814	NA
34.8	3088	NA	4878	NA
30.5	3684	NA	4596	NA
33.1	3273	NA	5335	NA
32	13774	NA	3967	NA
37.8	31503	NA	6059	NA
36	4272	NA	4941	NA
36.2	3255	NA	5870	NA
32.1	3519	NA	4974	NA
32.4	25001	NA	1319	NA
28.4	14373	NA	1377	NA
33.8	18520	NA	1938	NA

32	7908	NA	1313	NA
30	7292	NA	1350	NA
30	42692	NA	2569	NA
17	812	NA	127	NA
27.5	5956	NA	1206	NA
34	6857	NA	2740	NA
32	7870	NA	2092	NA
31.1	27144	NA	2314	NA
34.5	32191	NA	3199	NA
35.4	6353	NA	2039	NA
35	6973	NA	4222	NA
35	3725	NA	1511	NA
34.1	5771	NA	1998	NA
30	5019	NA	3765	NA
32.3	2486	NA	26	NA
32.3	12067	NA	1967	NA
35	11065	NA	1908	NA
28.7	2272	NA	1539	NA
32.5	2010	NA	1729	NA
34.1	1281	NA	821	NA
27.1	18772	NA	2624	NA
26.2	7298	NA	1571	NA
26	3314	NA	1649	NA
25	2898	NA	1516	NA
26.2	3489	NA	2365	NA
29.5	7552	NA	1426	NA
22.6	19644	NA	1468	NA
10	1912	NA	561	NA
24.5	1005	NA	526	NA
30	1479	NA	810	NA
26.4	12535	NA	779	NA
25	15980	NA	1412	NA
19	6300	NA	1612	NA
35.5	6657	NA	1971	NA
24	4678	NA	1734	NA
35.8	11019	NA	1171	NA
19.5	20040	NA	2301	NA
33.6	708	NA	201	NA
36	1523	NA	994	NA
34.8	1718	NA	1269	NA
35.4	18060	NA	2476	NA
35.4	805	NA	16	NA
34.3	8458	NA	2914	NA
33	2685	NA	2746	NA
31	779	NA	618	NA
35.8	711	NA	626	NA
34.9	9936	NA	2096	NA
34.3	17599	NA	2836	NA
35.3	1889	NA	1702	NA
33.5	1582	NA	1241	NA
36.1	2283	NA	1023	NA
32.2	13271	NA	4633	NA
32.6	8658	NA	1576	NA

33.5	3951	NA	3396	NA
31.5	2584	NA	1698	NA
35	4582	NA	6529	NA
34	23543	NA	2947	NA
24.2	13579	NA	1751	NA
26.1	3844	NA	1471	NA
34.8	3063	NA	2669	NA
15.6	3826	NA	3621	NA
26.9	18701	NA	615	NA
30.7	15836	NA	340	NA
30	6064	NA	648	NA
34	2460	NA	14	NA
30	7159	NA	484	NA
26	16791	NA	1114	NA
26.2	11669	NA	902	NA
5	7304	NA	1268	NA
20	6781	NA	1404	NA
29.3	8852	NA	1983	NA
34	26960	NA	1969	NA
29	24455	NA	1290	NA
14.5	7727	NA	1211	NA
14.5	3744	NA	520	NA
25.6	4420	NA	663	NA
21.9	1519	NA	689	NA
33.6	24397	NA	11	NA
35.5	2710	NA	1395	NA
34.4	2694	NA	1229	NA
35.2	5713	NA	1420	NA
30.9	21517	NA	2264	NA
32.9	24739	NA	2834	NA
30	6498	NA	2076	NA
30.2	3711	NA	2436	NA
36.1	4108	NA	2643	NA
32.1	13447	NA	1914	NA
34.1	17583	NA	3201	NA
29.9	4085	NA	2844	NA
33.5	2978	NA	1892	NA
32	2986	NA	2711	NA
29.6	22299	NA	2039	NA
32.1	14809	NA	1691	NA
31.2	2069	NA	1482	NA
31.5	2242	NA	1488	NA
33.8	2939	NA	1384	NA
31.9	10299	NA	1074	NA
32.5	16999	NA	1198	NA
25.8	4878	NA	1052	NA
35	8181	NA	1421	NA
29	9783	NA	2134	NA
33.9	12382	NA	1042	NA
33.6	26720	NA	3403	NA
35	4242	NA	1693	NA
32	2814	NA	620	NA
35	3430	NA	757	NA

33.8	2008	NA	1023	NA
34.4	2605	NA	1205	NA
32.7	774	NA	10	NA
34.9	17461	NA	1879	NA
30.7	20108	NA	3065	NA
33.3	343	NA	8	NA
34.3	4030	NA	2131	NA
33.1	2401	NA	2269	NA
33.6	11308	NA	1763	NA
32.7	10565	NA	2352	NA
35.2	5032	NA	1758	NA
28.3	5067	NA	1802	NA
27.3	3528	NA	1820	NA
32.5	22318	NA	3106	NA
20.5	10982	NA	1033	NA
28.5	2528	NA	1215	NA
30.3	2983	NA	1787	NA
28	2502	NA	1585	NA
	22018	NA	312	NA
	6287	NA	403	NA
	751	NA	40	NA
	281	NA	13	NA

4-hydroxyproline	Adenine	Alanine	Arginine	Ascorbate	Asparagine	Aspartate	Benzoate
141	87	217	57	7	1886	33079	690
89	91	239	85	23	1338	15761	570
163	157	8764	139	49	773	32682	679
187	79	7252	89	33	654	30464	625
265	291	19992	183	128	814	32178	740
231	179	1193	145	60	1889	13215	633
243	166	1126	182	70	1839	16103	618
639	323	52246	285	711	797	40518	1969
418	119	23933	183	224	316	24754	1131
200	140	7147	151	221	377	17947	519
247	287	908	268	50	3671	19435	640
384	251	3925	558	137	4094	30034	731
284	340	21194	223	426	526	23864	1064
199	187	10473	254	254	631	19766	940
2	12	13	11	4	12	1	25
323	252	6008	270	85	3653	36957	1140
344	204	4432	177	35	1706	25321	956
166	333	21149	259	63	505	31342	887
267	435	24654	281	69	946	54462	992
223	295	33331	228	56	514	37523	1072
266	352	9213	319	115	2723	29862	1908
303	190	6117	203	107	2148	29225	1269
397	365	22781	355	74	800	60590	1170
250	341	30819	397	46	854	44746	1044
248	311	29389	275	68	594	37822	1019
374	471	15271	579	193	5671	38441	1721
345	334	13127	426	128	2387	41034	1614
767	379	67628	560	425	1708	52771	2309
783	702	40352	1544	1990	4251	66004	2057
418	714	83207	306	770	1326	41094	2460
318	360	15585	208	148	2168	38367	2130
375	295	12079	176	123	1815	29884	1547
266	360	38298	145	313	1244	25290	1491
190	281	19804	161	96	737	19743	1201
321	353	37342	412	343	908	19945	1864
169	149	712	139	27	1485	15273	759
190	127	618	88	15	1382	15137	471
381	186	9389	199	412	843	35201	583
392	225	13236	246	126	466	37791	724
419	348	18386	353	564	878	72828	1207
279	178	3113	172	45	2499	19872	2721
257	288	3010	209	98	2213	21156	1172
583	331	33536	270	738	1746	56445	2757
535	299	34471	357	476	810	67096	2115
302	109	21298	128	246	340	27157	1141
411	202	2922	257	6	3178	23130	1298
765	235	3864	297	116	4663	41057	2924
665	473	18030	517	114	4194	61946	1200
476	321	20025	329	44	963	52980	1019
697	356	30236	370	60	1870	51819	1245
859	321	7690	166	65	3752	27520	1131

802	503	6454	325	84	11094	37574	1376
1376	475	21403	543	257	5874	47593	1081
421	497	34656	203	191	814	22854	1468
527	563	31287	477	131	2126	35769	1395
4	66	12	3	25	70	2566	683
800	345	7190	188	221	5204	35334	1328
1041	571	49828	267	622	2129	40863	2226
905	435	30871	264	604	3368	35784	2798
1047	808	18729	361	2057	11481	85891	2977
968	336	8343	206	218	3627	16951	2442
1733	487	8489	355	357	12329	31914	2105
1629	380	26379	137	238	3787	43700	1474
703	363	24621	254	175	2578	34282	2631
1055	497	13390	242	237	6293	62959	2547
229	77	485	111	23	1638	15263	621
309	141	521	114	21	1780	17684	452
463	285	22857	246	102	974	50341	823
519	350	18432	211	87	1901	49469	941
500	338	42653	223	141	998	43026	1608
322	232	1234	137	55	1894	16882	618
444	236	1442	280	27	3354	28259	648
520	286	14312	240	219	794	35196	708
568	305	24205	224	623	962	34358	1604
708	447	22794	407	117	1831	62130	1366
1363	178	8378	243	125	2215	51015	2780
1340	111	10470	225	168	6510	34456	1163
862	214	47635	239	720	558	28645	1189
782	415	40321	345	324	817	46324	886
516	414	30456	279	214	514	40918	1093
109	45	1179	100	17	856	7619	754
478	169	11354	146	47	1935	31214	1231
1416	553	140975	790	101	1172	99044	3525
169	103	11165	113	54	98	11313	293
173	101	25857	72	13	161	15020	757
162	91	2152	78	0	643	9889	775
522	224	8217	108	36	1413	37543	1224
786	341	55315	302	188	903	46943	2197
735	384	56158	393	167	916	54871	1982
1067	541	88520	296	254	1379	79487	2250
895	577	26609	716	136	4679	73997	1825
780	452	25308	488	100	2752	49585	1560
679	319	108141	395	78	1062	40610	1629
836	415	125697	226	181	693	39814	2139
894	516	88621	893	190	1622	49816	1181
760	256	22897	201	316	1275	31266	1781
757	395	38453	484	423	2403	44264	1848
427	238	62975	153	88	463	30854	2346
579	304	60725	115	370	631	35290	2107
446	287	30576	161	222	661	55503	1690
294	188	924	111	13	2707	23904	771
453	292	1369	186	44	3599	29734	705
574	308	22853	185	354	519	37417	963
613	269	21995	164	358	1107	46841	868

820	423	41034	275	437	880	54079	1241
1064	251	4601	206	56	7003	57627	1473
1284	198	11356	238	160	6763	58437	791
590	437	34923	224	98	692	74581	972
641	294	45256	240	199	878	53476	1099
527	428	19857	342	47	626	57826	859
1289	339	13762	296	114	2571	42356	3548
680	222	5027	163	51	1781	20602	897
318	91	11528	118	4	156	14130	1008
966	274	28722	470	36	772	51301	866
1554	394	36121	598	41	1257	84858	1214
787	247	3271	142	42	3052	35148	730
957	443	7287	246	96	5341	41299	996
1141	486	68730	275	225	1293	56359	1764
1950	569	74971	417	244	2326	73202	2064
1161	381	41113	266	152	1388	49646	1516
2298	388	13172	254	125	4955	22902	1728
2073	451	22001	233	59	4368	36583	1963
1676	579	43172	335	186	2124	58249	1201
1913	478	56331	309	217	1195	55574	1449
1433	493	21439	359	166	3573	38135	1065
3140	283	16191	305	304	4530	38441	1581
2145	371	11448	294	585	6317	18961	1625
4531	587	72705	221	879	3195	61643	1984
2024	387	39777	233	795	2060	30398	1557
1037	465	89310	308	492	1498	46109	2064
100	181	305	196	5	2706	28491	638
86	174	375	143	26	1443	16090	677
97	153	10649	127	34	532	34148	631
122	102	9261	126	65	379	17667	1003
112	149	10247	185	72	1020	26687	2005
135	346	1150	367	118	5409	21968	4195
127	226	1096	293	21	3235	27583	2423
0	68	924	92	35	764	17285	614
216	169	18666	186	394	587	33009	1698
265	317	18560	246	382	911	40494	845
174	159	1723	168	62	1702	20558	778
114	168	1183	180	20	2045	20331	779
156	114	11301	108	96	191	16769	690
233	198	15461	237	374	382	44476	572
156	165	12060	249	223	462	28381	469
192	248	5781	317	59	3210	42379	1640
247	250	6376	276	63	2780	39117	1414
171	294	19496	243	50	413	45702	2325
202	303	39195	333	17	587	53690	1068
178	381	29749	429	81	774	52278	1192
126	226	7113	114	116	1391	28171	956
157	195	5840	238	80	1301	29511	767
144	397	37357	428	99	678	28442	1265
238	440	53004	494	161	1467	79535	2113
321	369	66993	326	88	1546	46555	1981
237	350	16662	674	64	3896	59018	1657
259	362	9186	533	84	4029	49597	1558

308	561	75146	933	683	2432	59934	2193
236	335	55262	304	562	1357	41573	2578
95	188	11959	296	118	724	25200	1448
325	546	27207	981	360	3239	57591	1683
325	419	24455	1107	225	3090	49687	1503
189	346	95804	279	376	821	30057	2483
265	490	53831	588	298	1251	29616	2339
179	260	64695	142	207	857	31588	1740
114	148	1647	71	39	1002	16053	940
138	192	1246	278	81	2593	28195	716
296	281	16656	284	523	716	39448	761
279	309	23813	148	563	504	41953	780
188	289	22797	207	379	462	35151	820
71	177	2451	141	73	1316	14441	902
269	163	1797	190	70	1934	36490	1058
129	216	7332	114	165	419	25285	744
136	198	16530	91	151	311	21662	658
176	224	21145	190	329	486	35621	753
332	277	7136	369	11	3332	48867	1296
286	183	8509	199	31	2082	29179	1245
203	174	28171	316	34	464	36110	850
29	17	2	6	47	8	7	4
207	293	20440	560	76	467	39084	848
244	293	11741	290	257	2036	31238	1053
292	322	9484	250	148	1844	38803	1167
422	338	75133	368	178	1891	62890	2060
446	206	67323	541	125	1448	62260	1789
290	362	49980	344	112	1388	59984	1744
274	264	12394	354	341	5827	54978	1783
393	360	10873	399	182	4707	52013	1584
419	517	61954	439	1843	1922	88177	1931
244	447	64691	390	1697	1411	65491	2322
216	256	14623	152	1287	660	37801	872
754	483	21790	1159	533	7095	43504	1586
841	405	24931	697	266	6180	52539	1704
221	424	66390	162	542	1554	32679	1512
338	374	42272	223	1019	2165	32106	1803
439	141	65262	271	947	1385	41737	1636
1032	408	10740	62	337	50406	193477	8854
276	286	2199	39	32	16370	59180	3658
135	403	915	55	55	54860	124267	8854
120	195	1073	93	99	42328	115320	7774
135	233	1005	33	43	32458	95425	5959
116	73	2706	39	64	10062	71575	3827
38	168	1479	30	64	7197	46763	2192
307	554	5879	125	232	75842	312959	10766
426	529	6915	123	187	83899	290670	11871
260	376	5082	52	153	58802	220362	9292
9	53	13	19	15	94	17	315
265	497	9504	138	260	27315	180122	11105
302	431	8385	164	178	43657	179772	13152
352	659	6593	131	215	76623	257492	13869
339	498	9681	105	166	46851	214845	13660

222	457	8673	146	375	25854	151187	10258
27	NA	3	5	29	49	22	56
60	254	38128	40	215	2518	96170	4361
71	190	16656	15	116	3004	94555	2862
20	60	4683	8	7667	812	21301	1289
140	375	15043	100	208	13987	125414	9681
223	290	14617	138	235	22114	182221	13690
109	114	28337	79	478	2987	124205	5119
55	125	18249	25	11	1916	69452	4328
17	250	18775	23	217	1431	64287	4064
219	444	11736	93	113	11513	144646	9579
249	540	17272	110	103	17018	167610	12726
220	377	55468	172	134	5218	146357	8291
267	420	49539	161	184	5491	150852	6526
128	219	35347	61	85	2425	103764	6158
109	482	9045	134	80	10232	85532	7750
69	234	1900	4	10	5241	30601	3546
86	549	22579	7	182	2057	68318	6672
126	255	12899	8	49	2776	64635	5839
119	390	30290	82	134	3875	80844	4652
15	19	129	24	9	403	4772	174
19	32	39	14	5	295	3853	58
269	498	3068	67	116	67692	238582	7770
426	778	6550	119	241	83247	417954	10660
213	385	2921	14	59	45613	156223	5329
273	554	8775	208	242	32886	188611	11281
207	365	8496	150	216	26502	157181	11194
344	366	10013	108	210	38226	242767	10408
329	648	13110	120	282	48007	338695	12665
324	417	12299	126	209	53116	316886	12888
522	700	9573	255	363	43584	214594	16361
204	554	NA	108	622	31072	223371	2791
199	196	31427	66	166	6423	199122	4688
161	456	30478	56	733	4005	125978	5554
368	468	44190	81	264	5851	204036	5894
765	481	14948	199	221	47791	170463	17106
679	623	16522	279	204	45020	131638	16462
292	264	28204	105	147	9848	77846	6434
197	347	30113	56	81	9034	84227	5663
209	337	17285	21	45	6280	59340	5102
NA	531	1957	77	311	4269	17312	4226
695	434	15978	194	245	43403	130396	11024
271	348	72456	101	96	5140	125441	8597
225	278	31126	58	168	5918	100041	8889
247	404	20686	110	141	5808	48960	11149
412	668	13283	45	107	21106	42039	11538
1789	1145	29728	108	344	63248	176057	20196
505	771	48852	83	195	12043	64549	8093
380	720	35116	91	196	14582	92697	6921
455	523	26899	48	112	10806	58857	8979
212	362	3728	36	112	27556	124990	8620
263	246	2585	50	165	22282	103721	5432
294	577	3855	22	294	67435	234490	10975

	272	434	2343	34	49	54744	133032	9661
	152	347	2246	50	144	42737	116761	7261
	446	623	11191	156	1663	46857	364522	18018
NA		8	12	33	12	27	26	88
	256	382	2791	43	93	38627	138106	8744
	453	484	4761	76	97	42337	219799	8098
	411	420	8481	131	186	54463	252894	10316
	431	578	14141	157	297	35877	165737	16237
	500	683	25344	151	210	37136	160149	15031
	130	262	84020	26	337	5111	94308	8026
	229	520	148955	50	305	9684	160632	12342
	148	232	22770	61	96	2101	55034	3273
	221	490	50143	33	249	4719	139974	7972
	252	409	119853	101	316	7356	155343	7069
	142	212	23007	17	130	1267	75290	4098
	308	299	10572	137	117	14692	140464	8583
	255	340	7774	97	160	8896	100336	8302
	138	245	20542	102	70	1917	90077	4868
	103	215	26776	32	63	1759	109232	4139
	80	216	16563	57	16	989	56421	2500
	333	474	21196	181	92	17478	154685	12306
	189	317	10188	49	91	8857	97644	6682
	216	284	32855	5	107	3454	92201	5599
	173	213	30436	32	79	2693	83098	5907
	144	394	97539	9	124	6688	152363	7919
	263	473	10671	126	109	16018	199037	8067
	228	749	21219	202	190	18893	273457	11383
	87	220	26112	60	75	1595	100639	4360
	85	162	18628	26	27	1361	71082	2790
	199	261	27444	89	27	2012	95397	4398
	216	375	2860	78	56	12549	94304	7469
	189	329	4801	65	197	12065	143350	7631
	273	381	4182	81	196	27766	146254	8165
	377	346	8774	60	168	39286	169960	9699
	287	258	5948	77	77	27879	129782	6152
	178	253	4388	42	175	17716	68983	7604
	171	465	10998	90	148	22799	133442	11820
	10	54	9352	160	148	814	28684	1524
	87	113	16109	3	67	1073	51335	2294
	99	76	20494	16	117	1320	67737	2707
	900	409	16843	173	284	30802	135490	15282
	36	31	11	10	40	11	380	2442
	640	531	11627	313	103	32983	143853	12840
	396	510	32682	127	105	6172	116032	8047
	151	99	14233	33	59	1082	45785	2562
	251	125	14048	49	22	1393	56898	1716
	617	302	13593	150	106	23989	113933	7345
	599	426	14053	206	333	29450	155009	11397
	214	233	23358	108	135	2454	98393	4242
	303	132	29068	102	47	2730	93131	4270
	243	257	25378	65	181	2858	109808	5725
	846	566	17520	75	163	23599	67714	12910
	663	338	20204	176	181	23092	64207	8567

550	459	52811	60	133	6046	91775	8482
499	402	42429	97	113	4747	72968	6366
765	693	64590	146	153	8275	99863	9897
2744	1780	49599	351	517	43266	224251	14467
489	657	13482	114	180	13562	54743	9474
1360	905	47374	101	203	5986	48709	7309
951	881	59119	109	164	9141	82763	9357
1062	1228	65853	147	186	7991	96770	12354
194	212	1817	80	116	17984	61594	4360
166	59	916	66	17	11543	33845	3954
41	377	532	11	6	20663	37950	4610
41	102	275	5	35	9401	17163	5230
65	207	788	37	47	21061	37234	5828
90	212	3996	74	97	10807	74818	10388
104	112	4178	20	40	15299	77352	8436
141	251	2468	26	86	29341	86683	9349
151	230	3324	31	134	39147	120736	7654
194	318	5419	52	309	51074	167135	9753
197	423	12615	194	320	35946	273433	10610
130	399	8487	111	215	25624	223574	11096
168	296	5733	39	126	25985	162379	8813
82	130	27981	15	64	1548	63635	3944
184	97	32516	46	167	1899	84446	5522
131	98	40030	47	108	2207	61162	4245
110	413	10053	93	346	17293	182038	11780
61	274	27243	5	201	2890	120417	3421
62	299	15078	48	253	4571	152445	3474
36	256	45096	82	299	4241	236953	4243
224	368	11818	136	137	23916	135858	10744
212	399	15063	154	219	28687	221791	12783
109	238	33264	117	410	6360	124196	5707
111	258	40528	102	224	5843	132226	5251
177	394	87593	152	368	8397	300973	6229
138	324	11270	93	162	17613	97879	6726
308	516	18189	245	463	45428	183405	8747
146	293	37338	135	235	5898	177320	5139
140	263	57732	145	193	5019	141534	5199
118	331	34414	160	147	3949	138147	5197
254	441	14541	226	165	28795	139336	8599
147	453	11447	151	190	19271	93968	6341
75	239	25484	61	129	2856	76983	3490
111	338	20419	63	99	3391	59806	3793
47	241	23515	55	148	2951	80458	3709
144	194	4646	90	104	17461	146838	5121
109	194	5432	95	328	27124	205757	8601
140	204	2872	2	131	25349	100464	4711
155	320	5246	106	232	49175	206730	6594
249	394	6629	68	309	51919	242876	7506
64	283	18355	43	288	12639	97662	6206
281	429	10749	159	294	37683	225913	12089
100	258	84631	27	172	4877	144500	6425
58	96	44664	22	107	3241	88926	5348
38	102	59962	25	110	3239	90100	4939

	85	283	26034	66	141	4415	143503	2838
	61	150	30995	17	229	2194	86952	3032
	9	17	31	16	NA	7	32	11
	272	450	17028	128	189	19888	169330	10523
	344	392	21434	180	289	32041	276498	13115
	16	28	162	30	19	36	395	1583
NA		201	81163	NA	936	4197	29247	1497
	135	530	45546	107	298	6195	205537	4686
	273	396	17085	98	241	18836	144894	9061
	215	454	16295	133	83	23873	194276	7472
	197	293	60724	126	279	4520	163862	6111
	128	362	49427	88	208	4401	169471	6163
	202	278	38946	99	42	3036	126689	5965
	458	790	16575	253	281	46112	177318	13028
	256	505	9417	78	148	22718	68187	6458
	179	322	45809	110	278	4738	110010	5144
	209	270	43135	96	135	5810	121341	5727
	193	279	34598	95	135	6210	132502	4939
	491	367	85365	588	511	3937	21904	2530
	357	191	226441	313	472	1901	17871	2468
	18	55	57	3	16	37	193	1776
	39	4	72	2	31	16	24	1736

Benzylalcohol	beta-Alanine	Citramalate	Citrate	Dehydroascorbate	Erythritol	Erythronate
1355	621	1780	6225	2255	412	290
938	519	907	5087	4838	229	85
1350	908	1092	9382	15109	423	277
1412	881	969	8381	20207	361	224
1414	1322	1113	3767	42275	580	323
877	1189	1353	2683	19361	426	208
895	1071	2155	3666	20692	721	312
3320	2974	2714	5916	136276	2280	614
1809	1452	1422	1095	67557	910	278
1295	656	580	3021	27937	510	185
1304	532	1379	4906	27043	411	214
1321	1533	2736	8472	59827	1260	292
1199	1037	1279	6835	69725	961	310
2407	695	1107	11354	63692	731	330
148	20	12	12	3674	16	9
1252	1407	3490	21348	25274	1073	249
1317	1225	4106	3485	17550	977	300
2373	846	2540	19756	19847	669	242
2476	1247	3043	31868	29047	730	217
2534	1434	3549	24031	30519	1098	296
1382	1012	2497	16127	42489	767	234
1429	780	2479	12398	10608	799	364
1352	930	3245	37351	45960	727	305
1498	854	3275	59745	24210	611	385
1486	1389	6210	15146	26148	1255	323
1792	2371	3629	8557	47051	1267	342
1421	1309	2726	14900	36024	1151	256
2662	2315	2329	10370	87903	1714	390
2499	3148	2158	9968	120522	1725	409
2733	2399	3219	9624	180219	2247	341
2130	1711	3897	21111	24294	1272	303
1880	1534	3757	6141	57374	1177	329
2621	1441	2730	16211	56593	922	264
2352	1039	2403	8352	18970	727	298
2640	1577	1725	2613	116701	1053	120
880	1159	1835	3525	17773	597	227
1053	761	1174	2733	8248	406	309
1079	1735	1277	6216	63728	1131	340
1112	1661	2001	18107	49623	1126	365
1837	2339	1462	35334	55336	1138	665
1180	1227	1326	3527	37548	544	249
1327	1057	1336	4290	32360	637	224
3351	3219	2647	8271	61732	2097	511
3788	2203	2081	22652	68123	1440	612
1908	1355	992	4504	41320	830	216
1643	1739	3589	4715	21007	875	260
1568	2189	4396	16573	41970	1243	322
2700	2866	5173	52838	58620	1353	467
2479	1558	3053	36806	32869	809	304
1356	2259	3259	28532	59418	976	366
1210	1517	3576	7147	13061	897	443

1471	2770	5138	11632	40755	940	371
1553	2960	6444	27959	44174	1043	591
1589	1278	5029	29832	19891	1625	294
2523	2078	5068	37291	56216	1321	562
1563	46	29	4646	50	31	12
1589	3171	5002	41574	57582	1772	723
2706	4414	3717	7059	99150	2231	564
2789	3684	3425	8545	103392	2121	643
2659	5246	4318	93858	162613	2227	915
2068	2688	3840	4666	68696	1845	366
2238	3238	4874	10653	31353	1824	703
2388	2314	4553	21769	18147	1466	774
2473	1961	3122	38481	83384	1303	476
2545	1773	3862	107311	97075	1584	908
1038	729	1175	5187	7508	537	260
1032	724	1158	4367	4804	512	200
2065	1289	1361	19339	42168	863	458
2032	1267	1099	11595	37856	645	275
2180	1805	1224	11768	44586	971	331
868	930	942	1458	28902	496	148
899	1022	1211	2495	11734	483	238
1182	1396	673	4394	24262	709	308
2331	1523	950	7054	41438	713	302
2263	1598	1260	41945	16051	707	485
1292	2529	3042	2703	39111	2317	486
1119	3174	2969	3213	121247	2310	477
1180	1624	1329	2260	40368	1196	370
1186	1189	1270	13517	66949	1105	380
1313	1180	1147	21288	56196	1024	332
1486	308	503	939	2459	158	105
1519	1130	1836	8279	9834	717	290
5018	4232	8275	56286	61698	3573	800
999	319	411	3330	4164	191	64
1254	913	733	3609	8037	322	102
1288	329	450	2113	4994	286	34
1480	910	1825	11582	20623	1029	256
2635	1975	2064	6647	87486	1645	464
2318	1593	1952	9413	69616	1457	355
2930	2554	1778	63436	69309	2248	584
1880	2752	2418	42640	42546	1814	423
2029	1995	2666	21989	31801	1729	433
2662	1865	2984	10834	71223	1434	358
1912	1868	2739	15992	69034	1615	372
2724	2041	3082	4337	127245	2094	359
1915	2184	2623	3353	84140	1478	323
2031	4027	2146	3293	102043	1539	246
2673	1517	1697	16943	43332	812	191
2646	1624	603	41966	61393	939	220
2395	1209	609	122075	41719	647	249
817	727	826	2218	7480	486	146
1212	848	849	1916	24547	477	156
1136	1584	683	4776	73222	917	348
2506	1730	658	6497	60974	825	215

1565	2494	758	8680	95278	1156	372
1148	936	1479	1954	26033	856	504
1265	1634	3505	1425	34023	2440	413
2420	1177	945	47050	21949	979	438
2585	1278	1206	22457	49471	1078	433
2428	902	1386	39392	32331	832	358
1396	2239	2445	10670	31005	1022	329
1093	1193	1561	9498	25241	600	244
1848	543	1332	1675	14825	385	139
880	2182	3248	24665	36690	875	370
1718	2736	3914	44703	44067	1245	544
1350	1281	877	19134	18635	544	329
1292	2038	1549	27387	43012	1014	428
2550	3236	1906	6620	94672	1730	536
2944	4679	2051	17182	118124	2090	577
2619	2891	1309	9733	82227	1175	343
1798	2649	3276	2790	74673	1801	420
1903	3010	4190	13020	58561	1660	456
1374	2285	2204	31132	135501	1199	599
1459	2485	2438	31007	89253	1540	498
1275	2002	2387	13598	90667	1023	426
1977	3985	1670	18743	96319	1383	452
2198	4313	3583	3947	142428	1985	430
2045	3394	1090	45094	114051	1805	916
2240	4286	1424	3600	211891	1870	516
2409	5366	1279	18194	130503	1587	494
1174	581	1072	11538	5376	354	164
1208	462	890	6156	6128	259	144
1462	647	762	21690	23715	365	187
2186	590	980	8517	20660	281	201
1965	707	794	10327	27521	434	198
1211	1401	1267	7136	58901	524	139
1247	771	1074	23716	18258	579	202
2104	232	127	5939	2511	0	35
1361	1149	933	8966	61530	758	285
1041	1490	1395	2743	51239	1451	315
1239	685	663	2878	44208	441	125
1251	608	799	3473	7629	333	128
1644	611	390	955	55941	343	102
655	1622	1030	6057	69024	939	171
682	1190	1052	5258	20510	752	159
1631	1971	3225	23662	21090	752	174
1899	2142	3389	29271	28489	967	255
2426	1569	1991	59095	17895	630	275
1380	2022	2963	52070	35031	894	295
2511	1878	2022	66315	41634	806	283
1415	1122	1312	9729	24395	543	144
1382	1131	1111	15364	20302	493	148
1626	1814	4005	4505	32912	759	196
2748	4225	8598	31890	78694	1383	338
2751	3383	6136	10574	71715	1612	319
1792	2637	1487	40117	32304	940	233
1743	2536	1640	8873	29703	911	244

2698	4993	1633	8972	147918	1622	266
2632	2904	1141	14804	93282	1142	190
2515	842	439	20046	21592	353	167
1987	3085	1868	29816	64325	1250	182
2032	2472	2179	34497	77680	1235	274
2462	2918	2707	17887	145632	1839	183
2572	2348	1984	5830	133747	889	188
2508	2155	1979	27083	58312	909	154
867	1099	1093	3680	24036	494	216
848	1482	1316	14832	25561	572	185
2180	2057	788	2479	89926	754	198
1500	2121	851	10691	97612	865	283
1210	2140	1123	5415	100980	784	164
1244	1484	789	2115	20935	320	81
1306	3354	3621	5469	29293	1523	365
2027	1225	467	8010	33238	519	116
2306	1310	769	4366	21554	520	235
1114	1700	935	5973	66831	737	319
1874	2878	3616	31610	13567	1190	340
1673	2024	2709	13257	25917	913	188
1270	1859	1967	9652	21202	661	173
31	23	17	30	5	7	25
903	1649	1486	39185	58609	571	185
1360	2537	1772	35287	52408	907	198
1264	2209	2033	32517	34636	1033	183
2456	5320	6622	32262	82299	1880	343
2456	5085	6902	18825	68576	1533	420
2644	4010	4945	21530	53892	1194	298
1786	4166	2374	33170	59564	962	239
1711	3323	2834	49650	60485	1629	326
2599	4912	1933	91021	95848	1650	305
2844	5153	1972	97027	94120	1677	220
1165	1414	822	58205	45075	523	259
2178	6297	2908	5030	171346	1563	223
2064	6221	5140	32706	95940	1837	317
2564	3865	2647	7031	90823	1365	214
1849	3903	1512	31419	121114	1208	252
2641	5738	1386	11882	181826	1488	278
108	1246	NA	151285	64732	NA	NA
34	878	NA	23931	32691	NA	NA
121	1519	NA	91349	28449	NA	NA
68	1772	NA	55100	23368	NA	NA
73	1738	NA	41890	25098	NA	NA
23	916	NA	40662	27551	NA	NA
18	458	NA	37222	17799	NA	NA
96	3191	NA	217893	133489	NA	NA
31	3050	NA	183994	110973	NA	NA
117	3077	NA	138898	78117	NA	NA
298	14	NA	6	5	NA	NA
118	2131	NA	133354	86002	NA	NA
43	1967	NA	183048	92741	NA	NA
81	2603	NA	259354	79835	NA	NA
62	2160	NA	232163	98199	NA	NA

	69	2231	NA	144312	91933	NA	NA
	202	30	NA	4	47277	NA	NA
	141	1215	NA	126393	83559	NA	NA
	323	982	NA	145647	58349	NA	NA
	131	301	NA	26928	10340	NA	NA
	89	1842	NA	106559	80070	NA	NA
	63	1875	NA	222581	143630	NA	NA
	456	1247	NA	179487	87730	NA	NA
	468	674	NA	88169	50378	NA	NA
	426	721	NA	60083	43011	NA	NA
	26	1622	NA	71776	63048	NA	NA
	102	2095	NA	160914	107896	NA	NA
	84	1602	NA	168556	79409	NA	NA
	60	1807	NA	83176	67396	NA	NA
	75	1060	NA	117846	46858	NA	NA
	37	1380	NA	95557	48820	NA	NA
	47	429	NA	70613	18550	NA	NA
	569	1073	NA	117438	62993	NA	NA
	453	1066	NA	150133	42967	NA	NA
	552	1235	NA	61220	78122	NA	NA
	35	117	NA	2142	1126	NA	NA
	6	46	NA	3100	591	NA	NA
	41	2807	NA	127184	58845	NA	NA
	77	3757	NA	259150	96779	NA	NA
	79	1207	NA	86279	21239	NA	NA
	50	2923	NA	111510	74859	NA	NA
	126	2335	NA	86077	72086	NA	NA
	91	2517	NA	107910	98302	NA	NA
	55	3136	NA	181493	137812	NA	NA
	50	3145	NA	140633	137970	NA	NA
	97	3262	NA	292827	123391	NA	NA
	175	1986	NA	278714	195860	NA	NA
	392	1703	NA	186595	84340	NA	NA
	149	1280	NA	349087	98983	NA	NA
	476	1971	NA	347114	132936	NA	NA
	67	3288	NA	184749	111481	NA	NA
	82	3989	NA	215764	104429	NA	NA
	682	1346	NA	239244	59520	NA	NA
	583	1446	NA	135017	54417	NA	NA
	524	1235	NA	130216	49320	NA	NA
	119	NA	NA	237439	166932	NA	NA
	108	2946	NA	163669	134976	NA	NA
	130	1735	NA	215659	54409	NA	NA
	123	1852	NA	294301	101198	NA	NA
	226	1102	NA	209049	68312	NA	NA
	85	1371	NA	102041	49978	NA	NA
NA		4128	NA	366807	212079	NA	NA
	711	1731	NA	150909	127893	NA	NA
	620	1978	NA	114581	101597	NA	NA
	654	1275	NA	156769	94335	NA	NA
	59	1119	NA	107365	63705	NA	NA
	61	923	NA	111532	53921	NA	NA
	102	2577	NA	158637	150262	NA	NA

47	1660	NA	75035	17429	NA	NA
62	1778	NA	75827	43874	NA	NA
63	3023	NA	357016	214449	NA	NA
171	9	NA	45	72691	NA	NA
34	1965	NA	78837	46914	NA	NA
65	2460	NA	107003	64052	NA	NA
128	2476	NA	153926	96156	NA	NA
60	1684	NA	283685	150448	NA	NA
131	2433	NA	189949	157314	NA	NA
9252	642	NA	207792	125113	NA	NA
6188	714	NA	287754	220043	NA	NA
325	629	NA	61139	57925	NA	NA
223	2287	NA	119037	92491	NA	NA
626	1459	NA	93391	92201	NA	NA
151	902	NA	54011	38717	NA	NA
48	1349	NA	91179	74261	NA	NA
48	1106	NA	57287	50650	NA	NA
54	833	NA	66103	39701	NA	NA
120	919	NA	60619	43021	NA	NA
96	671	NA	21755	20260	NA	NA
59	1891	NA	102445	80103	NA	NA
99	1422	NA	63424	62372	NA	NA
396	1054	NA	73091	50930	NA	NA
454	1050	NA	68917	48901	NA	NA
860	844	NA	122168	29536	NA	NA
75	2146	NA	132478	51946	NA	NA
71	2985	NA	123893	115668	NA	NA
145	753	NA	85902	30699	NA	NA
119	860	NA	30023	15993	NA	NA
123	1075	NA	42207	23519	NA	NA
202	1449	NA	36781	26602	NA	NA
128	1837	NA	46624	48385	NA	NA
48	2028	NA	88485	76977	NA	NA
45	3023	NA	56434	97028	NA	NA
44	2074	NA	45428	31143	NA	NA
118	835	NA	63214	45228	NA	NA
61	1518	NA	141124	89373	NA	NA
204	144	NA	23837	10376	NA	NA
147	501	NA	41920	33021	NA	NA
436	552	NA	40803	39407	NA	NA
145	2769	NA	182990	110806	NA	NA
61	38	NA	83	16	NA	NA
110	3003	NA	92983	58977	NA	NA
420	2141	NA	131146	62379	NA	NA
60	752	NA	53343	20639	NA	NA
181	739	NA	40184	24451	NA	NA
108	2552	NA	70941	67106	NA	NA
53	2248	NA	145660	101886	NA	NA
106	1361	NA	69033	40635	NA	NA
135	1295	NA	110524	45083	NA	NA
23	1349	NA	111903	49701	NA	NA
55	1528	NA	159411	91400	NA	NA
36	1913	NA	100283	76763	NA	NA

577	1644	NA	205244	98651	NA	NA
418	1219	NA	93152	63188	NA	NA
495	1845	NA	190983	113876	NA	NA
40	4836	NA	286348	267559	NA	NA
82	1607	NA	87894	61305	NA	NA
145	1087	NA	174184	102975	NA	NA
218	1999	NA	151466	70964	NA	NA
1488	1916	NA	184588	86651	NA	NA
34	953	NA	37722	53512	NA	NA
80	649	NA	19802	29283	NA	NA
125	1053	NA	16444	6044	NA	NA
110	483	NA	7648	738	NA	NA
80	1181	NA	11817	10481	NA	NA
52	915	NA	64447	73325	NA	NA
91	973	NA	47287	39881	NA	NA
141	1415	NA	66299	61188	NA	NA
93	1674	NA	70715	79108	NA	NA
29	2231	NA	100632	109959	NA	NA
51	2810	NA	123058	124992	NA	NA
127	1943	NA	136018	113866	NA	NA
15	1818	NA	85479	79992	NA	NA
6388	738	NA	45836	45630	NA	NA
10376	801	NA	72300	64342	NA	NA
10194	484	NA	23742	43394	NA	NA
218	2645	NA	131971	103360	NA	NA
120	1391	NA	102594	74804	NA	NA
422	1642	NA	104333	86348	NA	NA
75	2383	NA	150053	142808	NA	NA
100	2720	NA	52536	112042	NA	NA
104	3226	NA	146040	165252	NA	NA
778	2379	NA	72010	133358	NA	NA
534	2164	NA	86282	89059	NA	NA
231	4252	NA	198490	141606	NA	NA
50	2016	NA	29593	76518	NA	NA
70	3383	NA	38958	117923	NA	NA
63	2792	NA	45000	108490	NA	NA
67	2267	NA	35852	88155	NA	NA
131	1835	NA	80515	70640	NA	NA
15	2242	NA	58495	112129	NA	NA
42	1737	NA	45056	75273	NA	NA
104	954	NA	38343	60483	NA	NA
239	952	NA	42137	60033	NA	NA
169	1169	NA	73768	68509	NA	NA
17	1400	NA	53502	50242	NA	NA
13	1713	NA	80609	71999	NA	NA
53	1674	NA	29465	51040	NA	NA
91	2738	NA	90821	89527	NA	NA
77	3189	NA	98639	151819	NA	NA
65	1235	NA	62106	45587	NA	NA
32	3723	NA	226131	176925	NA	NA
6569	1317	NA	101398	131327	NA	NA
10027	857	NA	71022	65536	NA	NA
10663	798	NA	67771	76779	NA	NA

333	1614	NA	81883	56926	NA	NA
137	1150	NA	56787	69463	NA	NA
52	38	NA	45	16	NA	NA
NA	3348	NA	98693	139714	NA	NA
146	4590	NA	152219	160623	NA	NA
79	24	NA	220	15	NA	NA
NA	209	NA	137529	33086	NA	NA
113	3143	NA	133042	103899	NA	NA
77	3727	NA	81138	98631	NA	NA
45	2943	NA	163801	103361	NA	NA
130	2743	NA	121063	79076	NA	NA
60	2881	NA	125250	85541	NA	NA
124	2000	NA	90214	58133	NA	NA
98	3764	NA	130107	196023	NA	NA
49	1738	NA	65173	79916	NA	NA
159	1665	NA	94814	96180	NA	NA
152	2125	NA	122736	87575	NA	NA
475	2264	NA	150616	84869	NA	NA
50	1369	NA	2907	69950	NA	NA
51	1053	NA	2468	43599	NA	NA
62	21	NA	3	11	NA	NA
51	26	NA	7	43	NA	NA

Ethanolamine	Fructose	Fructose-6-phosphate	Fumarate	GABA	Galactinol	Galactose	Glucose
21483	682	NA	611483	10907	5901	139	9820
24202	834	NA	124050	3969	7547	83	8229
70017	2097	NA	619982	19044	20313	283	24743
48872	1127	NA	626734	10640	14899	230	16360
51482	1766	NA	623072	17433	29541	382	27611
30629	2324	NA	677466	15553	34392	324	31915
31401	2974	NA	871288	17554	38323	368	37829
88863	12194	NA	1188142	49624	73273	1290	92350
32992	2734	NA	773881	14122	24096	492	32623
86142	4255	NA	690891	6986	38908	394	35663
48769	4348	NA	516058	18846	46453	459	60818
50768	5119	NA	823567	23060	68825	738	74210
26488	4237	NA	817677	15600	88241	675	52726
25427	3332	NA	642457	8587	61699	579	47427
29	0	NA	4	2	13	14	16
58410	3542	NA	712282	23461	55954	470	62868
20671	3450	NA	790537	13350	40924	431	41837
32287	2839	NA	478326	16885	48577	328	28576
120042	2478	NA	596787	23669	67657	456	42435
100831	3347	NA	626859	29504	88878	488	48282
31944	2751	NA	529828	16304	56450	441	42497
25633	2812	NA	685311	7870	50897	472	38962
28364	3258	NA	461229	28678	66609	541	39987
29313	4229	NA	568960	17306	68614	742	50665
16499	3509	NA	394797	20607	51822	569	39057
116851	4510	NA	535208	47945	80000	593	63261
44221	3658	NA	590289	15826	59759	497	45883
48098	9201	NA	824815	33338	67889	1183	165767
113146	12794	NA	551294	40499	108441	1333	196253
83173	7966	NA	900873	40470	108953	1149	91266
38075	2822	NA	532430	29049	68396	640	52491
24730	3179	NA	709024	12490	61803	661	39567
86366	3572	NA	411661	31421	90745	851	69544
36600	2417	NA	488762	17251	64422	624	37251
35046	4549	NA	246719	32179	75501	824	96810
21916	9544	NA	734428	9381	35630	301	45708
27816	2343	NA	736724	7588	27513	236	26667
50422	16807	NA	849682	27586	44365	556	53906
40716	5790	NA	897094	23925	69158	613	49050
69181	7428	NA	1215236	22724	55074	646	44971
45467	2392	NA	904244	19304	58576	424	43119
62814	3271	NA	916371	12801	64035	338	30842
88051	5404	NA	1381517	46651	100093	1112	46878
60139	4227	NA	1207171	25312	46121	680	51504
44522	2134	NA	781072	17035	15992	352	25654
29749	6384	NA	775165	16879	51531	856	81234
38576	3977	NA	1250901	30324	70327	746	55618
158926	5836	NA	1351635	45783	143746	1256	86414
43073	4246	NA	1056264	17034	73138	798	41928
83283	4700	NA	1160865	38475	105829	1042	73891
39019	7695	NA	1348605	23818	73091	1142	123281

55202	20059	NA	1488851	35634	81003	1062	239237
38327	42127	NA	1889920	40741	130051	4388	403036
43442	17592	NA	1103759	31200	113897	3123	148121
91844	37582	NA	1259107	34734	169970	5869	294530
851	116	NA	37071	317	386	2	503
45555	44939	NA	1903652	27827	100699	10125	398930
108276	8445	NA	1330836	31641	92890	2171	84996
55459	34309	NA	1619990	38445	114886	10057	344101
139077	28356	NA	2813866	60538	138057	8446	344700
34611	19827	NA	1432136	37315	81813	5453	358371
53751	56345	NA	1958133	45398	117592	7273	476566
45415	63415	NA	1708478	49095	102690	8150	568398
80567	11506	NA	1576122	54773	110011	3281	218028
111102	42305	NA	2363643	42996	136542	14294	531881
13872	1264	NA	490448	5057	13954	196	22643
21582	1793	NA	434499	8070	14795	256	26919
70856	4087	NA	657667	17122	50999	677	44412
117742	2365	NA	430583	27517	51724	476	47386
66472	3603	NA	555594	20177	51119	595	52167
24885	1314	NA	384443	10484	32233	223	16902
27647	2743	NA	227848	13240	31364	389	25699
41650	3138	NA	275041	12053	35934	435	52375
75338	3493	NA	534984	30568	52727	715	38966
97612	3785	NA	459486	23338	57992	754	43364
30736	6616	NA	507501	21164	92885	438	69031
39242	9639	NA	972941	29728	86576	837	92051
47309	5810	NA	539399	20970	70845	759	47681
37613	4350	NA	513032	25843	61211	696	55880
36985	4042	NA	691533	16331	62754	614	50536
9668	679	NA	112121	3446	6943	81	6231
48665	3173	NA	426676	17614	36887	394	39510
107519	8969	NA	1299975	56691	35013	1574	101711
15683	480	NA	121388	2838	7089	142	6105
18767	732	NA	225655	6004	15667	150	10625
5945	630	NA	179153	2866	10797	133	5915
46035	2595	NA	486672	20244	51817	393	41772
53330	7365	NA	857748	20935	71028	942	64213
48812	5016	NA	755084	24585	66756	707	48356
53818	5627	NA	989452	30009	81909	1090	62234
82141	7749	NA	726673	29514	76355	938	77425
43129	4053	NA	614618	18247	65852	764	46012
43460	5653	NA	657389	20957	74126	825	63497
40215	5441	NA	793171	24822	88266	866	61806
61199	9481	NA	486208	41052	105638	1173	96835
27203	2714	NA	901053	30045	66009	758	46123
98675	3256	NA	468672	49191	82134	921	61260
45162	2741	NA	689390	30480	47878	626	43710
28375	2127	NA	594142	22483	55974	663	29531
34792	1586	NA	453532	19544	38186	422	23444
19108	1472	NA	208172	7612	20367	240	18446
28264	3039	NA	339473	13412	36503	503	31053
34149	2882	NA	483150	15797	49670	652	36723
81573	4485	NA	441267	26847	72633	464	34983

84557	4786	NA	706748	25579	79425	892	56045
47551	2572	NA	887923	22732	44674	351	37063
32113	2606	NA	702599	23664	83632	382	35281
165833	3485	NA	681543	10952	62491	418	44489
70580	3887	NA	779616	21193	70306	583	49108
40977	2925	NA	565692	12644	46393	436	42715
43490	9331	NA	1039150	31191	76481	839	100424
22422	4365	NA	700163	16371	50373	697	69821
11586	1607	NA	334032	7130	21956	278	16880
27822	10144	NA	673100	33828	70335	855	84467
38894	8925	NA	887216	36788	91731	1134	65600
27333	10297	NA	805026	14861	37936	1059	120105
68103	22955	NA	1009366	21974	82972	1541	181084
67395	7050	NA	1147466	34053	77582	1257	72483
120657	12466	NA	1245303	61137	101561	1882	118980
81296	5204	NA	917738	34137	60371	843	47440
44263	43994	NA	1112133	41375	93416	5197	463147
73285	17662	NA	1322999	56946	101346	3735	246804
35458	32011	NA	1184172	43865	125992	4284	261360
41027	33003	NA	1147387	42954	110501	2218	329429
87745	31020	NA	1524743	49852	147881	3833	343651
43279	32291	NA	1331959	50458	61052	5748	507311
51012	19387	NA	1008790	57524	112524	7519	337975
57025	47482	NA	1766872	70803	105759	2564	898852
75008	51980	NA	1448012	61178	109608	7811	493204
107341	9746	NA	1222835	48897	126464	1602	117924
40378	883	NA	169144	4682	10418	175	11645
37765	724	NA	241312	6730	10999	112	12440
38483	2626	NA	210392	7513	19476	228	18016
23566	2028	NA	197347	7528	24552	220	14019
39477	1363	NA	171503	4474	28540	244	17104
86725	3760	NA	401859	12570	56916	425	35209
31403	3271	NA	331649	9704	32494	329	24343
30761	311	NA	40256	7481	8414	54	1697
37798	2360	NA	351490	18063	38315	324	24437
33750	7220	NA	338917	20112	43622	462	47069
28299	1084	NA	335023	7212	37910	160	14882
20790	1185	NA	192909	4229	31765	211	11053
21503	779	NA	198845	11424	17222	173	8909
28599	1536	NA	295312	16985	32576	218	20924
29359	1998	NA	243140	11068	54930	277	21500
42288	1721	NA	316036	33779	51784	367	39746
37499	1734	NA	379710	22680	50661	320	31985
35748	1598	NA	441531	18415	46748	289	17975
44187	2913	NA	456808	17351	75581	472	29411
79246	2769	NA	326803	30021	78237	607	36158
39814	1370	NA	247184	20529	34393	211	30632
33175	1222	NA	167403	11522	39935	220	19701
33040	2884	NA	246999	20153	92797	456	32492
72068	3605	NA	548646	44361	94201	676	40610
45822	5152	NA	456974	42303	64217	829	49527
58366	3517	NA	350744	43623	48532	504	53296
53757	2475	NA	255612	28590	50073	404	28340

72979	4671	NA		300691	46986	67463	918	44336
38571	2339	NA		268247	35081	57386	531	25560
14031	773	NA		161615	9646	16322	150	9705
37811	2964	NA		240911	37362	77588	566	46040
43603	3002	NA		300385	36010	69355	499	58477
33152	2556	NA		353571	31221	71621	570	42976
75819	2773	NA		267816	44412	87868	592	41871
48016	2530	NA		324233	38062	67190	429	34931
15096	1423	NA		463967	7894	27857	211	11261
21965	2409	NA		238463	10906	30684	327	23567
67028	1741	NA		255405	15804	43458	371	26923
41332	2426	NA		418203	21276	45014	455	29843
30025	2551	NA		266077	16459	46987	451	43456
31050	805	NA		214100	8323	60866	104	12510
25581	1279	NA		615708	24023	42168	354	19505
49383	772	NA		204557	15993	27258	208	11145
27547	923	NA		186212	7140	37669	295	7867
21127	1424	NA		305940	18398	56842	322	15352
43472	1710	NA		750422	21258	55068	378	23600
28091	1490	NA		441333	22643	41810	279	27769
25966	1310	NA		390752	15778	44130	319	15632
2	64	NA		16	1	44	21	15
22018	3383	NA		416257	21337	63739	703	36114
45112	2137	NA		471851	30014	61785	457	37097
33206	1820	NA		620209	18649	59630	387	29865
58026	3830	NA		846337	51034	85333	936	45341
41081	8411	NA		865963	36228	73654	732	46537
56859	2226	NA		545054	41583	63302	513	33351
89463	2388	NA		463007	43786	59480	406	43614
64118	4416	NA		822983	42594	69484	623	44572
111188	3676	NA		735275	45376	68934	663	35612
99917	3783	NA		723746	32041	64792	547	37339
14320	2072	NA		636466	17498	48167	365	19438
72118	7200	NA		584115	70716	93480	1165	74396
62659	6101	NA		1145637	66416	83841	2022	84214
75719	3006	NA		515544	44543	89211	743	36730
44828	4696	NA		921444	45327	77178	1407	54605
62900	4075	NA		992170	38682	83965	908	36160
382087	6584		929	11049	604903	93203	1178	237274
281545	5502		258	6208	233524	33037	600	100871
405805	4883		301	7341	484678	28127	646	160703
349885	4279		324	7055	386997	31223	1040	178172
346368	5670		405	5751	344638	30198	874	179880
188038	8052		357	4887	302685	22147	409	94218
134900	7378		415	2710	182402	17111	378	108047
389627	13620		2144	19431	721904	100322	2061	425687
379456	23203		2169	14199	711174	84171	1719	485100
386967	13988		1401	13744	648062	75601	1767	346252
48	8		50	11	11	50	11	8
786386	12914		1038	10166	759924	83204	1143	333923
564678	13771		2191	15865	761957	105689	1673	381214
778855	32001		3148	18105	804524	114713	2635	676076
516955	14534		2644	15115	718923	102122	1532	343090

440015	16353	1762	14729	557753	73030	1175	651447
81	56	6	19	36	23	21	45
218533	17878	902	8746	520559	81725	584	177375
407714	14715	610	4264	325076	55561	502	182437
201208	1114	79	1726	78904	16158	42	10704
350521	21856	1250	12473	450302	73577	1118	248494
376408	14777	1102	16871	563202	81477	1116	307205
259617	12834	610	9061	415547	71761	689	162893
316788	6262	327	3998	287385	49989	327	78154
314547	16798	390	4520	202614	55984	373	185138
349199	7194	1155	15495	525917	74718	868	219791
409716	11067	1383	14284	769787	81387	1024	279173
300897	15526	1046	13890	648215	84679	870	235760
331036	16509	1517	17492	724864	110467	699	201145
293773	11778	759	11797	375738	68443	761	207241
363038	8357	1258	7348	491358	73022	798	139782
348741	1941	332	2503	159622	21118	208	43885
319755	11069	853	6836	434227	84605	646	122949
363890	10541	351	4445	417448	51445	770	182751
324418	21486	1273	6619	486769	78202	685	206052
5767	150	15	390	9998	1510	24	3401
3230	132	6	127	8411	1880	67	14872
643837	9368	1383	11918	534452	64545	1204	386237
379145	15190	1907	19287	701275	93054	2641	482300
441665	8106	560	8448	150999	43194	891	298727
757341	23627	994	13507	530506	68353	1391	477350
441671	15613	847	10679	450017	65715	1368	368025
351922	16227	1147	15844	460078	82486	1860	428469
376819	12952	1568	13847	597193	98574	1850	398688
526356	25739	1198	15897	614064	97446	2048	438694
944917	59315	1094	15838	994658	83103	2962	849102
428580	132756	1036	22140	204001	102785	2731	660138
506840	70715	447	7071	419300	74954	1422	430175
237872	60021	585	7442	474239	69953	1676	478808
328437	123950	906	7874	720029	86118	1579	505283
486374	142557	1111	13440	568064	57216	2692	832885
493180	65183	1496	14834	830530	76323	1912	725127
558824	66020	977	4620	535686	53869	1133	474734
509187	113323	546	5303	425395	57073	1636	590924
452743	286257	608	4553	297970	49990	2105	804549
336971	114739	1183	NA	32988	85332	2493	723882
471307	378865	699	12516	411269	58282	6288	1463999
584446	368695	1191	12598	243110	79501	5225	856207
432079	210918	451	10380	358816	68408	3048	806066
428614	274728	1518	8434	329178	61030	7639	1055174
395732	166994	2123	12614	565765	56220	4356	1077401
526882	363978	2759	35669	1141590	100066	6374	1452340
399709	475367	1744	7549	658344	91132	5748	1085116
432841	515570	966	10075	456560	70499	6600	1053641
379281	482022	1296	13031	394285	71785	8714	1144845
293455	7376	1056	8603	610020	57242	520	125921
343348	2996	938	7949	525206	52440	409	109540
412266	7736	1256	13634	760335	87751	926	145209

560485	5477	766	10657	570802	56156	790	138280
356271	4189	825	9564	537523	54526	625	126950
356041	7339	2086	13772	1242004	116027	812	155967
73	74	10	6	12	171	16	2314
423800	4129	1366	6995	540400	59770	472	113564
383598	4999	1171	16348	681543	89028	1236	185231
394532	7964	1209	13574	672353	89636	1233	208156
410735	17548	2681	14559	896787	91240	770	298265
434089	20699	3547	14340	1002447	104637	1106	245915
246766	16547	1288	10350	148922	111194	625	126800
450269	34875	2916	19593	132798	213218	1209	323071
227477	11920	711	5241	387871	67031	427	118522
490733	20424	1370	14490	520082	101954	845	171206
326559	34472	2240	18412	252898	140884	1273	259691
291542	15378	699	14277	212501	58755	422	93575
367731	7645	924	11650	342018	61285	721	141921
363356	6952	823	10955	386672	58247	602	135020
340279	8671	806	9173	306750	59773	387	138602
275068	9839	390	13038	282913	69520	560	114941
272577	5577	195	5489	149700	40399	336	53893
368547	11403	2437	16230	807889	88514	853	206008
373018	9707	1151	11606	325120	46481	592	187667
273841	12754	1085	7902	345469	55888	484	143944
275710	12372	801	7430	282680	54106	472	139896
344279	17660	1908	12339	157596	66479	850	236477
620651	8098	1015	12362	575898	77416	1006	180798
452383	7266	1961	11832	876982	105211	828	185129
354701	3628	585	5371	216033	52114	312	59194
326893	4748	175	5372	118601	34704	370	54393
333022	6726	292	8928	164268	52613	459	79556
514402	1618	583	7898	341660	36595	354	51153
342612	2257	712	11660	465202	38192	391	81664
315697	2588	1795	10550	515942	67524	558	88752
426833	17985	1666	13624	566182	90529	1309	296707
338343	5241	705	11630	418447	64494	608	106632
381281	4901	699	7391	320701	40349	480	100296
364878	11528	1366	13463	613799	78389	792	194082
184702	1314	169	1512	59219	13088	106	14938
188158	7846	398	6477	207129	44402	368	84380
200787	11238	310	7891	191489	56810	448	161187
602471	45075	1631	12975	748002	80639	1644	519965
365573	84	5	267	23118	16	31	68
726196	96051	1491	18797	517694	65725	2526	789529
745393	50718	1396	12015	524681	89395	1434	353252
294070	11985	411	4396	174367	34063	352	107142
93055	23303	347	3461	119786	25783	470	108989
373978	63253	853	11502	339362	43738	1993	474261
447510	24160	1049	13443	657860	70705	1401	309010
492168	15250	403	8597	474327	64837	638	179616
424227	30795	251	7427	210038	45851	722	207482
305341	12028	298	9317	327965	50551	534	121073
381886	205235	1873	12051	537940	64829	3796	1176136
442588	116699	1228	10704	629798	51815	2110	657969

361893	123714	1132	12703	616600	81416	2130	622702
364091	71172	830	7028	476000	60657	1244	411826
409890	253959	2473	17077	743903	106988	5437	915261
601365	206907	2150	16394	1657247	134263	6189	1153537
383287	51778	1067	6668	628141	57724	2426	533269
372966	230971	1201	5992	575018	62500	7499	868804
452196	378774	1451	11074	729851	89011	5407	1073373
490876	440155	1918	13151	582423	107879	6815	1121489
255319	8364	348	3285	209597	30116	408	104610
248568	2984	312	2260	152984	24521	203	60232
460992	3734	357	4535	242709	21429	289	125081
340206	1751	100	2195	129787	9313	111	41080
393456	4291	368	4616	275577	25483	348	137493
146570	14245	483	4140	247125	39632	509	83660
211703	8720	361	4318	284124	34579	515	130226
296061	10634	1456	5098	331918	41956	543	167469
306474	14339	1343	6048	350877	50351	813	213503
324631	19486	1814	8310	500488	68250	1129	307131
438249	26366	1382	10376	504423	89299	1624	467929
386159	8851	1000	6889	482057	68679	806	254180
308735	11308	1121	6690	443366	66142	1074	278960
204856	7382	633	4087	118792	61033	306	135510
197609	5330	718	4546	106781	81861	245	119845
174063	21549	579	6902	49185	73630	533	221638
405916	15971	925	9861	425113	71911	1472	317479
238132	13037	372	5833	322972	69125	695	203153
340914	8365	318	4561	385169	69742	505	163241
254929	6295	670	6644	418159	103106	560	160117
360180	13545	1374	8452	539425	88062	873	174953
406843	7127	1003	11279	691298	104425	1019	239111
321511	20265	1197	4839	705203	107967	685	239468
333973	12681	1100	5924	586459	88873	763	213570
312209	161060	1310	12115	490923	107142	3118	641435
359544	10968	1050	7649	323157	72858	851	238336
377439	6176	1084	11124	512702	94225	736	227585
314234	10506	1297	7580	465880	100532	587	163722
313285	18059	1017	7347	315733	83569	829	241496
338405	15752	1199	8987	396484	102078	820	168022
368996	5798	2426	7259	642386	91231	571	119383
447658	4739	2093	7244	622529	81180	516	103254
313279	12653	619	7750	205330	63881	559	172172
330249	22567	1112	5373	244630	68177	751	212487
313447	9073	629	5318	313943	70638	520	135523
242200	15022	367	5015	247168	40293	1116	263586
279729	5609	788	6222	369030	64957	719	161032
349973	7254	232	5699	197245	46728	1183	223519
336278	6782	948	7295	382584	70742	1031	166844
377061	5900	1137	10514	512561	107704	1688	313689
299526	7753	848	6148	74351	57423	785	157499
445687	59109	1259	11787	524064	92832	3380	684257
252299	37775	728	8080	81279	123247	1241	344853
212660	9416	586	5645	50197	84225	579	119999
225361	12823	578	6656	48086	90531	582	170812

336965	62205		399	3518	184164	50384	975	345834
255966	23529		298	5594	235723	64191	621	211075
27	29		20	30	17	7	52	5
431735	20920		1094	9643	590324	88232	1402	409988
374519	98759		1355	12946	487066	82449	3832	804126
286614	438	NA		333	13577	143	800	800
157168	159140		1233	10182	2730	81213	1848	533052
563018	72421		688	10243	441074	87846	1544	485752
364911	49554		980	8159	367195	67145	1961	645541
370217	45895		1270	14793	429061	71913	1939	507543
334573	81679		1007	8290	316559	70868	1805	502348
301486	29001		1163	8461	432338	75158	1168	403202
290297	32006		972	8418	230385	58988	1331	380502
368195	162473		2239	9922	673585	85622	2798	938245
353202	53536		877	5003	277305	35242	1038	423088
324332	92367		1041	5551	291094	58208	1212	440684
352241	83199		649	8625	284915	58700	1631	447401
333875	211810		932	4447	212638	46349	1621	551386
344245	67118		22	238013	36662	84088	1354	445772
345786	109183		28	157668	29271	87182	1145	376641
300712	126		18	536	22106	51	3	113
325377	29		36	314	15451	28	22	59

Glucose-1-phosphate	Glucose-6-phosphate	Glutamate	Glutamine	Glycerate	Glycerol
16200	32	78694	728	857	34531
10378	19	50524	291	587	29831
23890	61	67255	2736	1525	28278
23972	21	56682	2173	1070	29842
43485	36	77631	5059	1259	31031
36022	47	55900	1228	2343	29340
37941	53	68684	1363	2911	27361
109536	18	90136	6873	5137	68207
54398	12	55154	5050	2491	45705
40734	30	48353	2141	1608	42483
49594	40	95819	2678	1999	40129
59880	49	129096	6131	3109	48725
103225	22	78225	9292	2579	41383
82350	31	52235	3795	2768	32670
1071	0	6	12	4	8
77072	85	98869	3533	2265	51375
37768	20	72870	2794	2299	48098
72067	98	47458	4971	1863	22416
119751	128	78430	7491	2095	19972
125772	140	51956	5166	2266	43862
66015	81	79927	4522	2041	50430
55262	79	88484	1468	2558	55181
125516	101	67121	8505	1990	45873
106739	108	65515	12215	1762	39875
120290	59	38679	7954	1677	160609
138004	70	126516	9672	2908	70898
92739	148	107004	7356	2441	55527
158872	142	50815	16025	3331	88423
207949	267	134720	25215	3606	355989
276242	170	38825	14172	4118	44317
144966	79	82498	6752	2547	50710
108411	113	71286	5094	1778	48576
202866	97	26248	6704	2043	67437
132520	31	35696	5261	1555	46654
137107	70	28868	7471	1410	40446
38697	47	65973	704	1665	32479
25312	2	57909	735	1758	36618
75262	18	74147	4194	1769	36722
79537	34	85121	4250	2034	36499
88780	83	134882	6871	2529	45359
36252	73	68889	2536	1775	39566
48154	33	64053	1784	2290	50918
183876	33	106069	8332	7151	92095
95898	60	107670	9816	4831	76284
62112	21	44774	4155	2490	57124
68878	35	75010	2939	2007	34163
110944	73	121294	5820	3074	49720
232088	124	164899	13559	5602	46613
123582	104	96057	10120	3559	26830
186535	131	122795	13949	2856	21749
85872	80	94135	5986	2601	28986

138456	123	129314	5490	3778	43070
270026	142	128010	12958	6511	26891
223244	161	44668	8158	3813	37235
176668	170	90011	11487	3996	62953
2022	31	1542	174	57	40746
206445	180	113361	4639	4706	59212
282484	104	69020	9910	10686	41527
241574	103	108095	6481	19546	74613
457061	411	216354	10954	10934	50701
176283	67	82650	6042	3744	53967
264846	93	170324	10595	6192	55509
226447	129	96764	12016	5927	43117
259362	156	79018	11567	5244	61700
343889	487	159180	7181	5912	58194
15540	32	52151	801	1428	27671
22106	38	57183	1314	1536	19005
54370	121	94757	3676	3256	85026
42465	98	113332	11058	2238	54844
52139	107	77602	7646	3111	57106
37212	32	59904	1235	762	19883
46563	44	98463	2874	963	21489
59292	63	102775	9443	1193	37084
92183	30	94022	9192	1957	53248
86773	180	163053	9544	3317	49020
41448	75	103899	4994	2823	61438
104061	127	97562	11197	3790	64725
95272	52	52816	12586	4038	19249
82890	68	54297	15818	4293	18575
71897	128	55595	9450	3250	33346
11655	5	21052	721	657	55508
30595	85	88053	5014	3994	47657
146134	62	114012	23452	13780	139981
12876	55	11381	1705	602	13581
23557	34	19397	2633	1863	15814
18556	77	21308	1698	435	24419
34197	40	77701	2529	1301	51700
100307	69	42122	12986	2402	25251
98231	75	46065	12625	1675	44633
123890	206	38830	17602	3692	40905
112756	286	167963	17217	6993	66913
86508	117	111073	13444	4846	49425
107394	132	26548	19739	4854	39251
93929	152	22464	18273	7004	33295
186545	162	59772	33170	4502	27060
86893	125	87490	8844	938	57440
91503	107	107306	18973	958	50340
96500	71	22190	2717	953	70660
73167	121	13644	8458	2325	55725
47530	240	29014	7073	1197	69824
26315	73	84415	1651	696	24449
48349	55	134729	1185	837	37909
81423	74	84511	6270	1301	35637
87823	104	116135	7414	1188	43301

98973	96	88642	12174	1405	261605
81770	29	178647	8817	2384	54112
45473	41	176198	9316	3600	84456
49559	371	65081	11030	3099	57109
87662	235	53673	11596	3904	36761
69297	283	74196	7931	2117	35704
56764	108	116588	7768	7289	53531
39251	46	70890	3813	2681	45799
32652	14	16640	3506	1595	15334
97148	93	80178	12439	5733	16869
136164	176	120169	15460	6248	23288
40904	126	63610	5104	1503	44647
77607	182	125720	8081	2553	73889
126407	75	50041	14583	4207	39125
157998	168	79384	20915	4458	36020
96498	23	60564	13690	3593	36104
152957	49	94299	9584	5293	57492
142986	113	106589	10730	4384	62442
198667	207	85345	21803	3340	35094
123940	223	58644	22285	3679	24429
140599	213	94167	14266	2876	29060
89407	57	111081	7865	2371	50177
216719	62	87897	8117	1515	61974
218972	535	100339	15107	5029	48476
178143	79	76728	11591	3133	57012
176099	99	59332	13641	3410	69866
18034	66	78968	1099	1224	24371
16219	28	45936	617	768	30487
23324	168	67391	1827	1052	32735
25176	34	36107	1712	902	33071
31435	128	63634	2043	1489	210297
95658	96	99139	1938	1171	34746
43819	146	106672	1901	1577	24423
8963	76	23172	824	118	40733
60673	77	70908	2232	2151	42360
85214	155	96246	6069	2269	23502
26974	57	61832	1732	753	40017
21600	64	54488	1151	602	45937
35686	27	25376	3007	455	45255
74796	55	76357	7863	662	23944
59350	44	53712	3877	542	31015
87895	40	99732	3137	933	36121
64509	75	105911	3199	1559	89623
44059	76	69958	4632	992	26056
66533	111	63363	8070	1255	25362
101732	157	81164	6623	1345	31354
52110	38	56956	2408	981	46361
30219	111	62442	2557	968	36454
116241	90	52500	6337	2046	26929
129287	112	82889	16046	1950	57636
105428	64	52543	14522	3394	92228
118338	139	94778	6288	1490	40812
69300	80	97361	6155	787	67789

201005	126	50711	11597	1078	52448
124834	100	45001	9225	1158	33787
36245	62	24293	3206	525	61396
130702	193	123320	10347	1242	58351
86458	89	102787	10192	1096	43936
121393	54	19242	6244	1766	49094
134571	82	48583	7902	1307	49446
92111	47	28707	5356	1687	54533
24947	19	64826	585	1061	146396
55686	67	112101	1398	948	21857
89825	99	97180	3037	1013	70867
79632	50	84266	3620	1630	33709
97416	57	66548	3002	709	45388
22899	168	49657	547	505	58502
59403	57	79731	1010	921	44237
45741	11	44819	1575	739	69244
47602	50	43976	529	878	48301
67326	72	68255	2685	719	34260
64594	74	138069	3988	1769	50254
46221	38	89279	2418	1305	83953
53875	62	69075	5088	1040	45261
331	7	2	7	5	44
85844	21	60740	3454	892	43234
89205	113	87288	3223	1007	52644
73354	41	103791	4227	1012	35695
141068	132	78891	8438	2942	86622
137271	63	66276	9161	2369	119175
105144	90	70002	8130	1521	76802
129441	105	119808	5509	1493	58118
96612	113	126983	4494	1633	40274
230730	143	92443	12049	2131	44802
219074	150	89922	9740	2077	46911
118878	67	76101	5052	1244	18335
234081	180	144498	7373	1197	56416
182683	148	154695	7330	2862	57484
174174	73	28409	7745	1369	49097
244347	28	58056	10298	2743	32799
125606	42	49571	12954	1526	31020
112477	1502	161681	16535	35923	10319
46025	403	62848	3321	48340	11606
52075	3012	121302	5539	22676	14294
40221	2408	89599	5078	35367	24220
41224	1741	74073	3157	31753	16180
57773	344	72811	3506	32139	7519
26941	462	49553	2505	70069	2862
104179	4063	330843	17040	74388	13019
108070	4129	300022	22225	97830	10653
90294	2438	234273	4285	87145	17066
58	4	4	26	9	33
142971	1826	203373	7051	86961	12584
124209	4243	213904	11226	110439	17023
88213	6039	292677	15962	346763	28776
95125	5041	241517	12829	148383	14696

70569	3212	139660	8016	88988	16778
248	NA	5	9	18	18
43711	3041	25193	14511	45820	15215
20340	1873	37401	7607	67754	22899
10317	417	5851	1385	5964	20480
93490	2429	106449	11077	46574	12217
134610	2217	178344	18971	122793	14786
57295	2100	50712	13438	43023	18107
37199	1288	28857	5829	16022	21943
42156	1290	25028	3713	12636	23813
96367	2269	107093	14311	31524	8763
140730	2784	145625	8921	32873	9912
68126	3302	39568	47529	65023	73903
57623	4763	58310	48327	24543	28945
45504	1670	19713	16720	87701	11631
81278	2448	72856	4680	43896	9833
27466	900	21482	1712	13457	7007
50016	2423	29671	5112	13756	56976
30882	1319	29974	3101	113242	29456
50570	2822	32138	21526	53389	29035
2713	24	3394	176	2038	198
739	10	4657	157	6750	138
32420	2536	195593	7999	170688	19293
116169	4218	289350	15585	228192	18694
25084	1818	80870	8150	91525	20361
98080	1824	155430	7156	208980	30237
85796	1829	121196	6651	222448	15120
78078	2108	177220	12570	269237	12503
103693	2907	267401	18080	137629	8904
95041	2413	256697	20572	167268	14483
146733	1798	204627	11508	371592	22779
178429	2002	60986	9899	354579	NA
22645	1611	70215	27315	332600	21390
43819	1733	39988	21610	335620	29530
41265	1881	67677	39570	247837	15249
83501	2287	156079	31263	454794	16138
120056	3130	159987	28167	215182	14138
34847	3839	65125	15499	111375	48137
37954	1724	61913	23548	286595	32068
29151	2190	52054	8644	283791	25463
105126	2873	3398	436	NA	NA
91976	1854	117825	30780	630190	9464
44036	2673	29009	38719	432109	23752
58555	1611	47472	22088	479653	25531
52584	3610	33283	9266	645288	16474
93177	4533	75822	7042	357172	16873
166041	6072	254606	44238	412291	17451
68987	5428	70635	29389	358149	37170
51453	3492	66594	31140	563354	31744
60039	4051	56252	14738	599575	35970
97826	1420	155864	7570	14292	6587
57936	1296	151113	5069	13473	6872
127251	3417	333178	11242	10507	17249

47760	3005	159558	6602	26602	20583
54294	1868	142358	4823	9331	20233
207468	3931	300854	18890	5333	17165
591	8	10	41	8	103
50687	2878	123496	5984	15068	17156
69256	3100	161791	7273	70714	12177
76822	3188	184259	14057	100588	14022
149801	4930	249360	15755	23118	11689
177467	6388	243367	17051	78963	13724
56965	5946	4769	30505	39783	NA
72654	11308	4220	45465	19459	NA
33350	2579	30041	7301	15160	19399
53484	2833	46147	18149	78686	34065
54223	6781	14677	25831	73595	48899
30836	1983	22934	5396	30676	21743
74048	1976	104673	10581	80661	10250
68134	1627	81281	6612	42011	10119
31199	1632	18745	6188	42705	18549
31123	1234	17335	6022	36169	25415
17062	682	11138	2525	14335	12326
112549	4996	155609	25972	29720	11212
48228	2104	77472	9055	32451	10363
36372	2558	30448	22013	29742	34810
33567	2650	31907	20143	28531	35810
40688	6473	33996	55999	41809	268161
51526	2238	89253	8447	68621	16336
113609	3719	101974	24867	16225	11005
26838	1860	7703	7360	10826	61452
12910	752	6901	2845	34728	25201
18978	1045	7888	8256	30321	40319
54139	1046	58516	1502	20943	20378
74662	878	105350	3800	20480	11713
61375	3679	129378	4047	19416	26884
64758	2965	180007	8359	49408	12452
47309	1965	113881	5749	49222	14561
66475	1392	85648	3713	20031	12754
120268	2404	174197	8956	35756	25331
6964	616	9241	1137	5095	11205
17542	1092	15600	4104	25332	8439
19537	1302	21055	5372	60305	10185
100493	3398	191544	17601	97043	36868
3269	9	5	70	34	2980
48538	3051	161657	12736	348651	23203
30473	4466	73228	13513	162211	30677
9203	1344	17844	4831	77642	14215
10421	773	16771	8798	113956	5324
62887	1808	91680	16518	335744	11204
109376	2444	119604	17126	181112	12481
25799	1219	22299	6926	61392	19359
22062	1155	27763	10037	119567	18194
29420	1116	21699	10853	87738	15182
101355	4098	120967	14786	323668	12931
64016	2489	86693	18543	91482	14417

49480	3961	55767	35295	146361	100746
31471	2648	40759	20984	99660	46525
60872	5843	79472	31674	329562	84799
149510	4286	245261	70460	61935	22941
93453	2215	54760	7609	127424	16553
55994	3870	29532	32190	46638	27612
49470	5181	44245	31728	227374	45708
60855	6378	46799	40457	258655	56353
53505	492	81083	4491	19157	5611
44800	665	52797	2353	2459	5573
21358	1287	55945	1074	10853	14137
12708	620	24106	721	12596	8665
31237	927	59202	1610	10298	12433
79311	734	110306	4391	22120	5002
50523	889	92864	4187	32378	5214
56186	2748	113271	3961	26361	9461
57590	2427	155165	7620	55608	8959
72780	3541	222418	11425	77286	9940
131998	2498	215660	14692	102293	25333
124953	2038	142782	11144	69365	25459
71016	2859	132782	9097	37285	9085
18361	3543	2284	11038	26089	6017
27210	3943	851	8884	42387	NA
15788	2814	455	9061	104421	NA
135927	1515	110538	10765	109306	19836
32128	1326	21092	18160	171395	11308
30347	1147	47190	15185	52069	19717
54420	2201	26482	34666	40451	18330
115374	2739	127048	20799	41237	10633
137686	2432	154504	24983	89388	11396
83764	2894	77332	34544	29248	17754
50121	2403	43421	26348	51366	33120
57829	3160	47036	77366	403801	26876
82161	1938	67958	23027	90183	12584
101750	2156	139602	83003	61836	13840
56403	2504	34278	66906	50610	24524
38218	1955	23275	89840	134157	14175
44609	2502	25583	30848	28233	26627
109951	4988	107526	34784	19592	12104
85788	4343	78617	18534	9841	10165
34886	1979	16763	16592	59488	20171
33800	2429	22717	20848	108583	31718
41036	1845	17542	10005	35019	22959
50603	743	116785	5842	138143	9983
71772	1145	139772	5573	111773	15726
41284	943	85730	4731	104631	11575
70153	1932	166431	8964	73936	22517
92641	3342	188386	10341	125485	22841
73602	1776	24849	8214	69621	45482
148338	2485	190234	10095	373060	21250
33591	4403	1062	28136	263117	NA
29302	3183	797	6258	98719	NA
33277	2488	245	6877	158895	NA

21954	1573	30068	17578	214081	18046
30726	1226	15527	11824	127680	15582
394	11	3	16	10	1614
119630	2322	99049	20588	182934	13196
125484	2698	140150	32195	487051	11030
2471	177	14	28	91	11761
71571	2940	270	71	263387	NA
34002	2417	41757	29751	226226	39435
82288	2180	83220	23560	229344	10245
82534	2583	103068	16793	339650	7523
48182	1961	21120	36118	275436	15479
53277	2190	22926	33439	152021	18211
36653	1905	17568	23408	287490	17590
138504	4546	136184	58245	355710	14035
69816	1855	47903	16790	104460	18420
35734	2446	19076	19276	216044	30488
42799	2037	25213	53483	342447	26914
32484	1989	36270	38208	266730	27352
84911	245	74493	19820	2722	14012
49812	229	10450	19082	2160	32858
3769	15	31	84	5	1147
2123	56	16	NA	10	1861

Glycerol-3-phosphate	Glycine	Glycolate	Guanidine	Histidine	Isocitrate	Isoleucine	Leucine
NA	63146	873	4174	19	2763	4153	4763
NA	10663	952	6853	44	2077	1955	3154
NA	28474	624	2329	30	4975	4784	7021
NA	28210	488	2247	60	3380	5368	6024
NA	49091	776	2249	166	5211	9749	10760
NA	21693	1108	3124	76	4695	4481	5045
NA	37325	1039	5969	84	5028	4858	5502
NA	109948	1912	2145	200	3740	18023	18341
NA	88648	898	1512	129	2026	7496	8590
NA	28671	854	5585	53	2478	5260	8331
NA	23784	807	3210	145	9752	3530	5141
NA	64621	1069	3358	201	12241	4961	4927
NA	66411	907	2883	154	6869	5068	5163
NA	47303	1018	5419	76	4331	5004	4882
NA	69	431	4	9	0	99	72
NA	22367	766	2910	117	8236	5952	6515
NA	40303	759	3390	80	4597	5114	5075
NA	40403	1012	2779	114	3037	7894	8159
NA	21033	1348	4782	95	5997	7517	7540
NA	23690	1445	4813	111	2526	15489	17015
NA	74349	859	11691	197	8966	5865	6077
NA	71576	933	35837	40	6154	5041	5114
NA	36105	1637	7061	102	4585	6017	5899
NA	73510	1966	7056	87	6085	6636	6596
NA	58979	2582	4440	16	2576	5999	5552
NA	150825	2145	7356	224	14695	7949	8474
NA	95232	2351	6173	174	8513	6699	6610
NA	318998	3624	23965	117	6770	10961	10926
NA	279881	2223	11906	295	24833	14420	15282
NA	121961	2298	9865	503	4515	14334	13292
NA	25543	2407	10582	77	5552	13302	16015
NA	19205	2246	11796	149	5106	9642	10284
NA	31666	2544	10966	69	2618	17524	18859
NA	29703	2047	7791	75	3158	13246	13158
NA	50527	2239	24299	119	4145	14354	15461
NA	15677	876	9153	74	3449	5212	6195
NA	16328	1065	4481	14	3071	3894	4885
NA	29399	776	1881	45	3746	6213	7630
NA	20272	858	1152	121	3495	6575	7332
NA	25595	894	2261	63	6387	7879	8874
NA	36819	836	3537	169	5571	5596	6247
NA	29978	1674	5114	246	5160	7384	10287
NA	90224	1762	670	73	5046	22672	26293
NA	44928	1187	2994	100	4343	13523	20086
NA	33471	740	5352	48	1585	8279	11689
NA	16237	751	3450	181	7199	8247	9566
NA	42077	1175	2784	129	11769	11544	12252
NA	37765	1601	2151	173	22229	27591	31080
NA	26436	1171	7414	76	7008	13788	15745
NA	24969	1469	4617	142	10251	12133	13707
NA	28378	1479	13659	107	14066	9031	10847

NA	31083	1061	31304	286	21755	14848	18560
NA	50848	2208	4794	178	27708	17522	17887
NA	17942	1998	9307	24	6106	16127	19149
NA	30620	2140	4981	211	21777	20518	20890
NA	47118	1045	2228	17	105	117	220
NA	66416	2006	8208	347	12400	16832	18827
NA	27375	2136	13428	257	7503	23949	25167
NA	29684	1828	4712	264	10267	43346	53387
NA	35388	1964	7914	841	22780	54828	62556
NA	29376	1766	17297	295	9987	21588	28461
NA	89070	2199	21432	353	23703	24410	30358
NA	52026	1745	16260	201	19955	31042	29341
NA	34695	2145	14825	236	17731	21976	24759
NA	27539	1822	22230	521	20656	33903	37923
NA	20296	1043	9298	54	3866	2909	3394
NA	30495	764	2375	48	5303	3319	3790
NA	58290	1187	60329	164	8316	6039	6109
NA	75473	855	4195	146	13742	8704	9945
NA	56550	1102	6449	212	6017	10850	10978
NA	48128	1276	10107	125	4488	4436	5019
NA	44233	1660	6192	58	7817	5131	4839
NA	32172	443	903	44	5809	4381	5713
NA	63038	769	4763	48	6575	11755	11937
NA	61928	689	8211	52	11901	10948	11046
NA	156136	852	1684	141	9559	6434	6264
NA	126621	1147	6523	121	10669	7096	7897
NA	119232	1500	1516	47	5041	9392	9339
NA	83788	1728	4491	132	7136	6830	7175
NA	64913	1674	2213	136	6315	6105	6072
NA	27559	940	6176	5	1078	1912	2284
NA	27474	963	2096	28	6070	6841	7482
NA	134456	2451	5318	70	7551	26138	28090
NA	46489	328	1156	32	420	3590	4986
NA	24174	375	2392	48	870	3820	4493
NA	14328	677	1714	25	1256	1607	1797
NA	35644	1103	26577	71	4165	6937	8171
NA	79453	1122	3093	141	3686	11130	11304
NA	134158	1233	6106	63	3653	8133	8321
NA	81351	1286	6988	184	3205	11950	11508
NA	200229	3390	20041	244	18930	13573	11725
NA	113226	2759	5995	134	11214	8900	9093
NA	149958	1718	11708	147	4432	9316	10120
NA	83201	1776	8060	151	3827	12460	12847
NA	248257	1962	17949	256	11822	7744	8354
NA	21417	1763	5093	176	4927	14831	18204
NA	24439	1515	8305	275	10351	20464	26480
NA	35709	1290	343853	87	1259	16022	20038
NA	32619	1076	12353	118	1137	15055	16528
NA	31344	878	13749	4	1959	10462	11619
NA	519023	743	2816	99	3696	2657	3141
NA	12370	1423	8932	87	4419	3889	4712
NA	12878	458	1159	86	3209	6050	6601
NA	23758	844	2868	65	3906	13298	14060

NA	21036	846	3427	130	5751	11720	13320
NA	44569	742	4447	138	10358	4907	4852
NA	63426	1010	9110	105	8440	5195	5479
NA	225361	1411	8741	58	5646	8346	9124
NA	112275	1321	14108	124	4556	11575	11594
NA	58826	1031	17129	97	6099	3933	7483
NA	50152	1370	7760	121	12136	12196	11919
NA	21645	1090	3481	74	6360	6724	8359
NA	24751	591	1079	34	908	4302	3706
NA	48684	491	1370	70	7919	8609	8351
NA	43486	899	4401	84	12002	12438	12319
NA	16199	558	2641	55	7725	8565	10045
NA	33538	940	8364	265	16479	10497	11853
NA	41952	1139	5384	179	4455	15224	16303
NA	52417	1408	6118	228	9839	21503	23456
NA	33597	1074	3987	138	7504	15131	16199
NA	75639	2155	15683	241	15787	15957	19611
NA	56923	2179	9369	241	13846	19403	22433
NA	57868	851	9944	275	16163	14181	18074
NA	65329	1294	3846	137	12022	10890	11858
NA	47221	1536	4835	329	20467	18405	20509
NA	40672	1494	10134	234	11249	41123	46932
NA	27341	1925	7471	390	13651	47118	55819
NA	78161	1691	20305	623	17709	61242	51699
NA	40310	1472	6763	353	12508	44507	50252
NA	46087	1642	14553	321	7047	37615	44383
NA	27013	1034	3099	145	3993	3195	3441
NA	15781	985	2908	65	2405	3118	3480
NA	15628	563	2247	85	2438	3425	3796
NA	22098	933	1989	129	2155	4965	5396
NA	48646	1081	8899	78	3932	5606	5614
NA	29857	1063	7559	259	12228	5281	5972
NA	21519	660	3990	66	5458	3265	3840
NA	4328	380	12804	11	1155	898	2525
NA	26626	636	22146	99	2982	6579	7251
NA	22699	898	1625	58	4041	5507	5639
NA	56644	666	3873	81	2984	3242	3632
NA	60894	665	3746	121	2568	2510	3618
NA	61263	483	1930	105	882	2746	2807
NA	57513	536	2118	51	2657	3875	4168
NA	68624	352	1818	31	2334	2890	4648
NA	20628	823	2136	106	5307	8139	8472
NA	40089	1673	7558	113	5142	9287	10068
NA	202	845	9791	109	2620	7814	10033
NA	23740	457	1161	87	3108	10897	12712
NA	15550	1127	6137	103	3803	9748	10723
NA	32719	656	7578	112	2751	6190	6557
NA	24380	857	5864	64	3095	5275	5754
NA	58916	1374	3575	96	2757	10044	10213
NA	32878	1511	8351	203	7825	20451	21214
NA	97926	1929	8652	112	2340	15283	16556
NA	77331	1867	13574	144	7219	11892	13789
NA	70397	1429	5009	114	6832	10902	13965

NA	80843	1949	24377	348	5259	21075	23111
NA	62435	1196	4151	140	2261	17976	19692
NA	22953	1047	6148	14	1265	5801	6296
NA	27941	1996	25540	261	8880	15229	17988
NA	28106	1548	21287	242	7470	13945	16574
NA	52801	1547	26335	202	1296	17651	18291
NA	37720	1779	14116	315	3399	26323	29713
NA	36105	1891	23613	101	1422	13079	12547
NA	10351	891	2659	42	1000	4083	4487
NA	9414	835	1739	127	3283	5643	6403
NA	8638	881	4567	118	2120	7920	12277
NA	10346	498	3454	164	1835	8316	12989
NA	11062	405	4029	113	1389	7996	9003
NA	11532	821	9437	61	1114	7138	9210
NA	19824	852	4065	100	3436	9515	11784
NA	6544	510	4540	41	1000	4622	5324
NA	9231	736	96711	39	923	6118	9779
NA	11772	703	5225	94	1524	9826	12141
NA	35074	1462	3583	90	6472	14397	16266
NA	18595	1756	8485	93	3670	11276	13148
NA	18963	325	2340	76	2147	9781	11821
NA	2	0	3	1	19	13	77
NA	17097	593	375	86	2309	8494	11246
NA	16396	1013	13282	216	6237	12287	14384
NA	12543	913	4967	160	5467	9926	12095
NA	30065	1987	28923	186	5986	27780	32981
NA	25419	1495	19621	67	5422	21502	24292
NA	20799	1475	12742	65	5120	20929	22519
NA	19650	1766	16372	149	9946	12538	14682
NA	73796	1679	12889	159	8326	16427	21335
NA	29528	1837	14035	158	7203	26362	29318
NA	33977	1768	9678	92	6534	25936	28972
NA	12891	1359	4099	16	3073	7586	9531
NA	38764	2558	82173	358	19325	28806	37220
NA	26817	2185	64554	218	16019	32349	41455
NA	25812	2568	10230	168	2047	33036	35344
NA	18963	1902	11821	179	5569	26805	31144
NA	34096	1990	14520	147	4787	30689	36776
1202	25808	1554	NA	87	NA	18484	31810
471	20765	1057	NA	40	NA	10436	17935
327	31202	2186	NA	4	NA	10561	17078
319	27458	1896	NA	9	NA	14823	27919
319	23961	1825	NA	31	NA	11630	19817
520	16598	663	NA	20	NA	8826	13887
297	11729	385	NA	20	NA	6243	9962
806	44755	1540	NA	423	NA	23245	36654
770	50495	1406	NA	189	NA	24107	36305
579	42665	1354	NA	193	NA	20179	32080
21	15	524	NA	74	NA	14	3
668	59988	1474	NA	66	NA	17833	26367
827	38490	1088	NA	45	NA	22740	32614
981	61454	1593	NA	102	NA	39931	56747
987	34000	995	NA	151	NA	28797	41857

1165	39914	1075	NA	98	NA	25327	37780
6	18	NA	NA	3	NA	210	84
655	24821	1366	NA	61	NA	15190	24370
386	20206	1136	NA	81	NA	12185	19522
178	7746	581	NA	6	NA	3571	5821
796	59249	1192	NA	145	NA	21767	30185
721	52656	1146	NA	117	NA	23847	32452
524	38799	1455	NA	83	NA	15920	22565
361	18461	971	NA	34	NA	11243	16380
205	18136	1093	NA	54	NA	8949	13577
776	32636	893	NA	73	NA	12690	20311
899	51780	1245	NA	86	NA	18255	27206
643	49299	1926	NA	79	NA	17087	26168
867	67941	1646	NA	61	NA	11974	20631
534	27340	1258	NA	63	NA	15896	23678
994	31305	873	NA	67	NA	26626	41522
315	11174	735	NA	47	NA	9893	15157
730	17581	1363	NA	108	NA	21030	36882
461	24425	1372	NA	56	NA	22894	37541
677	44866	1229	NA	89	NA	18504	31561
58	554	46	NA	5	NA	375	555
41	479	25	NA	44	NA	207	297
892	37516	1186	NA	236	NA	30268	51271
948	45053	1291	NA	340	NA	36447	59808
598	33656	127	NA	65	NA	18439	33474
972	52195	1500	NA	47	NA	39488	58670
1067	37044	1075	NA	67	NA	34422	49738
957	38864	982	NA	24	NA	35162	52655
1090	38585	1026	NA	144	NA	38803	56072
1230	47300	1045	NA	102	NA	38243	58647
1384	85324	1906	NA	190	NA	40494	59302
1793	NA	48	NA	182	NA	NA	NA
490	37125	1182	NA	70	NA	27482	43855
538	27339	1205	NA	199	NA	33208	60916
589	62915	1291	NA	217	NA	27776	44603
1381	68827	944	NA	151	NA	78587	108414
1855	71333	1128	NA	132	NA	113352	162541
721	41163	1394	NA	22	NA	28654	40909
661	42695	1544	NA	82	NA	40462	56779
550	32305	1273	NA	49	NA	37526	58079
2022	5082	13	NA	168	NA	3250	7358
1090	68816	1020	NA	121	NA	62488	82945
991	70504	1483	NA	89	NA	59054	89041
564	40728	1353	NA	143	NA	42866	64513
707	36225	1332	NA	229	NA	55147	93547
1696	67018	1667	NA	127	NA	38388	63667
4640	63304	1691	NA	494	NA	87864	125797
1844	45950	1792	NA	122	NA	82020	119960
1566	57393	1470	NA	188	NA	75518	97486
1136	40274	1337	NA	156	NA	48140	75099
1058	21316	933	NA	120	NA	11948	20626
993	16137	846	NA	66	NA	9982	17573
853	30245	2006	NA	269	NA	19662	31653

745	30916	1359	NA	98	NA	15690	25268
719	33227	1594	NA	114	NA	15787	25797
1566	35806	1829	NA	258	NA	35452	61497
53	23	NA	NA	15	NA	427	189
620	28040	837	NA	65	NA	19377	32600
972	31129	946	NA	190	NA	30159	51890
882	38931	904	NA	273	NA	36500	57952
1106	42159	1268	NA	149	NA	18686	23495
1537	43998	1689	NA	145	NA	44483	64040
4117	55750	NA	NA	113	NA	15591	21431
1255	29260	11	NA	183	NA	24927	NA
420	58821	1361	NA	52	NA	14024	19733
813	47593	2439	NA	116	NA	32213	43939
1399	36899	NA	NA	92	NA	36193	53889
455	21253	1283	NA	123	NA	12546	18705
882	31028	1041	NA	67	NA	26917	38019
756	23124	1005	NA	80	NA	19686	28843
553	15665	1046	NA	73	NA	19096	26940
503	26153	1151	NA	93	NA	15293	21317
214	13415	727	NA	38	NA	12546	16934
1307	48829	1095	NA	79	NA	20770	33403
855	34085	680	NA	59	NA	13150	20997
575	70754	1066	NA	75	NA	13646	21136
446	74909	1283	NA	84	NA	13529	20466
679	178498	NA	NA	29	NA	38333	56570
946	39889	1175	NA	130	NA	36763	61077
1418	35407	1345	NA	231	NA	50747	78921
379	13849	939	NA	52	NA	17382	32892
252	15740	834	NA	36	NA	16892	28472
468	20437	911	NA	44	NA	25663	41923
527	23314	1617	NA	57	NA	16827	30135
814	24046	1362	NA	83	NA	18664	31124
804	19859	850	NA	159	NA	23718	40283
935	40743	948	NA	187	NA	31836	54779
760	29109	931	NA	16	NA	29234	51598
587	20680	898	NA	50	NA	10429	14817
839	28735	1330	NA	148	NA	17284	25036
94	79795	739	NA	18	NA	12091	20760
278	18511	1078	NA	29	NA	7187	10970
308	30053	1099	NA	87	NA	8523	13916
1321	48370	1650	NA	82	NA	42367	61893
3	4898	383	NA	17	NA	419	576
1384	49772	1274	NA	41	NA	62582	88029
1394	54149	1603	NA	87	NA	50804	73678
432	19631	741	NA	11	NA	14525	21149
418	12743	353	NA	65	NA	14720	20493
1336	42514	806	NA	33	NA	73760	100088
1527	54155	1207	NA	89	NA	58738	83649
793	18583	1409	NA	50	NA	30184	47680
500	24785	1217	NA	12	NA	33154	48654
646	20552	1277	NA	49	NA	35185	50429
1628	34765	1034	NA	294	NA	31551	43802
1691	37330	941	NA	90	NA	59169	83527

1284	37226	1635	NA	127	NA	38304	56610
1224	36391	1553	NA	80	NA	40835	58985
2200	169059	2359	NA	249	NA	56866	83718
7510	60453	2224	NA	601	NA	138037	214129
2078	31407	1255	NA	146	NA	68553	115179
2983	28002	1209	NA	251	NA	43024	63810
2388	38896	1474	NA	91	NA	87557	124853
2780	39695	2053	NA	124	NA	101719	147924
412	15809	794	NA	126	NA	7913	10331
287	8986	660	NA	67	NA	4149	6635
210	15285	1496	NA	18	NA	7589	11185
116	68230	1433	NA	14	NA	5195	6986
252	16703	1391	NA	6	NA	8843	13114
402	18021	405	NA	114	NA	7132	10622
326	20046	841	NA	21	NA	8094	12751
516	23188	890	NA	59	NA	10072	15665
610	29515	758	NA	180	NA	13844	21345
693	43423	945	NA	252	NA	17955	26196
979	43995	1227	NA	130	NA	27771	39894
709	27898	1267	NA	152	NA	16615	24518
595	29667	885	NA	92	NA	13987	22041
10547	16764	5	NA	28	NA	6297	9410
18365	11297	13	NA	20	NA	10190	14705
18995	14719	NA	NA	67	NA	9789	14354
872	34885	1366	NA	67	NA	27797	44008
484	24102	1123	NA	94	NA	22882	36195
486	24090	1114	NA	146	NA	19492	30739
706	21157	1412	NA	96	NA	30430	46766
1103	49271	1102	NA	213	NA	30641	44000
1285	38721	1269	NA	252	NA	38244	57095
645	84424	1614	NA	154	NA	23558	35765
740	39029	1338	NA	115	NA	24223	38213
1128	70260	1881	NA	164	NA	68800	106483
957	40835	957	NA	162	NA	26624	40850
1410	69809	1368	NA	384	NA	41394	62215
1039	38916	1510	NA	147	NA	27772	41628
753	44716	1102	NA	209	NA	37583	55133
870	31146	1227	NA	106	NA	28477	42794
1595	66237	1047	NA	397	NA	51383	80381
1281	39648	803	NA	160	NA	36478	60113
606	25736	992	NA	39	NA	19533	31690
532	32839	1170	NA	62	NA	24247	40963
569	19687	989	NA	41	NA	20084	33441
445	26037	1043	NA	36	NA	18577	29380
706	26722	1234	NA	82	NA	23339	36215
526	28167	753	NA	75	NA	14807	25485
644	30394	933	NA	124	NA	29882	48714
1058	36277	1295	NA	361	NA	35940	58912
867	15752	113	NA	43	NA	34569	52111
1097	43327	1041	NA	187	NA	56248	95027
2988	28840	NA	NA	76	NA	33754	49291
17928	14131	13	NA	38	NA	28298	40648
17070	20061	NA	NA	32	NA	30333	45223

418	35296	1076	NA	45	NA	23460	36826
547	31700	1079	NA	79	NA	26973	42947
22	18	20	NA	37	NA	NA	441
1310	40644	924	NA	162	NA	56461	92369
1303	70565	1521	NA	253	NA	61759	94555
12	5056	668	NA	22	NA	306	519
929	3868	6	NA	19	NA	2593	NA
1034	40275	1581	NA	112	NA	49730	76780
1025	45263	1004	NA	154	NA	40775	61682
1223	40261	724	NA	152	NA	49136	72543
908	46419	1356	NA	123	NA	43399	64575
881	36794	1464	NA	126	NA	34477	53360
716	33266	933	NA	93	NA	36712	54715
2009	199301	1184	NA	511	NA	57080	85612
1157	50593	829	NA	77	NA	46200	67900
1052	56690	954	NA	123	NA	45565	70033
909	42400	857	NA	102	NA	56026	83954
805	52193	831	NA	123	NA	49611	75307
397	463004	1864	NA	24	NA	10534	11007
135	446606	1649	NA	65	NA	8929	9761
51	2816	340	NA	12	NA	292	305
18	1685	290	NA	3	NA	51	198

Lysine	Malate	Maltose	Methionine	myo-inositol	Nicotinate	Octadecanoate	Ornithine	Palmitate
505	37976	227	1259	24149	648	12404	406	67267
428	15421	196	806	14732	414	14170	202	45549
968	24194	303	2406	42075	514	5169	320	30611
820	26232	309	1975	31457	516	4241	258	30463
1457	21711	573	2443	60629	545	4086	486	32837
787	15350	710	1992	49774	847	4409	249	21843
898	21114	834	1574	61205	754	3961	334	21314
2348	50649	1804	8269	126541	2007	7647	565	79459
909	26200	309	3065	53585	821	2172	431	29446
758	15217	643	1726	52650	470	1544	259	14855
1140	26437	740	1585	79060	603	6710	385	48850
1942	28094	1129	2243	109826	1371	4741	639	48516
1216	28262	635	3977	104118	1037	2802	546	36104
1259	25985	890	3072	101456	811	4472	326	44088
127	24	11	14	72046	4	12	1	424
1773	28443	878	4738	74721	1761	3050	528	28760
1010	29436	563	3214	73849	1071	2504	409	23077
792	25874	764	2429	54589	832	3646	279	32977
1656	36663	1027	2946	67790	1282	4242	408	39398
1880	31957	1417	3831	79000	1672	4301	358	40079
1291	24022	799	3819	71936	949	4360	537	37646
1058	29145	662	3029	79044	927	4678	305	36620
1778	35321	977	3119	65883	908	5851	647	62791
2004	40635	1121	3199	74801	1001	6444	766	65634
1620	29141	1003	5439	52169	1483	3379	396	32959
2455	29116	1224	4535	78019	1946	4377	1198	43593
1593	31668	976	4897	83334	1588	3786	795	37687
2305	34563	1161	8882	102831	1782	26664	1314	185915
4255	26736	1408	7034	118549	1659	26942	4250	291213
2348	31540	1858	8921	130471	4076	17913	554	227761
2527	36339	1266	5958	97053	2359	4089	531	29521
1832	32921	1060	5494	108957	2093	3848	466	38994
2721	21222	1250	5637	103354	2111	12996	287	146520
1733	22926	905	4586	109576	1250	19067	232	198471
2500	9905	1160	5355	99902	1441	15527	868	185382
994	21581	1005	1662	57711	773	3956	249	21251
671	18697	793	963	43073	749	4727	258	19496
1762	30236	839	2140	69642	606	2557	517	25840
1809	43346	1113	2609	83784	719	3979	444	38241
2115	83023	1015	4094	91881	1512	5368	717	52545
1281	18767	1152	1841	93857	945	7163	278	40030
1538	19394	1627	1730	105328	762	8601	312	61432
3091	51967	1614	5155	144082	2533	8275	529	73326
2036	61908	1117	4679	106607	1952	6634	622	65870
962	28967	337	2500	49593	1244	1311	229	25363
1844	26797	887	3223	132344	1108	5889	322	42253
2123	39273	1217	3578	166997	1371	2680	680	26023
3676	62665	1737	3978	189690	1628	5809	894	51700
1896	53152	1034	2812	120293	1100	5852	701	53793
3099	50581	1509	3476	154330	1379	4975	766	43403
1648	43358	1013	2637	189625	1150	3217	414	28491

3855	50791	1311	2942	184207	1213	5256	644	41222
4665	74265	1686	4220	225121	1443	4342	751	45940
2236	41496	2016	2815	156682	1527	3759	453	40530
6572	51823	2371	3604	223028	1614	3993	527	46697
27	4615	41	1142	716	261	1933	29	16682
4058	67964	1742	6959	262998	2202	3917	365	38770
4620	45609	1562	8951	178001	4003	21742	587	255769
10116	59560	1707	8276	229895	2805	24598	521	274358
11770	127217	2190	13309	285721	4084	22465	875	248781
4123	42427	1550	5836	227474	1949	3781	541	38958
5812	66060	2367	6405	302866	2516	4683	823	42100
3534	94869	1484	6041	248089	2233	19062	442	195873
3325	63745	1679	6134	225846	2265	14621	531	143833
5410	123572	2609	9128	305805	3341	13770	420	153301
610	15610	541	1001	27202	526	6686	253	25667
615	16036	589	1115	28825	478	5741	237	29260
1954	40653	1089	3091	66719	784	6288	578	55068
1824	29833	970	3159	52197	663	4865	623	46091
1946	29776	1348	3368	62549	709	5272	488	50094
1680	15467	874	1117	57255	463	4840	145	37872
1374	17186	1020	1293	47989	555	4592	509	33849
1570	22421	973	2247	55643	585	4455	495	40026
2117	29533	1043	2796	76012	736	4697	433	44843
2557	55497	1463	3255	73051	571	4714	745	41655
1082	44386	1003	6391	64329	1783	2354	376	18593
2819	45648	1779	6382	78557	1835	2400	646	19788
1468	30574	1685	3535	73098	1022	3450	336	30280
2293	39546	1404	3265	64680	810	4269	613	43865
1731	40731	1461	3121	67816	863	3642	332	37793
348	9553	137	1187	11880	494	5586	156	38351
1378	30726	778	2342	56342	816	2914	312	22970
3782	104346	495	15062	179226	3514	3882	840	119911
222	10813	98	853	15703	282	734	116	11709
496	11832	278	2058	25505	616	1869	97	19127
301	8229	272	1064	18137	509	2591	113	19834
1280	30893	1085	3151	66077	858	2413	281	22723
2129	41805	1841	7794	85955	1871	10292	482	184356
1760	36485	1562	7909	88548	1856	15303	494	195236
2450	67280	2471	9404	107025	2239	14254	517	187142
2902	46939	1734	6199	86462	1606	4448	1720	45409
1934	42560	1315	5242	94429	1601	3690	1180	38474
1763	36744	1326	7185	88414	1621	25708	965	260666
1726	37198	1476	8045	97495	2012	12339	414	108290
2667	28550	1934	5451	86317	874	14756	2323	168355
2634	31056	1042	4960	141282	2202	3205	378	34046
4533	18708	1579	5561	97509	1926	3406	1446	42656
2091	25240	784	3844	92747	1792	14838	282	170000
1817	30733	1119	4682	90424	1570	15720	288	166322
1565	47350	614	3878	66360	1216	16515	334	184239
921	14507	480	1175	38843	446	4810	235	28635
1902	15062	977	1061	70735	522	7299	343	52554
1952	25483	955	2325	91528	726	3828	296	57282
1975	26747	1488	2502	70312	1089	4619	392	42398

3383	38090	1913	3847	105044	1283	5039	517	53294
1080	53176	914	2173	77392	720	1969	877	15179
1071	44475	1909	2023	79234	777	2148	1088	17703
1362	61893	1926	3633	64310	1130	4474	417	45244
1538	51644	1731	3399	67057	888	4629	406	42543
1672	44344	1568	2949	67374	701	3948	470	35015
2080	37255	1376	4302	134933	1191	3113	533	25015
1258	27662	918	2599	97340	564	2184	293	18286
423	11393	382	1255	35722	489	2112	136	20637
2288	50234	1493	3323	104635	959	3862	554	35769
3157	64795	2013	5020	118945	1363	4697	731	54035
2188	38183	1103	2585	76114	679	2507	396	20734
3411	48341	2227	5098	150339	1067	4219	637	36381
3034	57113	1897	6661	127608	1943	26555	488	295979
4837	73158	3005	9753	150215	2375	23772	897	266164
3271	53312	1383	5619	82793	1565	20269	449	227242
3313	42771	2147	4976	208037	1671	3438	488	36965
3779	55455	2116	5211	174918	1963	3801	484	39876
4328	83458	4035	4963	178360	1238	17958	771	197067
3029	70374	3939	5436	150798	1488	17151	555	142247
5828	59765	3253	5493	177786	1418	19256	1014	214808
8626	66763	1606	5132	168084	1728	4608	870	45943
10148	37891	3836	6895	206934	2297	3887	833	45808
11512	141497	3333	7432	252085	2698	20475	833	202691
7081	51259	3006	8123	227059	2726	15816	603	177396
5971	38821	3207	8782	161833	2963	16046	628	186211
449	20177	212	829	27280	713	9424	496	34180
446	11797	393	703	18981	525	8836	296	42250
857	23426	261	1362	32234	614	4468	339	35348
532	18020	319	1552	27391	663	4512	134	39942
926	18638	327	1864	32542	886	5491	419	48389
1994	15178	1381	1662	77482	1135	8308	454	56442
1285	21271	1010	1170	45220	814	5865	589	42056
343	9224	84	1216	4244	261	4510	113	62903
1328	27449	691	2217	51586	965	4916	292	47244
1809	26695	1594	2182	57844	1181	5566	469	62955
798	11063	1226	995	41674	551	4782	334	37157
755	11498	1113	997	38005	487	5420	226	37306
501	9102	375	1310	23441	632	2151	186	32363
910	19049	696	1887	53652	1087	2341	544	18009
946	15369	1293	1806	44121	811	3613	291	23328
2098	23794	930	2891	67179	1526	2960	679	31410
1563	25995	932	3309	69724	1656	4631	572	35769
1506	34037	686	2950	52739	1120	3574	554	34171
2312	32125	1332	3318	78914	1175	4373	471	35236
3342	30097	1212	2894	76125	1293	5421	616	54082
1212	15480	676	1899	42626	1085	4971	361	35368
1066	16250	713	2182	38863	945	4024	527	25455
1285	16450	1392	3501	66587	1172	3002	907	43265
3653	36475	1248	6386	87871	3431	3774	1192	53533
1416	33819	1086	6672	61960	2473	3912	1880	55026
3131	26554	949	3522	59832	2287	5808	2137	55329
2652	19111	725	3638	48997	1402	3560	1905	39936

4780	20330	1217	5941	60630	3253	16854	3434	224472
2993	20063	771	5351	63751	2289	12379	871	177724
1121	14016	251	3459	19027	1293	12615	836	164884
4193	22516	1307	4235	81158	2287	4456	3922	48502
4192	28113	1287	4908	79420	1905	3863	4561	43545
2414	18440	1232	7852	94080	3199	19057	745	215331
4009	14563	1404	6460	96049	1908	22746	2093	247777
1840	23834	1286	4980	78476	2162	13454	459	155809
743	20326	607	1299	39707	862	5956	198	45529
1554	19097	627	1494	66637	1077	4192	558	30855
2264	20120	877	2058	78895	1366	4312	575	46657
2513	32105	691	2469	84889	1498	3048	363	51536
2262	19118	747	2153	71444	1639	5090	329	55479
1490	11824	623	1682	49789	1151	5637	218	32763
2117	42857	742	5039	87236	1711	5330	338	42697
1066	17293	414	1911	35904	894	4009	189	35639
1447	14769	625	1428	57931	988	2263	202	21856
2333	26894	786	1761	71622	1220	2842	507	28912
2367	28646	876	3319	113195	2118	3472	750	27833
1834	18459	783	2995	91280	1469	2743	453	22824
2121	18479	732	2034	72296	1144	3725	595	27562
36	24	12	52	543	2	18	79	213
5506	21689	836	1802	151714	900	5747	451	88724
2800	20407	929	2331	95983	1611	5541	442	44338
1863	24821	865	2429	102090	1486	2747	479	26656
5721	34322	1339	6610	138134	3253	5338	952	65374
4153	39273	1089	6991	132913	2466	7929	1301	70510
4076	24914	724	5493	102261	2623	24876	928	272445
3087	25402	937	3935	71346	2833	4119	888	44912
3988	35956	1218	4766	130268	2574	5077	905	54421
4540	47843	922	5517	114967	3313	15050	866	163438
4144	35183	949	6346	117773	3465	14507	795	95373
1839	31786	629	2217	86703	1238	9242	288	82543
8204	20544	1682	5098	161629	2399	4189	3552	51181
6891	42216	1423	5294	190452	2940	3699	2060	41824
4767	17520	1355	4764	134910	2883	18621	368	209031
6076	27817	1271	4592	178081	2644	15261	667	169871
6382	30993	1211	4121	165280	2702	19938	826	222395
3019	67440	8525	4592	48946	1850	10471	1933	128415
719	29794	3078	1450	16756	897	4488	666	60483
2539	42112	3813	3109	26856	696	67346	1398	447973
3224	48433	3937	2505	24304	925	48397	1095	296924
2146	49823	3632	1749	22246	1003	29619	817	193620
1054	36839	1617	1742	16983	791	1883	367	24663
916	42852	1580	1306	13351	392	1931	315	17054
4302	160027	9300	6890	63805	1791	5103	1778	122526
4290	135684	8367	7289	55566	1943	4225	2220	104396
3215	128380	7442	4660	47316	1526	3382	1288	99320
17	15	5	23	28	12	4	27	28
4013	111246	6430	6705	45853	2190	4277	2183	110260
4212	137509	9613	6109	59303	2176	2435	1167	84455
8406	312373	11122	7115	80862	2369	2584	2442	80170
5079	183884	9742	5990	63979	2221	2731	1424	87995

4624	140442	7274	3727	45851	2393	5549	3827	108345
87	4	9	43	16951	35	5	8	5
2247	102129	5695	2503	46550	848	3878	1011	62218
2325	102522	3681	1885	41407	583	3214	963	54564
374	14979	652	458	8594	169	3194	425	52674
3657	104883	6146	3818	37254	1914	7482	11421	159651
3843	156988	7740	5369	47761	2333	6425	11903	142452
1556	101528	5225	2654	41750	1089	4040	965	84505
1058	48715	3403	1772	26348	774	3365	896	74040
856	49763	3245	1520	27404	675	3346	518	76326
2460	94680	6974	3231	36689	2216	6800	1776	142648
3378	92167	7171	4413	45434	2155	7333	3047	160863
1978	124772	6724	4772	42021	1134	2052	2109	78908
3331	102921	8537	4610	53013	1167	1820	2865	63907
2141	129365	5101	2990	30762	847	1428	968	61055
3093	76967	7224	2658	42368	1901	6471	3253	139631
719	29948	1817	1096	12999	638	4489	924	98648
2841	69093	6566	2318	49876	1032	2448	982	91564
1178	120031	4151	2074	27472	618	2641	644	84166
2556	70846	6080	2500	40272	1026	2882	1539	96046
91	2395	185	89	898	128	57	189	1176
59	5643	139	80	391	28	69	48	425
7870	148497	7052	5886	59546	1416	3521	2662	98922
7940	251004	9820	7910	68592	2264	6352	3225	110889
4028	101363	4482	3378	39684	1155	3601	3319	69230
9984	187649	7194	6042	55010	2608	5095	2906	109062
5255	143501	6678	4317	42998	2004	3412	1972	74033
5761	205994	9125	5660	55010	2284	2490	1668	97213
7314	165325	9884	6970	66517	2537	2716	2107	88649
7467	169157	9982	7401	72176	2517	2646	3966	96300
6856	238364	7975	7731	89111	2897	5231	2682	106496
4134	252232	10036	7122	89940	3225	5423	1780	141420
4641	189306	6149	4123	75149	727	3615	1232	64552
3533	206250	6228	3895	84650	943	3360	1179	66156
3678	195066	6688	4227	101237	909	3164	1411	63609
10446	248509	7082	8861	71125	1879	6637	4802	157627
15293	208577	10037	10418	79173	2637	7811	7450	177875
4858	141197	4618	4359	64138	951	4508	1771	91639
4393	150101	5804	3449	63495	837	3966	1556	87767
4100	191121	4987	2389	74526	722	4262	748	86957
2070	230340	10530	973	96212	476	7731	379	126104
5599	204586	8057	6519	93860	1666	6277	3597	150420
5955	245075	8991	4753	131560	1127	1834	1852	71406
4431	240104	7422	4434	89288	933	1720	923	83687
5083	245975	7193	3455	96278	863	1664	957	66647
3691	121491	6667	4350	71422	1397	7353	5767	181414
8549	278993	12274	10301	190871	2384	9388	4336	200385
5017	168380	8208	5052	130995	1202	3095	1680	113743
5627	226783	8144	5076	112159	1099	2897	2350	102050
3831	200690	7825	2943	138101	919	3305	1267	121417
2050	55187	5531	2753	34064	1166	3902	722	58148
1834	57735	4804	2190	33391	852	3173	863	40077
3338	74151	10015	5131	64815	1973	13162	1003	138961

3515	66953	6414	3264	49395	1082	9599	1442	128254
2524	51165	6335	2579	35549	1431	8183	1663	133869
5748	111970	12210	6748	77167	2113	4325	2232	112633
44	4	17	26	26842	NA	170	12	1910
4158	53102	6423	2700	31700	1053	3030	1704	91844
5870	137954	9164	3663	46385	1388	2708	2507	92858
6137	127601	9602	4426	49162	1476	2880	2720	92970
3459	116332	10205	4690	48010	2095	4159	3934	86832
5690	131981	12079	5426	63546	2117	4583	2402	99609
1704	138373	9662	2417	52082	840	28575	1094	160802
2415	174072	16740	3888	119848	1383	25921	1404	149294
1440	59249	5554	1376	32785	631	10763	985	77447
3892	113525	8373	4017	47773	1671	5758	2072	104182
3797	150708	11936	3202	60429	1828	11479	2435	170693
1970	63262	4551	1389	25789	792	4950	937	85419
4945	113998	6271	2961	32444	1903	6275	5096	133245
2679	78192	5824	2173	31961	1817	5514	3097	118533
3022	81839	5017	1996	35073	888	1423	1051	57805
1928	92155	4888	2023	32864	945	1945	1259	78568
1699	39549	2766	1213	19401	587	1288	575	49367
4455	98219	10018	4852	48422	2030	6338	3969	129055
2301	82216	4735	2426	27535	1170	5496	2172	114543
2554	88860	4843	2477	28956	793	3069	2095	94888
2383	83883	4890	2372	27728	786	3035	1971	92106
8466	121307	7393	5100	34216	801	3620	64295	125504
5491	92529	7111	3014	68639	1502	6197	3605	127166
7327	64437	10979	4465	64795	1810	6916	4232	140356
1020	34540	3560	1159	29439	734	2727	858	61348
2333	45734	2709	1121	18976	485	2210	1052	50163
3375	54510	4276	2047	28935	840	3950	1761	78463
2623	48932	3439	1750	21258	1416	5573	985	113830
2017	67745	3270	2017	32797	1448	3071	546	57413
3121	70679	7095	2004	39754	1337	3035	870	104710
4126	79622	8622	2736	63275	1958	2851	1366	96523
2852	70949	6759	2095	36731	1341	2943	1105	94376
1907	55313	3548	2314	21545	962	5165	1187	105474
2912	102503	7396	4177	42621	1769	3804	1335	93975
2890	15050	741	1037	6769	246	6134	6378	63624
1110	63319	3328	1141	22801	446	6773	613	63609
722	73225	3850	1334	32095	483	6493	640	71328
5731	139284	8240	5330	65959	1765	4356	2825	91522
488	56	7	119	20	356	2585	15	54327
11850	237219	7384	5851	71615	1779	3616	3334	77297
6437	171325	8838	4926	82806	1257	5189	2506	80505
1281	74702	2977	1122	22608	408	2775	467	33194
2113	77185	2116	1664	20723	353	1478	896	25651
9191	188313	6084	3965	56296	1479	6343	4171	137427
6568	154325	7953	4429	66740	1730	7716	6809	157022
3609	91757	5934	2703	54355	922	1477	1163	56502
4134	109557	4329	2604	48004	788	1360	1239	54611
3821	94645	5087	2764	39607	852	1573	1167	63960
3860	211900	8023	3509	68743	1494	6404	2129	133072
5875	109604	6157	3882	70937	1319	5030	3094	110037

4700	165878	8185	4460	75633	1071	3388	2172	113125
4110	115709	5719	3343	54116	969	2959	3477	96048
5356	249791	11036	5390	104557	1406	3378	3043	112552
10796	182403	14136	8595	209882	2618	8211	5579	181172
3680	65424	6222	2341	60237	1519	6647	2102	140461
2826	117194	5749	2575	126380	1430	2659	919	56099
5304	172635	8053	4229	118221	1156	3142	1827	67003
6420	197468	9996	4838	139130	1408	3958	2420	86751
1295	18250	2845	1423	17592	940	4067	652	52993
931	9693	2256	823	12745	812	7290	512	92923
1805	15701	2474	1159	28786	1103	21287	411	157875
976	7442	1218	708	7462	531	14803	542	153641
1292	12344	2649	1253	23426	1289	10994	305	110043
1010	42150	2792	1323	27388	1211	1433	320	27681
1159	44085	2465	1694	20064	1162	1390	624	31623
971	47662	3683	1773	24425	1388	2248	1474	71595
2746	63579	4460	2474	29043	1667	2649	1852	83644
3644	88929	6114	3449	40749	2144	4391	1561	138672
5528	115228	7466	4880	53173	2984	3571	1712	79560
3741	71138	5286	3975	39175	2347	4221	1912	97746
2929	66605	5374	2637	33587	1995	2391	1590	80083
692	43058	2995	1003	30302	681	72836	1056	286156
821	52731	4465	1153	45885	757	73065	483	302646
436	77407	3816	867	38315	713	56653	952	226695
4515	102977	6154	1374	45517	2547	3743	2049	83855
2654	99581	4495	2303	40114	781	3106	1400	69711
3116	75951	4598	2261	44158	755	3651	1524	69069
3968	85695	6685	3113	63142	1281	4099	1674	79826
4429	62337	8030	4077	47476	3076	7307	7774	162677
4965	102974	9344	4301	63685	3300	10547	4890	232637
3009	56284	8162	3475	64696	1416	4251	1764	92177
3004	78769	6354	3880	50326	1178	4433	2083	95739
10165	232496	9209	5860	108797	1678	4863	3532	105220
4007	60588	6276	2625	48370	2706	6830	6257	144233
8042	75603	8806	5932	55936	3223	5320	7549	116481
5318	73334	8138	4820	60521	1982	1808	3039	66631
5243	104627	6172	3679	48805	1632	1872	2576	64017
4582	96028	7043	4027	51312	1985	2284	2561	77106
8195	55898	7909	6534	45069	2857	8729	14636	166075
6661	47645	6518	4399	41777	2237	7647	8115	147050
2677	65849	4477	2087	32103	1143	4062	1765	80926
2566	60502	5168	1920	32520	1094	3673	2043	74676
2404	69759	5197	2095	37178	934	4145	1338	86584
2400	91780	2779	3083	33330	1258	2213	1253	44472
2408	84623	5118	2967	40969	2287	2335	1283	44724
2019	54676	3779	1808	34385	1627	3350	1548	122892
4245	84852	6434	3489	48605	2053	3751	1732	76463
5943	122587	9300	3560	75365	2982	4573	1811	86202
3808	72572	5086	1889	40313	1938	2448	1220	61476
7461	210830	9686	4591	81387	3929	4762	3283	101293
3391	143025	8945	2902	103487	1665	61433	1622	255962
2113	89164	5633	1599	58649	1210	39061	999	197804
1633	102015	5708	1629	61083	1238	26152	936	153089

3906	120082	3746	2334	54202	880	3230	1861	61562
2325	76138	4278	1684	55799	1005	2579	1034	54067
19	3	23	11	2672	NA	12	17	117
8281	116971	9040	3603	76777	3141	5912	4288	129681
10156	217901	8481	6577	80983	3312	9106	10302	180060
523	236	NA	32	103	189	4029	6	75617
1046	169203	6486	920	77929	1732	1904	195	44395
9767	161368	7253	3880	87700	1747	4527	3064	99302
6398	113786	7419	2856	56829	2881	7460	3471	161575
7619	222414	7253	4138	55071	2551	6475	3200	142030
6117	147108	6399	3828	65601	1501	2798	2075	81103
5185	131203	6388	3742	56012	1564	1679	1894	66342
4452	157533	5698	3101	46728	1341	1487	1558	56754
9532	133847	8967	6565	88866	3008	9339	5239	180592
4022	65714	3932	2718	48033	1612	7074	3447	144729
6529	129927	5851	3320	57252	1193	3648	5217	133044
6013	153059	6183	3796	73883	1388	2483	2128	61607
5616	141005	4593	4011	66522	1062	3028	2959	97854
3127	7161	6897	4211	106618	787	4181	18106	162564
1851	5518	6054	3496	106035	452	2656	14447	93468
674	21	63	84	86	300	4975	216	98847
199	12	4	28	21	147	2350	NA	46533

Phenylalanine	Phosphoric acid	Proline	Putrescine	Pyroglutamate	Pyruvate	Raffinose	Ribitol
1395	8300	59196	3482	138162	731	494	40461
1055	5679	18419	2446	61729	906	110	23208
2366	12264	101921	3961	149625	3669	550	34197
1821	14700	65859	4533	115659	3829	324	31777
3505	5752	195262	9238	191192	7063	545	36469
2472	3017	122857	6579	77157	3147	1592	22782
2514	2738	129556	5454	88334	2231	2027	28344
7365	14825	262196	13607	250680	17334	1354	63337
3393	5571	214939	8692	166391	7408	455	31496
2344	5409	64493	4205	101859	7260	578	31672
3523	1949	60190	6400	147134	1700	2990	39364
5432	4668	173254	23992	262776	4737	3559	45884
5112	9003	103442	13111	264286	6453	8261	53391
3365	18277	52131	5905	153686	7526	6521	41604
17	8	2033	2	13	3	4	15
4243	21473	146547	20119	178120	886	3333	42290
2865	1319	199032	6487	147099	770	2625	40146
2756	61633	69885	7350	201170	2410	1545	44290
3656	72193	92908	18956	313883	4035	2532	46277
4646	47217	63357	19400	266606	5469	6017	44881
4054	16880	187586	16422	159123	2581	2037	35647
3734	8902	171333	7007	188419	545	1678	39082
3826	40036	117301	17575	336138	2979	2965	54139
4982	37324	97211	13726	338602	3424	1803	49830
3827	12415	76963	23209	226453	1427	1418	48786
5306	13416	212487	70919	364331	3192	5210	42245
4023	14699	246224	24147	247802	3221	2040	45018
5527	38427	703322	33117	631631	6649	1798	58356
8023	42028	475613	62591	734021	6804	1376	59309
7213	16511	181412	35875	505787	8190	3706	61980
5641	15847	102552	33455	193107	3824	4966	53041
4450	3490	143888	15514	186165	4342	4843	45700
4212	22135	84422	23279	249515	6124	3536	48163
3936	5303	86209	17398	231223	3811	4858	46642
3950	7431	209090	37768	298836	7551	3135	46789
2092	2871	85762	4249	74816	3021	2382	26027
1379	4258	90905	2618	75752	2239	1474	14856
4652	12261	100767	9591	169896	4915	960	31535
4578	28605	111884	10465	194134	5108	3850	37181
5030	44382	139583	8796	328606	6054	1766	52915
2728	2097	145216	7723	119528	3163	5682	36868
2901	3024	104323	10952	87687	3838	10789	25646
9435	25058	223357	14952	385286	13714	2941	72326
5994	44575	192716	12931	328439	10068	1914	67923
2657	17131	160936	7960	155843	6430	879	34056
3562	6005	277429	8233	139014	522	17591	39857
5675	19214	609888	9816	209256	737	14364	39885
12069	75966	916067	14543	475681	2359	34685	49971
5938	40330	322633	10154	359644	3712	8900	50813
8863	53144	707416	17781	453872	3265	25930	52000
4315	12562	1E+06	6609	190138	635	41419	44366

6468	3126	1E+06	11525	277728	635	26898	38792
8324	36171	2E+06	9718	376764	1835	89415	45172
11028	27519	481312	15731	383496	8611	47718	55594
8462	40684	384271	10008	423899	7030	100325	47963
66	4757	5534	180	9023	220	150	81
7876	43340	786428	10888	230716	1996	161480	51951
8692	12242	850012	35908	479562	5756	35193	62292
14986	16326	670167	13895	452146	5644	156844	66197
14353	118215	658924	17905	575764	6402	145764	67368
9753	8947	2E+06	18190	209475	4048	119501	42890
12257	9144	2E+06	25751	340663	4930	168802	59557
7372	31326	3E+06	18114	456059	3332	134648	54655
6929	71486	957477	14852	396343	6863	99995	46638
6486	109783	1E+06	15438	390442	9049	334512	54296
1719	7764	87655	4159	73973	2455	686	18133
2062	4652	139877	4654	71859	1855	617	21669
6015	21332	187053	9196	278759	9178	1467	56438
5302	29447	282146	17466	361986	6863	1311	49084
5984	23200	270247	15384	269354	9131	1214	36038
2318	2077	302348	7452	79095	2281	1502	23102
3808	3378	420441	13651	153868	1801	931	37285
5284	8618	356475	16739	265606	2488	939	49506
6365	16612	480960	13150	253379	4520	3349	56319
6693	41991	708575	16412	339112	4711	2534	55231
7583	3455	598215	9755	160478	3757	4199	35775
10298	1621	586098	14610	154022	4548	5665	44845
8777	3782	727157	14199	241223	8316	4114	46268
8414	30174	341005	18771	367840	8259	4047	47004
5175	49236	293449	9405	248013	7835	5415	38326
1099	2814	76610	1917	60190	647	385	37943
4176	9197	444191	8850	156745	713	3138	38357
12968	166517	1E+06	19500	569775	12955	1073	86528
860	20381	143280	1967	71277	1744	266	13563
1397	36682	468608	3653	111816	1460	548	14618
967	7454	110492	2426	60803	1395	729	8070
3874	18521	280079	7307	132023	4007	3656	45210
7080	18957	427296	16775	336490	5441	2959	59004
6484	25600	436608	16914	391296	4833	3594	57411
7714	149547	651591	24337	462789	9708	6515	65291
11541	86982	923807	44215	380526	8443	3215	49432
7736	39412	767690	23552	266242	6519	4910	49728
7098	26707	552505	27049	466968	8256	3508	53522
6284	40395	694118	15916	310776	11346	3765	51198
12004	25870	552780	46482	645026	9425	632	56790
5819	4212	411517	13892	217413	5731	10434	46129
8577	8879	434490	39838	357292	5064	3789	49745
3084	52682	322990	7507	294685	4091	5718	41687
3328	128918	632555	11515	269526	5882	8297	35260
2397	203203	479920	6374	293070	4573	2495	33872
2629	3102	251432	8782	106284	582	1737	29051
5114	1918	241281	13700	158210	1721	2781	45636
6191	11559	313827	8955	255262	3474	3344	45319
7927	46683	479097	14787	308045	5307	4061	53604

9573	27944	321706	18483	361456	7215	6106	54385
8362	1568	450662	16816	367110	2689	6975	48392
9633	2036	570252	26038	381859	3435	8262	49314
4341	201700	612003	10802	374760	8685	5461	41795
6536	62169	313127	13376	363348	8664	6591	47851
4827	84031	343267	18302	315767	6901	9770	51538
6714	22052	2E+06	14511	221953	2444	25563	42405
4396	13572	816897	7264	130813	2075	26095	29174
1582	23065	275907	4666	120421	1919	3982	11394
6422	52070	837500	13851	302298	2086	16549	36742
8942	103195	1E+06	14223	427835	4358	21084	42192
5392	41916	910519	12194	152036	2735	28853	25213
7996	31248	877942	21312	228286	4242	43075	48513
6977	15227	638809	19419	422768	6101	15968	59118
10886	46397	2E+06	26046	538236	7878	22463	64619
6670	27948	961836	14386	420282	5309	10210	36681
11410	6520	3E+06	25335	221471	4342	146534	47956
10921	28866	3E+06	27706	316448	3967	123750	53004
12883	63747	2E+06	26613	583853	5367	96622	63469
9655	74772	2E+06	17163	519823	10891	62874	55305
13928	41905	2E+06	14086	381775	7299	150715	50628
11750	44808	4E+06	27768	219331	3958	125940	35240
16632	16597	2E+06	41420	212142	4621	163626	51551
14005	160031	5E+06	37575	536829	6366	303465	53515
11373	11444	2E+06	16603	367535	6194	197657	46531
10421	53404	904524	17639	435326	6608	32694	51256
2689	6225	27740	3367	99350	2083	128	29270
1185	8240	39580	3616	64360	2147	125	19586
3048	18942	26955	3629	150579	3795	136	39051
2611	8511	29979	1410	114819	2050	197	51488
3835	15589	25648	7706	167264	3823	221	50452
5271	2746	51179	7195	131333	2658	280	26628
5532	16187	35239	8033	144928	1698	81	35927
990	13606	5353	408	116454	472	36	57836
6734	13755	54657	4315	189289	5355	305	44652
8079	6970	59821	12120	246161	4783	186	45852
1668	2258	43144	6759	88471	2379	849	23992
1264	1914	36926	4023	65873	1180	354	25680
1320	2204	31482	3820	133869	3477	161	32489
3341	9453	40616	9702	282999	4563	176	39541
2586	7192	37907	9371	156748	1983	527	29393
4445	22344	81603	14746	164147	488	1010	43652
4289	31987	82906	11865	156976	2806	676	36781
3356	69444	53382	5229	238724	3863	508	45446
4690	68052	62733	9663	276124	2210	454	44128
6029	80516	52231	14559	306067	4511	412	51924
3412	12250	47187	10624	122474	2400	438	42976
2287	16905	45846	7360	108967	2441	132	33186
4287	20095	72143	19773	260053	1750	666	45902
8351	40086	55743	16499	452570	1842	322	60457
7343	22359	79458	29925	304625	2767	835	61071
6272	90287	89500	40234	245512	5117	2944	46115
4933	36320	85625	28774	236366	3658	130	46877

7952	52481	106432	65681	560151	7784	1007	58587
5990	33713	107626	18107	330176	5552	465	60271
2025	67922	35702	7836	204890	2744	87	16272
7827	80226	131277	39650	364715	8188	707	46309
6774	65409	109332	38568	291025	7243	566	53379
5287	54349	101559	20740	273465	9775	3261	44370
7315	29090	114803	22427	299333	7166	1217	48971
4738	38237	71067	14406	264087	7363	1744	52067
2149	2976	41615	3001	85281	2258	240	28147
4345	13983	54829	7763	116858	2237	353	33166
6381	5691	53110	8883	222807	8329	429	43085
6685	16069	49838	9043	231639	5871	493	47849
7087	11669	36317	16040	212917	4246	1692	44624
2251	1873	36593	5482	65189	1944	2805	23945
4184	2174	56933	4272	91372	1425	2568	36909
2609	16575	24335	5183	118042	3026	1074	11242
3083	10964	46294	4112	131349	5204	657	31440
5645	8954	49328	5194	210371	6097	658	39763
5606	39090	162947	9890	189771	854	2085	41094
4324	14454	172132	8196	144064	2648	2030	37390
5515	19048	83876	8181	327588	2121	1625	45797
27	13	1557	17	7	36	19	2
6149	37767	85501	8592	313739	2975	3216	58502
4461	31529	98916	14820	162022	4351	3800	40085
4158	35550	93922	12206	162150	3476	1651	41999
9962	67107	186822	16722	531808	2763	5071	52893
7475	27979	279627	17540	520767	2565	2175	64113
7344	47085	139386	17611	446000	2283	1558	44931
5333	67317	142896	23313	280792	3073	3318	44683
7909	41799	138435	17142	241997	5699	2303	50131
8802	113735	127386	30419	533607	7277	1745	55643
7843	116207	161027	25252	541294	7337	1992	61278
5622	41146	59390	7982	321152	5713	2610	53966
12639	15393	672355	43110	372930	4868	22440	53588
12374	80293	1E+06	31694	346356	3275	42934	54092
7046	10863	115783	24978	329844	6286	15160	36014
10383	39662	239236	38312	432576	4894	30636	54885
9729	17795	235414	18333	401981	6909	16804	50080
3715	88011	40748	1434	1018536	4261	1318	130496
2010	52095	16124	394	310940	3392	404	69867
2078	20263	41757	2958	609115	3214	622	105022
2682	27011	44935	1942	413110	2140	541	106734
2219	31402	38981	1174	352133	2485	537	103466
1675	49451	32854	555	425377	2084	347	46817
1390	50669	19530	305	255301	1559	51	38317
6804	147517	58049	3107	1253616	6388	1741	110609
6775	139450	113305	6477	1319858	5451	1576	107713
4723	80556	44484	1824	828345	4883	1204	104148
3	49	11	4	4	32	9	4
5081	174085	48879	2670	1259217	6537	1858	75265
4995	168587	48535	2323	982804	7893	6532	114134
8997	225583	66278	1951	931070	7324	4411	145537
5961	207149	56759	2593	1067539	5828	2751	121107

4468	197772	75878	2457	762961	6035	1081	98953
12	21	3576	29	43	NA	22	12
2838	150965	9506	1737	1010071	4737	2934	84225
1757	98520	15630	659	584838	3768	1035	79468
444	19325	3987	431	158727	586	389	10344
4587	129106	48481	2193	836861	4848	1718	107997
5950	102759	87065	1433	1265312	5783	1913	106089
3263	105914	35098	1191	1049459	3688	3062	113958
1849	40287	11694	484	582788	2948	1379	71183
1580	51086	6475	486	469548	2956	1442	94771
3524	192701	27492	5143	911311	4955	1481	110726
4580	201859	72362	3085	1236434	5786	2461	111452
4000	213115	38355	6600	1742419	5366	3945	121047
4125	580574	21407	7053	1853022	7678	1256	116822
2802	104823	25333	1928	942422	3729	2215	118375
3094	107447	26732	997	485046	4224	2808	100903
875	80506	23091	337	158880	1099	933	97185
2797	154757	25036	1112	507546	5475	3404	112347
1895	82734	31719	325	351609	3306	2634	111778
3264	138345	19183	3522	960010	5921	4478	115006
129	9303	999	52	17016	88	15	1366
23	2280	693	18	21570	41	29	3506
4450	189540	113895	2008	749915	4414	1120	95240
6933	306022	110211	5618	1140100	5553	1813	124668
2833	84692	60095	449	604972	1291	708	103053
5948	264071	122459	2769	902332	5819	1360	103023
4732	209732	63843	1557	736826	4005	1274	89011
5075	174221	69579	1871	906722	4140	1470	127182
6946	291760	94673	4172	1370897	5734	1881	125885
7084	201062	128864	3102	1256633	5763	1875	122132
7144	183380	528522	1472	1501201	6953	8735	125663
10111	NA	NA	310	2716884	159	4603	121502
4328	96500	129496	631	1278179	4471	3450	84570
3670	170542	123933	794	1092370	4844	17913	80434
4487	124399	519929	1015	1515421	6477	19590	87642
12932	168448	945897	1739	1325490	4652	7521	109927
18754	178193	891550	2976	1321499	5673	20778	113637
5137	125805	550266	714	909609	5234	30797	91470
5233	128484	147304	490	1058340	4327	24265	116303
4214	46067	275605	188	602255	5017	34848	104640
1784	NA	31811	NA	1583472	377	12450	141609
9258	91641	1E+06	821	1151193	6280	18820	114233
7154	147536	175139	502	1318113	5703	52719	132040
6529	95279	225042	570	1005540	5652	29002	129686
5231	155301	228561	686	493184	6831	59299	117638
8209	144209	707629	607	525914	7453	102841	125945
18823	411607	4E+06	2600	1865165	11580	129806	132409
14123	141806	789689	368	1174929	11422	166965	120484
12041	195215	580710	525	1062433	9419	55889	131888
4695	119399	784832	341	658970	9096	114977	115362
3105	257980	63716	1808	803124	4165	806	100076
2490	116918	50767	991	630290	3125	576	82082
5520	86925	94080	2735	1174937	5564	1438	112905

4134	84894	126181	3189	673819	3743	753	118625
3529	55969	48645	1945	543987	4278	1038	103491
7520	174456	292026	5977	1633229	5977	1240	140773
9	13	4560	35	15	13	45	176
2952	85084	162224	3540	553618	3502	752	103752
4722	141820	173513	2508	662754	5192	925	115460
5703	121714	232158	4027	939584	5118	1010	112915
6586	167573	206588	3541	1398007	7309	1110	109929
8829	190238	325126	5426	1507702	7531	2038	116734
4000	110193	47956	96	1606123	792	1697	130422
7926	221291	39443	NA	2487436	389	4849	190381
2204	67940	34835	1122	683893	3810	1055	79063
5181	102868	76240	1351	1258404	5528	1618	98381
6185	203763	49859	650	1512334	2530	2122	176464
1824	76468	45296	1410	577648	2073	801	96818
4204	150708	128581	1628	795457	3539	729	109794
3004	116297	92455	1198	585141	3449	568	83825
2221	74757	33548	884	703130	2736	1156	110378
2123	66191	36241	593	805579	3384	1182	110341
1313	42334	27827	424	437978	1889	524	76848
5336	268815	110336	9625	1520392	6385	1576	110951
2594	134792	96448	1917	606210	3547	922	90225
2726	134911	52001	3010	976096	4501	876	118969
2710	127178	49104	2228	918366	4509	914	117270
12899	382212	37837	761	1826479	10432	1173	202852
3954	135378	133275	537	633239	6221	1574	112699
6402	169355	82358	6535	1303154	6377	1872	124444
1512	140919	56517	180	437156	3855	997	137026
1584	64879	64218	201	299475	2321	618	106368
2876	54314	86860	374	502573	3222	765	114432
2041	127338	84948	822	373234	2715	273	90015
2710	124647	66572	1041	559164	4249	260	70024
2775	125978	110239	2237	498631	3500	1168	113190
4577	116986	163229	2060	764527	4972	1408	113085
3504	87772	101630	811	508272	4433	863	109196
2138	61238	91277	1094	480124	3010	860	54919
4127	96887	143863	1908	967633	5270	1865	114784
1737	32183	21526	156	182007	802	351	68986
1165	36035	34986	588	476100	2019	1263	79793
1541	54786	41102	429	622495	3495	1992	78230
7525	149561	983577	2396	1318156	6281	5277	97304
NA	6371	1934	NA	3004	NA	32	14
9374	199782	641507	1168	997707	5646	3460	102708
8141	127479	416510	590	960994	6801	10365	113565
1528	49999	184409	182	316130	1667	2074	62520
2601	61977	131829	227	357075	1325	1583	40133
9667	135566	656639	934	835699	3401	8775	113989
7813	205221	603040	1372	1032404	5221	13012	96807
3440	70368	105410	766	681216	3837	7726	102891
3759	57479	235656	1007	722959	2366	3828	105423
3675	68784	163348	815	775566	2581	2812	102616
7552	132862	2E+06	1313	888358	7659	58787	123498
9488	92876	2E+06	925	889359	4894	45659	104588

6848	158728	648767	993	1399938	7418	55711	122653
6203	110350	939113	529	962040	6547	30862	116038
11918	185248	1E+06	640	1415499	11531	95521	146267
23639	246593	5E+06	1418	2488437	16086	167539	132042
6602	85690	531274	571	538120	6403	77269	119557
4655	237025	3E+06	760	1138695	10246	267336	116928
10664	220395	2E+06	310	1127396	11721	112767	125700
13255	253488	2E+06	309	1355364	13082	133231	144102
1546	42528	7893	977	378849	1819	442	72721
899	18669	4643	599	259515	1237	423	83535
1612	19611	8408	394	198362	2302	595	98210
505	7887	5671	281	111636	1327	267	88051
1539	14865	8743	475	227822	1883	918	101865
2644	88459	10258	1144	509529	2207	511	30214
2238	96874	9978	1165	505690	2224	255	49405
2504	165252	14640	1271	449943	2640	901	101217
3200	175102	25445	2435	640074	2990	894	79231
4663	236333	33491	3792	948808	3778	1340	112816
5131	395120	28487	4633	1383325	5169	4925	100167
3283	232399	23242	3056	1014176	4277	2368	98247
2858	283071	25753	2864	704407	3462	2327	113319
1156	147371	7118	291	639776	410	1564	102963
1555	164596	4631	188	837780	732	3091	103800
1706	117220	6095	69	704970	711	1115	117915
3660	237843	31605	1861	904608	456	910	109706
2562	145101	11182	493	865772	3376	744	87269
2606	108845	8810	1306	945681	2881	875	84334
3634	346239	9610	1336	1606523	3793	1207	114755
5372	381228	39724	3424	1202754	4261	1377	114424
5784	203561	42931	2636	1402543	4375	1953	119046
4663	383373	12070	2063	1612443	4588	2471	117719
4088	283102	14227	2904	1296890	3908	1287	116059
8703	407060	96648	1459	1612721	4483	4578	138317
4175	446343	30944	2658	987653	3748	1912	110862
8592	699095	50229	6112	2479095	4443	994	120351
4204	634012	15731	5350	1858989	3352	1585	122317
5038	440640	29097	2176	1856279	3041	980	115555
4348	337907	19253	2601	1262507	3661	1636	124953
9409	611629	51770	5701	1563389	3700	2288	124672
5958	498402	31515	5398	958311	4430	3221	100095
2880	240819	13849	739	715218	3053	2464	99679
3400	291774	15877	636	736775	4058	2578	104909
2844	151292	15473	605	663501	2825	2661	118902
3109	210952	20747	815	657599	3009	260	74937
3178	178105	24305	1269	696328	3418	644	85845
2562	71245	15401	651	394179	2271	675	104651
4484	109412	29962	1421	743000	3168	1312	102709
5913	262054	36700	2879	806227	4790	2244	103952
3939	285420	37287	133	839693	783	1251	70625
8216	241730	117067	1120	945161	5953	4111	94508
4509	322672	15068	169	1513039	309	2701	140622
3459	265789	12513	137	817581	754	1428	120613
3414	187706	9906	54	827622	725	1439	102563

2626	281484	40304	757	789674	2612	1364	87209
2533	108307	17794	300	716512	3210	6635	86079
4	14	25	60	12	81	16	12
6587	251720	161054	1929	1015677	4137	4182	117145
8053	370172	165318	2487	1416133	4671	2070	120697
16	6074	701	179	2283	NA	17	3
1004	NA	587	34	2469315	251	2345	99005
5847	231673	49812	906	1218846	5033	3667	104198
5342	369222	141868	1668	1098574	4491	2649	120918
5942	363959	87257	1340	863961	4229	3086	104385
5287	270087	66552	1058	1306368	3227	3798	125851
3878	395002	34311	2077	1288599	4176	3419	115474
4354	304203	45690	669	909413	3280	2517	113410
10716	955120	341831	8564	1997756	6469	16044	131846
4961	286296	417003	1611	713809	4539	13700	120906
5015	237244	110552	661	862781	5027	8352	112233
5737	376457	140403	1070	1282055	3848	15977	129963
5517	324512	219897	742	1140760	3815	6708	134949
9892	18882	284890	79771	695399	10033	539	63398
7936	10978	155422	67923	566087	9354	570	66592
33	3623	1215	353	1848	NA	9	28
16	1409	1177	240	1061	NA	37	32

Ribulose-5-phosphate	S_methyl_methionine	Salicylate	Serine	Sinapate	Spermidine	Succinate
140	NA	69	81980	827	439	2442
121	NA	51	46512	1424	471	674
233	NA	72	105611	1410	843	2583
175	NA	32	85497	964	730	2074
258	NA	71	141147	1280	2233	3071
241	NA	70	167878	2379	2341	2762
244	NA	43	190476	2612	2072	3998
206	NA	141	439460	3960	4590	9593
39	NA	93	252016	955	2395	4021
66	NA	29	141446	1412	1303	2727
379	NA	137	199284	4053	3101	1927
307	NA	140	259994	2604	4850	3476
265	NA	82	303107	1974	3164	3563
161	NA	93	185168	2606	2571	1830
8	NA	2	4	27	8	71
266	NA	82	253278	3338	3457	4839
174	NA	87	166419	2311	2761	4692
206	NA	101	144936	2500	3055	3051
360	NA	110	170840	3902	2819	4442
353	NA	105	169617	4667	4700	4214
254	NA	84	208291	3421	4322	3855
268	NA	114	186322	3439	2983	3903
237	NA	134	167881	2794	3384	2798
180	NA	101	172817	2676	4873	3526
131	NA	183	141055	2135	3191	2670
406	NA	97	302629	6560	4297	7108
276	NA	103	291217	4900	4731	4668
217	NA	147	500033	4954	4277	5999
544	NA	157	608200	7384	8559	6552
412	NA	236	340364	7335	5698	8600
317	NA	145	260596	5500	5088	4760
221	NA	115	262728	5073	4082	4461
280	NA	142	203594	4458	4813	4223
188	NA	124	176994	2779	2668	2770
257	NA	187	344455	4610	6156	2691
253	NA	108	155474	3067	1630	2716
211	NA	24	116933	3335	1078	2400
143	NA	77	176130	1983	2535	3286
199	NA	100	141849	3395	2777	3374
213	NA	121	217292	5287	3249	5033
277	NA	51	171443	4213	963	4069
422	NA	53	180502	6209	5632	3869
122	NA	191	388074	3380	4534	6962
144	NA	91	274282	2665	4186	6185
23	NA	92	170757	614	837	3609
289	NA	118	204039	3874	2140	3631
362	NA	84	242556	4816	3406	6424
679	NA	200	344172	5524	3373	7729
441	NA	92	181140	4646	3978	4732
492	NA	142	274779	5852	5784	6085
441	NA	88	306039	5717	3197	5215

583	NA	137	324539	6468	2463	8899
763	NA	148	472260	7299	4322	7881
345	NA	97	205272	7522	3046	4283
534	NA	106	404398	7348	4956	5302
10	NA	85	4101	462	11	152
665	NA	164	455103	11452	1572	10417
487	NA	235	406249	7664	3869	8382
763	NA	172	498440	8913	3323	9952
1568	NA	250	661269	17659	2763	19445
385	NA	170	465794	6363	2436	5900
605	NA	122	625660	12154	4213	6936
666	NA	174	318552	5705	1790	6708
656	NA	140	364727	7885	2562	6889
1007	NA	133	450254	14302	2622	10393
219	NA	81	113400	1334	2252	1297
241	NA	75	140059	1719	2405	1992
305	NA	50	193326	2320	4798	2958
372	NA	100	184351	2010	4491	3050
312	NA	130	245134	2471	4146	3926
263	NA	55	121858	3369	5904	2342
232	NA	111	180797	2790	5239	2539
176	NA	129	144812	2250	4679	2536
197	NA	117	211423	2281	5078	5285
213	NA	99	259830	2447	4038	3608
270	NA	84	397392	2968	5411	4008
300	NA	120	633457	4819	1971	9312
186	NA	148	351371	3216	6442	3572
218	NA	76	297409	3019	7362	3420
253	NA	124	257672	2778	6288	3090
30	NA	38	65323	971	399	768
225	NA	88	248105	1967	3553	3537
77	NA	337	702180	1179	10941	10934
44	NA	10	88498	262	648	529
42	NA	40	193492	722	1259	1693
95	NA	53	59587	1303	867	817
312	NA	61	193435	2608	4046	2536
246	NA	248	314780	3728	6945	4870
167	NA	177	267008	3632	7427	4558
297	NA	181	352334	4015	7603	5379
428	NA	115	486637	6571	12843	5176
345	NA	134	407665	4853	10301	4437
286	NA	124	389635	3958	8478	3946
319	NA	104	455395	3616	8480	4969
359	NA	176	399069	5619	16428	4564
247	NA	84	252837	4773	5866	5080
384	NA	127	348121	6220	11722	4323
243	NA	76	213777	2497	2776	3076
184	NA	73	172519	2568	4705	3121
150	NA	155	114309	1864	2085	2128
169	NA	48	57026	1768	2031	1187
246	NA	123	97554	3073	5483	1845
142	NA	68	123353	2228	4976	3117
193	NA	80	153339	3131	3151	2525

274	NA	73	230419	4929	8697	3843
320	NA	183	224139	2679	3358	2978
252	NA	171	422843	4653	4714	5516
290	NA	113	235405	3124	5519	3321
317	NA	178	273495	3313	7780	3686
189	NA	111	137886	2305	3031	1754
522	NA	162	381320	3285	5222	5468
304	NA	165	282272	1961	2886	2417
75	NA	73	82432	880	1158	1248
208	NA	280	282918	1821	3187	3273
267	NA	335	335642	2813	5761	5611
189	NA	115	231206	1924	2749	2172
535	NA	203	295435	4528	5993	3901
256	NA	142	312654	3985	6181	6250
446	NA	361	482838	4713	8447	8719
202	NA	263	350746	2499	3802	5063
472	NA	155	650520	6089	6616	3364
668	NA	164	605035	5879	5018	5325
583	NA	321	622230	5036	4992	4396
466	NA	317	448929	4235	4166	5494
772	NA	221	585267	5840	3533	5259
348	NA	122	565854	4328	5430	4032
567	NA	266	571586	5949	4724	8074
705	NA	112	646377	5427	6768	4357
572	NA	215	510337	4660	4833	6571
544	NA	206	345543	5948	4190	10336
133	NA	17	80132	1817	395	809
114	NA	66	62086	1260	816	1500
133	NA	81	75445	1619	706	1083
115	NA	157	67062	1479	124	701
148	NA	115	96589	3095	581	691
379	NA	80	185836	8486	2721	2697
185	NA	83	124882	3879	2511	2441
27	NA	32	18206	703	70	416
185	NA	140	180113	3078	1831	2337
98	NA	111	164014	3848	3407	2334
134	NA	26	118732	3554	2846	1939
87	NA	34	94296	2395	1708	1762
30	NA	91	85722	933	474	1261
105	NA	54	116007	2471	1287	1687
64	NA	49	116130	3281	1399	1622
213	NA	103	124392	4471	2669	4118
215	NA	129	135283	3989	2932	4920
211	NA	128	98067	2362	2376	2789
231	NA	109	143009	3591	5894	4046
191	NA	89	118631	3255	2824	2910
124	NA	100	114545	3514	1504	2607
176	NA	90	104211	3202	2822	1881
214	NA	157	239019	6093	6104	4324
140	NA	249	239327	4615	4727	7120
162	NA	185	226755	3447	6154	5925
307	NA	149	235402	8013	2974	3165
170	NA	80	252999	4671	4009	3306

341	NA	129	271332	12028	7091	5296
161	NA	74	243398	6561	3419	2891
45	NA	48	72310	2282	1747	1283
262	NA	103	347840	8995	10051	3612
226	NA	103	318187	5805	8640	3971
278	NA	116	192148	6401	4971	4154
302	NA	183	278664	5654	5179	3908
162	NA	117	156709	4070	2737	3580
146	NA	40	104702	3749	892	1945
166	NA	88	92224	3565	1978	1413
139	NA	140	141875	4640	1884	1725
127	NA	103	136565	2239	2834	2317
151	NA	121	96136	4614	3171	2010
119	NA	55	92697	3905	1340	1357
196	NA	100	132008	4960	1096	2053
69	NA	41	60549	1715	1238	841
69	NA	49	102135	1971	1562	824
161	NA	97	133342	3187	1723	1370
222	NA	87	152523	4738	1553	5327
322	NA	91	112081	3636	1832	3820
116	NA	109	106129	2913	1502	1965
2	NA	2	2	10	28	4
66	NA	132	90440	2438	3382	1648
267	NA	90	146630	5423	2019	3679
233	NA	49	135196	5418	2437	3347
259	NA	163	247113	5142	2213	6515
135	NA	207	248937	4988	1518	6236
131	NA	139	175727	4199	1504	4957
339	NA	110	199721	7412	1646	4891
243	NA	104	206746	6612	2766	6889
278	NA	155	265932	6291	2211	7719
215	NA	139	251455	4716	2259	6863
111	NA	94	113293	2995	1006	2006
491	NA	177	508081	12803	5987	4724
434	NA	116	440462	9196	2626	4831
279	NA	125	175816	5558	2885	3763
281	NA	178	249556	5052	1439	3371
326	NA	98	269384	5661	1892	5522
934	842	661	117336	NA	513	8245
222	146	334	41267	NA	234	3546
328	683	629	79794	NA	509	5508
336	474	395	68583	NA	344	5437
260	307	321	54176	NA	308	4519
221	240	310	61601	NA	146	3441
148	117	167	41493	NA	97	2243
804	1287	509	172447	NA	870	15155
753	1099	574	194549	NA	833	11797
585	1032	395	146182	NA	764	11634
4	37	72	22	NA	9	15
443	815	842	142544	NA	988	10000
808	1063	567	156699	NA	908	11930
829	1467	516	193711	NA	753	16639
848	1108	581	156069	NA	930	13364

622	687	696	106253	NA	462	11374
6	53	626	16	NA	38	4
466	645	447	69612	NA	214	8374
435	761	367	63129	NA	92	6114
174	241	152	16286	NA	48	1413
627	498	649	111355	NA	686	10440
649	759	715	147023	NA	594	13256
556	519	593	79262	NA	207	9264
325	519	531	53138	NA	274	5248
231	513	553	39498	NA	185	4947
406	400	992	81928	NA	535	11109
675	973	1157	121014	NA	849	11345
556	1005	498	113379	NA	367	11451
514	1663	434	155014	NA	211	11162
298	506	341	67386	NA	193	9259
654	565	1046	70777	NA	418	7885
219	227	764	37080	NA	149	2818
477	704	465	62397	NA	418	6850
205	355	445	68943	NA	224	6485
494	678	524	87574	NA	305	7806
33	56	8	2937	NA	26	196
3	15	24	1650	NA	23	135
719	1378	358	149559	NA	474	9235
804	1479	815	186338	NA	1078	13841
347	783	544	107485	NA	275	6121
404	792	663	147176	NA	569	10318
433	689	687	118625	NA	585	8521
704	848	494	150049	NA	547	11377
861	1134	533	180971	NA	868	12799
899	1066	566	191198	NA	823	13239
1116	1350	925	240721	NA	1200	16278
986	954	939	149463	NA	1296	22031
517	1261	437	142761	NA	179	10199
523	1282	523	150323	NA	341	9551
952	1481	554	202937	NA	291	11195
780	975	662	215199	NA	633	10810
1002	1494	724	247462	NA	890	11875
653	1661	564	166570	NA	310	7511
548	1190	660	152447	NA	287	7417
386	664	652	123587	NA	242	8466
1246	1238	1450	NA	NA	918	NA
735	868	1296	206959	NA	781	10648
849	1247	608	174632	NA	330	11636
622	1054	551	217390	NA	334	11369
886	1182	626	126544	NA	412	7508
1045	977	1163	164794	NA	593	7706
3165	2982	1643	306243	NA	1267	15809
1376	1498	632	243955	NA	546	11632
1076	954	583	204711	NA	379	13626
1248	1136	609	158398	NA	567	8773
773	570	551	70040	NA	450	6468
615	580	346	58031	NA	418	5529
991	1155	634	104281	NA	858	10683

564	913	476	77550	NA	632	7071
585	509	493	66529	NA	512	7234
1337	2143	869	123405	NA	1179	13310
24	1624	506	10	NA	20	15
543	845	487	63365	NA	575	6236
803	903	483	86358	NA	524	11588
740	1292	499	119509	NA	657	11650
1008	992	799	120799	NA	955	15652
1043	1356	750	146788	NA	1063	15119
758	1542	756	72244	NA	425	11130
1393	3437	906	142070	NA	448	21851
409	737	366	49013	NA	216	5806
757	1204	589	102680	NA	374	11704
1141	542	849	103068	NA	353	14569
519	223	489	44006	NA	134	5870
505	596	693	75096	NA	526	8304
500	337	812	56920	NA	519	7028
487	326	419	41421	NA	112	5860
352	245	374	42263	NA	88	8021
249	174	301	26449	NA	123	3542
719	721	1100	110449	NA	788	11870
440	287	779	60168	NA	343	8117
358	498	441	66776	NA	246	7154
454	475	482	63585	NA	222	7109
501	963	790	245929	NA	373	9562
791	869	715	87858	NA	449	7967
982	2043	856	105277	NA	703	8242
372	441	555	37221	NA	236	3309
246	212	425	31356	NA	68	2924
291	204	461	44536	NA	119	4037
328	371	648	41636	NA	611	3885
382	269	408	54460	NA	426	7011
574	703	493	58626	NA	484	6716
792	634	553	102719	NA	630	8741
440	387	355	70309	NA	518	8065
407	396	633	57084	NA	567	6229
739	674	705	94165	NA	679	11595
138	77	219	75099	NA	47	1193
293	159	253	33135	NA	162	4928
312	182	318	38343	NA	204	6132
1126	924	763	183376	NA	912	11207
9	12	58	2107	NA	9	92
773	648	652	157323	NA	358	10785
1059	1095	676	142763	NA	389	10217
446	351	271	59710	NA	74	2975
313	217	240	43151	NA	64	2920
779	632	1082	141863	NA	410	7753
999	964	1180	145851	NA	770	9890
532	441	421	67669	NA	193	7167
528	716	402	83250	NA	112	5661
537	744	363	81312	NA	200	5392
1198	655	1187	152110	NA	710	11022
1192	715	958	132382	NA	528	6134

1343	966	587	174294	NA	359	10003
907	590	517	142443	NA	245	6232
1689	1022	591	222522	NA	606	16760
5147	2723	1063	429536	NA	1067	12109
1277	976	648	122929	NA	740	5330
1983	867	681	111196	NA	414	3531
1726	1431	657	197785	NA	462	8698
2251	1897	817	219523	NA	580	10640
337	329	490	49722	NA	240	2597
278	161	329	28621	NA	196	1517
235	187	309	37975	NA	171	2597
130	113	252	24796	NA	12	1198
298	145	258	41422	NA	192	2636
283	382	389	51267	NA	340	5189
141	349	400	56958	NA	392	4436
482	459	322	63959	NA	290	4994
482	589	409	81777	NA	356	5671
624	770	422	112020	NA	570	7995
572	866	777	132987	NA	899	9010
508	567	720	98381	NA	616	6975
657	482	454	81207	NA	353	6290
931	284	295	30485	NA	77	4159
1927	325	600	38968	NA	177	4306
2053	162	555	31166	NA	39	4297
597	652	736	94770	NA	776	12178
402	760	463	76858	NA	205	6656
419	995	576	73975	NA	161	6905
607	1058	589	100683	NA	373	7217
733	706	532	119039	NA	666	7815
952	1071	712	142227	NA	688	11256
701	943	676	130933	NA	441	8662
617	883	589	107171	NA	239	8769
800	895	667	209332	NA	457	11449
612	349	932	95869	NA	430	5582
671	646	1141	184626	NA	667	9342
672	654	506	100596	NA	361	7829
378	508	481	126391	NA	243	6374
614	819	457	84671	NA	194	9773
784	1297	1087	225204	NA	610	6910
624	971	976	123609	NA	449	5723
342	294	478	62755	NA	250	5200
397	522	508	108236	NA	229	4889
470	512	475	62192	NA	282	5268
169	409	401	84327	NA	181	5263
423	525	562	93156	NA	390	6541
311	182	370	67541	NA	238	4974
585	726	648	123888	NA	463	7427
832	811	654	111245	NA	726	9420
388	418	592	89603	NA	418	5119
876	831	781	210076	NA	667	12401
753	482	614	101168	NA	176	8210
2191	259	514	64321	NA	119	4555
2136	272	434	67154	NA	149	5186

	328	483	405	94678	NA	106	4400
	451	394	433	69013	NA	146	5067
	18	1940	9	44	NA	3	71
	862	650	560	137870	NA	745	7171
	757	842	745	187587	NA	630	9833
NA		36	154	2000	NA	13	80
	466	2259	725	31207	NA	421	7564
	697	703	695	139214	NA	174	11128
	575	379	1037	122280	NA	530	5408
	505	571	1048	128627	NA	526	9091
	670	542	447	123244	NA	226	6888
	536	695	517	123766	NA	293	7031
	398	381	602	102844	NA	166	5774
	1289	1363	747	288196	NA	922	8762
	573	442	625	131089	NA	423	3565
	605	426	483	120545	NA	290	4756
	528	454	572	144528	NA	321	5298
	414	585	624	149282	NA	323	5474
	735	2398	81	404939	NA	13493	4982
	440	2835	89	352096	NA	8514	4386
	5	32	54	974	NA	2	39
	32	74	48	531	NA	27	55

Sucrose	Sulfuric acid	Threonate	Threonine	Trehalose	Tryptophan	Tyrosine	Uracil	Urea	Valine
2050	NA	5462	32596	NA	54	40	152	3333	22163
3750	NA	2620	16886	NA	40	46	17	1824	14425
5765	NA	3516	42512	NA	225	72	21	1728	35502
5947	NA	3368	36935	NA	147	73	58	1105	30316
10666	NA	3296	60835	NA	234	78	42	1601	50442
18203	NA	2581	51473	NA	70	38	159	1626	24731
16878	NA	3609	54609	NA	87	56	68	1479	30983
26301	NA	5321	149632	NA	408	100	99	2724	97801
9040	NA	2770	71995	NA	129	63	101	1896	45278
8720	NA	2038	48983	NA	303	55	46	1133	43325
20909	NA	5677	26238	NA	232	117	81	1182	26937
23957	NA	6335	69144	NA	208	84	75	1206	44509
13581	NA	4136	71132	NA	376	109	35	1355	33924
7417	NA	3538	41528	NA	379	89	54	1468	41135
19	NA	4	17	NA	14	20	11	51	0
13114	NA	14154	94954	NA	416	126	70	3881	32706
20092	NA	13867	67355	NA	140	72	97	3265	29456
9539	NA	8667	47280	NA	529	55	66	1867	45875
7614	NA	12926	61355	NA	1299	130	68	3570	65169
9681	NA	11385	65172	NA	1265	196	70	6497	73488
15570	NA	5855	66373	NA	297	126	78	2016	35138
15911	NA	10666	56667	NA	225	60	46	2300	30910
9143	NA	16544	53222	NA	618	113	90	2942	51777
8037	NA	10670	54058	NA	519	148	64	3574	33612
6509	NA	20173	51088	NA	547	121	67	4978	30832
18366	NA	8929	105190	NA	1297	155	124	2559	54912
12298	NA	7765	97967	NA	495	97	90	2513	40632
22752	NA	7753	113753	NA	590	94	146	4042	69525
27551	NA	6821	110102	NA	1618	200	162	4411	78308
17580	NA	9197	123837	NA	1809	162	123	2709	81897
13069	NA	11753	90606	NA	733	199	105	3836	56670
13869	NA	8689	74165	NA	519	125	104	2533	43772
13717	NA	6066	70334	NA	1112	155	98	2839	83600
11130	NA	5846	51801	NA	680	148	80	2355	62642
20925	NA	3625	75158	NA	1034	161	61	2288	76926
10016	NA	2973	41571	NA	143	89	87	1866	24514
10997	NA	2517	40647	NA	130	64	35	1842	23363
11089	NA	3308	58456	NA	335	63	85	1649	33706
14066	NA	4685	52809	NA	415	123	109	1998	32161
12574	NA	5573	72984	NA	446	122	139	3126	40007
13758	NA	3873	56485	NA	241	153	29	1221	38645
17369	NA	3513	63273	NA	408	164	51	1762	40016
15895	NA	8243	140578	NA	475	222	227	8442	106786
10893	NA	6736	96669	NA	385	158	86	3303	86695
4556	NA	2920	60466	NA	86	59	64	2308	51457
14333	NA	14546	54308	NA	354	142	77	2492	36901
21286	NA	17729	91984	NA	495	159	104	2524	55300
17516	NA	20579	103048	NA	2284	264	91	7135	125989
10083	NA	11790	61083	NA	818	173	121	4187	64416
13320	NA	12027	89554	NA	1406	187	161	2118	94538
22141	NA	14548	87256	NA	502	153	48	1926	47374

29959	NA	21750	100233	NA	1388	292	129	3486	68636
28981	NA	19031	110601	NA	2004	363	146	3220	67089
21051	NA	6240	60438	NA	1220	321	118	4117	63029
28242	NA	11939	95921	NA	1991	440	40	3794	122873
52	NA	70	533	NA	4	8	18	981	558
38033	NA	13821	95136	NA	1789	303	102	3076	52890
24498	NA	11091	139840	NA	1945	305	149	5606	100876
28845	NA	9903	91991	NA	3531	718	209	5677	117748
36925	NA	15626	102998	NA	5579	826	219	5844	142781
34306	NA	5647	84779	NA	1868	325	109	3953	71105
43450	NA	9948	126674	NA	2454	368	132	3497	86121
46032	NA	13374	77734	NA	2520	237	94	3397	107635
26501	NA	7730	78601	NA	2236	256	120	5281	89838
43225	NA	9511	57284	NA	3997	430	77	4568	108972
8074	NA	1874	38628	NA	54	56	23	1428	22912
8410	NA	2694	48658	NA	57	63	45	1186	23093
11927	NA	4623	70519	NA	426	148	9	2764	58655
9814	NA	3471	66557	NA	511	99	20	1401	54338
12930	NA	3774	88908	NA	485	107	56	3598	52978
17566	NA	2421	60931	NA	202	93	62	1422	23153
17079	NA	5008	67160	NA	186	156	34	2132	25620
20942	NA	3340	59606	NA	365	97	36	1118	26340
15723	NA	3412	69659	NA	403	68	102	3248	56180
17235	NA	5044	81542	NA	498	100	94	3041	56630
30402	NA	6735	109250	NA	168	60	118	1119	38193
39267	NA	4963	176536	NA	235	108	81	1400	45833
24604	NA	4216	118287	NA	555	112	83	1672	39046
16203	NA	4103	85963	NA	864	87	60	12003	37024
13252	NA	3231	79983	NA	574	82	21	8703	32225
2469	NA	1074	15660	NA	120	32	60	2362	10344
19148	NA	7278	70814	NA	191	57	37	2016	35479
19277	NA	19460	270457	NA	212	202	259	7519	148612
3458	NA	1508	25920	NA	77	65	42	826	22457
3366	NA	2430	63239	NA	187	27	39	1916	25501
2610	NA	811	20330	NA	96	45	65	1002	9489
15952	NA	4272	72240	NA	175	89	66	1400	35796
16057	NA	6114	128984	NA	602	94	130	2220	60536
14118	NA	6542	117870	NA	506	106	85	3676	46052
18531	NA	6967	149642	NA	642	128	139	3465	69520
34050	NA	8401	153998	NA	862	181	145	3275	64279
27792	NA	8023	140319	NA	413	105	122	2403	52324
25133	NA	7344	126592	NA	666	103	114	4004	72660
31307	NA	6428	162393	NA	626	137	123	4453	70354
33798	NA	8583	106347	NA	710	185	115	2114	92677
25463	NA	5467	112962	NA	479	185	71	3266	59341
28222	NA	5365	121413	NA	618	231	135	3279	76346
16150	NA	4598	64517	NA	274	129	110	10062	66972
16825	NA	2600	77543	NA	574	136	75	5837	65371
9994	NA	2636	49675	NA	428	81	95	5043	45375
12723	NA	3295	31008	NA	88	55	46	1786	20428
20957	NA	4208	42022	NA	166	88	26	2248	24041
20605	NA	3037	68475	NA	312	121	40	1203	30009
20545	NA	2962	73545	NA	550	102	36	2389	64334

27486	NA	3630	100030	NA	678	171	47	1326	59134
13734	NA	8910	88524	NA	175	66	15	1207	30872
21565	NA	8533	86077	NA	146	73	61	1645	32087
12108	NA	5290	77536	NA	505	41	94	3434	51071
17699	NA	4509	88216	NA	641	109	25	1763	65492
9499	NA	6489	60930	NA	596	45	39	2912	41475
21092	NA	7775	125409	NA	338	96	128	3081	54564
15738	NA	3980	73100	NA	307	80	75	1047	33935
3311	NA	2591	37143	NA	181	94	17	1290	26939
11882	NA	9656	87826	NA	590	104	58	1853	34754
13336	NA	12460	120827	NA	980	179	64	3567	57957
11832	NA	2348	66114	NA	497	132	51	1108	32645
23431	NA	3834	107021	NA	793	164	115	3411	47253
15791	NA	6222	129975	NA	781	158	95	3963	72322
20549	NA	7155	187585	NA	1166	239	135	3629	99190
10906	NA	3645	125744	NA	803	131	49	2986	64772
41303	NA	8806	159003	NA	1351	208	67	3466	69537
31519	NA	11507	176979	NA	1269	234	77	3224	74602
33002	NA	8285	164045	NA	2220	288	40	2570	70252
31752	NA	6911	125716	NA	1362	170	118	2097	52352
37615	NA	5671	125052	NA	4015	198	46	3633	69972
35328	NA	2852	149934	NA	2600	334	105	2242	106373
31871	NA	8369	154126	NA	3935	543	58	3476	121096
63978	NA	5443	160569	NA	9105	561	30	4627	145468
37740	NA	3806	137413	NA	3532	443	94	4274	133319
25901	NA	4491	154067	NA	2592	345	90	6093	124122
7005	NA	4938	27740	NA	74	33	102	1186	23531
4126	NA	1847	25038	NA	91	15	43	1933	23234
3399	NA	3370	29017	NA	186	79	12	2738	26445
5805	NA	2961	23746	NA	157	67	23	2021	24285
7758	NA	4988	33339	NA	383	107	46	2651	27235
14529	NA	3821	75825	NA	321	151	44	1443	49440
10356	NA	3538	53195	NA	159	129	24	1476	24175
811	NA	670	6247	NA	49	40	18	1227	8232
8750	NA	2879	59442	NA	325	145	53	1448	36395
16205	NA	3770	65908	NA	321	117	57	1689	29051
8562	NA	1975	39263	NA	102	74	76	887	24551
6764	NA	2542	31737	NA	106	46	108	1016	19521
3028	NA	969	29041	NA	128	28	46	1044	21699
12032	NA	3270	48353	NA	223	32	70	658	33867
9362	NA	2498	44160	NA	317	89	95	793	15516
12926	NA	17953	70352	NA	275	105	70	1893	36247
17533	NA	9953	69024	NA	281	108	94	2387	43523
10653	NA	7072	45014	NA	323	88	126	3404	55644
17320	NA	9133	69577	NA	472	119	142	3868	47775
13579	NA	8451	59362	NA	585	222	102	3680	68000
9792	NA	3493	47897	NA	168	84	54	2511	27830
11295	NA	2422	45216	NA	154	43	28	980	27167
21406	NA	11280	71031	NA	523	238	81	3087	48102
12993	NA	22048	132169	NA	586	119	216	6598	95750
16994	NA	14791	88007	NA	508	176	146	10639	70949
13965	NA	6371	80839	NA	683	253	121	3165	49224
14256	NA	5416	71732	NA	371	177	184	5540	45523

17992	NA	5892	97734	NA	1206	269	285	5249	93219
12546	NA	3649	83614	NA	633	197	228	2830	77338
3034	NA	1093	23951	NA	314	83	64	1895	25600
19028	NA	4791	96069	NA	557	222	187	3374	73980
17352	NA	3811	80385	NA	342	201	209	2563	60638
17325	NA	4013	86411	NA	626	154	152	6594	82782
20871	NA	4669	78509	NA	860	235	133	5851	103917
14889	NA	4236	69503	NA	551	203	186	6237	76165
10999	NA	2073	45799	NA	75	51	69	2246	17198
12617	NA	3416	40051	NA	93	127	127	1352	23923
11665	NA	3085	63768	NA	447	171	89	942	62540
13297	NA	3170	61829	NA	204	187	101	1043	45167
10949	NA	3464	56730	NA	403	162	77	1589	31600
10074	NA	2059	34902	NA	160	127	64	1205	29003
13022	NA	8986	48393	NA	365	218	96	2170	33628
4740	NA	1492	28871	NA	287	90	114	1025	30891
6987	NA	1893	42396	NA	136	52	77	1557	51201
10298	NA	3546	56295	NA	379	188	79	1793	35403
19976	NA	14579	95108	NA	311	188	238	4733	51937
16137	NA	8289	62660	NA	202	168	117	3993	45894
10506	NA	5363	48275	NA	565	159	116	7613	32424
16	NA	5	9	NA	44	7	10	23	0
24096	NA	6462	45676	NA	386	352	112	5426	34304
22378	NA	3567	73555	NA	611	221	141	2251	46675
23418	NA	4266	67988	NA	451	158	81	1647	38434
17991	NA	14801	119905	NA	773	374	274	6374	95095
20249	NA	15822	104855	NA	582	284	202	4760	79270
12348	NA	11890	99831	NA	668	243	159	6317	73287
17395	NA	7855	92341	NA	920	194	128	2759	52693
24934	NA	8270	103595	NA	742	279	148	2943	49754
21457	NA	7590	124794	NA	1274	392	147	4034	101957
21812	NA	5547	111132	NA	1146	266	159	4597	99933
16564	NA	2973	37690	NA	586	141	73	1686	48655
30680	NA	5043	130240	NA	1516	466	109	4041	97635
30771	NA	11347	149880	NA	1595	508	111	5445	108108
23078	NA	6173	103339	NA	1110	332	107	4284	121937
22846	NA	4242	92564	NA	2069	501	102	4814	92599
23200	NA	3955	98996	NA	1509	535	157	5670	133553
427907	74	3193	118863	373	186	4792	295	7189	80699
165646	32	848	31169	186	75	1790	240	5009	37413
147132	46	6034	58337	58	171	2691	201	4917	53628
134471	16	2500	51357	158	91	2593	225	8179	49521
123230	5	1489	36526	125	16	2626	241	5903	43084
294708	29	994	44641	13	7	1482	256	3523	37303
199905	26	618	30067	44	5	1264	134	1482	22347
643848	112	5359	151526	334	135	6128	468	8141	95701
598705	126	3656	151814	376	72	6493	398	12241	94478
441792	193	4011	110638	239	100	5445	461	9569	80956
16	26	6	24	124	118	31	28	95	40
628525	63	2106	98917	236	98	3877	2093	4974	77665
707534	95	2428	120840	286	301	4762	896	5583	83048
713571	246	3760	149022	429	322	8321	904	5352	111531
650361	136	3032	130601	292	170	6330	931	7557	95541

455106	106	1736	88303	43	104	5731	804	15121	74237
74	20	11	18	3	8	36	78	61	32
460517	56	1357	76569	466	84	2969	323	2678	58798
270259	50	1081	60148	264	25	1990	247	1819	46941
88194	24	316	15310	56	26	639	129	3582	16199
447739	47	1578	63010	186	101	5319	805	15346	63615
522603	78	2189	105467	372	150	6096	746	31268	76507
310205	31	1814	59325	282	56	2562	353	3633	60212
285687	28	1029	45075	394	42	1734	265	2330	45796
269881	63	1078	36305	363	59	1487	356	2542	38138
452801	107	2078	52500	349	216	3105	684	8285	45855
474371	NA	2723	79203	447	297	4718	924	10471	70796
409038	54	2581	81052	688	96	4193	661	4801	66625
469970	48	2355	59308	782	110	4169	774	14495	55460
347607	28	1765	50237	380	87	2931	494	2747	52177
497961	97	1745	79511	383	195	5787	882	7145	70718
121046	23	588	27645	115	46	1420	385	3380	31625
426178	25	1598	69661	602	176	4204	592	4052	71618
272465	15	1305	57401	377	135	3616	457	4330	76130
454995	17	1233	62449	575	182	4018	619	4684	64168
7555	20	90	1921	6	55	136	26	114	1253
15922	28	55	990	29	25	21	11	52	652
320734	289	2887	92258	165	196	6734	542	5358	94400
375254	160	5991	142753	412	202	9870	673	10343	115532
223540	138	2053	61065	175	66	4182	371	6693	NA
373967	127	2306	95298	161	124	7601	1284	10049	105108
309731	77	1914	81784	168	128	6808	924	8521	88375
316756	56	3309	102256	241	106	6652	704	7892	95800
439891	57	4560	151129	363	136	8919	800	8663	116669
413443	161	3769	146248	394	157	9826	769	9570	116203
572483	74	3144	199490	74	127	9027	3325	4201	132328
585077	2130	3227	139572	352	255	11275	1803	NA	NA
360068	54	2311	117155	471	141	5964	280	2140	88862
468730	19	2062	120923	728	251	6738	269	1654	106780
434554	60	2729	173437	671	149	5505	266	2557	104100
612336	68	2341	162007	285	255	15801	718	9478	163530
728030	88	1747	206193	71	392	33248	791	10969	230540
502157	42	1282	125956	705	138	5336	464	4417	90852
443628	96	1208	117234	650	194	7718	407	3895	113049
545532	24	1030	77233	895	193	6281	272	2745	90956
654057	2690	1002	NA	633	117	4968	742	7559	22487
694206	35	2419	173709	245	306	11691	658	5143	128889
619747	66	2553	123220	1239	260	10168	404	2333	147908
466635	35	2139	136083	858	200	7716	510	2174	114971
590522	101	1793	81157	1053	263	9472	396	1490	129658
1167157	32	1246	89071	726	216	8171	699	10139	95264
2009783	240	2994	274620	2503	674	23109	957	9276	212094
971809	147	1603	202477	2102	533	18666	500	3144	189685
724039	84	2120	152852	1145	459	13759	525	3622	177231
859913	45	1706	104850	2117	315	8946	466	2725	116402
565511	60	2237	84714	486	225	2953	328	6563	51272
467407	129	1343	68969	409	156	2689	281	4958	41117
644390	97	4914	123180	588	192	5818	347	9591	82741

431417	122	2058	67389	477	273	4289	374	10072	57938
379136	96	2405	66679	623	124	4415	376	11388	55912
609593	135	6164	182046	603	377	10377	880	9910	131380
134	5	5	13	14	194	9	37	12	NA
375375	79	2071	60951	579	149	5180	569	9543	59704
454721	183	2710	84122	334	153	8199	612	13107	85373
535191	167	3726	109129	466	122	10396	687	13750	94847
919139	104	1937	132383	896	149	5579	783	10313	71642
1018678	184	2314	154812	812	231	10789	835	12252	135034
628741	17	2008	111430	11853	111	3772	237	4625	NA
859601	17	3004	191159	3576	151	7059	401	7861	NA
474586	140	859	56680	695	77	2583	170	2954	48271
564277	39	1976	101141	1246	181	5435	1488	7663	95129
690896	47	2791	93076	1608	208	7779	1029	11384	105047
363179	56	1746	37311	618	27	1934	499	4217	41521
395344	50	1633	69107	150	170	5645	570	13383	64738
380420	62	1358	58466	120	89	4124	562	7781	55393
320032	99	1544	60445	844	123	3115	270	3059	54883
323409	51	1704	43332	673	73	2613	275	4007	46728
248050	55	730	25356	435	110	1716	359	1972	34696
718818	133	2548	71371	629	194	7329	867	13962	74079
368899	19	1210	35460	123	117	3245	750	10572	39197
380472	29	1457	46892	464	82	3114	504	6924	47649
374640	57	1322	44573	383	154	3141	611	6730	46603
437118	21	3386	74247	497	173	20087	893	65266	96928
537454	218	2348	96247	719	294	9559	776	7066	90720
646762	72	4136	118800	1067	361	15808	1024	8296	131891
332527	21	1954	50930	490	76	3009	439	3035	58876
202247	41	1055	28672	225	100	2618	426	4118	46577
287537	17	1624	42621	285	60	4794	457	5027	66399
245407	74	1090	37215	57	42	2692	1960	5758	55180
350059	125	1780	61031	57	70	3395	943	7405	56501
444757	89	2986	66539	922	298	5631	495	10473	69051
645668	168	2500	91763	497	193	7666	629	18008	98018
465958	89	2045	68012	447	137	6310	634	12124	84002
347876	81	1028	45630	447	32	2397	501	5508	35725
584344	69	2184	83958	1103	89	4926	723	7654	61900
96227	15	425	16488	11	85	3320	84	2524	30391
297478	15	885	26094	399	58	1467	100	1904	25076
362893	36	1041	31509	322	88	1826	139	2183	29993
699040	91	1467	140183	588	209	8671	1045	10645	118581
359	40	18	222	117	10	140	439	5930	772
683738	112	1738	95949	463	376	11725	813	9091	122942
615128	119	1665	89799	600	207	9448	678	4361	120912
338837	20	663	41334	323	126	1898	256	2427	40619
248954	27	727	30973	97	20	3503	144	1634	31858
511376	78	1615	97261	79	212	14099	603	11889	128115
561030	144	2051	152274	1035	355	13407	665	9192	131240
358760	59	1193	86322	1027	115	5653	369	2637	84602
247474	3	1519	66567	449	89	5190	421	2321	80141
243313	34	1628	84923	642	167	6307	347	2483	89078
1175331	89	1378	105233	253	315	7148	709	7495	90010
956981	91	1097	107262	586	242	11661	738	8615	122867

617079	42	1783	167832	1155	358	8107	544	5394	114135
633239	39	1528	115045	1145	225	8599	441	8637	105692
800852	42	3487	172909	1985	477	13984	669	6949	155430
1998387	342	3724	380464	2545	906	33513	1171	10694	305694
787191	110	1255	125626	968	346	13585	813	6140	153211
1152175	115	1232	138972	2411	293	6384	414	3495	129663
1073722	97	1809	161875	2296	433	16997	651	4735	202012
1133947	98	2359	184128	2657	538	20828	727	5517	230509
256491	24	1359	37754	27	26	1272	190	4724	33953
224172	36	1137	20953	253	38	784	114	3108	20359
159784	100	875	31580	170	21	1454	166	2681	32615
666101	12	1024	21007	88	26	655	72	3231	22226
457398	50	1615	36729	156	21	1474	187	3759	39032
173869	NA	1020	57051	86	87	1739	346	2792	29575
192374	33	1074	53817	191	54	1489	321	3628	39192
255881	17	1562	56298	288	36	2198	313	4933	41617
355996	107	1523	67284	310	71	2930	415	7148	49306
459322	91	2196	95056	346	125	3710	513	10146	62719
423405	54	3145	111714	442	146	6283	855	8817	93990
314471	42	2516	96981	338	62	3632	743	5180	62476
289938	61	2278	68202	333	37	3214	602	5690	50614
245119	59	925	37325	82571	NA	1016	175	1233	NA
299184	14	1102	56454	150954	58	1517	60	1957	NA
279855	14	1005	31810	179160	63	1446	96	2974	NA
219587	73	19794	102333	4275	517	5333	1142	6740	83856
186810	34	1351	101878	517	119	3980	277	1616	76307
173322	56	1703	114080	515	77	3781	291	2863	69531
370833	20	2759	162130	719	87	5835	351	2649	100086
416505	44	1645	106247	87	177	7260	765	16982	93343
262519	61	2639	196508	163	165	7800	787	11203	127548
424603	46	1656	150163	1171	124	5254	553	4808	98880
281655	34	1708	138800	668	133	4484	511	6212	99122
364811	24	4548	196284	1010	266	14408	501	7035	169630
237864	66	1302	76096	68	152	6060	773	17213	74524
207802	112	2348	124960	101	193	10136	974	18471	113947
230220	16	2288	98252	638	94	5841	613	5048	92385
301041	62	1839	78696	429	156	7501	490	3929	100606
283901	43	1744	95262	700	130	5348	593	4225	95939
494577	45	1809	139115	738	310	13694	973	23367	142545
537754	163	1157	92016	694	237	9393	924	12486	114927
306905	21	1113	50022	522	174	3805	470	4704	60965
356259	28	1116	56942	767	121	5271	516	5117	72989
342002	17	1211	71202	556	75	3793	489	5587	68576
239365	88	1742	76131	130	89	2681	325	3840	68006
275775	118	2545	93243	36	62	3262	377	7431	79996
149836	74	1620	50560	203	152	2401	393	4548	52286
316296	70	3290	110373	430	77	5936	499	7801	101218
358466	89	3311	129010	594	144	7439	614	9338	104772
321215	52	1424	70826	159	60	6064	680	10804	NA
562085	136	3121	170121	453	247	13301	796	5956	148335
495209	85	2309	113875	12028	142	6336	123	1821	NA
375646	9	1375	72649	173887	69	3780	116	2271	NA
388703	40	1390	75136	144326	79	4047	120	2450	NA

239823	29	1404	73333	58	47	4422	234	3228	66860
375261	89	1094	83102	491	146	4728	262	2167	78680
10	11	12	29	35	62	38	12	43	47
427164	81	2075	143399	470	249	12336	688	14956	135252
312587	92	3309	155739	360	341	13636	745	11642	134126
1475	NA	9	242	116	31	33	117	2416	1082
326589	974	959	34532	507	106	1682	428	11791	NA
317208	73	2607	141995	923	164	9640	434	5018	126572
326184	39	1823	84845	370	125	9343	710	10153	94631
410979	57	2550	100113	99	181	10649	612	8408	103821
294939	71	2292	116339	771	153	8390	457	2695	113347
278202	30	2191	109466	736	100	6839	560	2868	107673
251158	30	1947	79591	322	137	6761	447	2646	95408
644466	186	2437	168941	936	447	15970	1096	16602	137094
429186	86	919	100349	56	145	9188	642	22264	106456
385230	13	1577	119746	430	159	9566	411	20300	108869
365981	74	1863	125642	478	200	11043	473	4163	131926
306963	43	1720	127611	437	98	9184	562	6350	120221
872205	20	4476	89155	295	NA	1433	644	4459	62184
629544	NA	4548	79763	371	19	878	499	2677	57302
818	10	18	132	134	15	26	253	920	314
333	34	9	122	71	29	5	202	398	287
