# Supplementary material for: Analysis of metabolic dynamics during drought stress in Arabidopsis plants
Source: Sci Data. 2022 Mar 21;9:90. doi: 10.1038/s41597-022-01161-4 (PMC8938536; doi:10.1038/s41597-022-01161-4)
Supplement: Supplementary file 4 — Supplementary Data 1 [file 41597_2022_1161_MOESM4_ESM.pdf]

Assay Class: Eukaryote Total RNA Nano
Data Path: \\C...Eukaryote Total RNA Nano_DE13701056_2017-08-30_12-37-58.xad
Created: 8/30/2017 12:37:57 PM
Modified: 8/30/2017 2:25:51 PM

Electropherogram Summary

W1

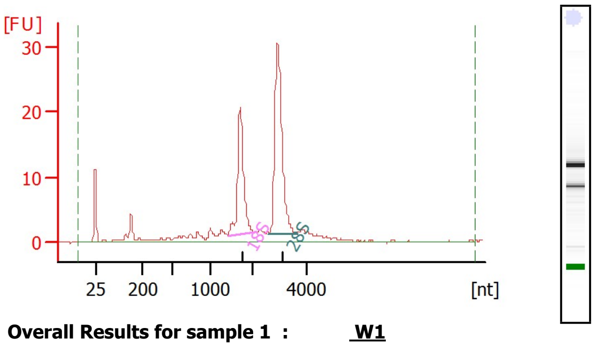

Overall Results for sample 1 : W1

RNA Area:	183.2
RNA Concentration:	85 ng/µl
rRNA Ratio [28s / 18s]:	1.6
RNA Integrity Number (RIN):	9.5 (B.02.08)
Result Flagging Color:	
Result Flagging Label:	RIN: 9.50

Fragment table for sample 1 : W1

Name	Start Size [nt]	End Size [nt]	Area	% of total Area
18S	1,431	2,181	42.5	23.2
28S	2,656	3,637	68.2	37.2

W2

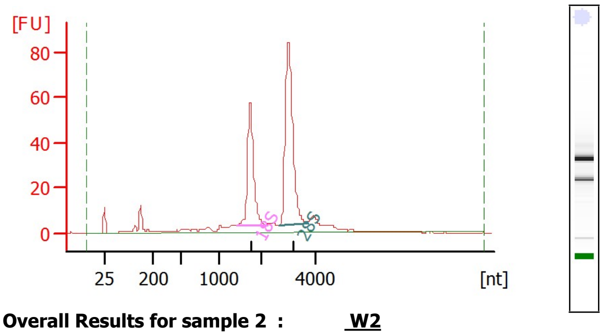

Overall Results for sample 2 : W2

RNA Area:	511.5
RNA Concentration:	238 ng/µl
rRNA Ratio [28s / 18s]:	1.6
RNA Integrity Number (RIN):	9.8 (B.02.08)
Result Flagging Color:	
Result Flagging Label:	RIN: 9.80

Fragment table for sample 2 : W2

Name	Start Size [nt]	End Size [nt]	Area	% of total Area
18S	1,424	2,197	119.7	23.4
28S	2,657	3,679	195.2	38.2

W3

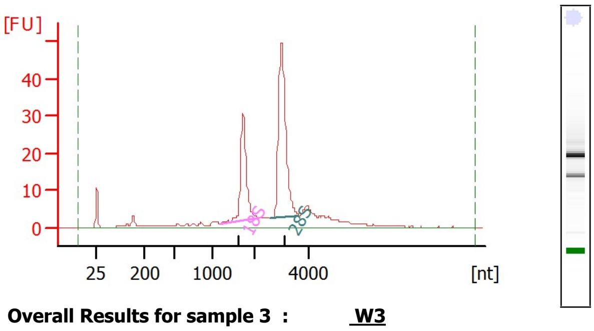

Overall Results for sample 3 : W3

RNA Area:	310.3
RNA Concentration:	144 ng/µl
rRNA Ratio [28s / 18s]:	1.8
RNA Integrity Number (RIN):	9.9 (B.02.08)
Result Flagging Color:	
Result Flagging Label:	RIN: 9.90

Fragment table for sample 3 : W3

Name	Start Size [nt]	End Size [nt]	Area	% of total Area
18S	1,201	2,171	61.4	19.8
28S	2,613	3,643	112.0	36.1

W4

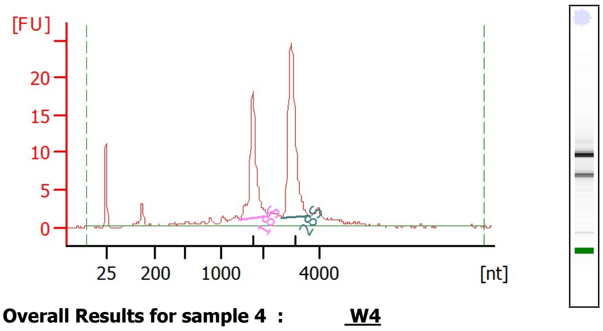

Overall Results for sample 4 : W4

RNA Area:	159.0
RNA Concentration:	74 ng/µl
rRNA Ratio [28s / 18s]:	1.5
RNA Integrity Number (RIN):	9.6 (B.02.08)
Result Flagging Color:	
Result Flagging Label:	RIN: 9.60

Fragment table for sample 4 : W4

Name	Start Size [nt]	End Size [nt]	Area	% of total Area
18S	1,424	2,158	37.5	23.6
28S	2,642	3,656	55.8	35.1

© Copyright 2003 - 2015 Agilent Technologies, Inc.

Assay Class: Eukaryote Total RNA Nano
Data Path: \\C...Eukaryote Total RNA Nano_DE13701056_2017-08-30_12-37-58.xad

Created: 8/30/2017 12:37:57 PM
Modified: 8/30/2017 2:25:51 PM

Electropherogram Summary Continued ...

W5

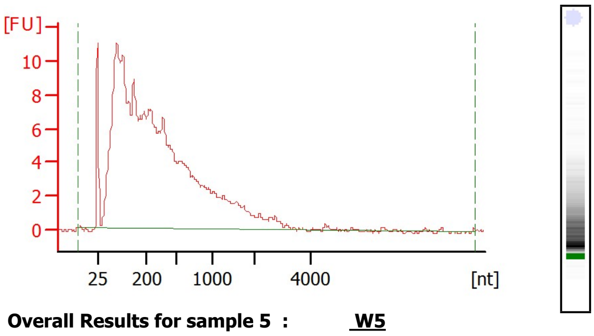

Overall Results for sample 5 : W5

RNA Area:	290.4
RNA Concentration:	135 ng/µl
rRNA Ratio [28s / 18s]:	0.0
RNA Integrity Number (RIN):	2.2 (B.02.08)
Result Flagging Color:	
Result Flagging Label:	RIN: 2.20

W6

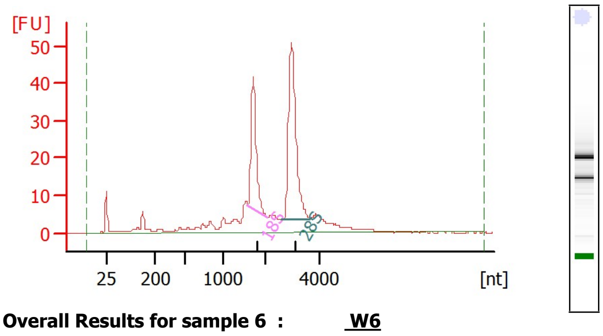

Overall Results for sample 6 : W6

RNA Area:	376.6
RNA Concentration:	175 ng/µl
rRNA Ratio [28s / 18s]:	1.8
RNA Integrity Number (RIN):	8.8 (B.02.08)
Result Flagging Color:	
Result Flagging Label:	RIN: 8.80

Fragment table for sample 6 : W6

Name	Start Size [nt]	End Size [nt]	Area	% of total Area
18S	1,588	2,056	64.8	17.2
28S	2,613	3,639	114.0	30.3

D1

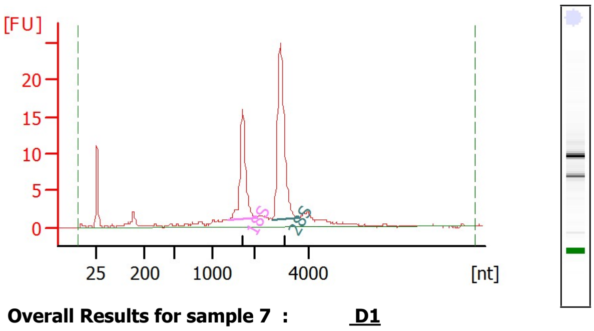

Overall Results for sample 7 : D1

RNA Area:	144.1
RNA Concentration:	67 ng/µl
rRNA Ratio [28s / 18s]:	1.7
RNA Integrity Number (RIN):	9.8 (B.02.08)
Result Flagging Color:	
Result Flagging Label:	RIN: 9.80

Fragment table for sample 7 : D1

Name	Start Size [nt]	End Size [nt]	Area	% of total Area
18S	1,383	2,068	30.5	21.2
28S	2,627	3,614	51.8	36.0

D2

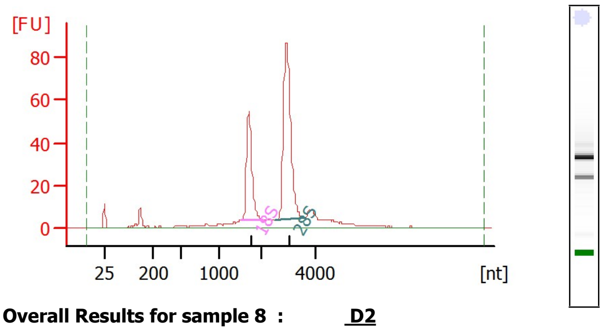

Overall Results for sample 8 : D2

RNA Area:	476.7
RNA Concentration:	222 ng/µl
rRNA Ratio [28s / 18s]:	1.9
RNA Integrity Number (RIN):	9.9 (B.02.08)
Result Flagging Color:	
Result Flagging Label:	RIN: 9.90

Fragment table for sample 8 : D2

Name	Start Size [nt]	End Size [nt]	Area	% of total Area
18S	1,501	2,064	101.7	21.3
28S	2,508	3,605	197.5	41.4

 © Copyright 2003 - 2015 Agilent Technologies, Inc.

Assay Class: Eukaryote Total RNA Nano
Data Path: \\C...Eukaryote Total RNA Nano_DE13701056_2017-08-30_12-37-58.xad
Created: 8/30/2017 12:37:57 PM
Modified: 8/30/2017 2:25:51 PM

Electropherogram Summary Continued ...

D3

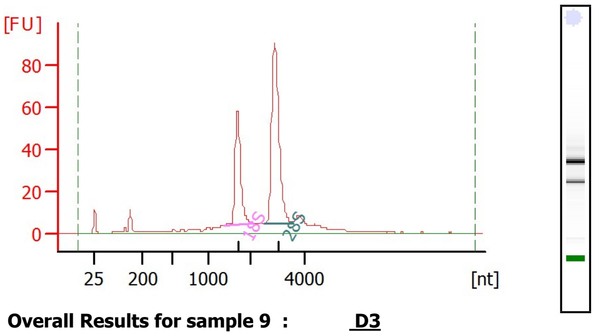

Overall Results for sample 9 : D3

RNA Area:	540.3
RNA Concentration:	251 ng/µl
rRNA Ratio [28s / 18s]:	1.8
RNA Integrity Number (RIN):	9.8 (B.02.08)
Result Flagging Color:	
Result Flagging Label:	RIN: 9.80

Fragment table for sample 9 : D3

Name	Start Size [nt]	End Size [nt]	Area	% of total Area
18S	1,407	2,104	114.4	21.2
28S	2,552	3,552	208.0	38.5

D4

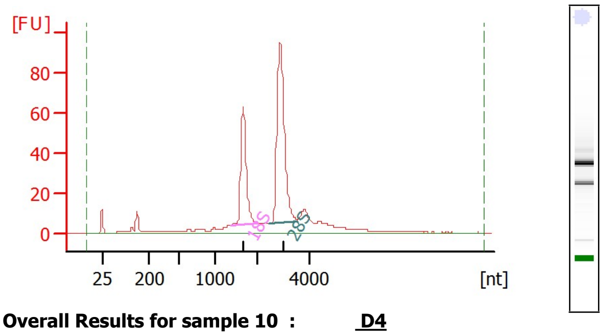

Overall Results for sample 10 : D4

RNA Area:	607.6
RNA Concentration:	283 ng/µl
rRNA Ratio [28s / 18s]:	1.8
RNA Integrity Number (RIN):	9.9 (B.02.08)
Result Flagging Color:	
Result Flagging Label:	RIN: 9.90

Fragment table for sample 10 : D4

Name	Start Size [nt]	End Size [nt]	Area	% of total Area
18S	1,380	2,055	123.3	20.3
28S	2,461	3,528	220.3	36.2

D5

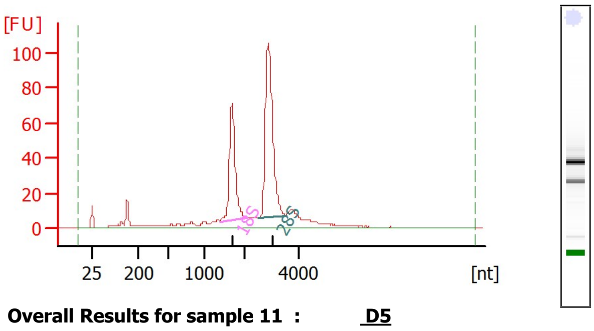

Overall Results for sample 11 : D5

RNA Area:	691.1
RNA Concentration:	321 ng/µl
rRNA Ratio [28s / 18s]:	1.7
RNA Integrity Number (RIN):	9.7 (B.02.08)
Result Flagging Color:	
Result Flagging Label:	RIN: 9.70

Fragment table for sample 11 : D5

Name	Start Size [nt]	End Size [nt]	Area	% of total Area
18S	1,373	2,050	142.8	20.7
28S	2,519	3,518	247.1	35.7

D6

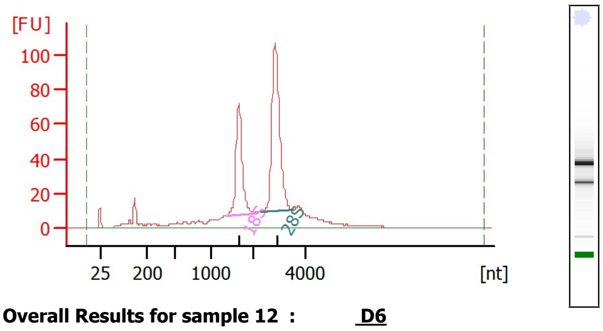

Overall Results for sample 12 : D6

RNA Area:	802.7
RNA Concentration:	373 ng/µl
rRNA Ratio [28s / 18s]:	1.8
RNA Integrity Number (RIN):	9.3 (B.02.08)
Result Flagging Color:	
Result Flagging Label:	RIN: 9.30

Fragment table for sample 12 : D6

Name	Start Size [nt]	End Size [nt]	Area	% of total Area
18S	1,358	2,017	145.4	18.1
28S	2,321	3,522	256.3	31.9

 © Copyright 2003 - 2015 Agilent Technologies, Inc.
