# Supplementary material for: Analysis of metabolic dynamics during drought stress in Arabidopsis plants
Source: Sci Data. 2022 Mar 21;9:90. doi: 10.1038/s41597-022-01161-4 (PMC8938536; doi:10.1038/s41597-022-01161-4)
Supplement: Supplementary file 5 — Supplementary Data 2 [file 41597_2022_1161_MOESM5_ESM.pdf]

FastQC Report

Tue 12 Sep 2017
22002_ATCACG_L001_R1.fastq.gz

Summary

- Basic Statistics
- Per base sequence quality
- Per tile sequence quality
- Per sequence quality scores
- Per base sequence content
- Per sequence GC content
- Per base N content
- Sequence Length Distribution
- Sequence Duplication Levels
- Overrepresented sequences
- Adapter Content
- Kmer Content

Basic Statistics

Measure	Value
Filename	22002_ATCACG_L001_R1_001.fastq.gz
File type	Conventional base calls
Encoding	Sanger / Illumina 1.9
Total Sequences	21422426
Sequences flagged as poor quality	0
Sequence length	50
%GC	46

Per base sequence quality

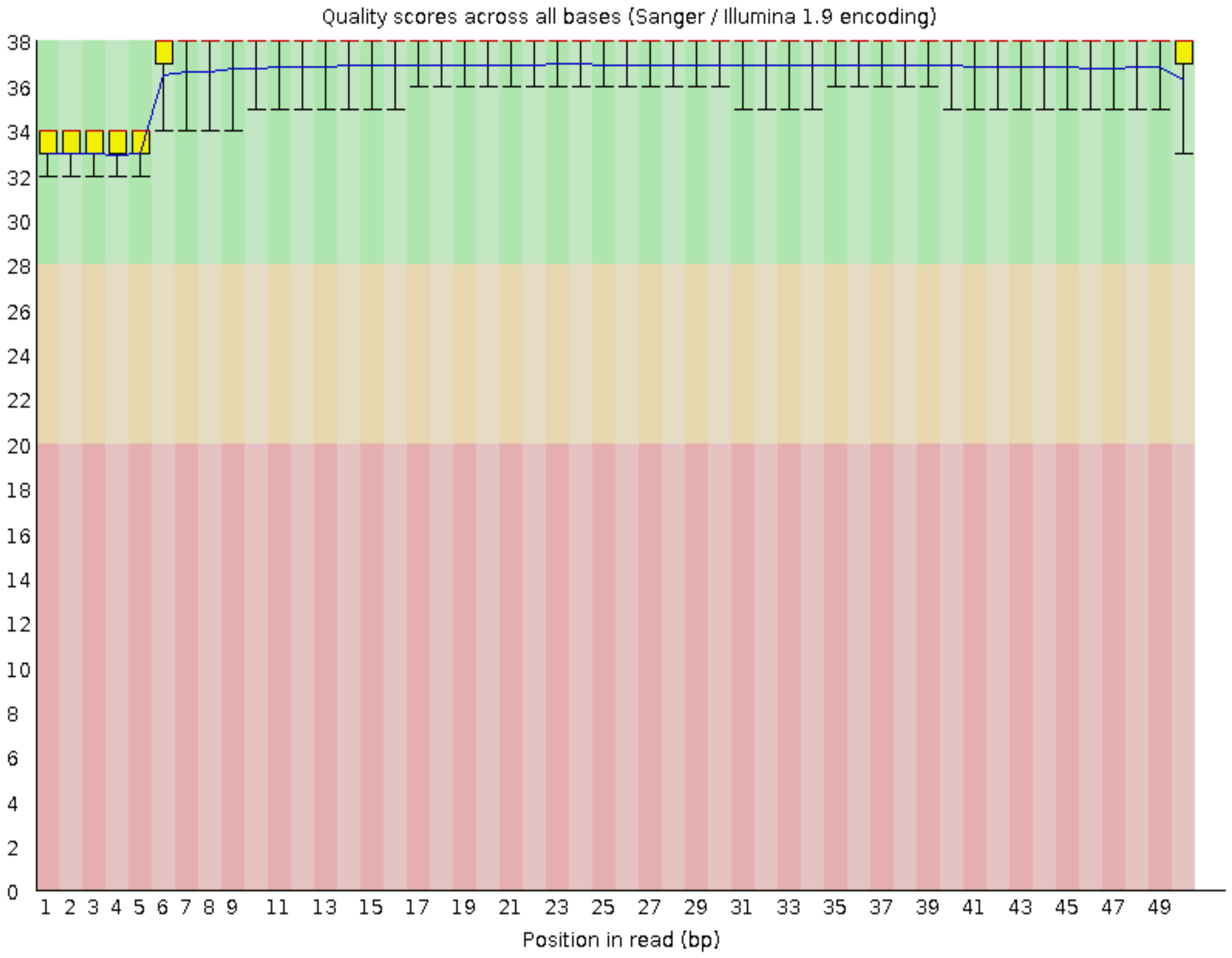

Per tile sequence quality

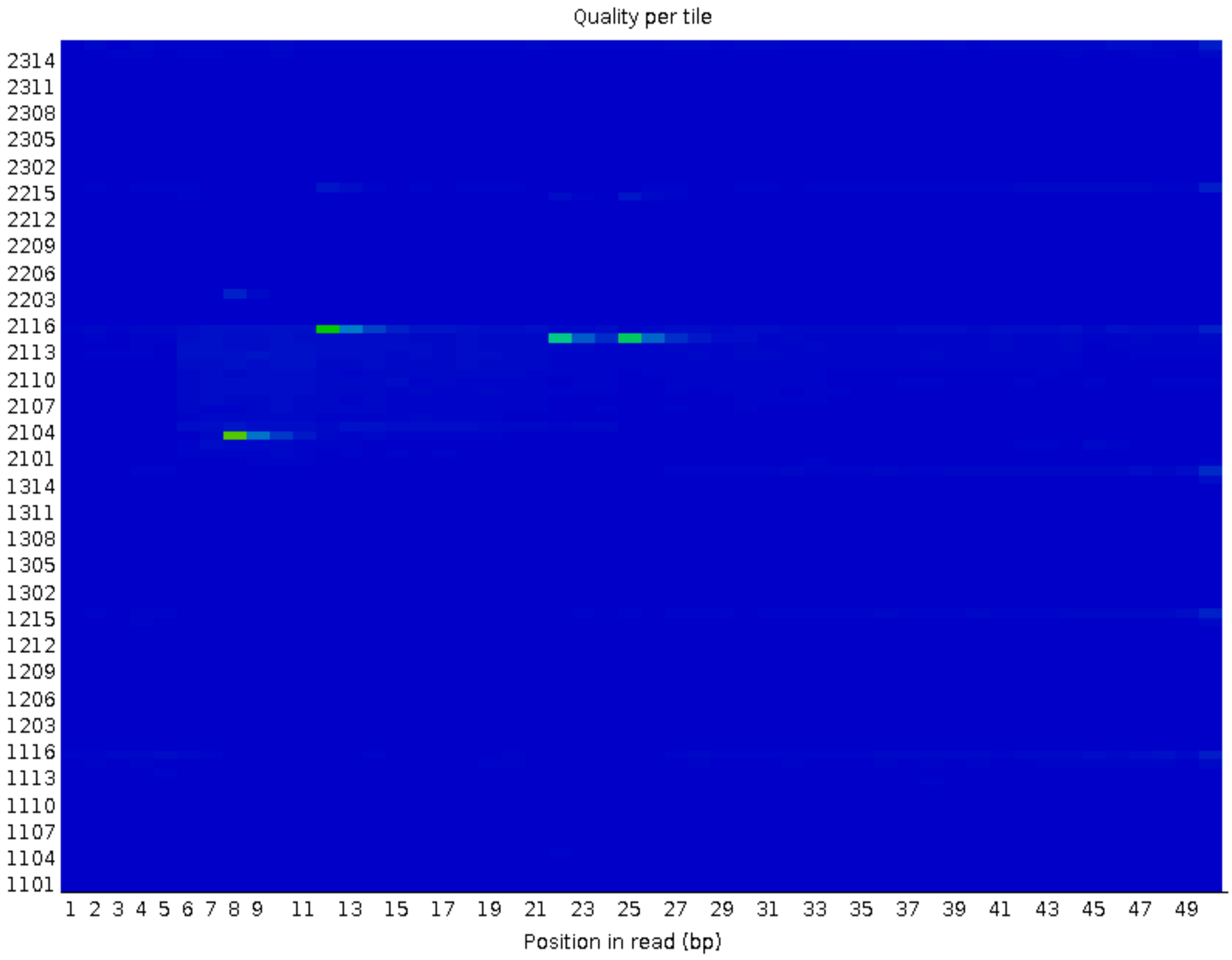

Per sequence quality scores

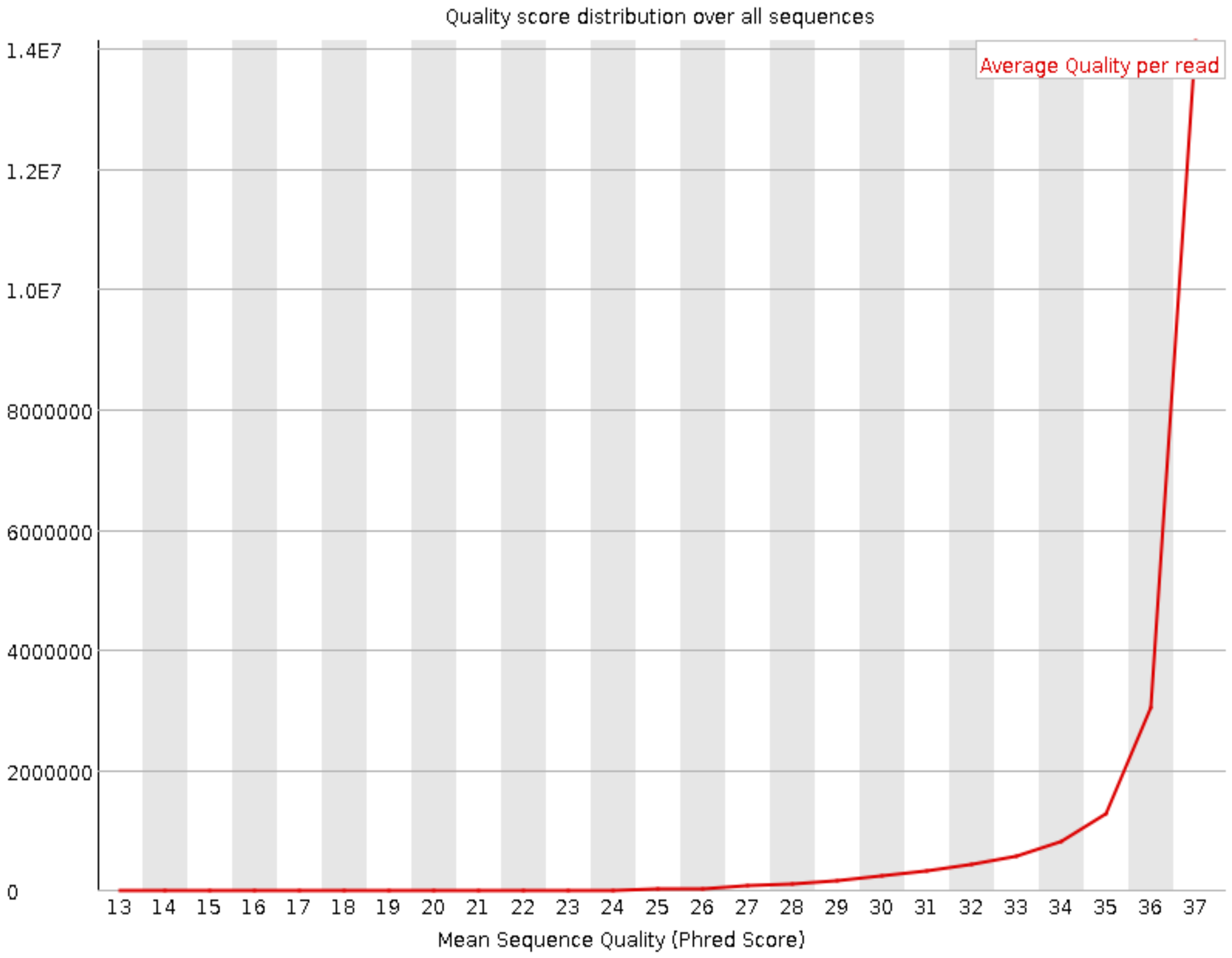

Per base sequence content

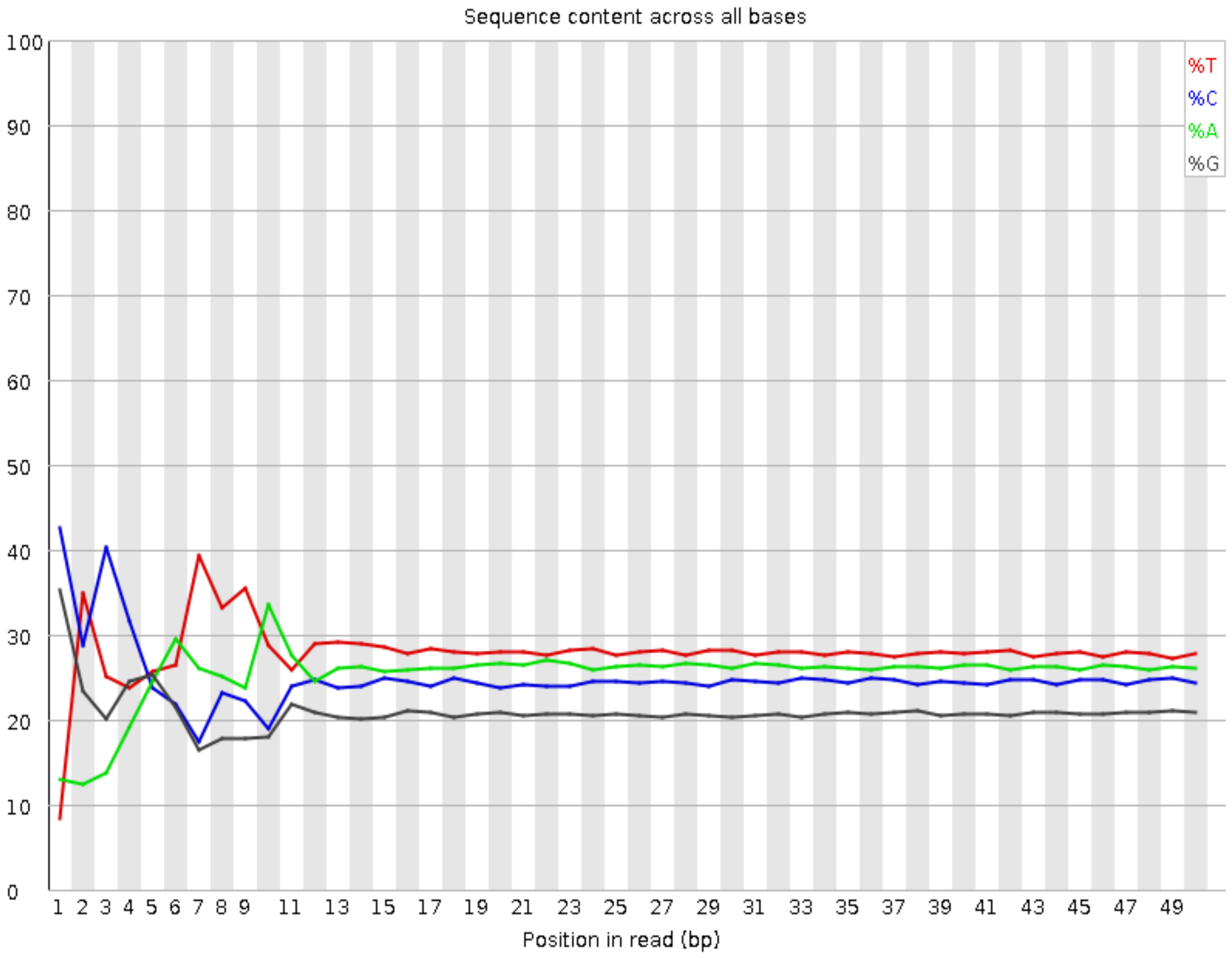

Per sequence GC content

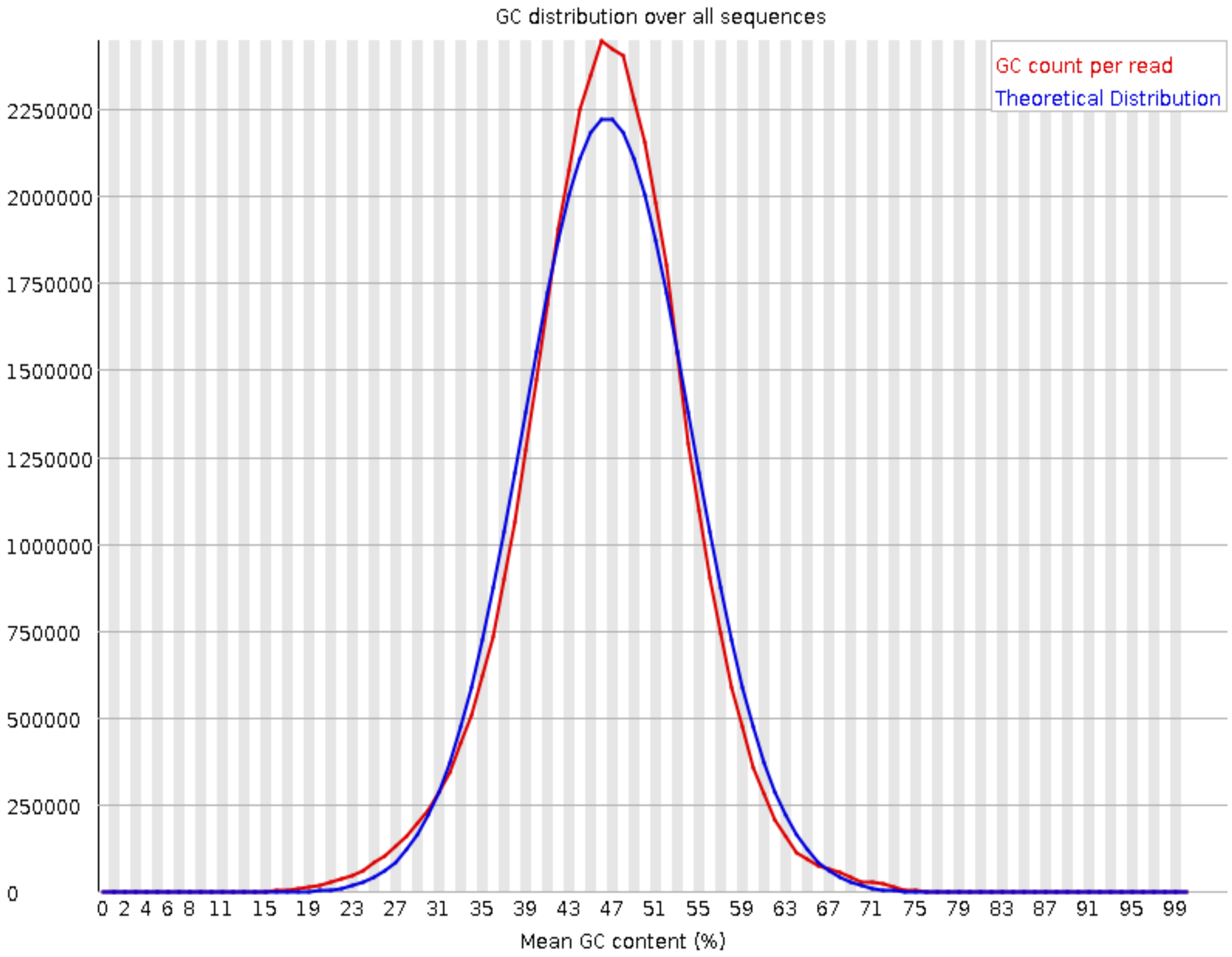

Per base N content

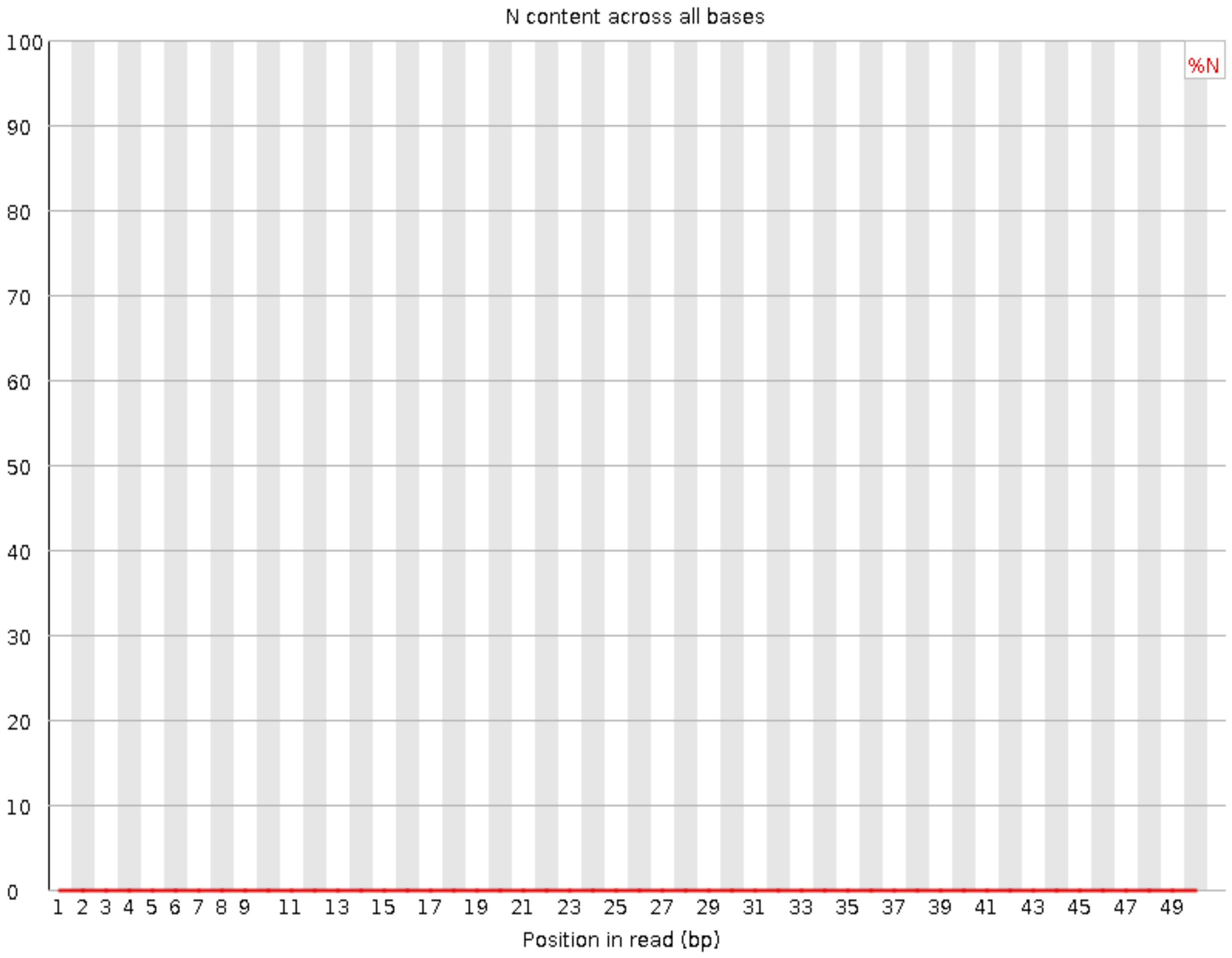

Sequence Length Distribution

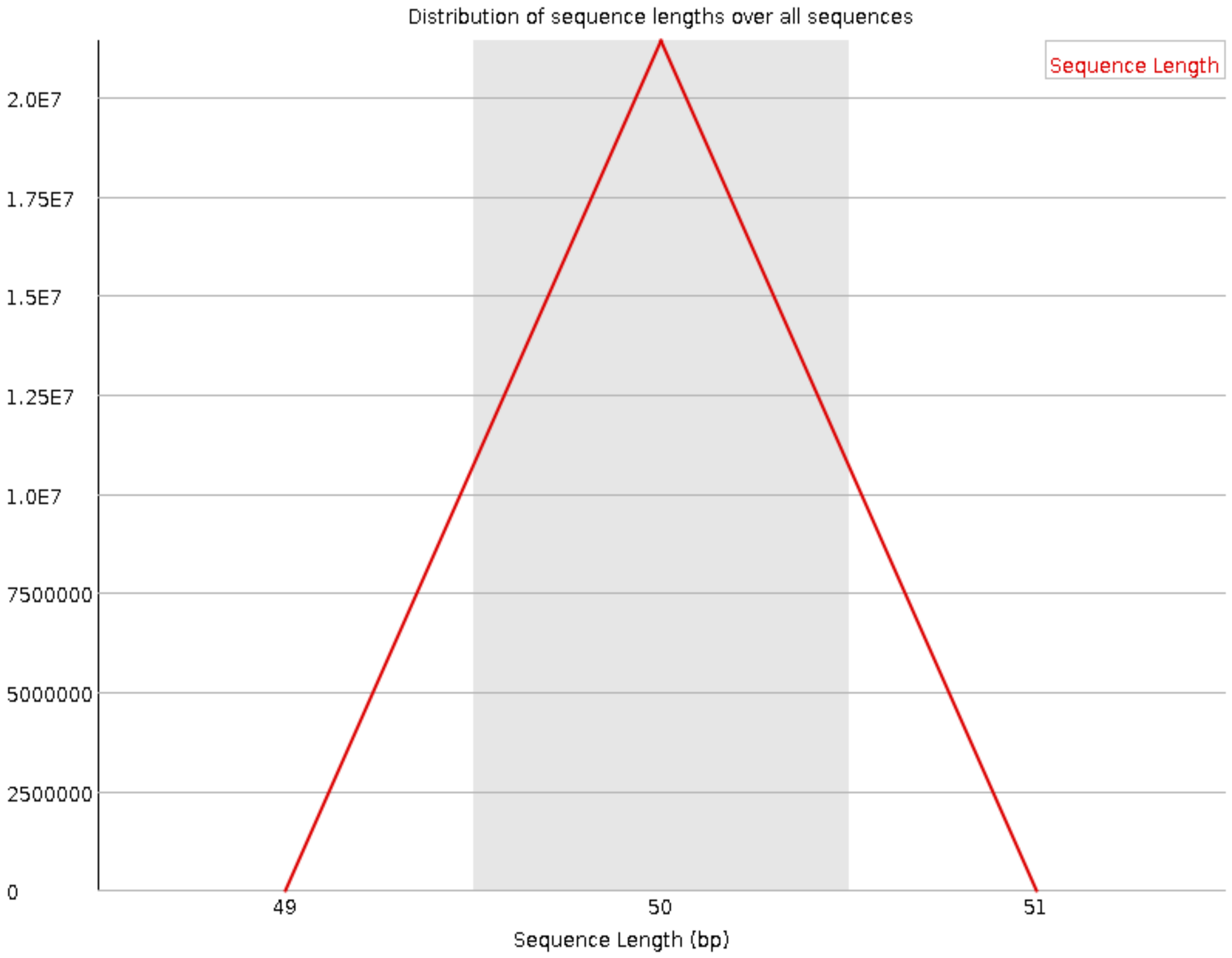

Sequence Duplication Levels

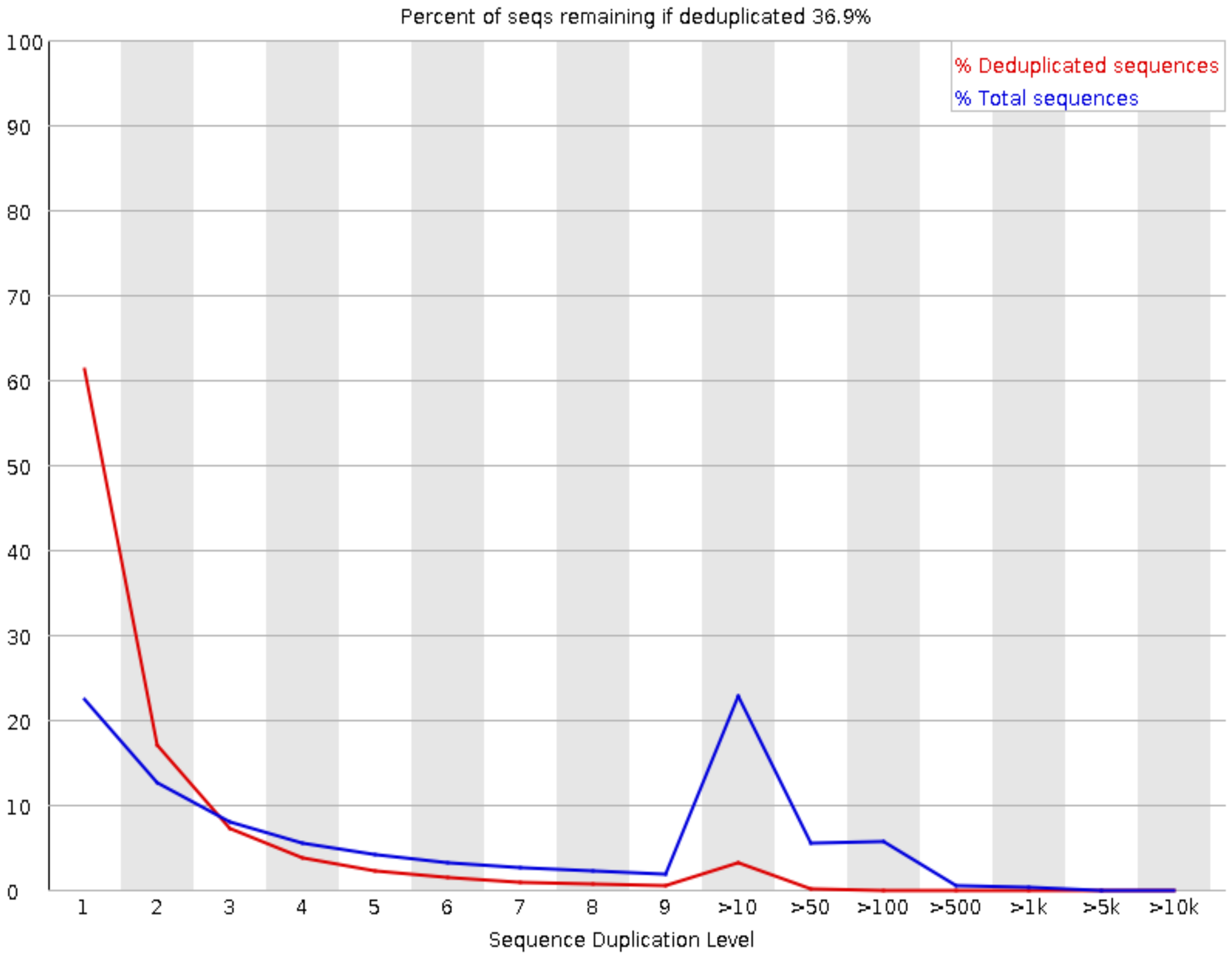

Overrepresented sequences

No overrepresented sequences

Adapter Content

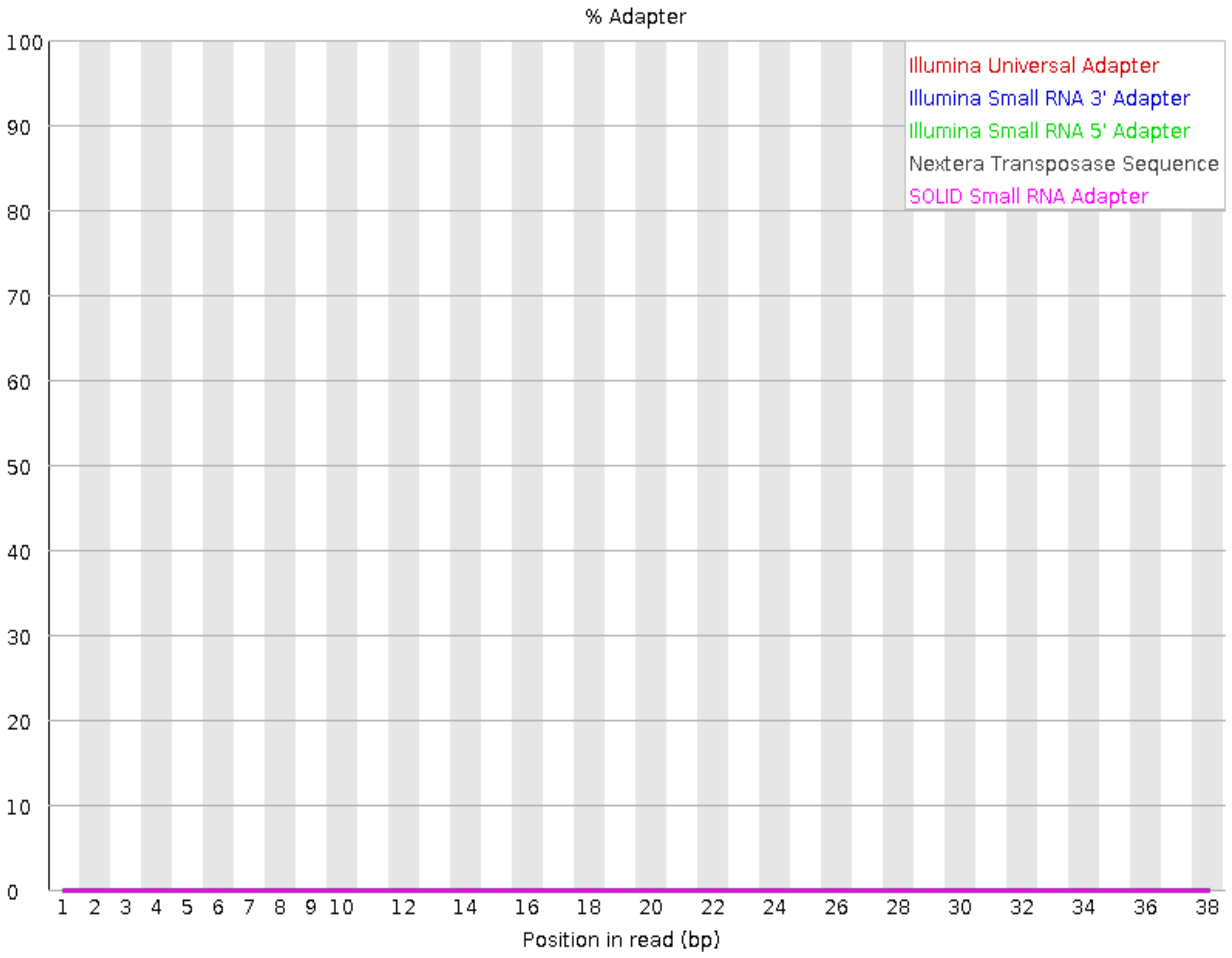

Kmer Content

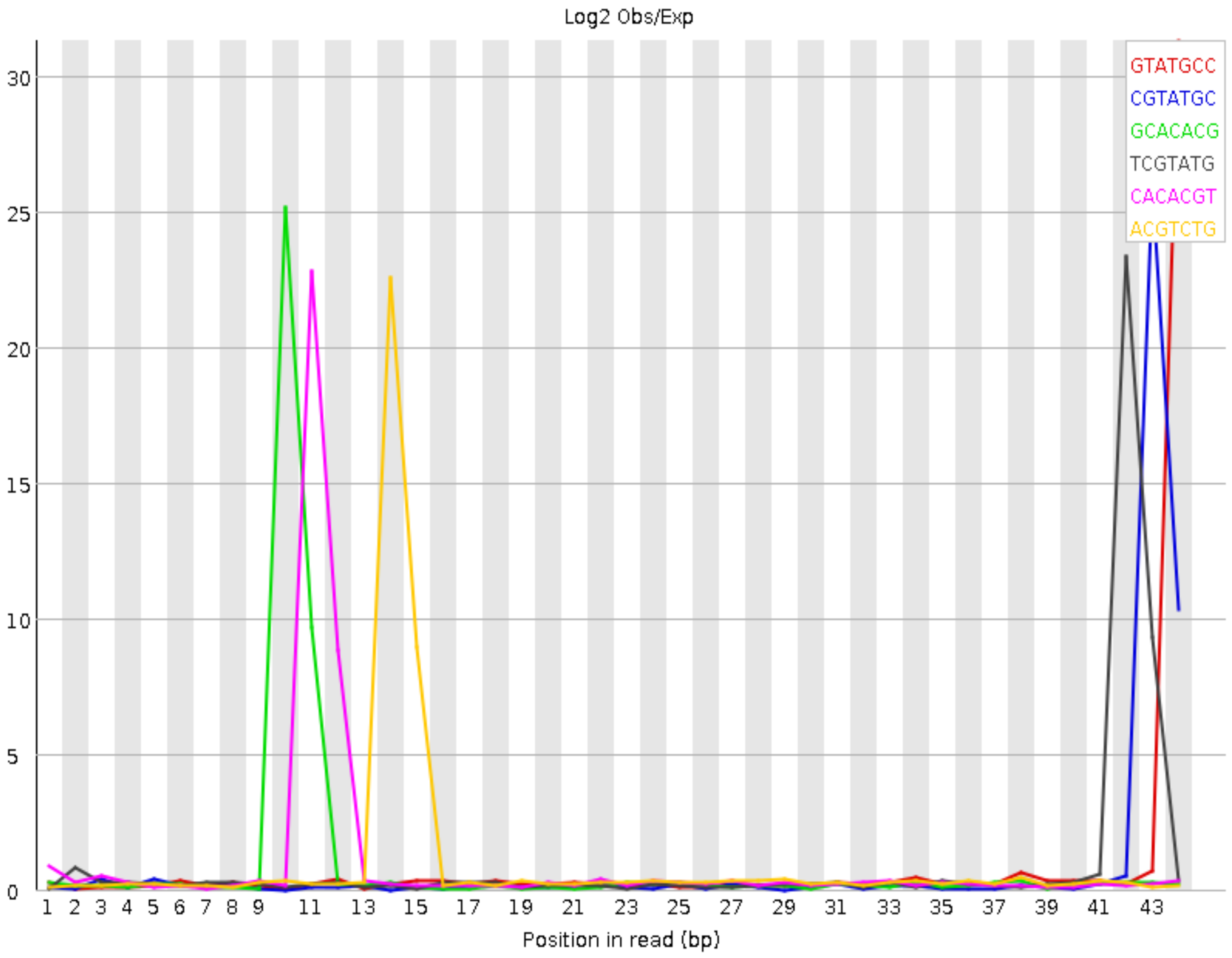

Sequence	Count	PValue	Obs/Exp Max	Max Obs/Exp Position
GTATGCC	6835	0.0	31.288307	44
CGTATGC	8200	0.0	26.02623	43
GCACACG	8655	0.0	25.18329	10
TCGTATG	9140	0.0	23.34941	42
CACACGT	9625	0.0	22.851051	11
ACGTCTG	9665	0.0	22.620468	14
ACACGTC	10410	0.0	21.06451	12
GAGCACA	10800	0.0	20.364893	8
AGCACAC	10995	0.0	20.08373	9
TCGGAAG	11680	0.0	19.811174	2
CTCGTAT	10930	0.0	19.706656	41
CGTCTGA	11390	0.0	19.407043	15
CACGTCT	11710	0.0	18.78231	13

Sequence	Count	PValue	Obs/Exp Max	Max Obs/Exp Position
GTCACAT	11990	0.0	18.292162	28
AGAGCAC	12205	0.0	18.164719	7
CGGAAGA	12685	0.0	18.154886	3
ATCGGAA	13170	0.0	18.088068	1
CACGATC	12350	0.0	17.599705	35
TCACGAT	12865	0.0	17.132254	34
ACTCCAG	13135	0.0	16.680506	22

Produced by FastQC (version 0.11.5)

FastQC Report

Tue 12 Sep 2017
22003_ACAGTG_L001_R1.fastq.gz

Summary

- Basic Statistics
- Per base sequence quality
- Per tile sequence quality
- Per sequence quality scores
- Per base sequence content
- Per sequence GC content
- Per base N content
- Sequence Length Distribution
- Sequence Duplication Levels
- Overrepresented sequences
- Adapter Content
- Kmer Content

Basic Statistics

Measure	Value
Filename	22003_ACAGTG_L001_R1_001.fastq.gz
File type	Conventional base calls
Encoding	Sanger / Illumina 1.9
Total Sequences	23633429
Sequences flagged as poor quality	0
Sequence length	50
%GC	46

Per base sequence quality

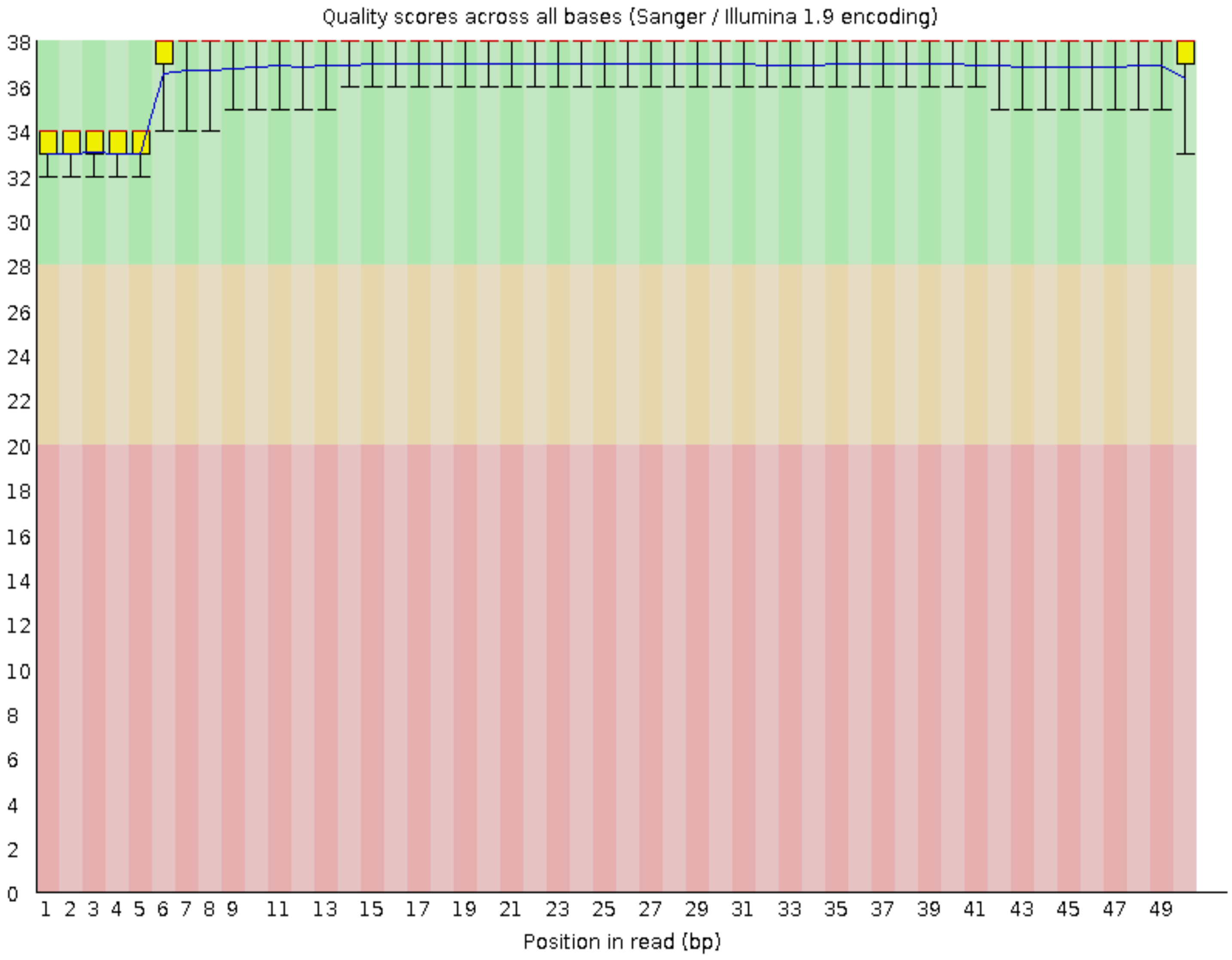

Per tile sequence quality

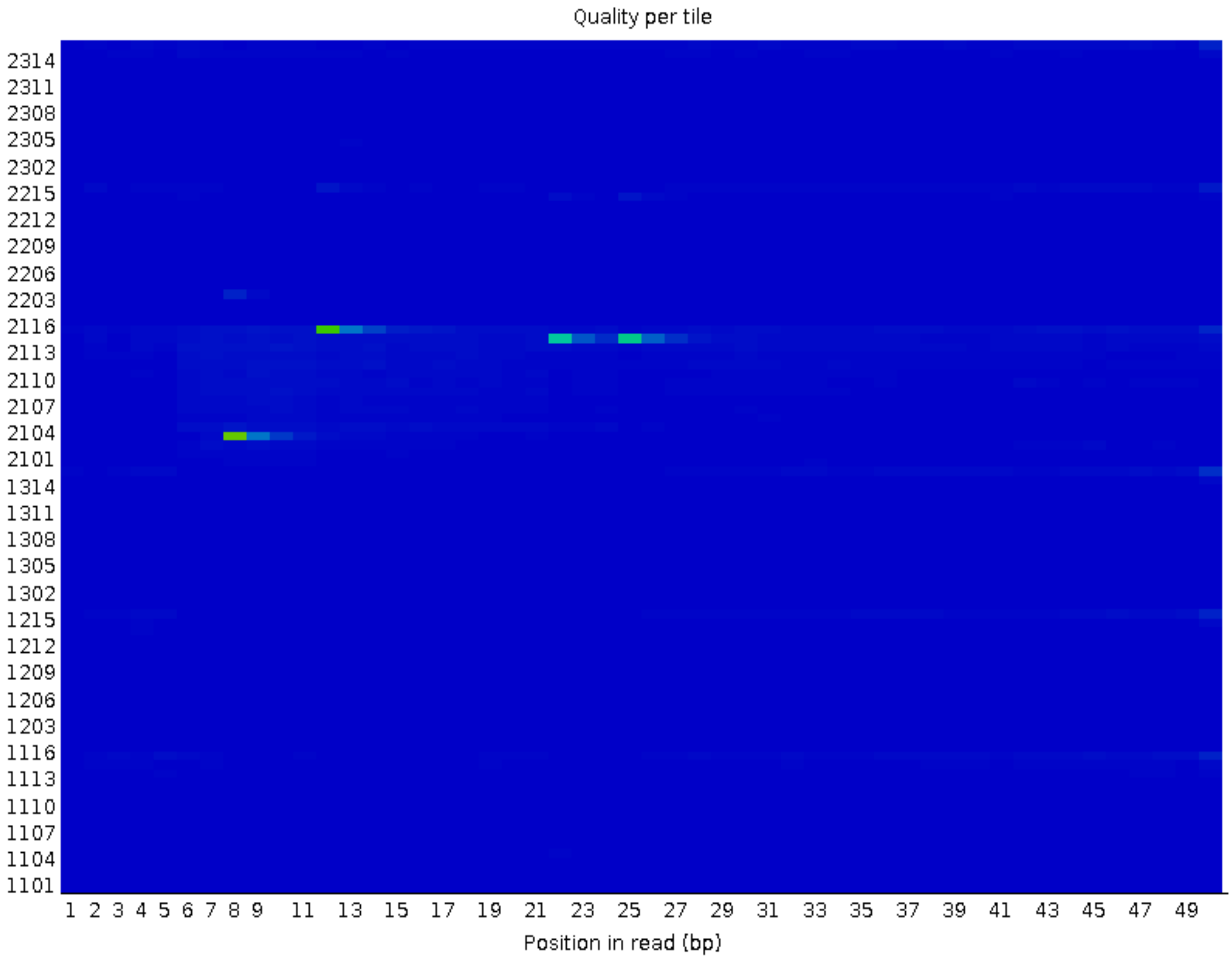

Per sequence quality scores

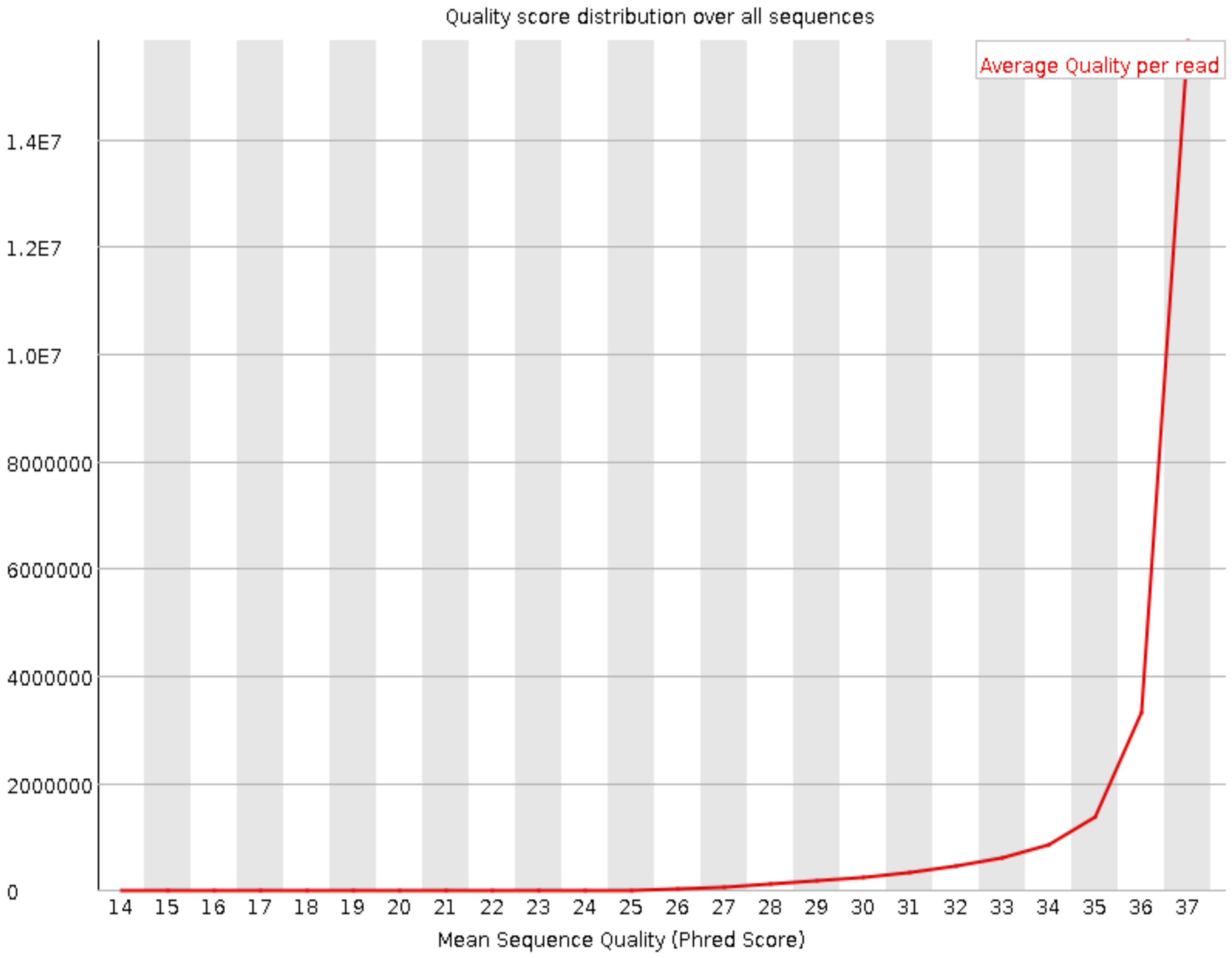

Per base sequence content

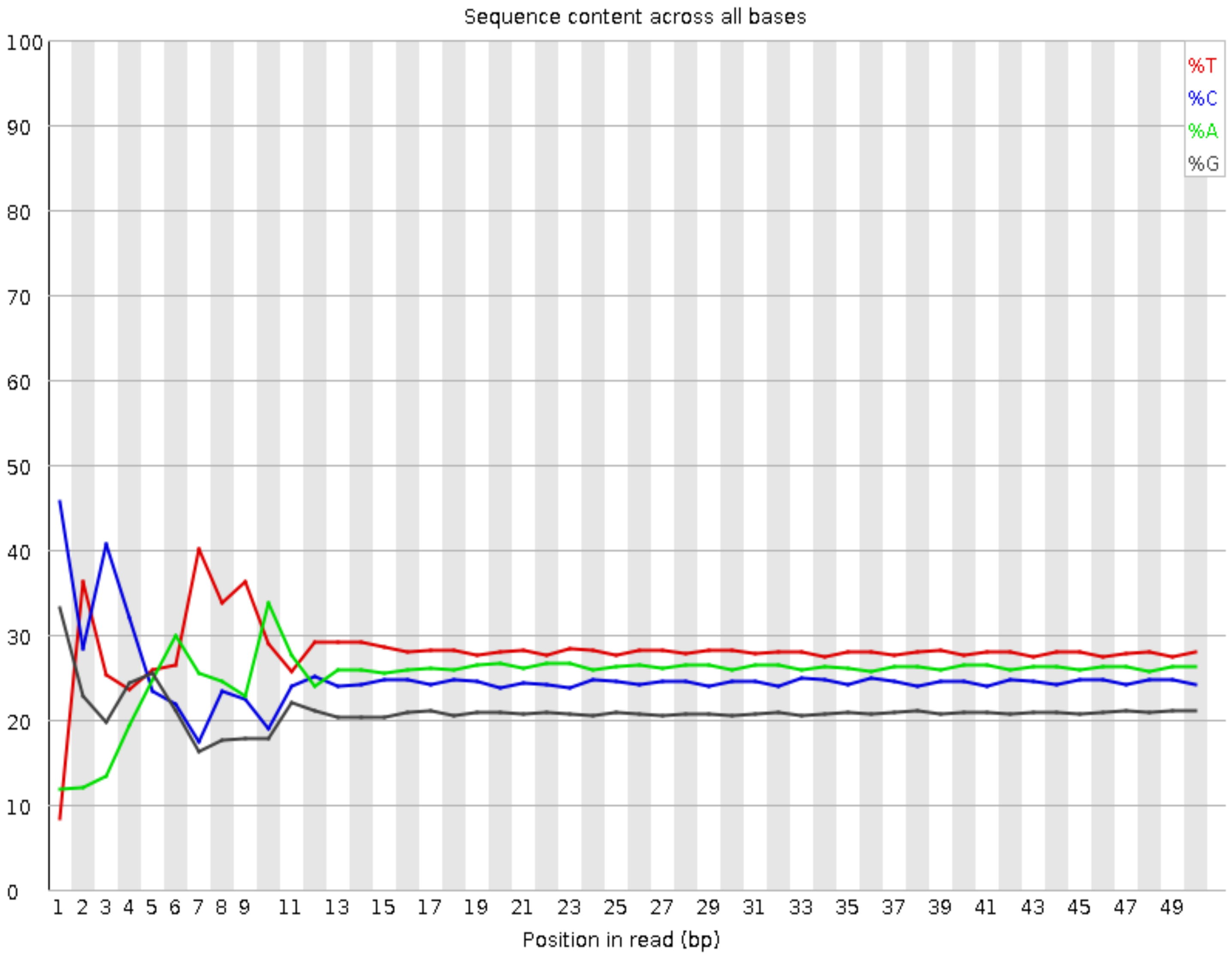

Per sequence GC content

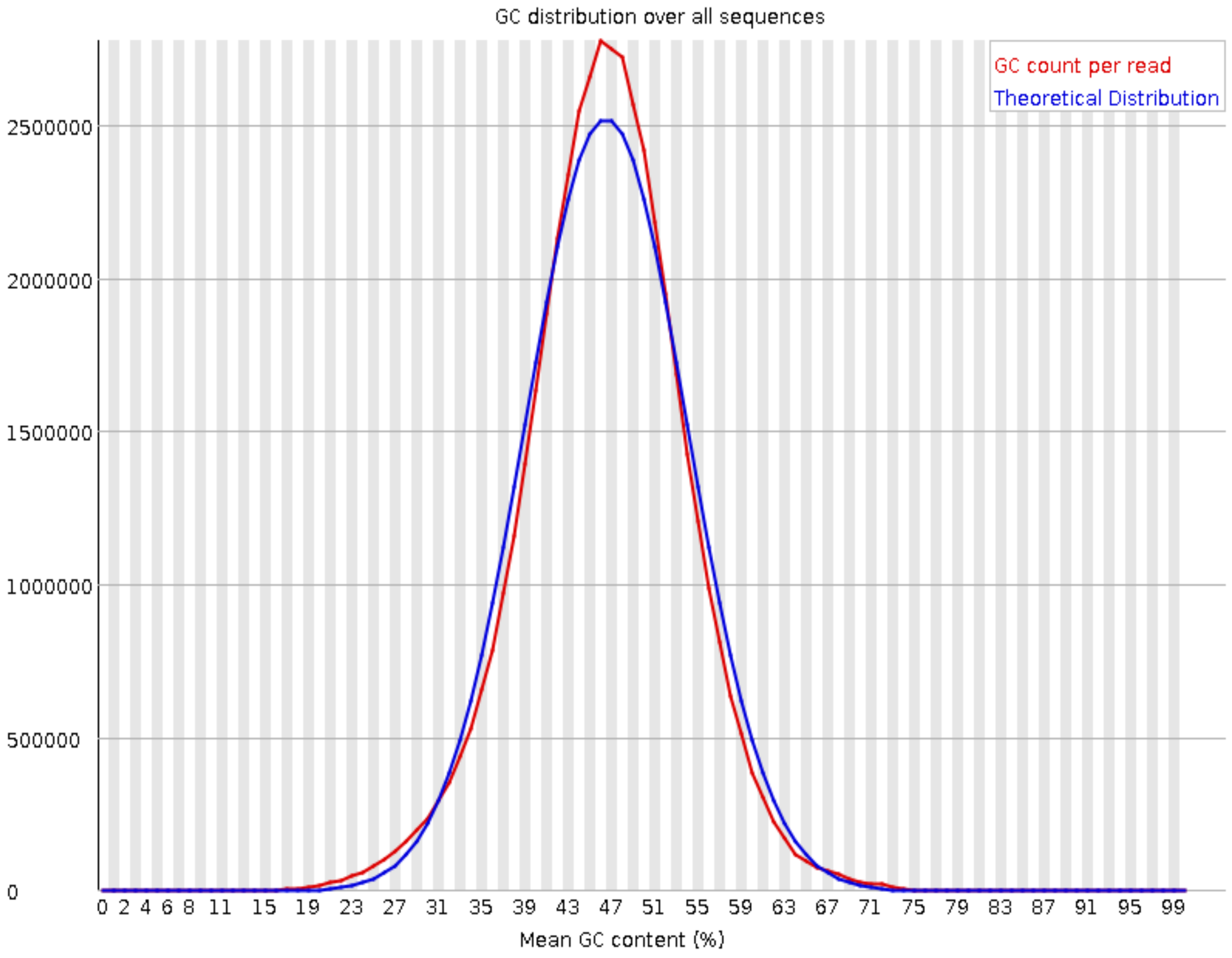

Per base N content

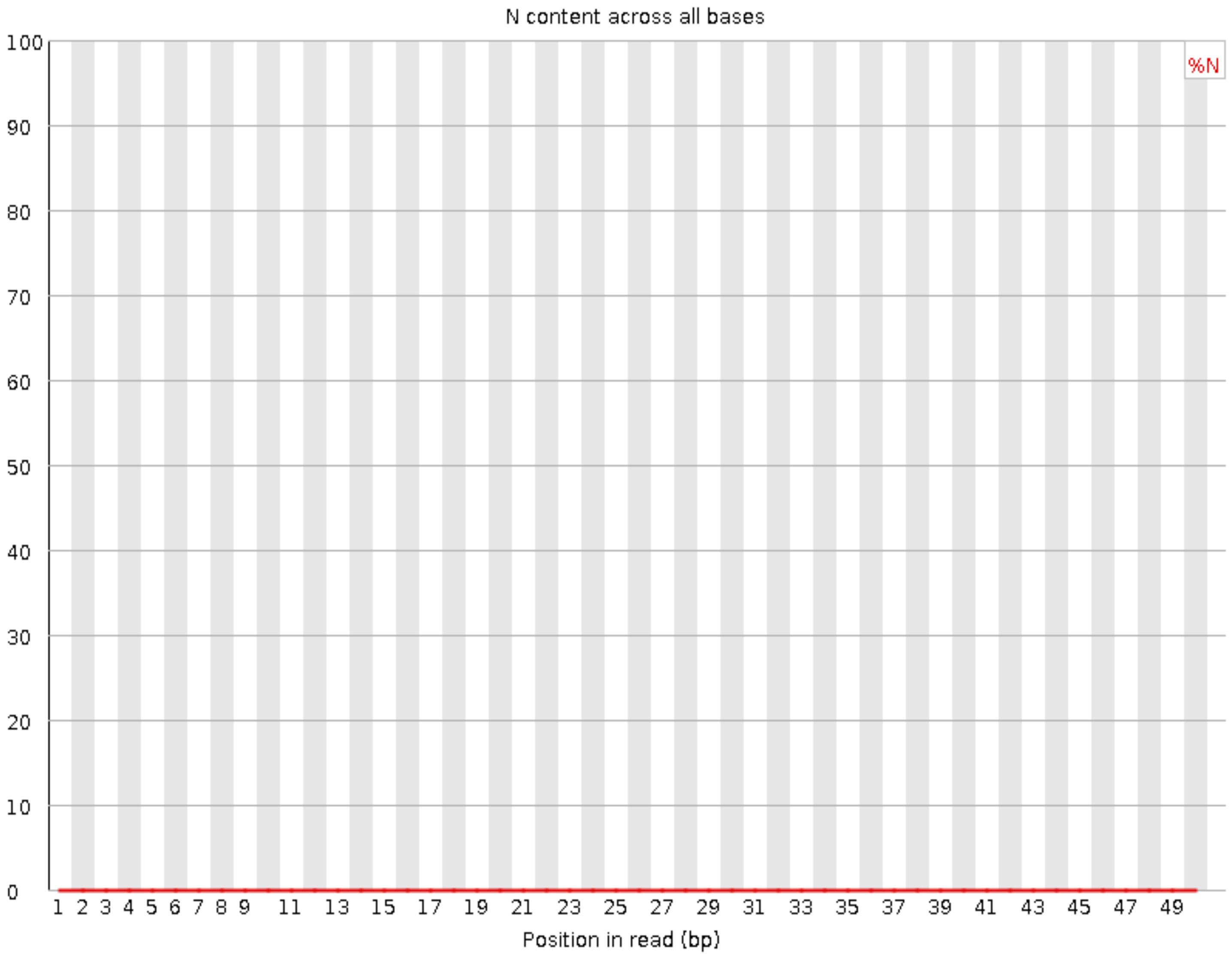

Sequence Length Distribution

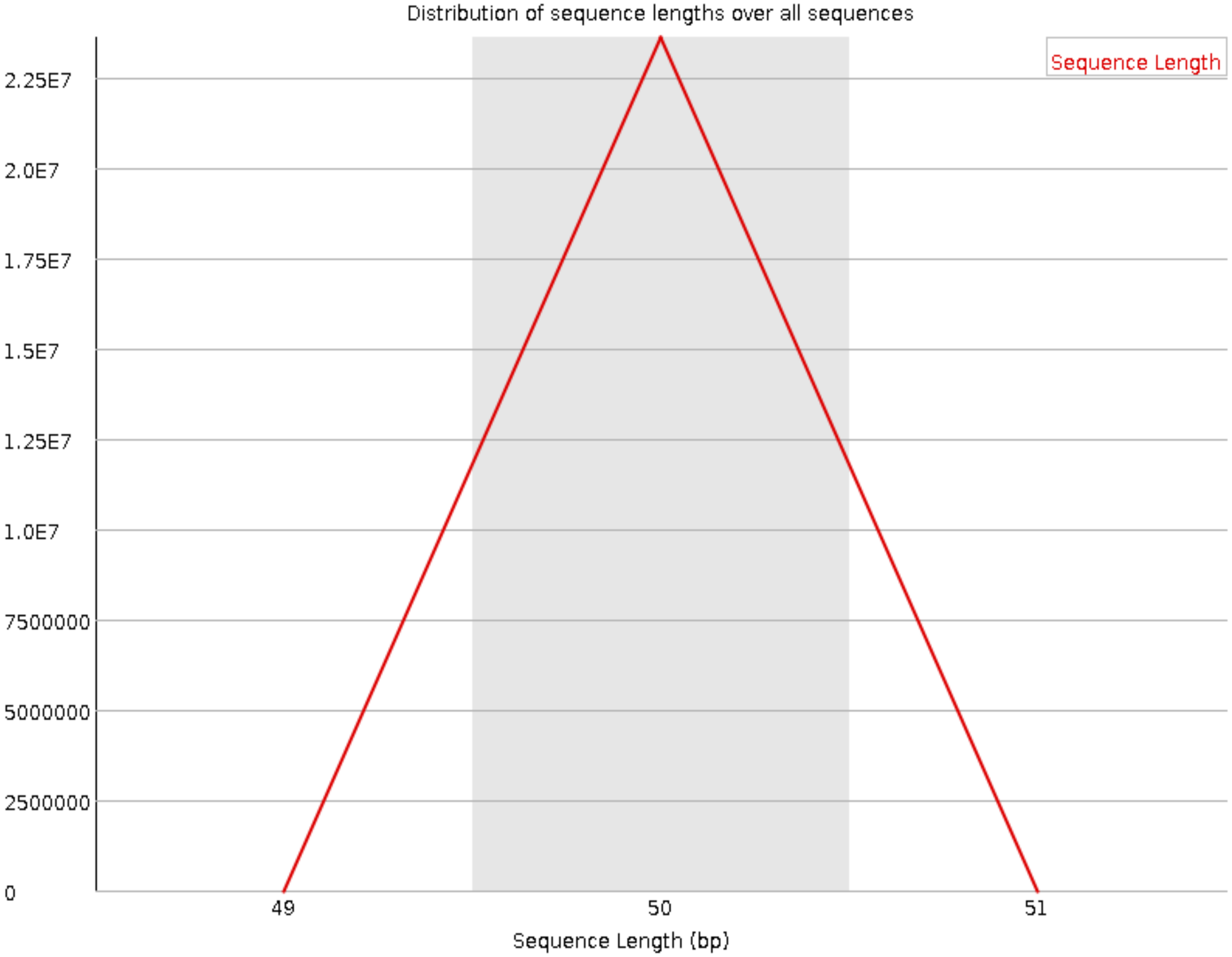

Sequence Duplication Levels

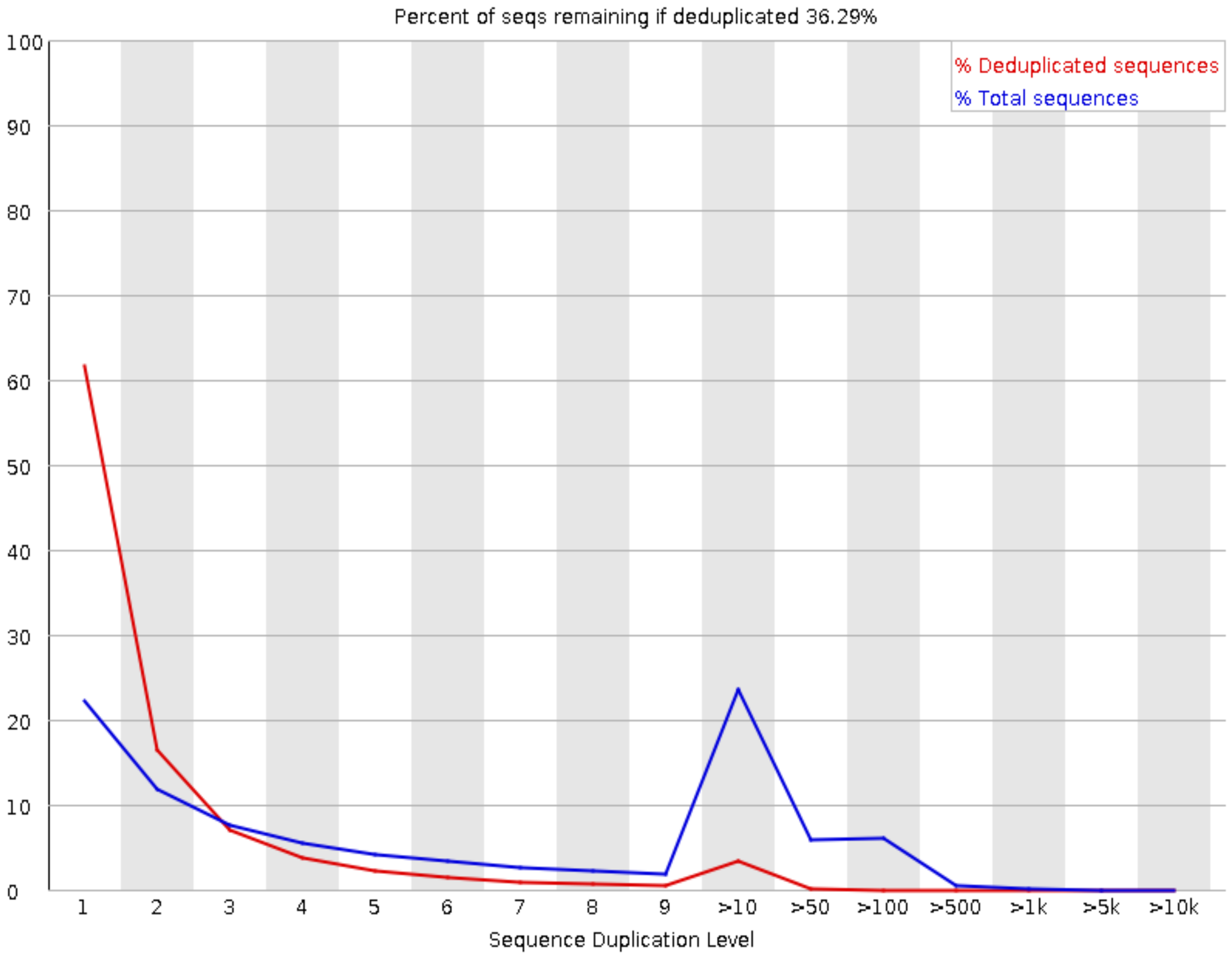

Overrepresented sequences

No overrepresented sequences

Adapter Content

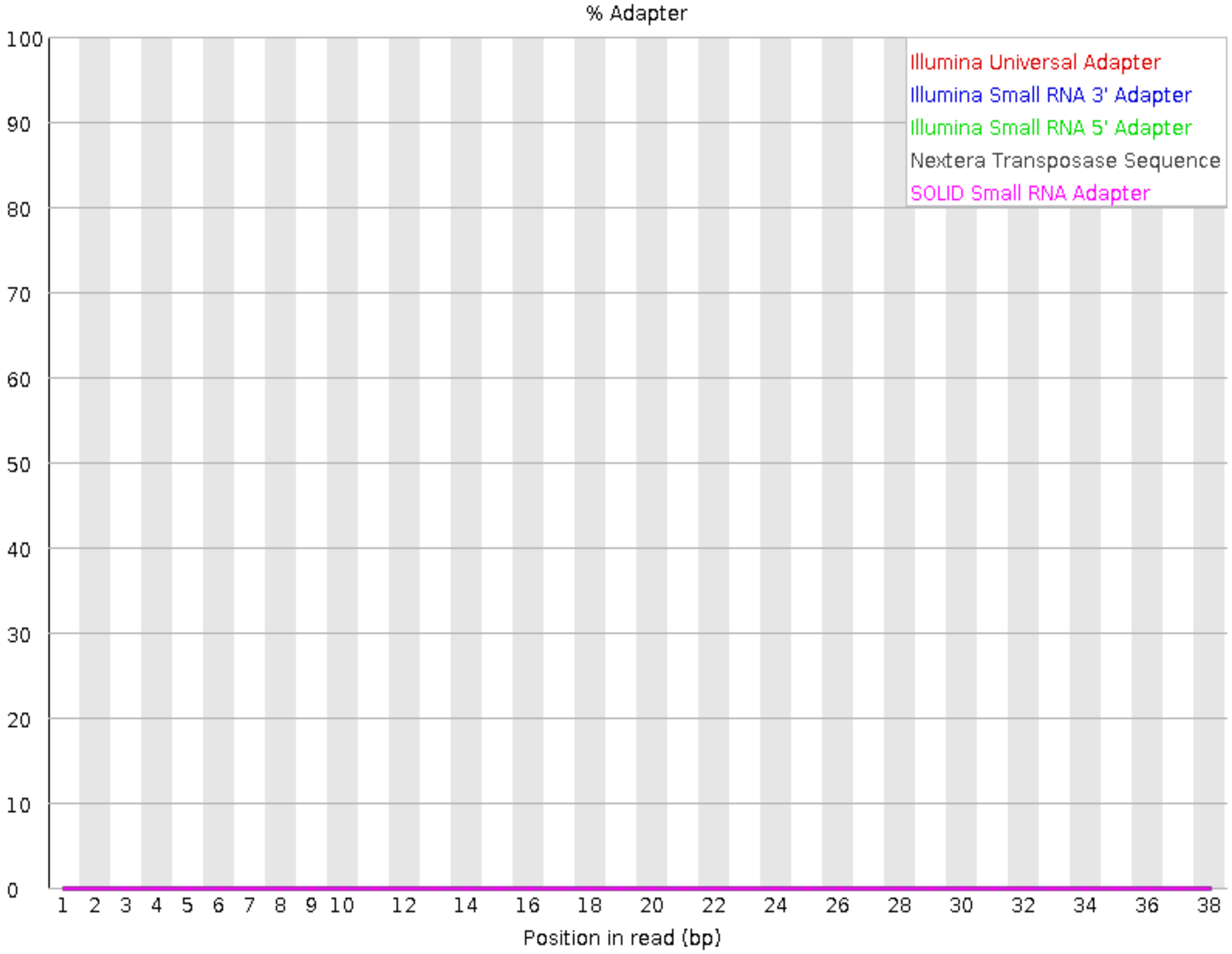

Kmer Content

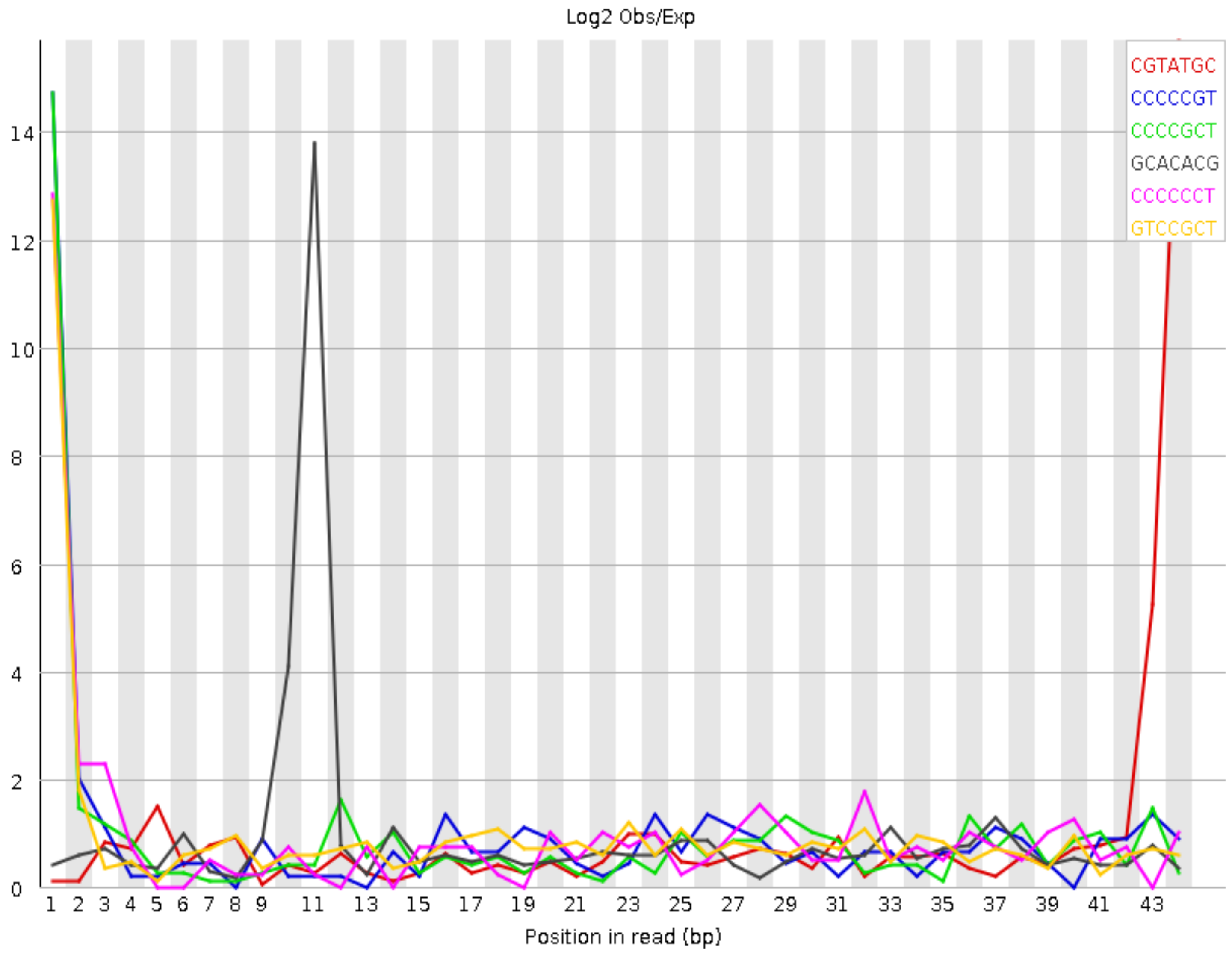

Sequence	Count	PValue	Obs/Exp Max	Max Obs/Exp Position
CGTATGC	3020	0.0	15.678765	44
CCCCCGT	970	0.0	14.739827	1
CCCCGCT	1465	0.0	14.714286	1
GCACACG	3520	0.0	13.808928	11
CCCCCCT	855	0.0	12.863367	1
GTCCGCT	1795	0.0	12.744407	1
TCGTATG	3910	0.0	12.447894	43
GGGGGAT	1720	0.0	12.277037	1
GGGGGCT	1880	0.0	11.934193	1
GGGGGGT	525	0.0	11.731389	1
ACGTCTG	4130	0.0	11.663113	15
GGGGGCA	1045	0.0	11.57703	1
GGGGCCT	900	0.0	11.486986	1

Sequence	Count	PValue	Obs/Exp Max	Max Obs/Exp Position
CACACGT	4395	0.0	11.359974	12
GCCCTAT	1105	0.0	11.346538	1
CCCCGGT	1545	0.0	11.247328	1
GGGGGTC	575	0.0	11.093815	1
CGGGCGC	855	0.0	11.061724	17
GGGGTGT	1200	0.0	10.9981785	1
CCCCCTT	2410	0.0	10.952542	1

Produced by FastQC (version 0.11.5)

FastQC Report

Tue 12 Sep 2017
22004_GCCAAT_L001_R1.fastq.gz

Summary

- Basic Statistics
- Per base sequence quality
- Per tile sequence quality
- Per sequence quality scores
- Per base sequence content
- Per sequence GC content
- Per base N content
- Sequence Length Distribution
- Sequence Duplication Levels
- Overrepresented sequences
- Adapter Content
- Kmer Content

Basic Statistics

Measure	Value
Filename	22004_GCCAAT_L001_R1_001.fastq.gz
File type	Conventional base calls
Encoding	Sanger / Illumina 1.9
Total Sequences	21187548
Sequences flagged as poor quality	0
Sequence length	50
%GC	46

Per base sequence quality

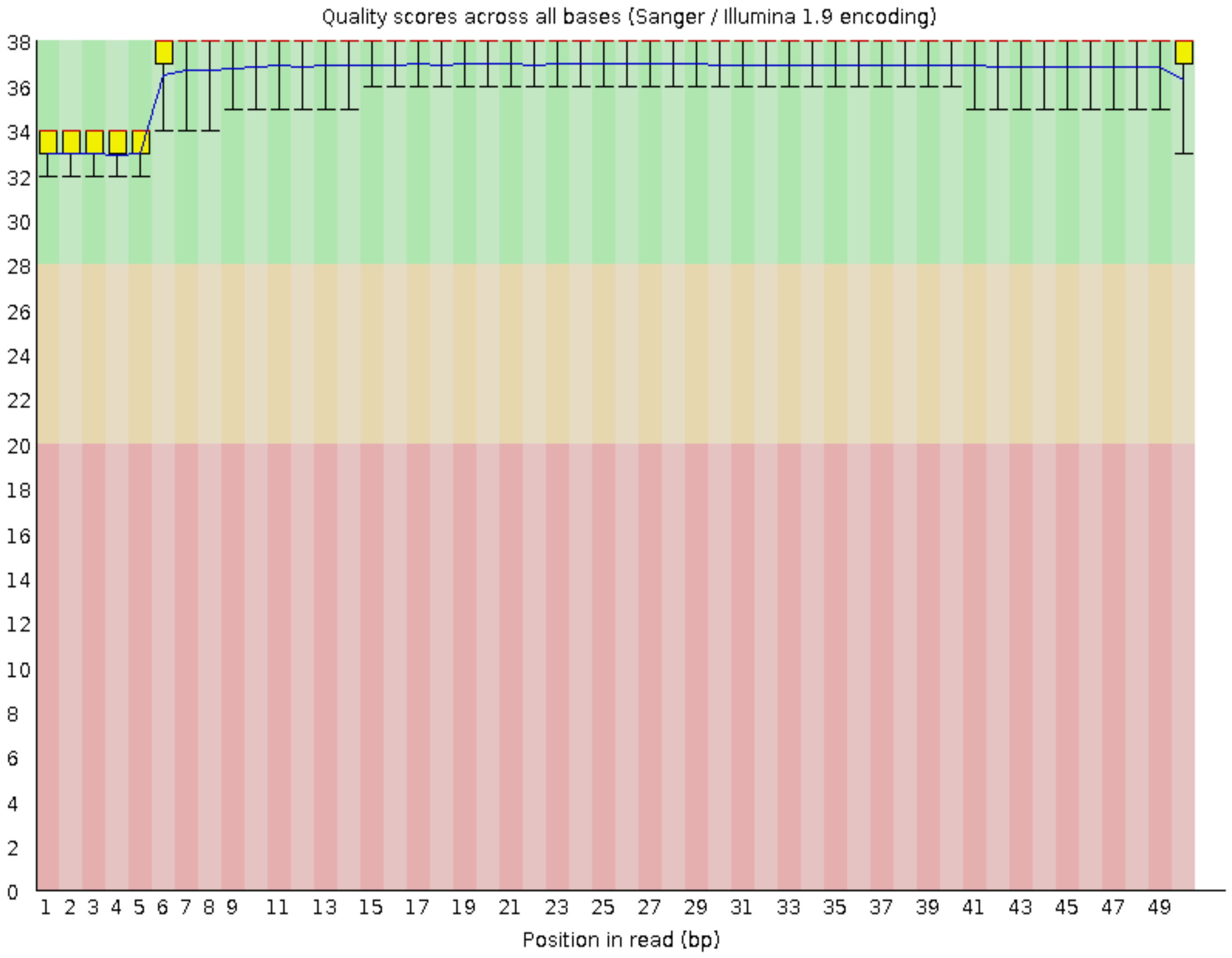

Per tile sequence quality

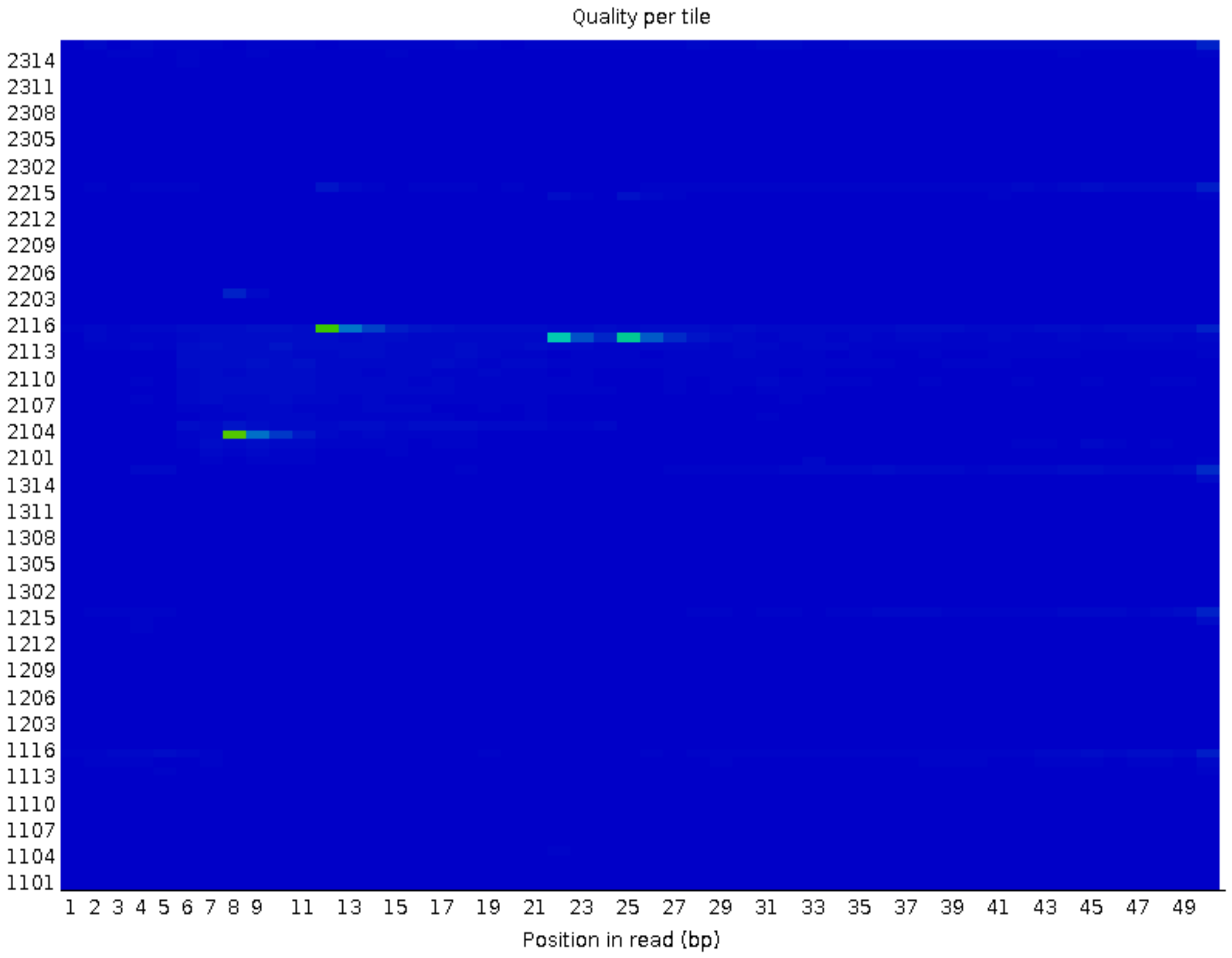

Per sequence quality scores

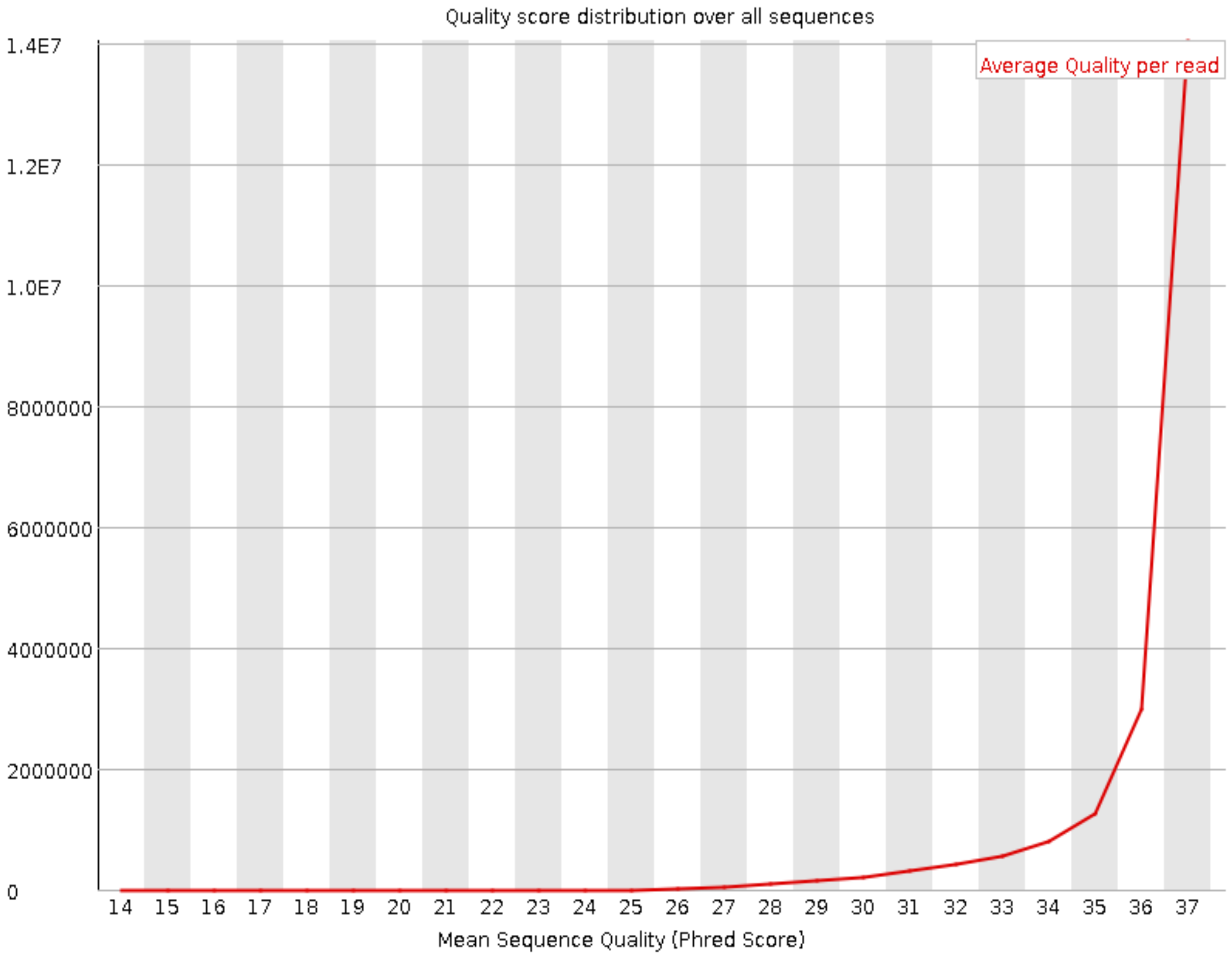

Per base sequence content

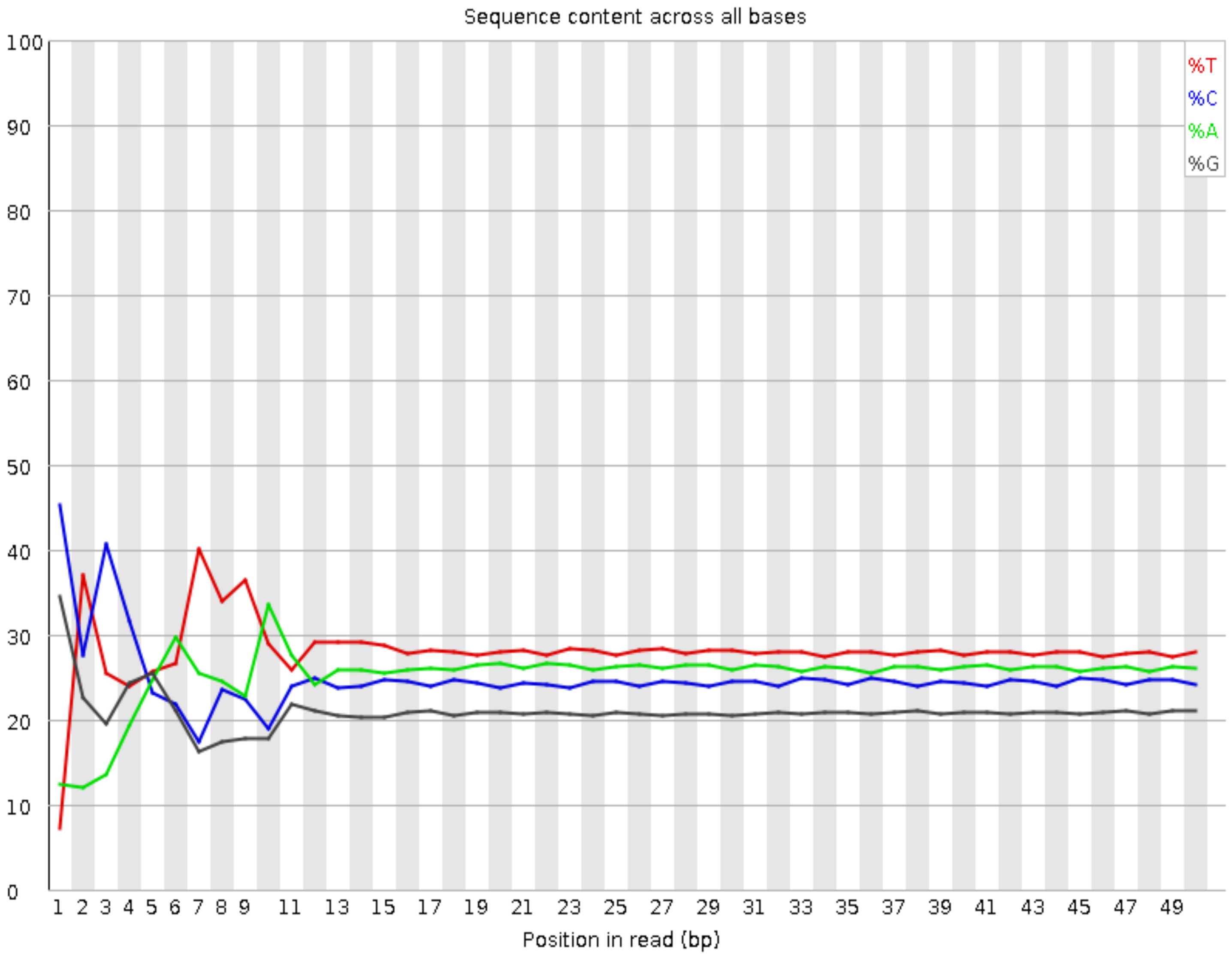

Per sequence GC content

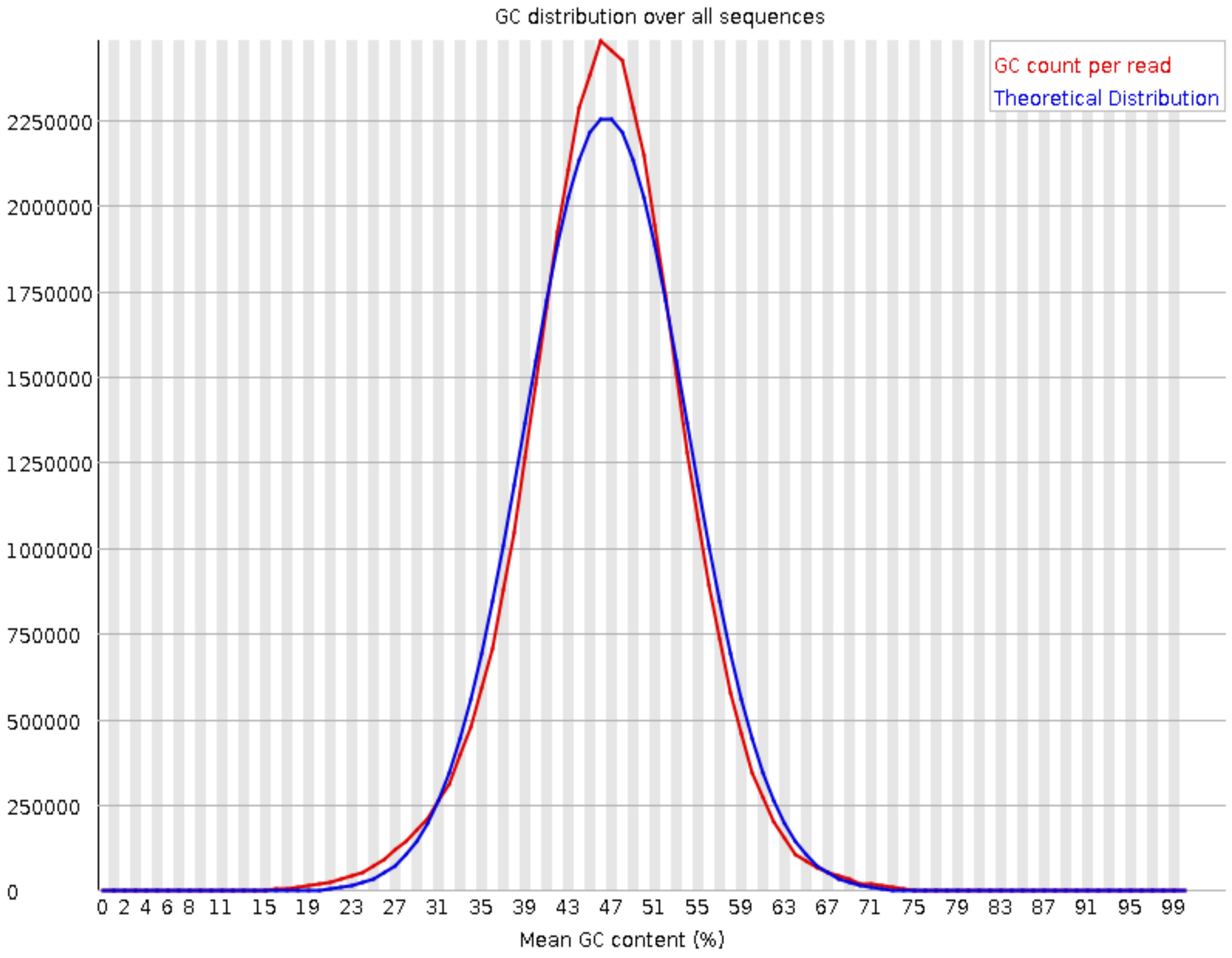

Per base N content

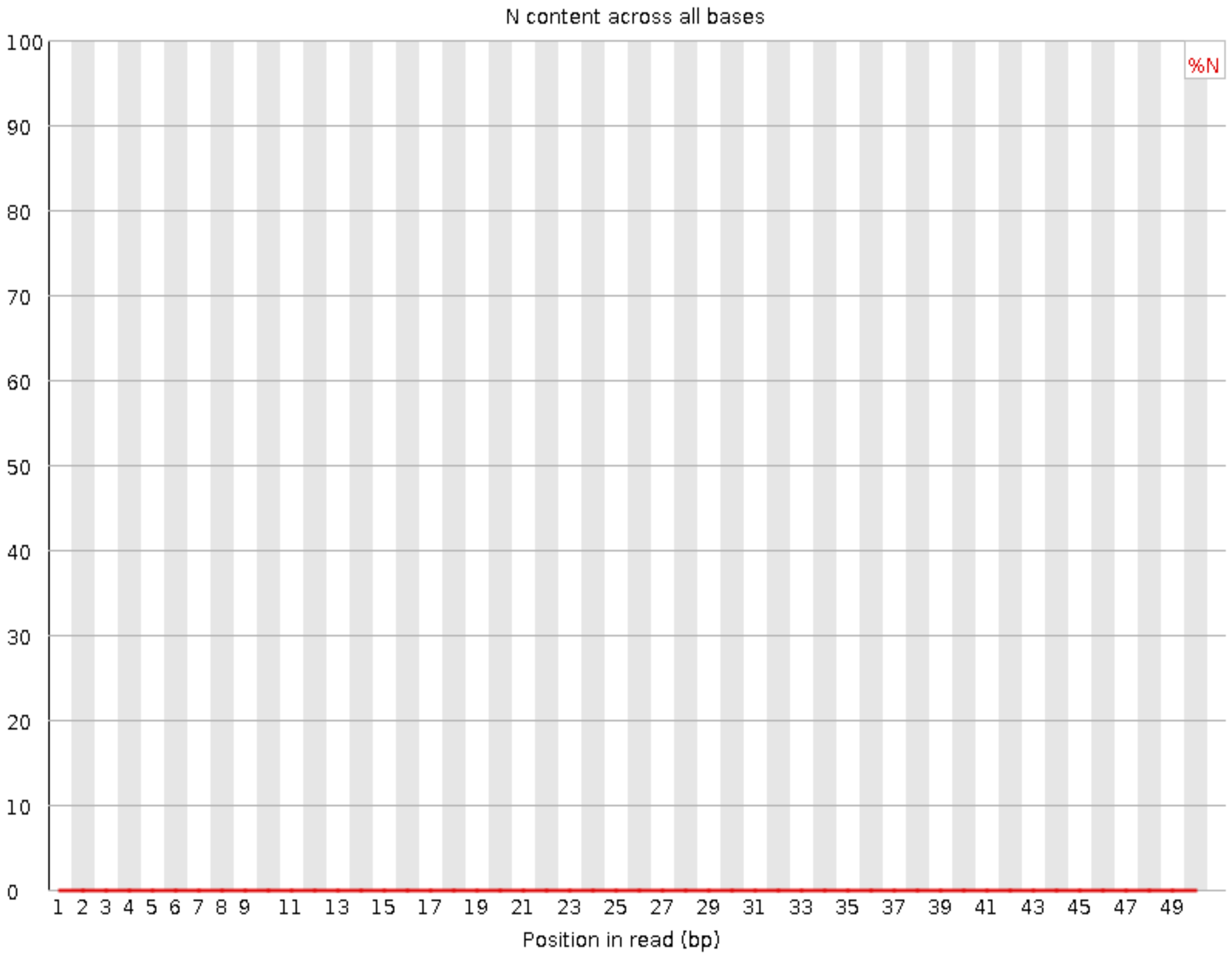

Sequence Length Distribution

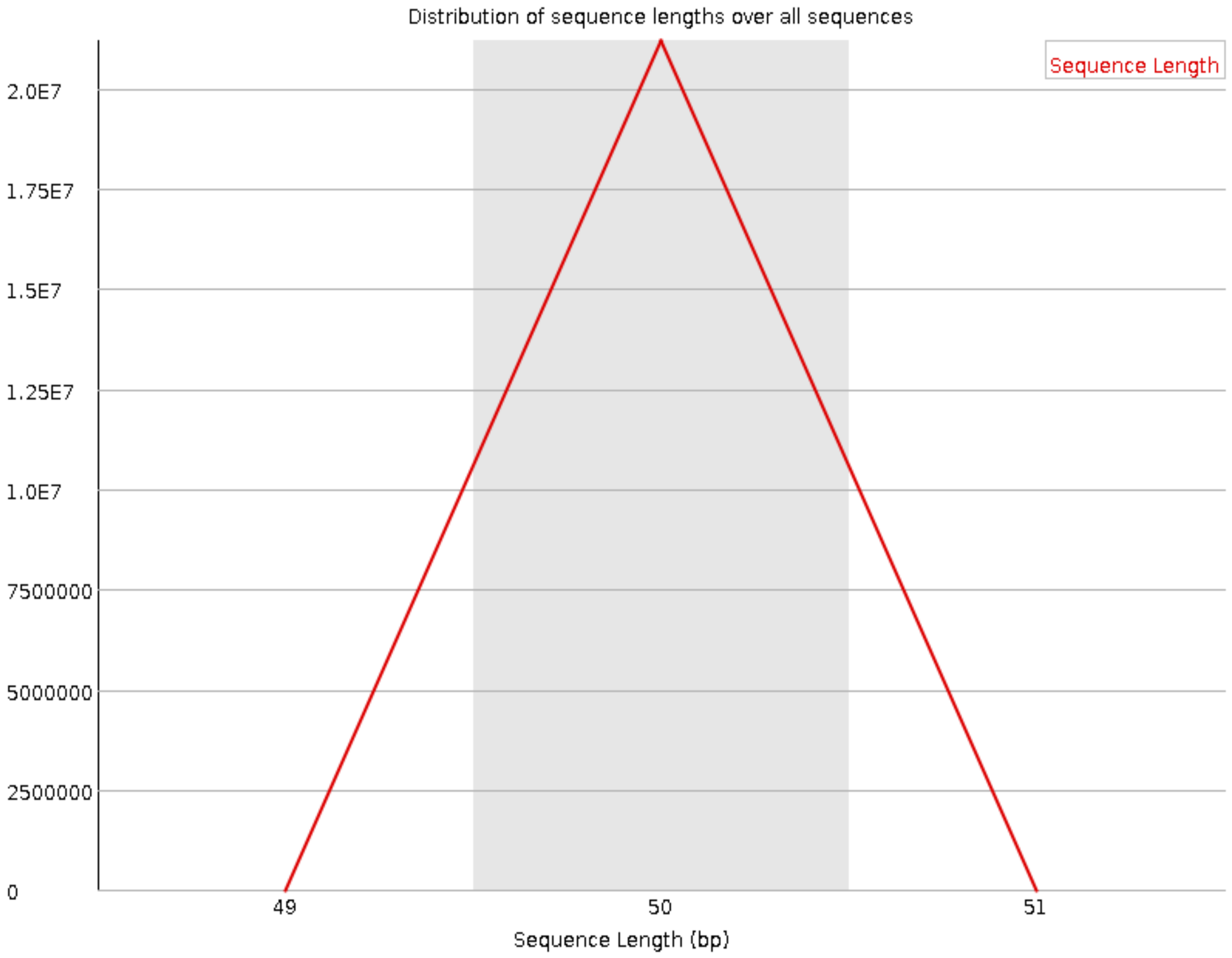

Sequence Duplication Levels

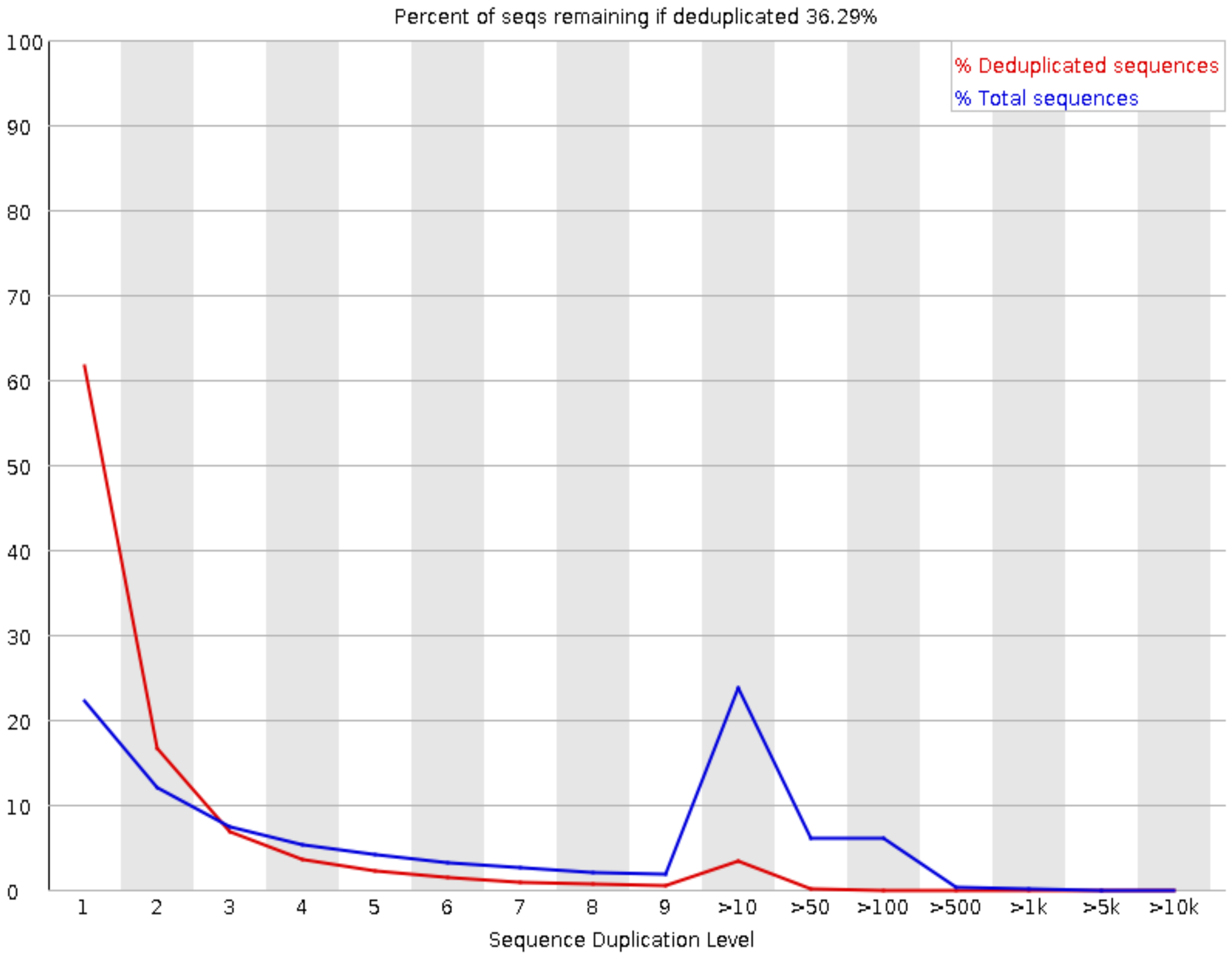

Overrepresented sequences

No overrepresented sequences

Adapter Content

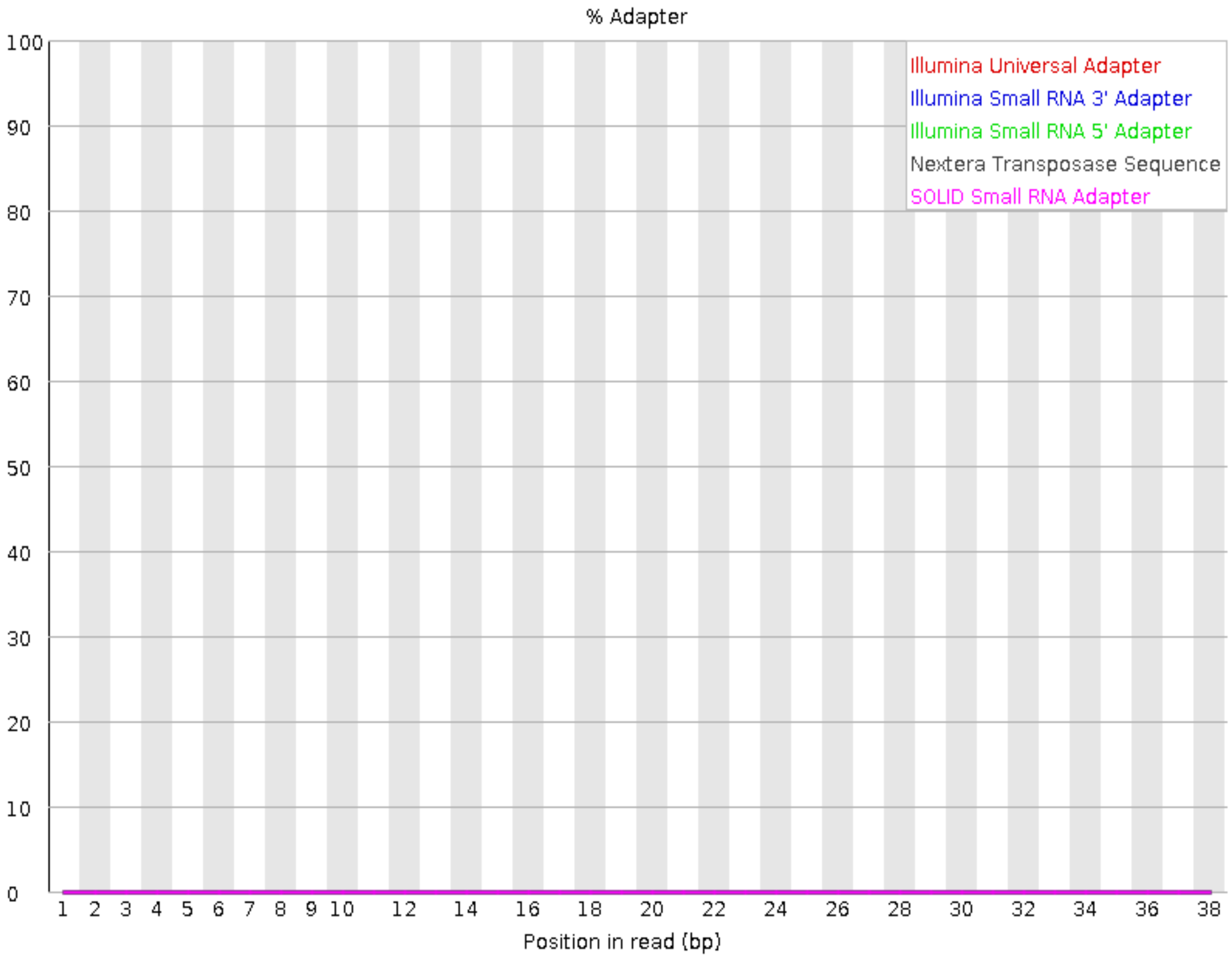

Kmer Content

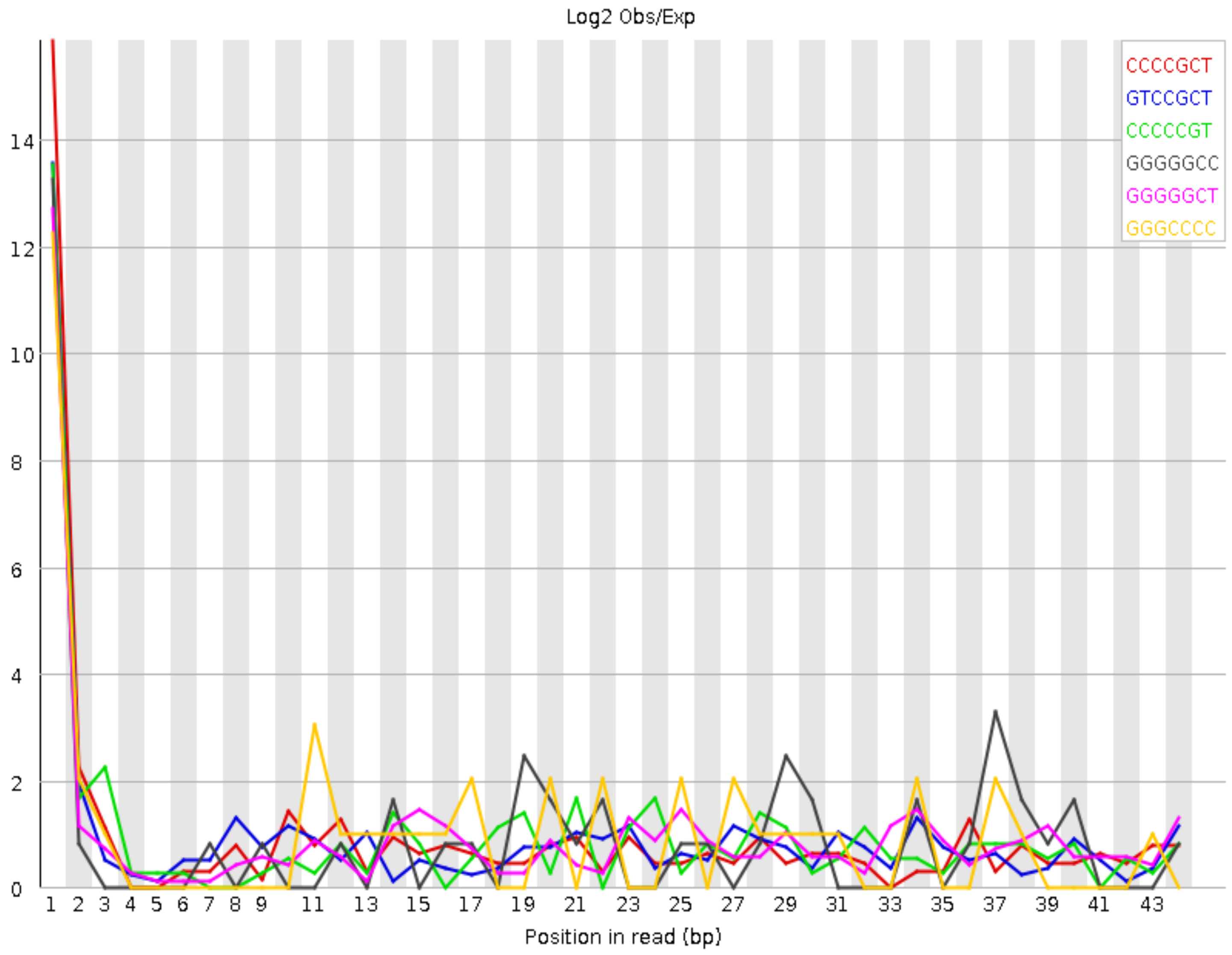

Sequence	Count	PValue	Obs/Exp Max	Max Obs/Exp Position
CCCCGCT	1360	0.0	15.850302	1
GTCCGCT	1670	0.0	13.566604	1
CCCCCGT	780	0.0	13.536208	1
GGGGGCC	265	3.5106495E-10	13.2808075	1
GGGGGCT	1485	0.0	12.73862	1
GGGCCCC	215	7.906656E-7	12.277025	1
CCCCGGT	1530	0.0	11.64512	1
CCCCGAT	1835	0.0	11.627492	1
GTCCCGC	1325	0.0	11.454696	1
GGGGCCT	840	0.0	11.260029	1
GGGGGGT	320	6.846676E-9	10.998169	1
CGTATGC	2295	0.0	10.939479	43
GCCCTAT	1050	0.0	10.683935	1

Sequence	Count	PValue	Obs/Exp Max	Max Obs/Exp Position
CGGGGCG	310	5.048969E-8	10.643389	1
GGGGGGG	145	0.00808166	10.618921	1
CCCCCAT	2815	0.0	10.548865	1
GTATGCC	2335	0.0	10.46913	44
GGGGGAT	1455	0.0	10.280075	1
GCCCCTT	2120	0.0	10.1681185	1
CGGGGCC	455	1.8189894E-11	10.152155	1

Produced by FastQC (version 0.11.5)

FastQC Report

Tue 12 Sep 2017
22005_ACTTGA_L001_R1.fastq.gz

Summary

- Basic Statistics
- Per base sequence quality
- Per tile sequence quality
- Per sequence quality scores
- Per base sequence content
- Per sequence GC content
- Per base N content
- Sequence Length Distribution
- Sequence Duplication Levels
- Overrepresented sequences
- Adapter Content
- Kmer Content

Basic Statistics

Measure	Value
Filename	22005_ACTTGA_L001_R1_001.fastq.gz
File type	Conventional base calls
Encoding	Sanger / Illumina 1.9
Total Sequences	21207350
Sequences flagged as poor quality	0
Sequence length	50
%GC	46

Per base sequence quality

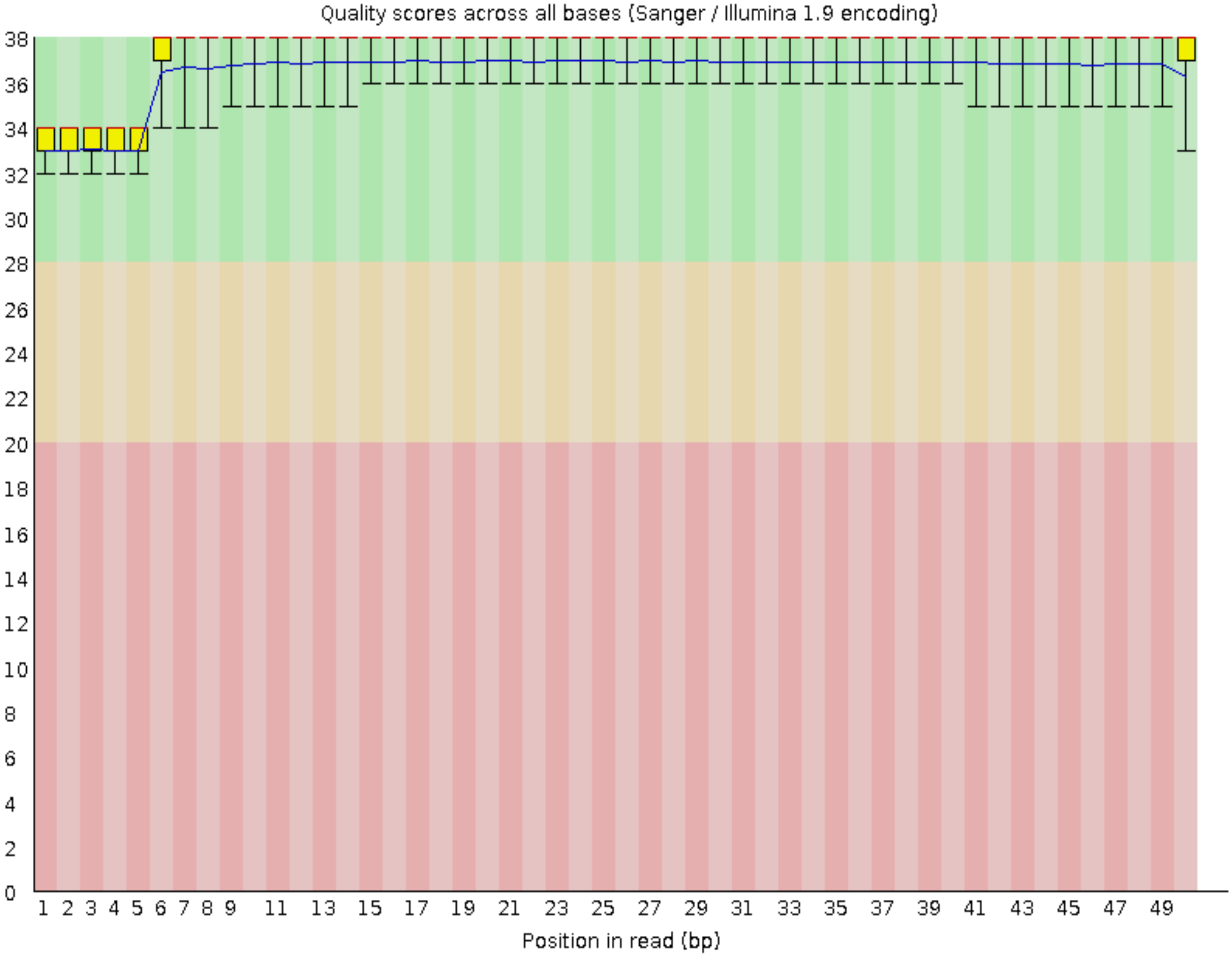

Per tile sequence quality

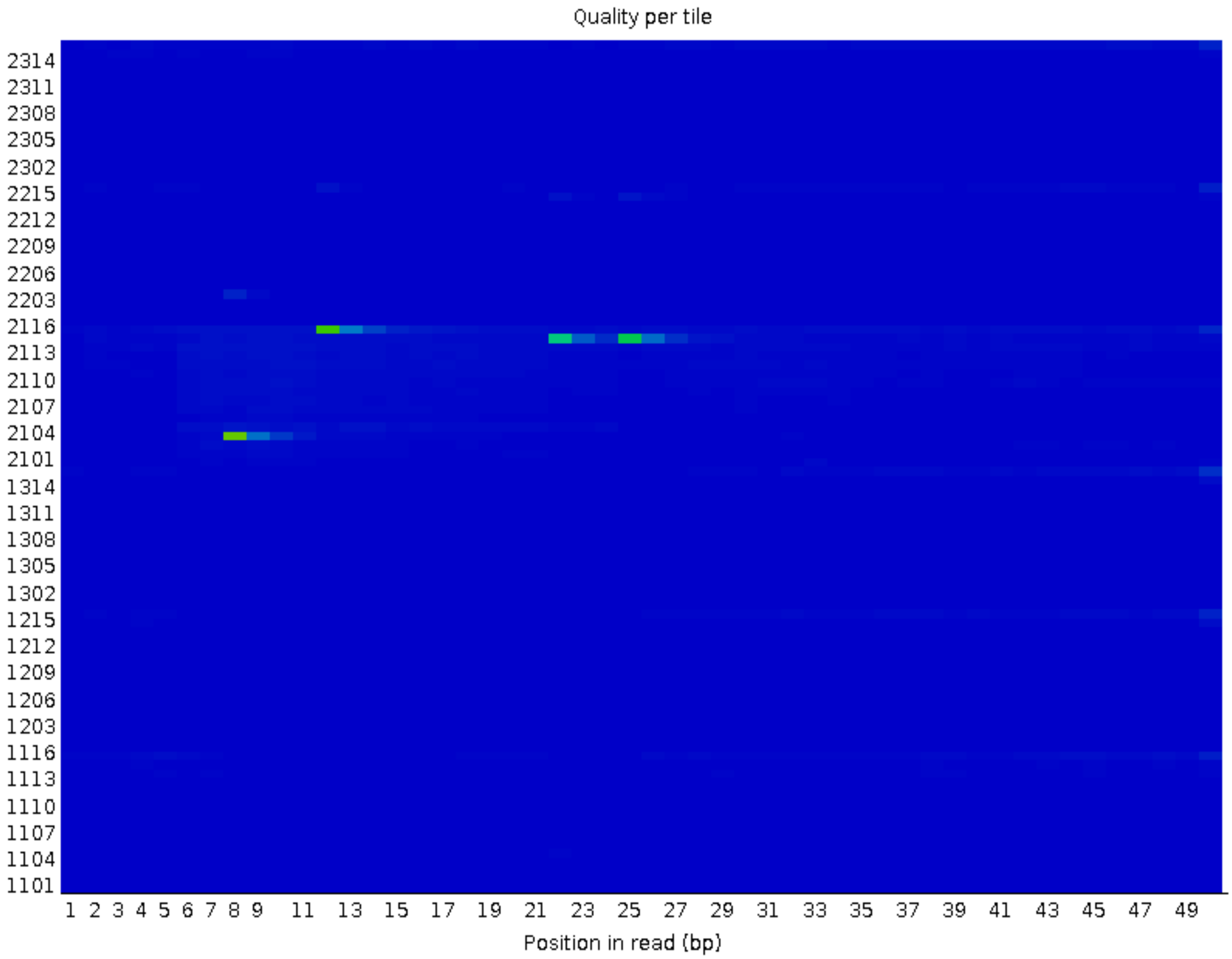

Per sequence quality scores

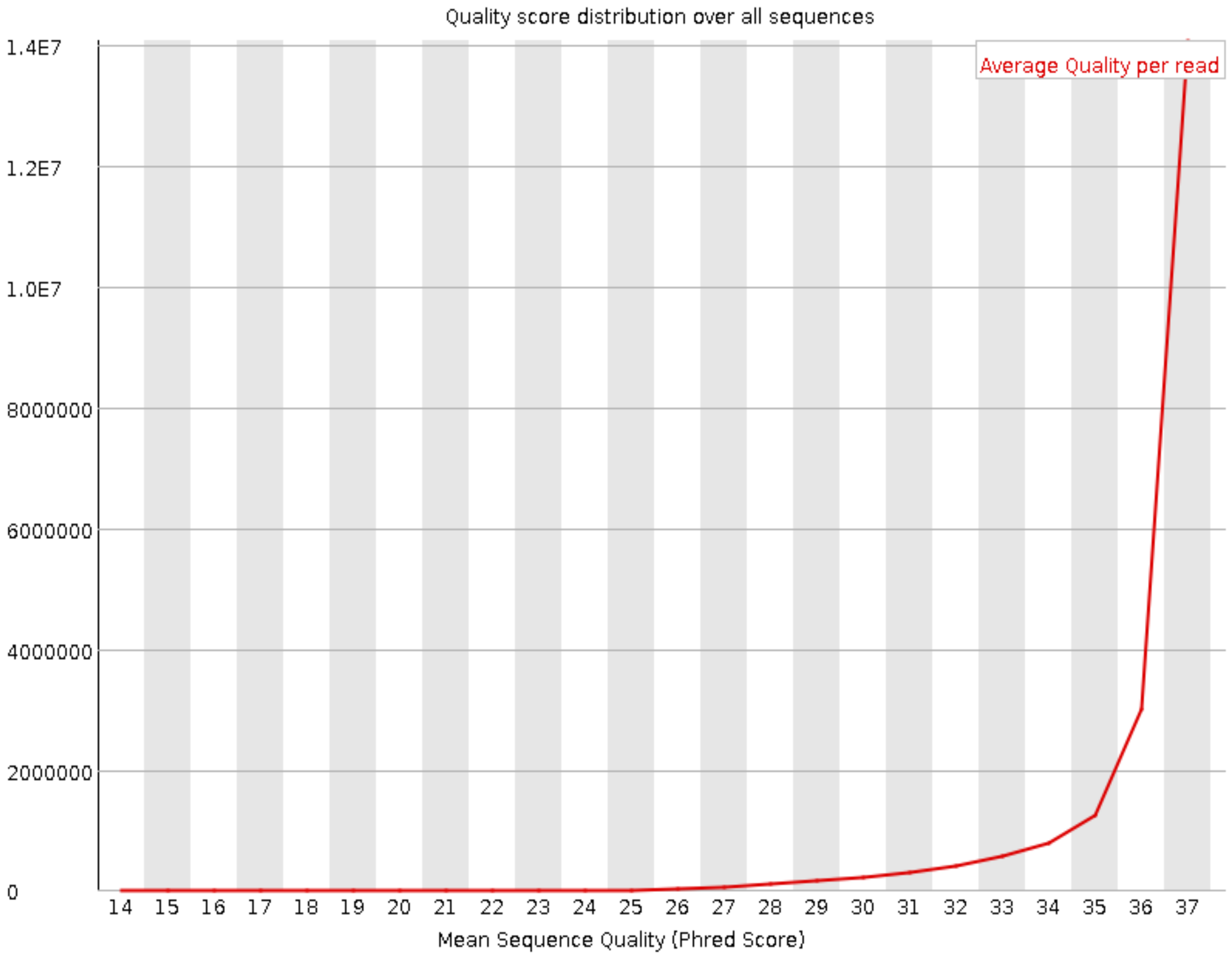

Per base sequence content

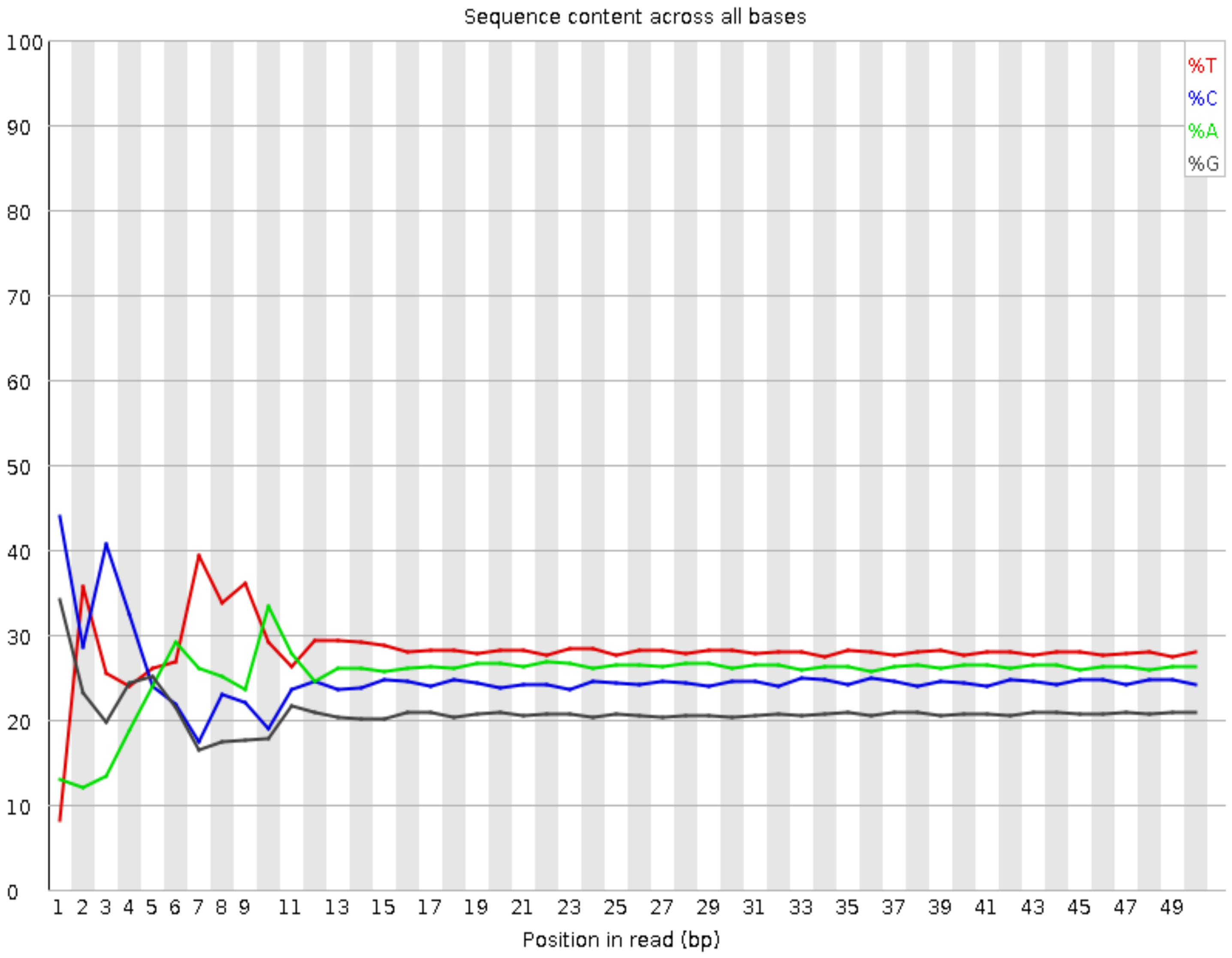

Per sequence GC content

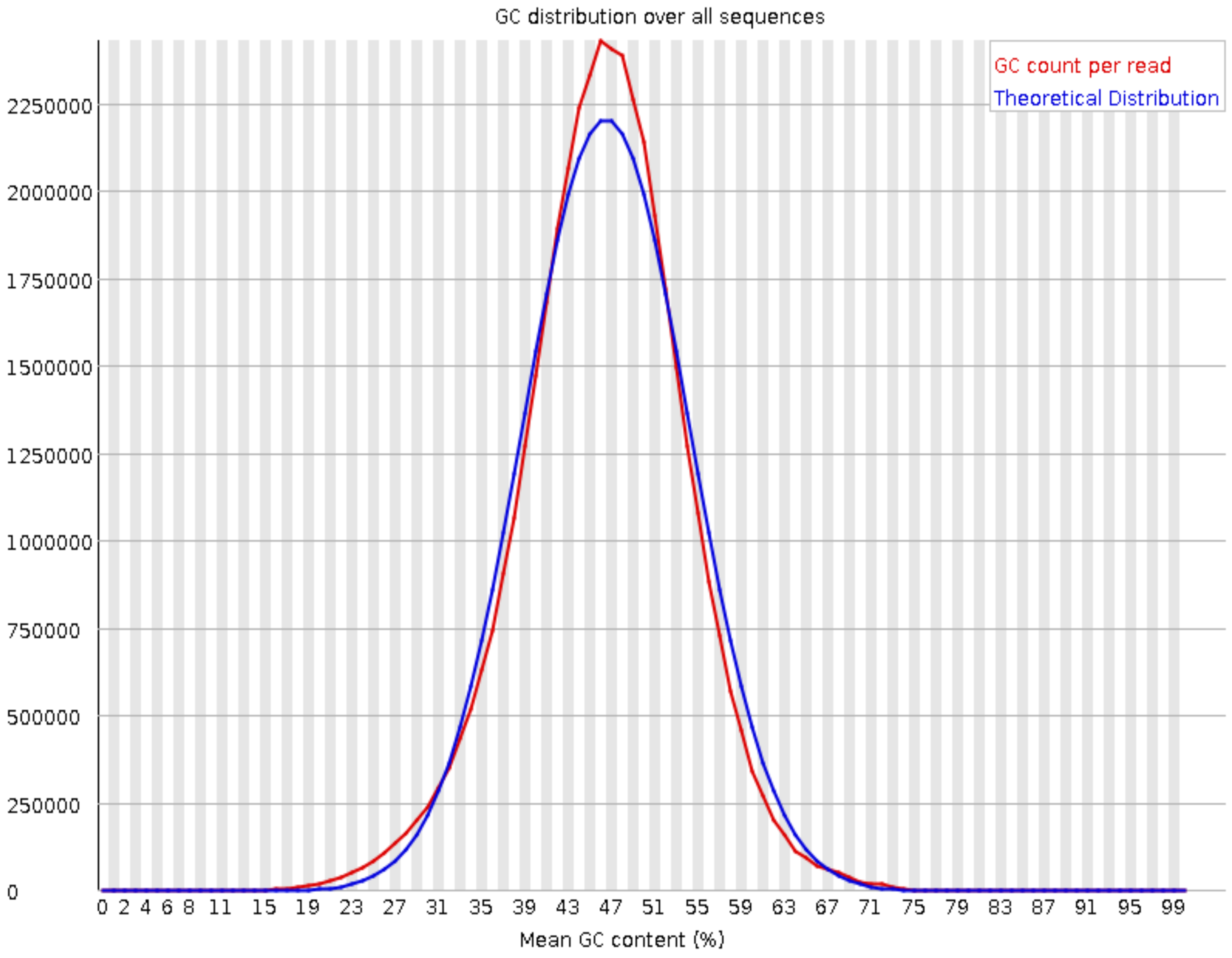

Per base N content

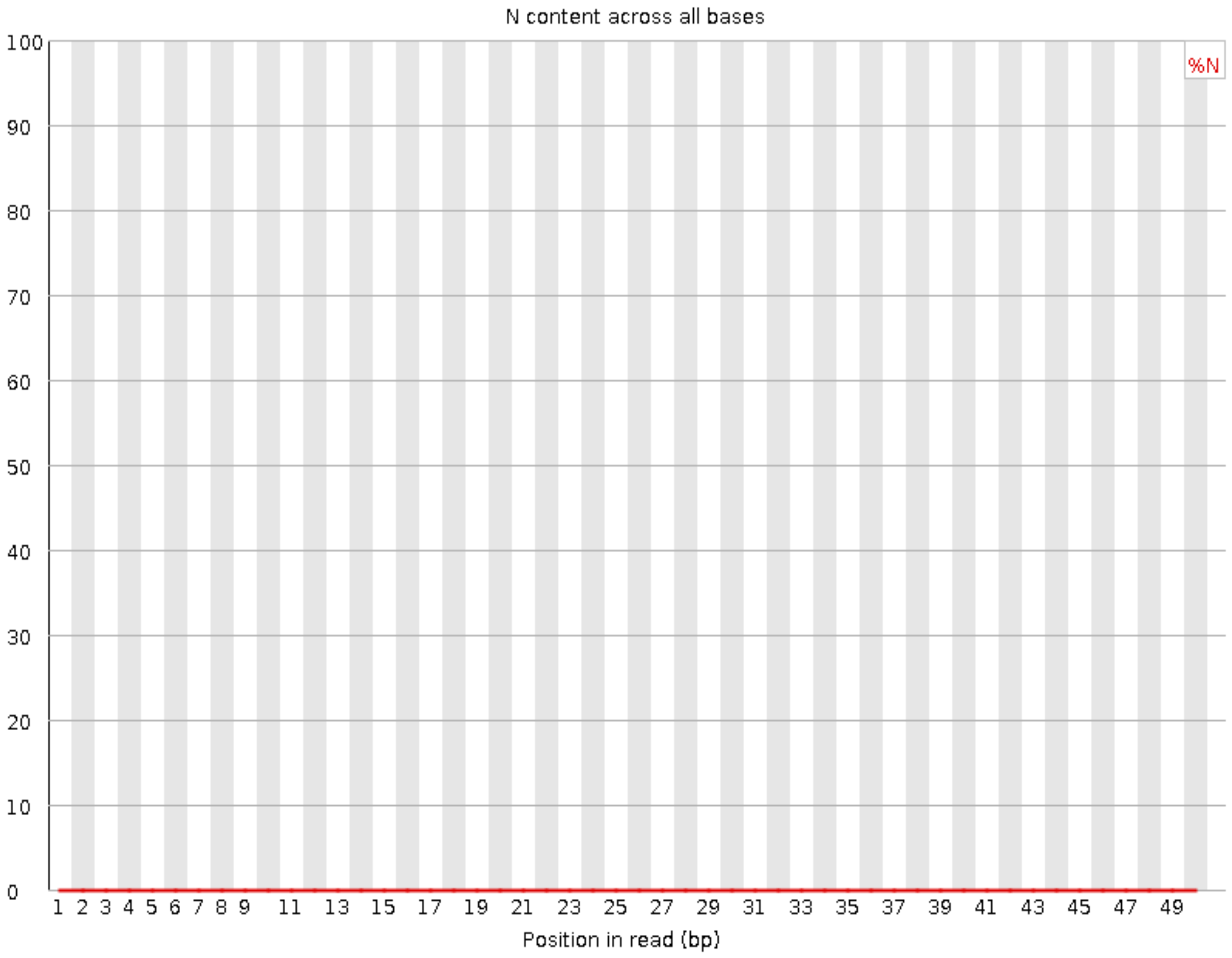

Sequence Length Distribution

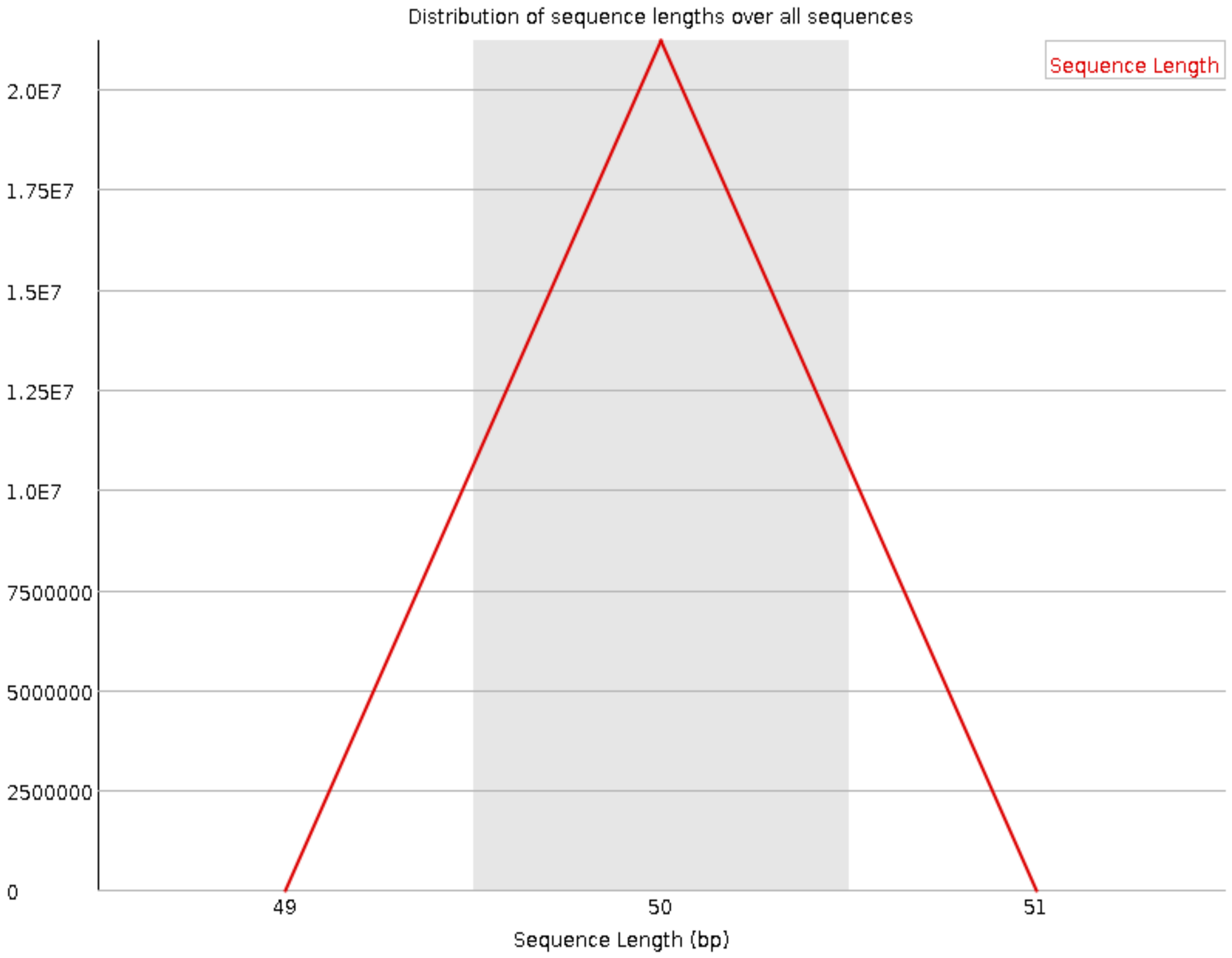

Sequence Duplication Levels

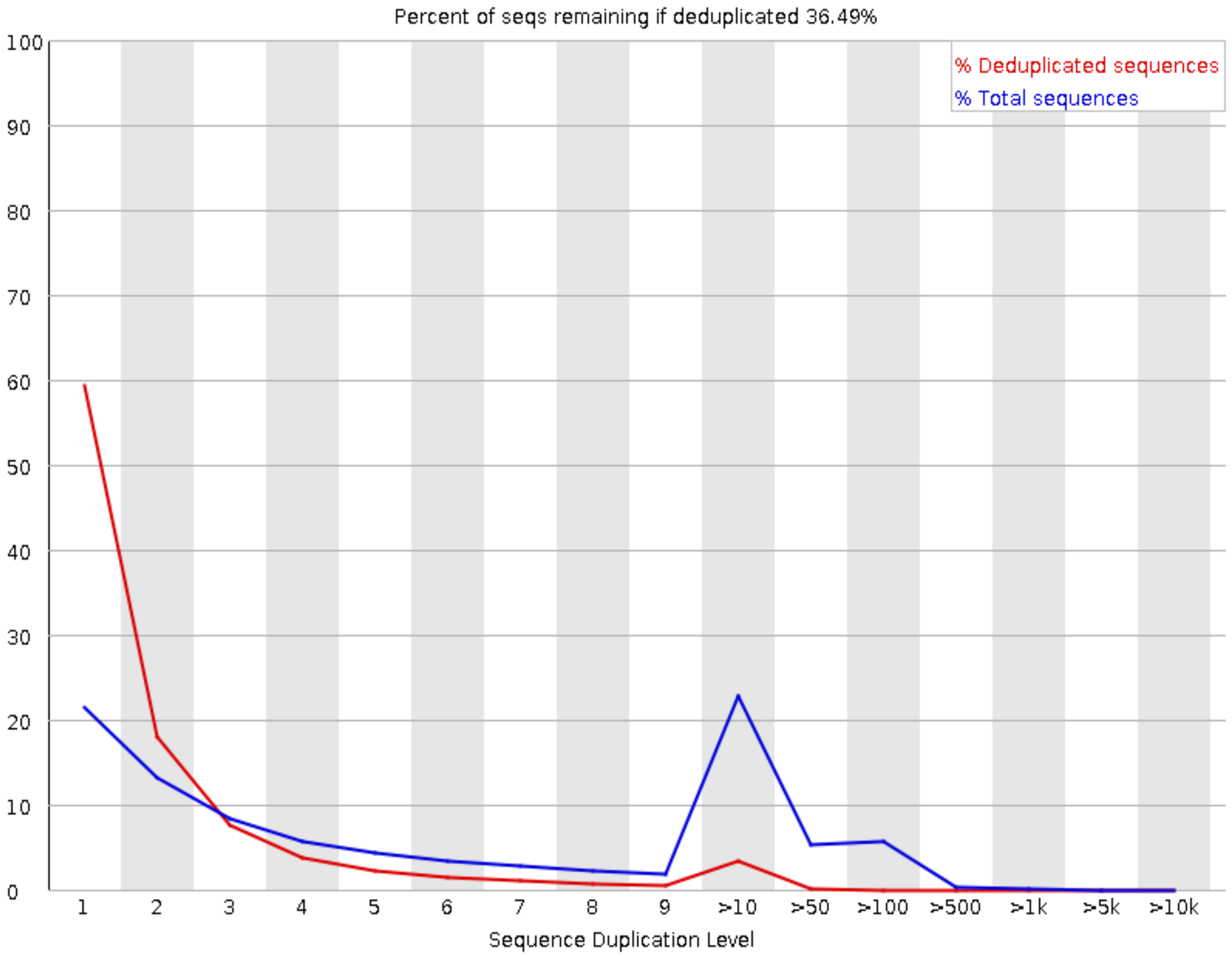

Overrepresented sequences

No overrepresented sequences

Adapter Content

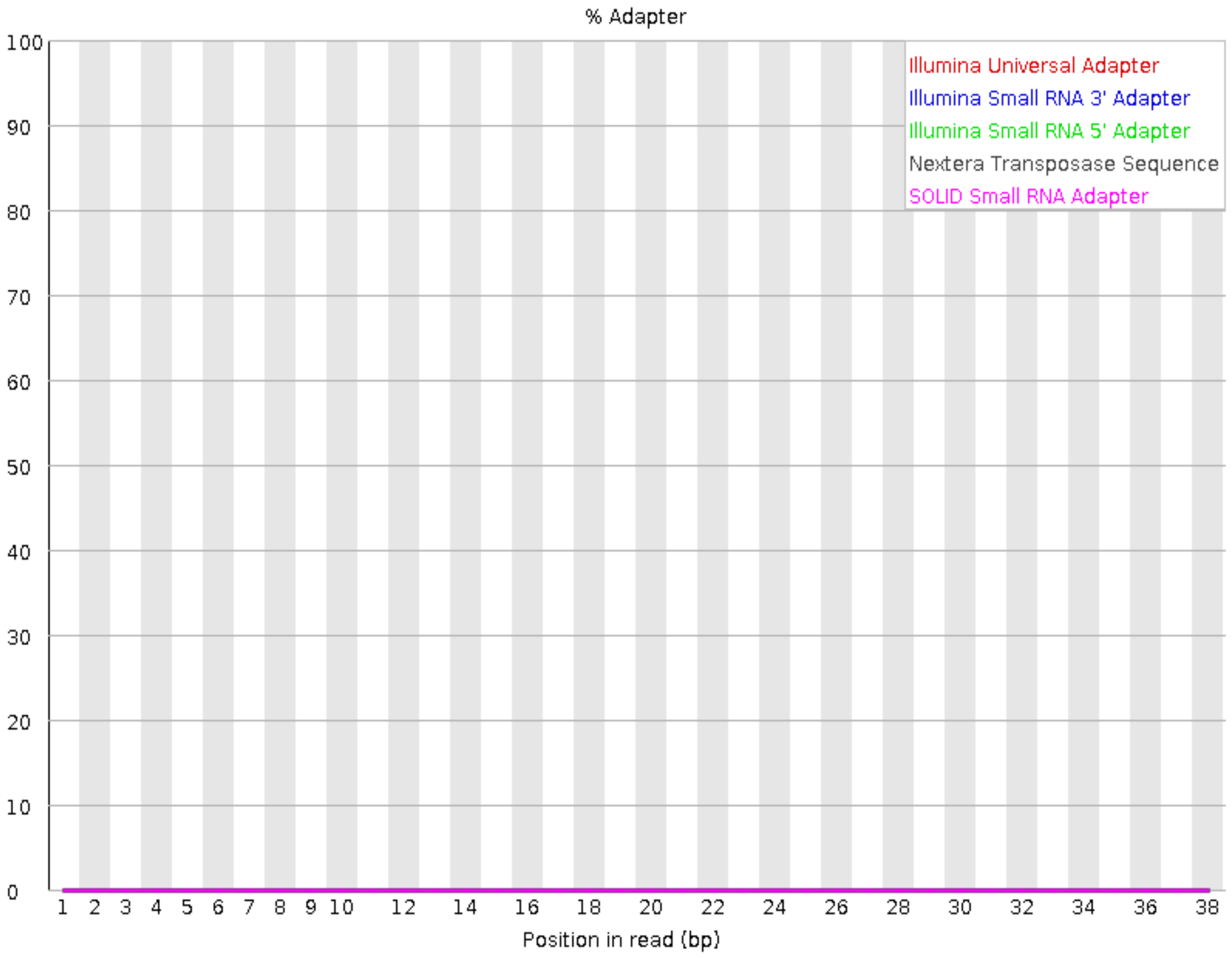

Kmer Content

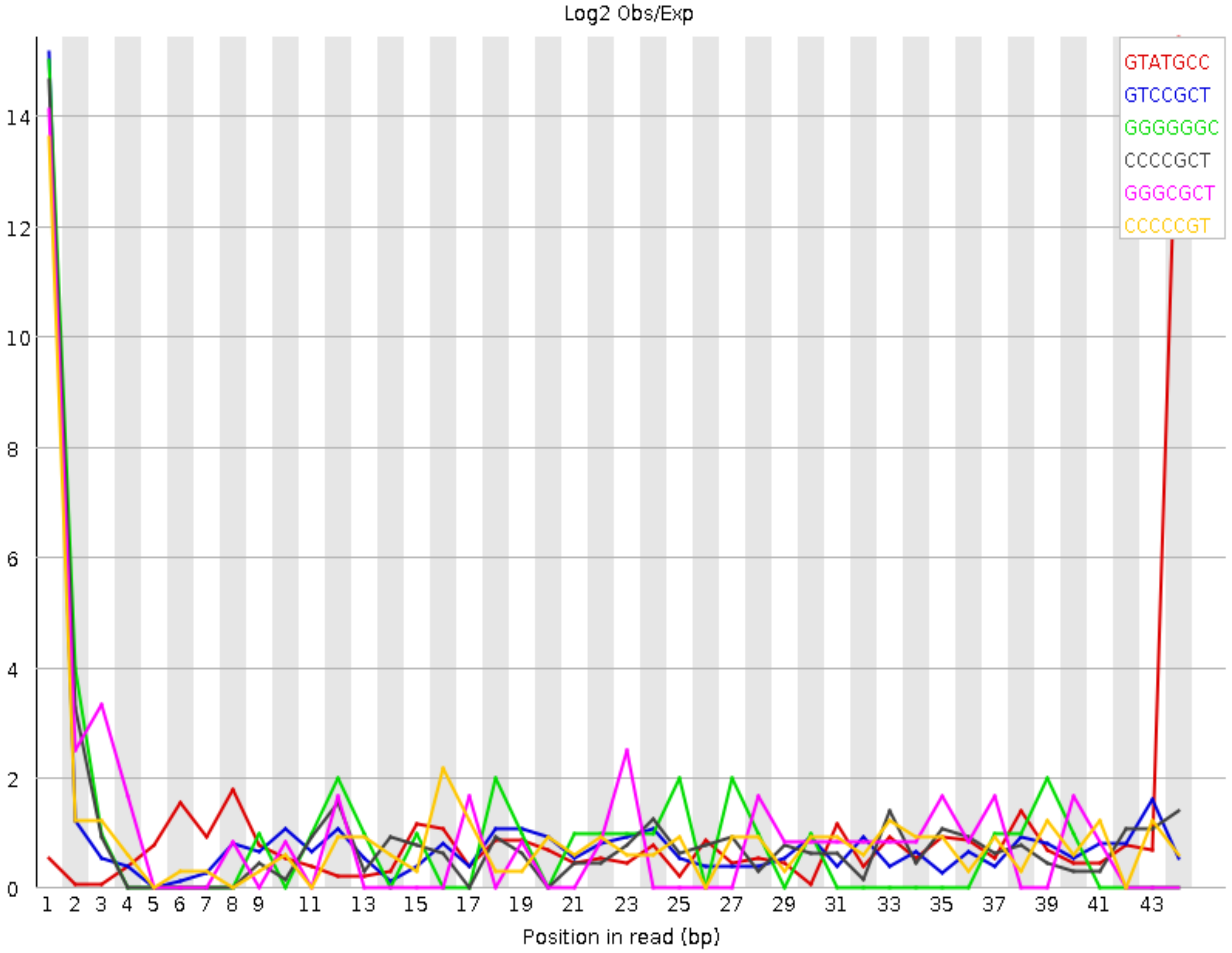

Sequence	Count	PValue	Obs/Exp Max	Max Obs/Exp Position
GTATGCC	2815	0.0	15.411333	44
GTCCGCT	1625	0.0	15.160766	1
GGGGGGC	220	3.074092E-10	14.997715	1
CCCCGCT	1410	0.0	14.664432	1
GGGCGCT	265	2.3646862E-11	14.111056	1
CCCCCGT	710	0.0	13.631725	1
CGTATGC	3210	0.0	13.583528	43
GCCCCTT	2225	0.0	12.753112	1
GGGCCCC	230	1.3251338E-7	12.432888	1
GCACACG	3710	0.0	12.212734	10
CCCCCCT	865	0.0	12.206232	1
GGGGTGT	1350	0.0	11.731545	1
CCCCGTT	1405	0.0	11.5854225	1

Sequence	Count	PValue	Obs/Exp Max	Max Obs/Exp Position
CGGGGCG	210	8.141367E-6	11.522053	1
CCCCGGT	1565	0.0	11.384846	1
CCCCCTT	2225	0.0	11.17133	1
TCGTATG	4035	0.0	11.024429	42
GGGGCCT	925	0.0	10.701072	1
GTCCGTT	4445	0.0	10.49109	1
GGGGGCT	1765	0.0	10.344031	1

Produced by FastQC (version 0.11.5)

FastQC Report

Tue 12 Sep 2017
22006_TAGCTT_L001_R1.fastq.gz

Summary

- Basic Statistics
- Per base sequence quality
- Per tile sequence quality
- Per sequence quality scores
- Per base sequence content
- Per sequence GC content
- Per base N content
- Sequence Length Distribution
- Sequence Duplication Levels
- Overrepresented sequences
- Adapter Content
- Kmer Content

Basic Statistics

Measure	Value
Filename	22006_TAGCTT_L001_R1_001.fastq.gz
File type	Conventional base calls
Encoding	Sanger / Illumina 1.9
Total Sequences	24947175
Sequences flagged as poor quality	0
Sequence length	50
%GC	46

Per base sequence quality

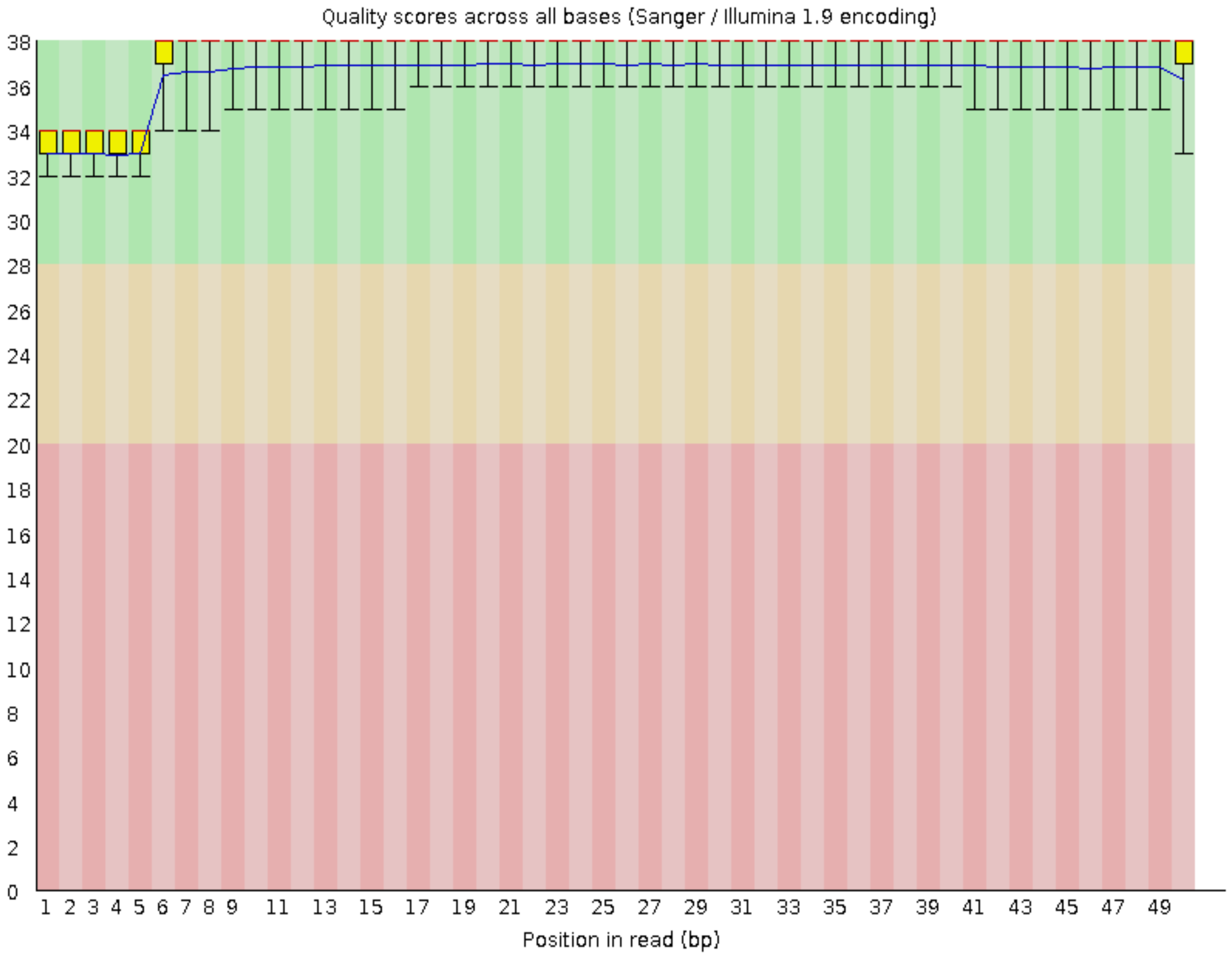

Per tile sequence quality

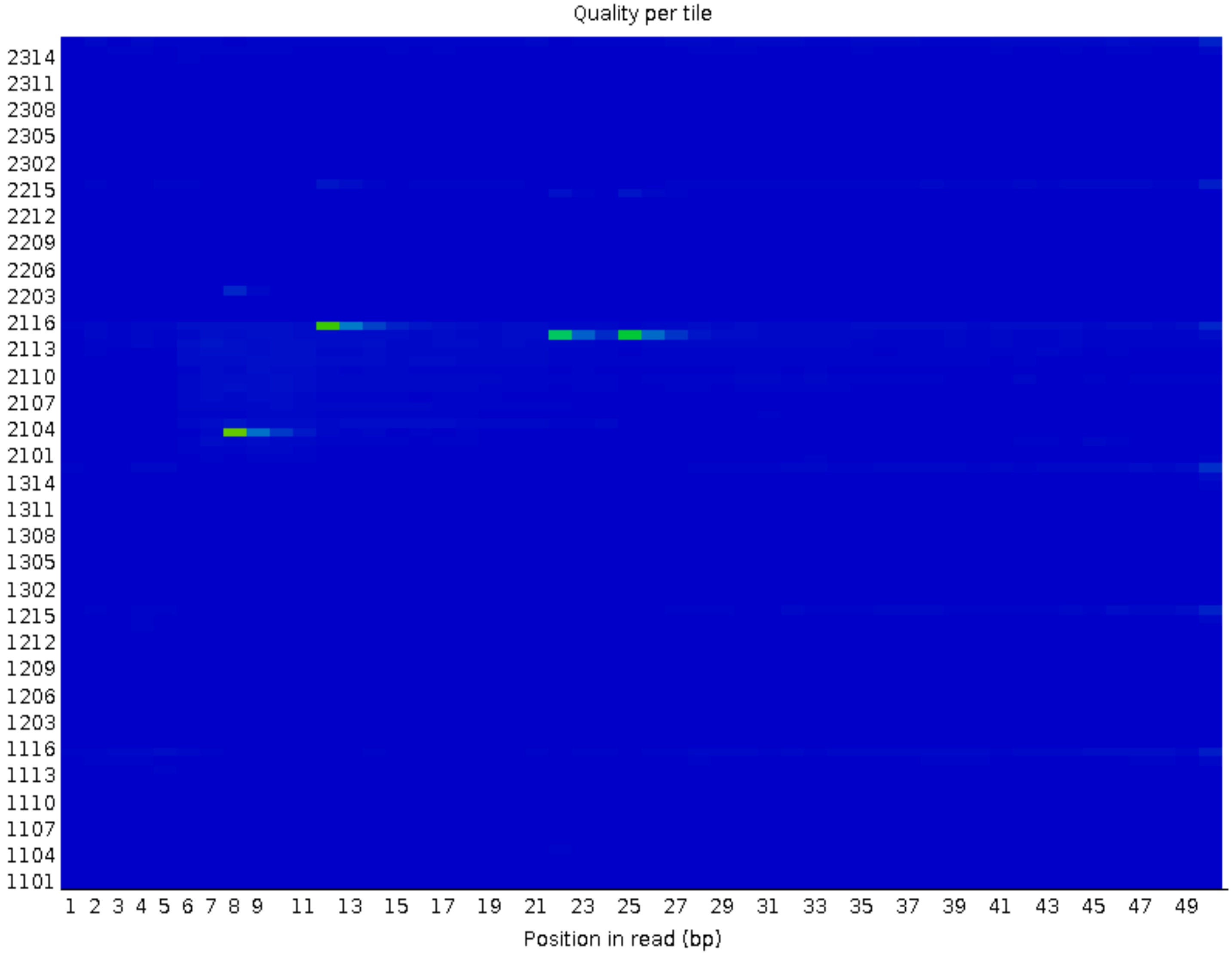

Per sequence quality scores

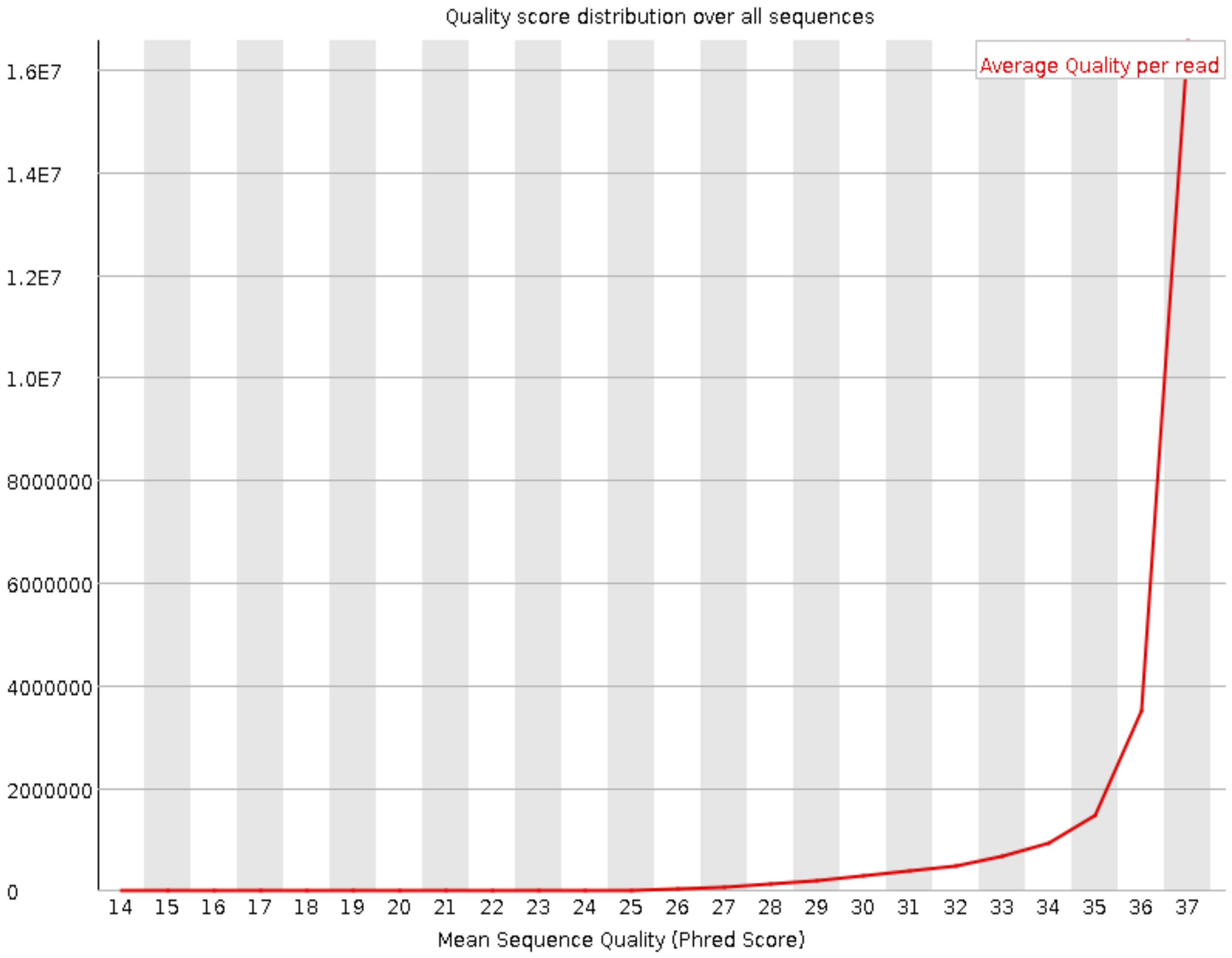

Per base sequence content

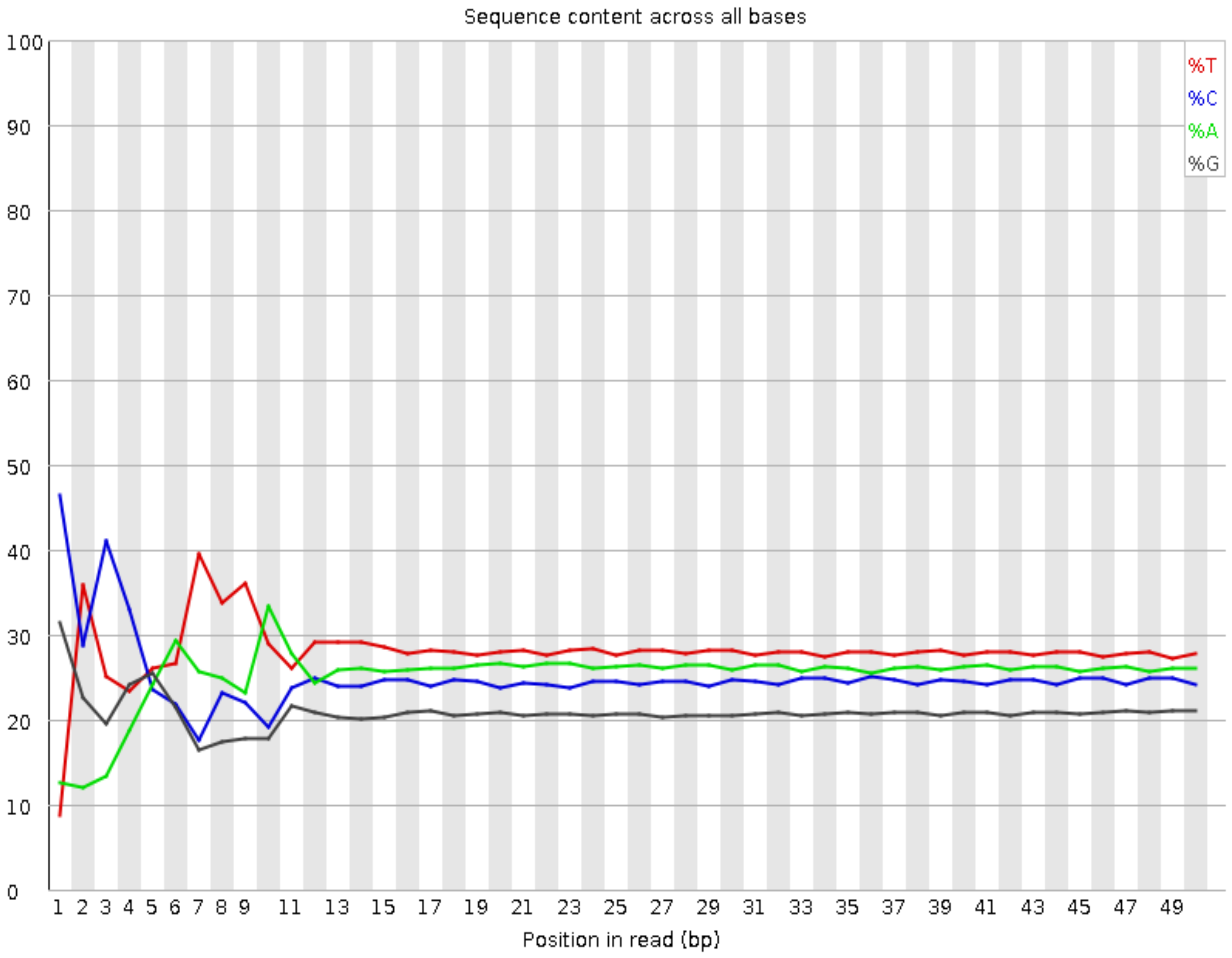

Per sequence GC content

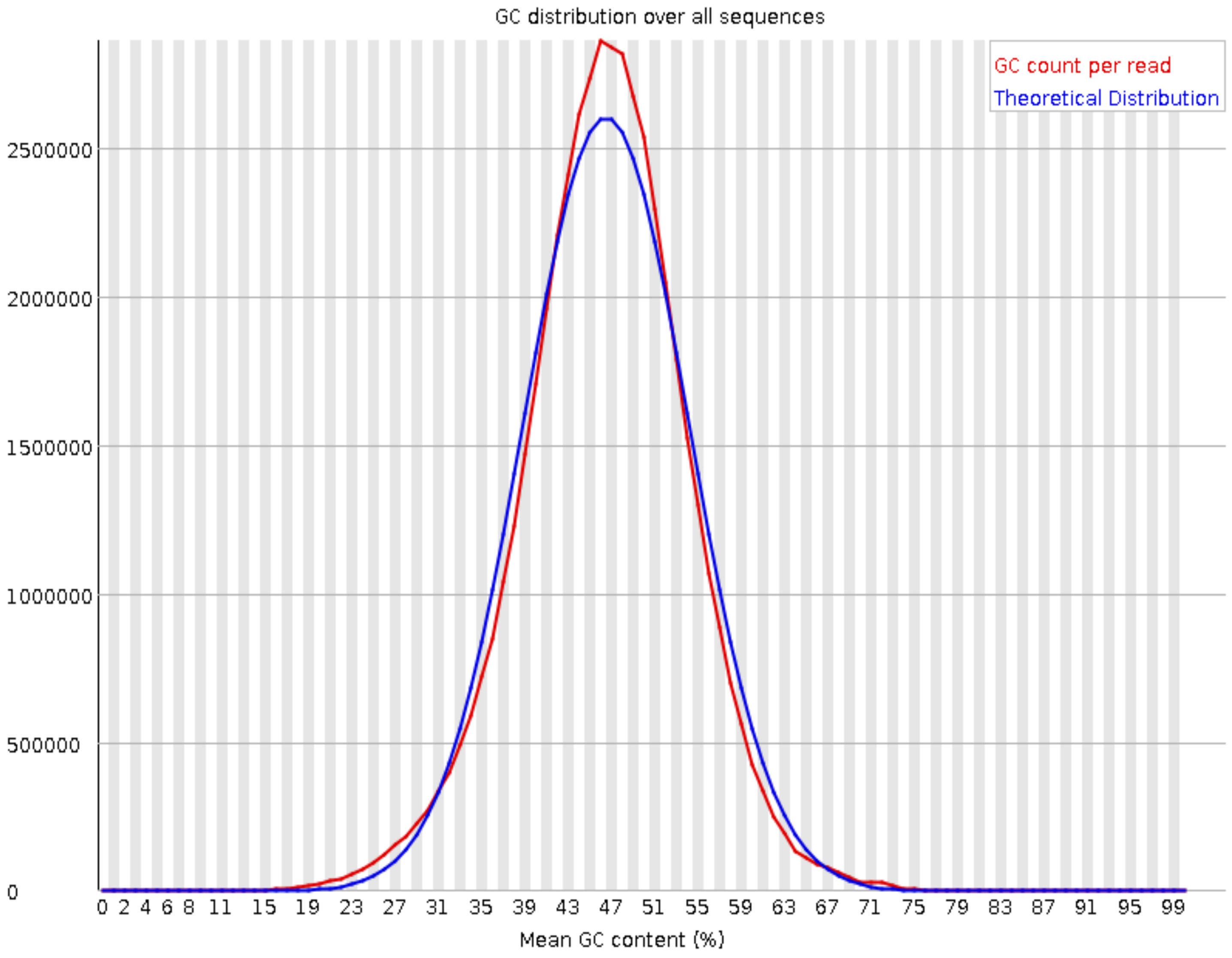

Per base N content

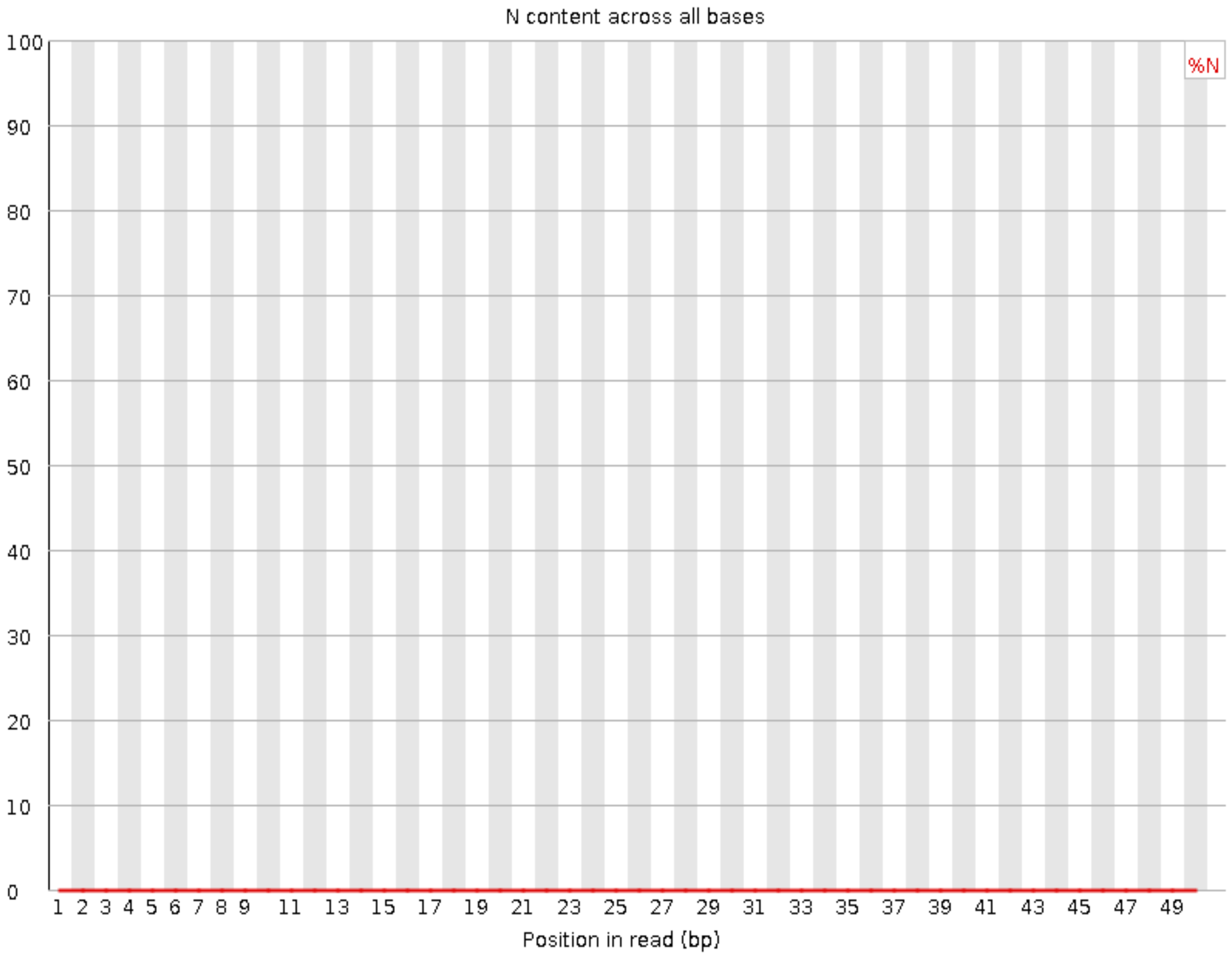

Sequence Length Distribution

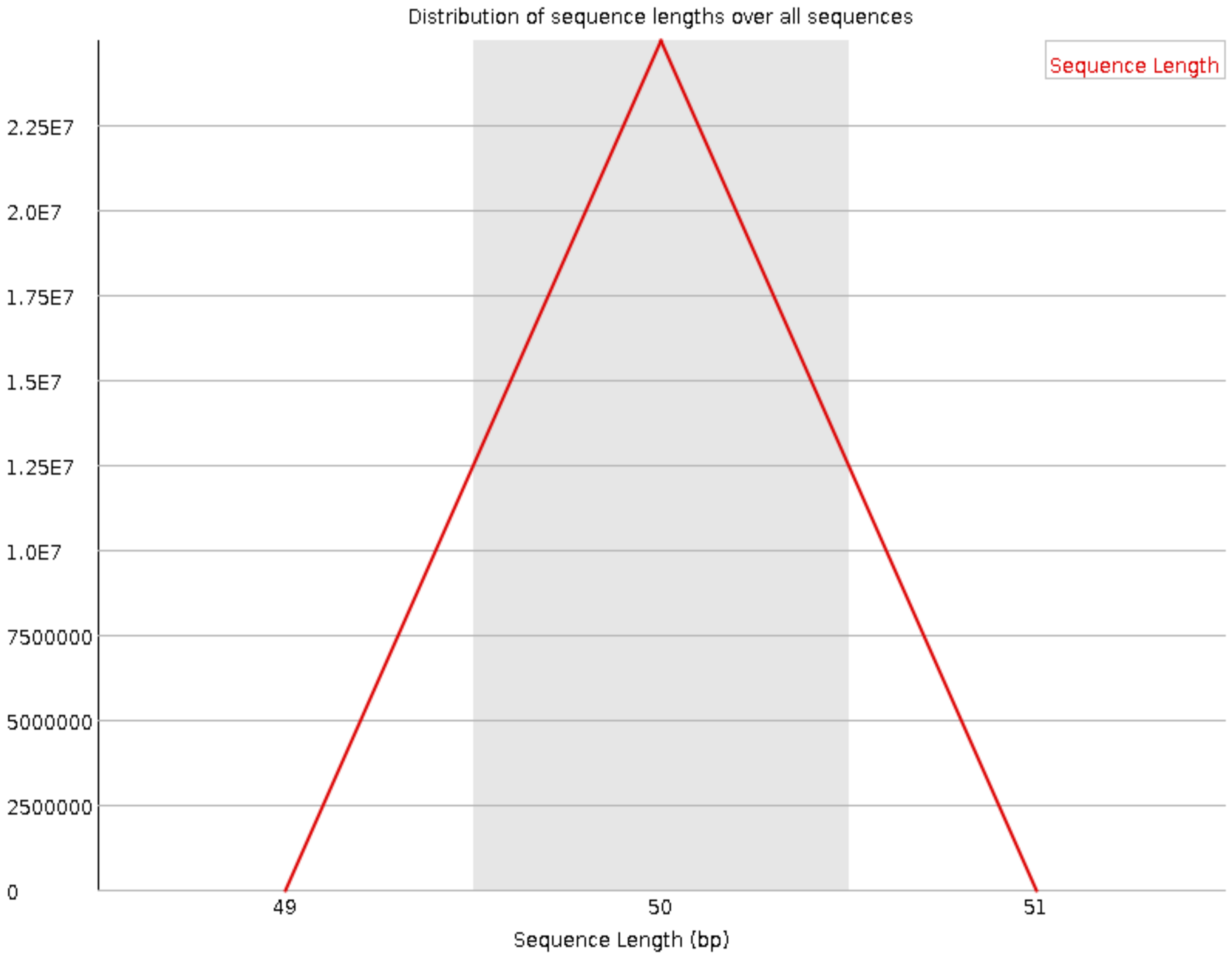

Sequence Duplication Levels

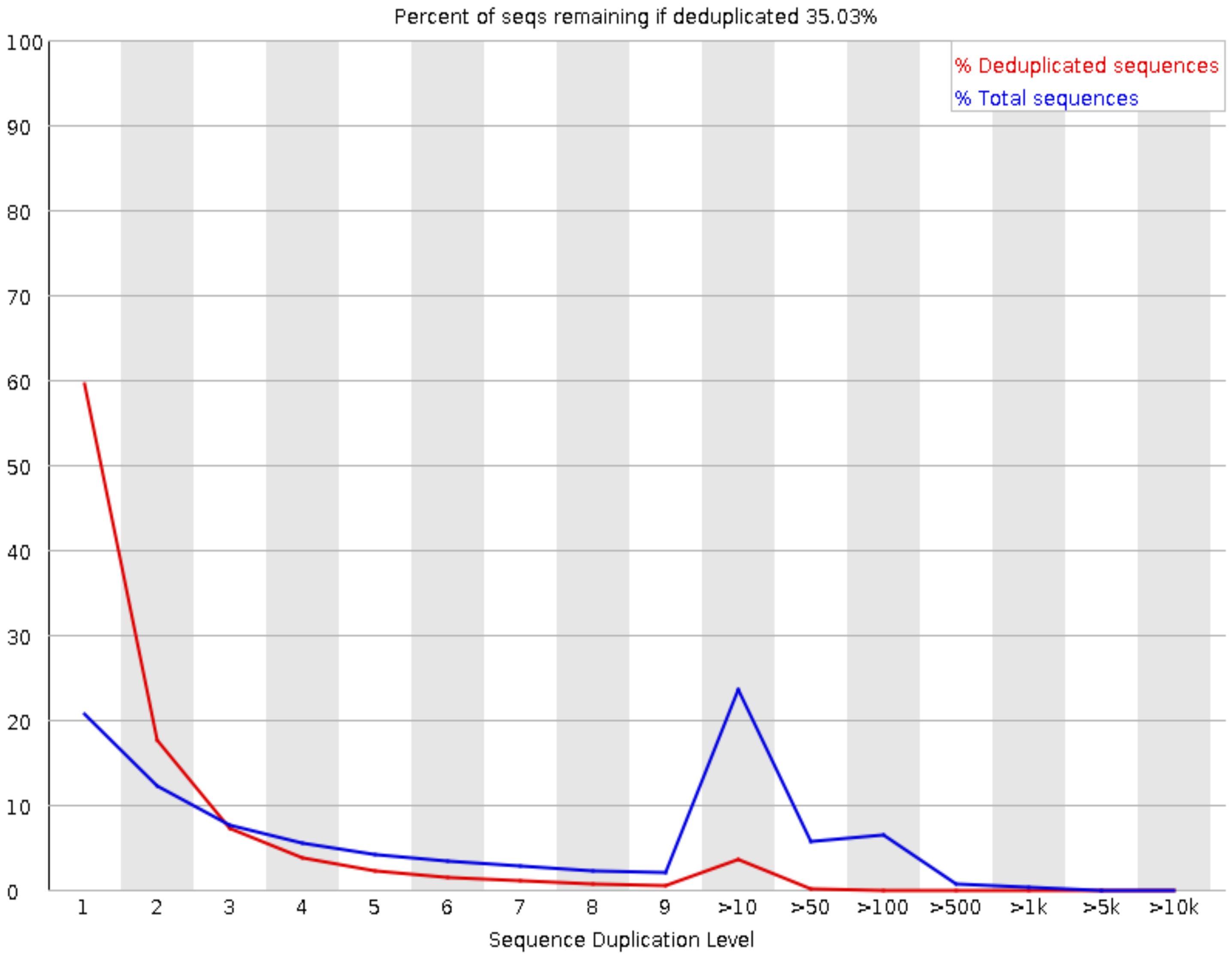

Overrepresented sequences

No overrepresented sequences

Adapter Content

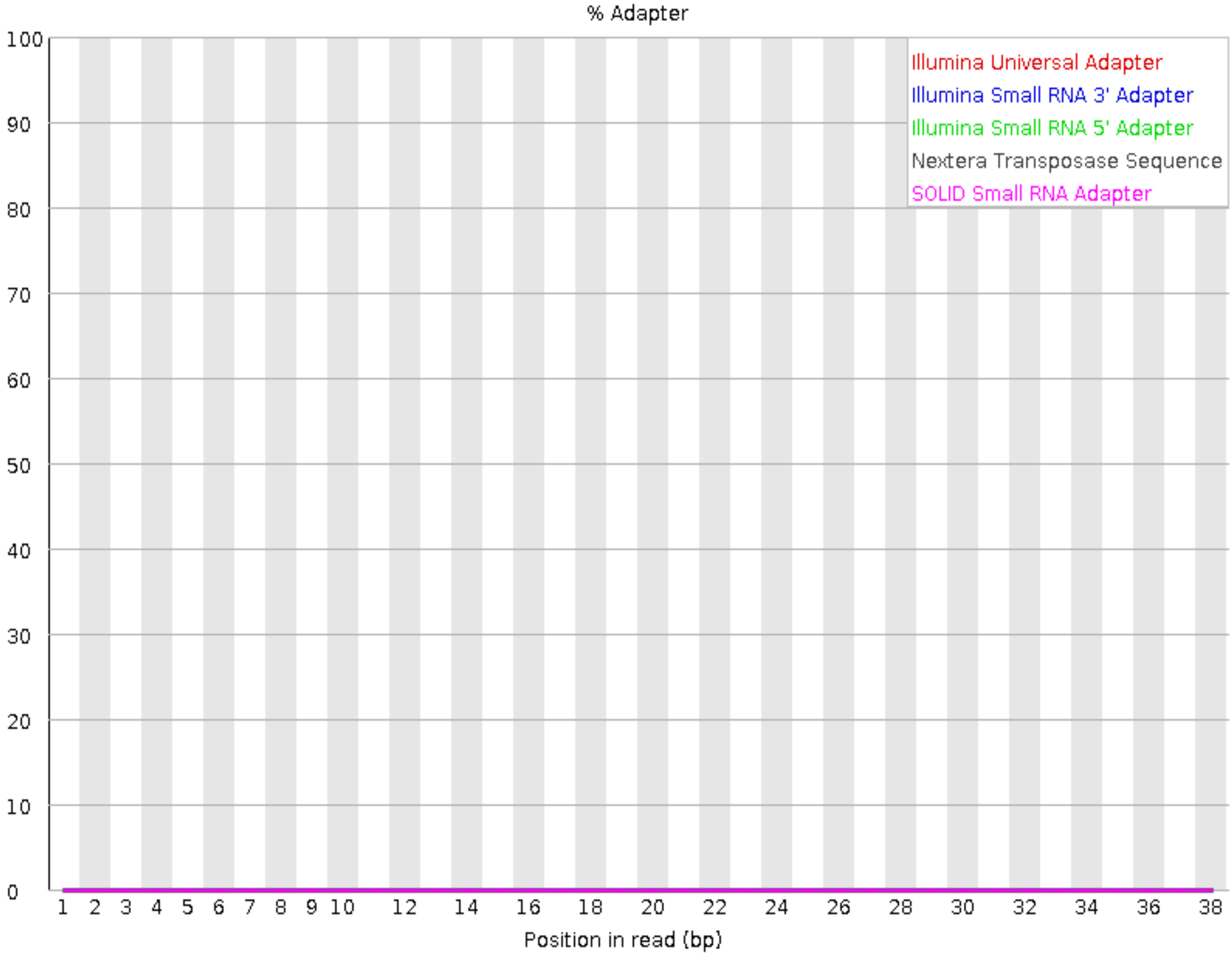

Kmer Content

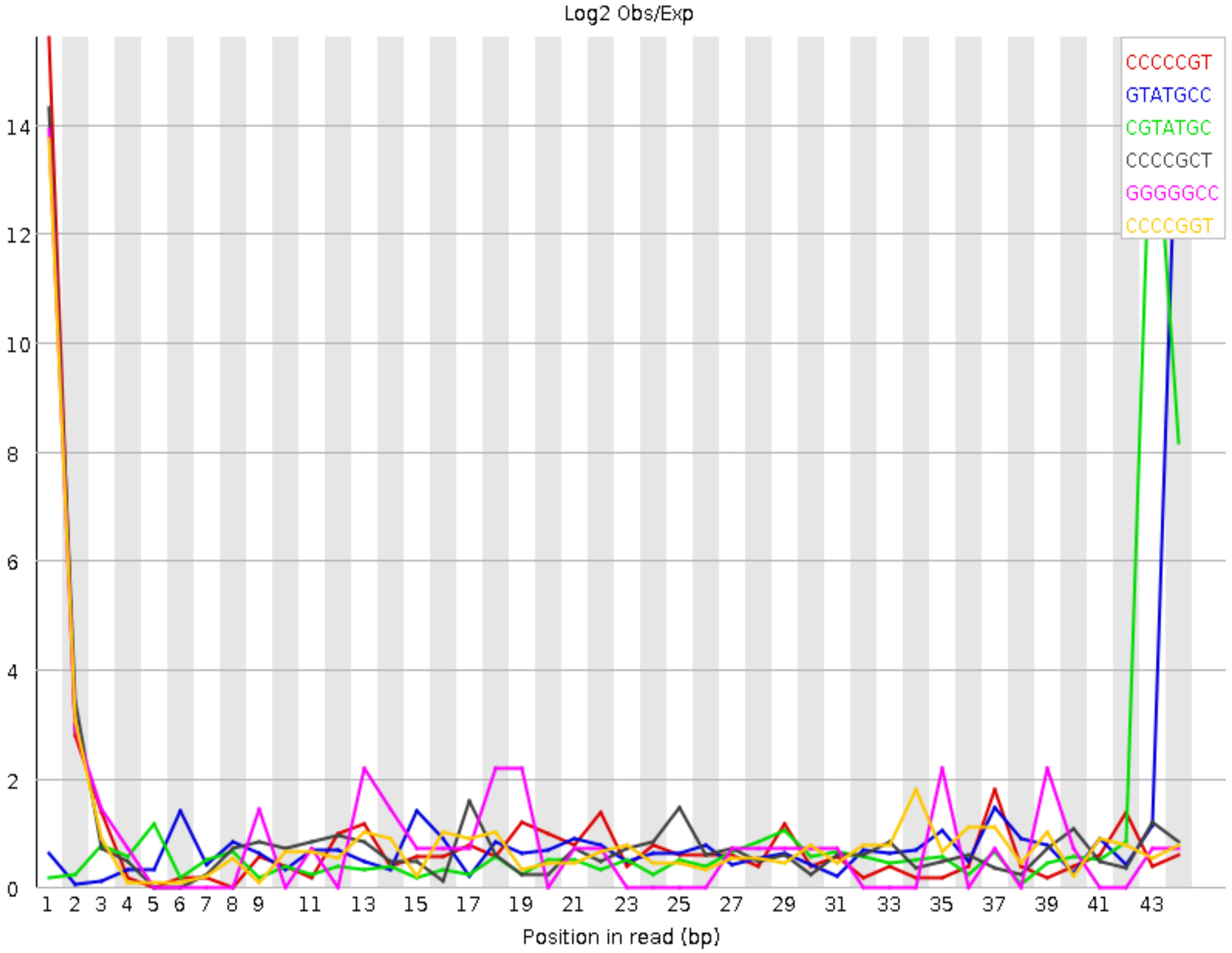

Sequence	Count	PValue	Obs/Exp Max	Max Obs/Exp Position
CCCCCGT	1100	0.0	15.597489	1
GTATGCC	3090	0.0	15.537191	44
CGTATGC	3320	0.0	15.190495	43
CCCCGCT	1795	0.0	14.337525	1
GGGGGCC	300	1.8189894E-12	13.93109	1
CCCCGGT	1935	0.0	13.75489	1
CCCCCCT	885	0.0	13.670115	1
GCACACG	3955	0.0	12.623579	10
CCCCCTT	2790	0.0	11.983734	1
GTCCGCT	1925	0.0	11.883801	1
TCGTATG	4350	0.0	11.542969	42
GGGGGAT	1625	0.0	11.370478	1
GTCCGAT	3450	0.0	11.348897	1

Sequence	Count	PValue	Obs/Exp Max	Max Obs/Exp Position
CCCCCAT	3810	0.0	11.25803	1
CCCCTGT	3385	0.0	10.981984	1
CCCCTCT	4410	0.0	10.923411	1
GGGGGGT	545	0.0	10.897327	1
GGGGGCT	1735	0.0	10.776363	1
CCCGGCT	3940	0.0	10.719086	1
ACGTCTG	4765	0.0	10.662546	14

Produced by FastQC (version 0.11.5)

FastQC Report

Tue 12 Sep 2017
22007_GGCTAC_L001_R1.fastq.gz

Summary

- Basic Statistics
- Per base sequence quality
- Per tile sequence quality
- Per sequence quality scores
- Per base sequence content
- Per sequence GC content
- Per base N content
- Sequence Length Distribution
- Sequence Duplication Levels
- Overrepresented sequences
- Adapter Content
- Kmer Content

Basic Statistics

Measure	Value
Filename	22007_GGCTAC_L001_R1_001.fastq.gz
File type	Conventional base calls
Encoding	Sanger / Illumina 1.9
Total Sequences	23359464
Sequences flagged as poor quality	0
Sequence length	50
%GC	46

Per base sequence quality

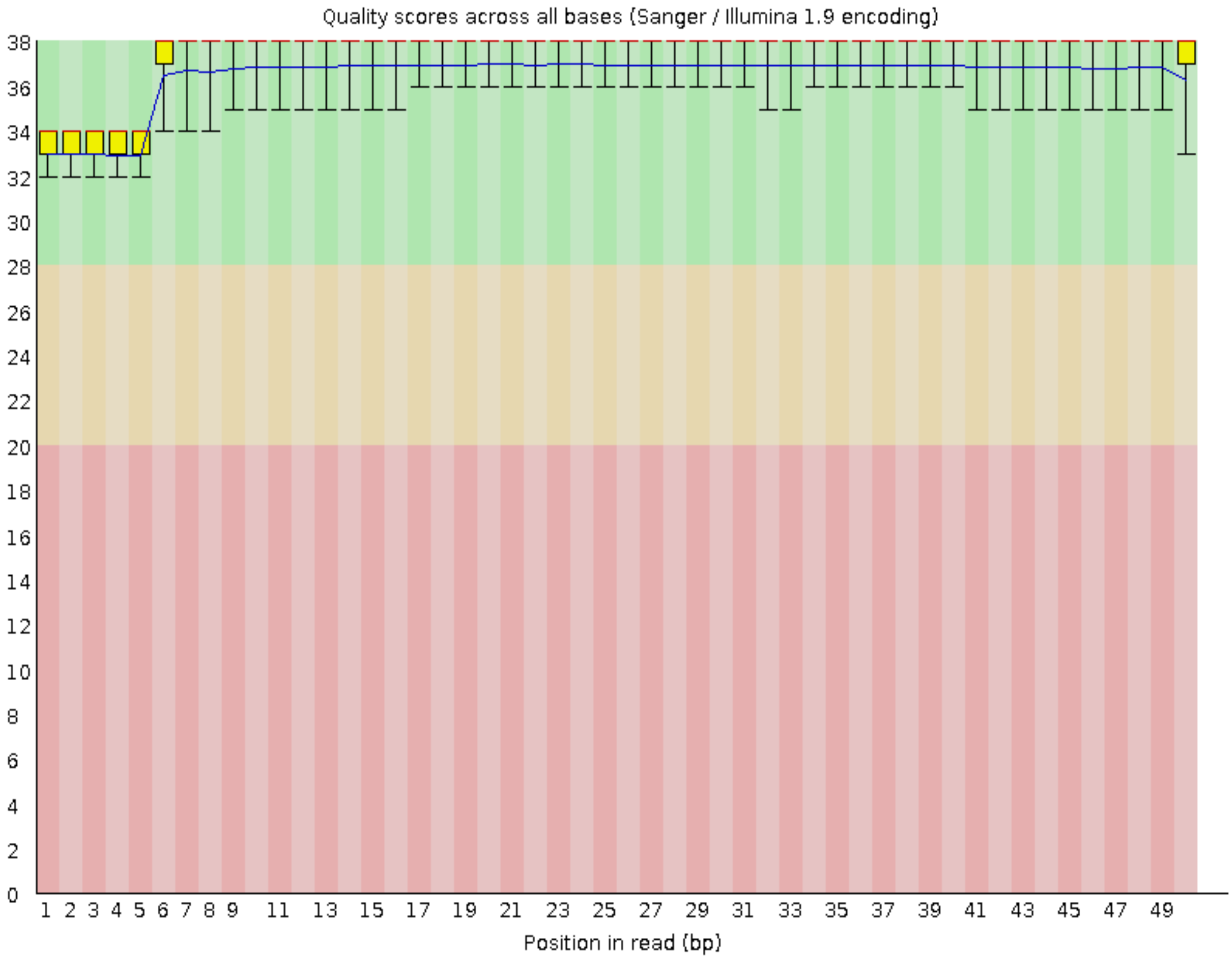

Per tile sequence quality

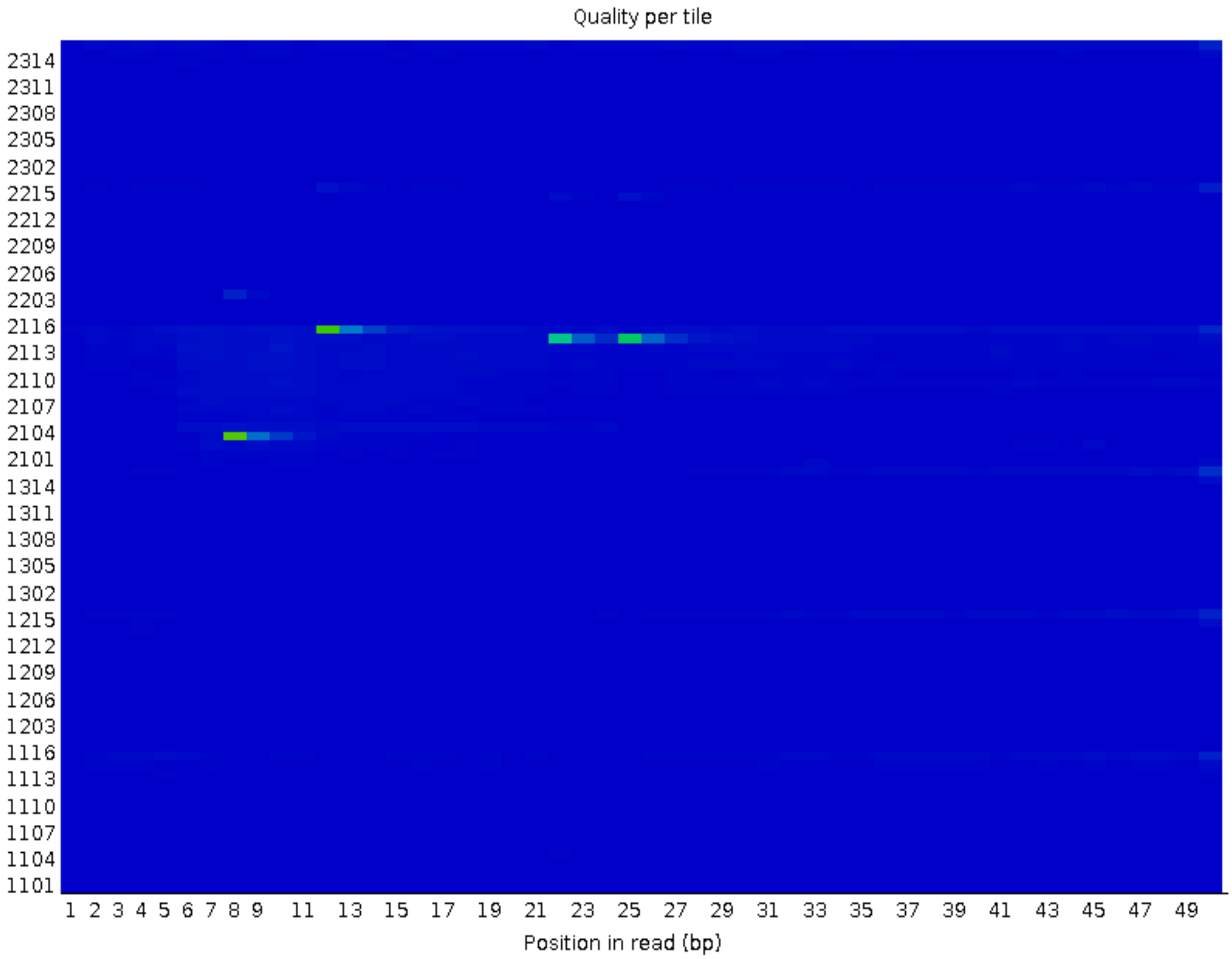

Per sequence quality scores

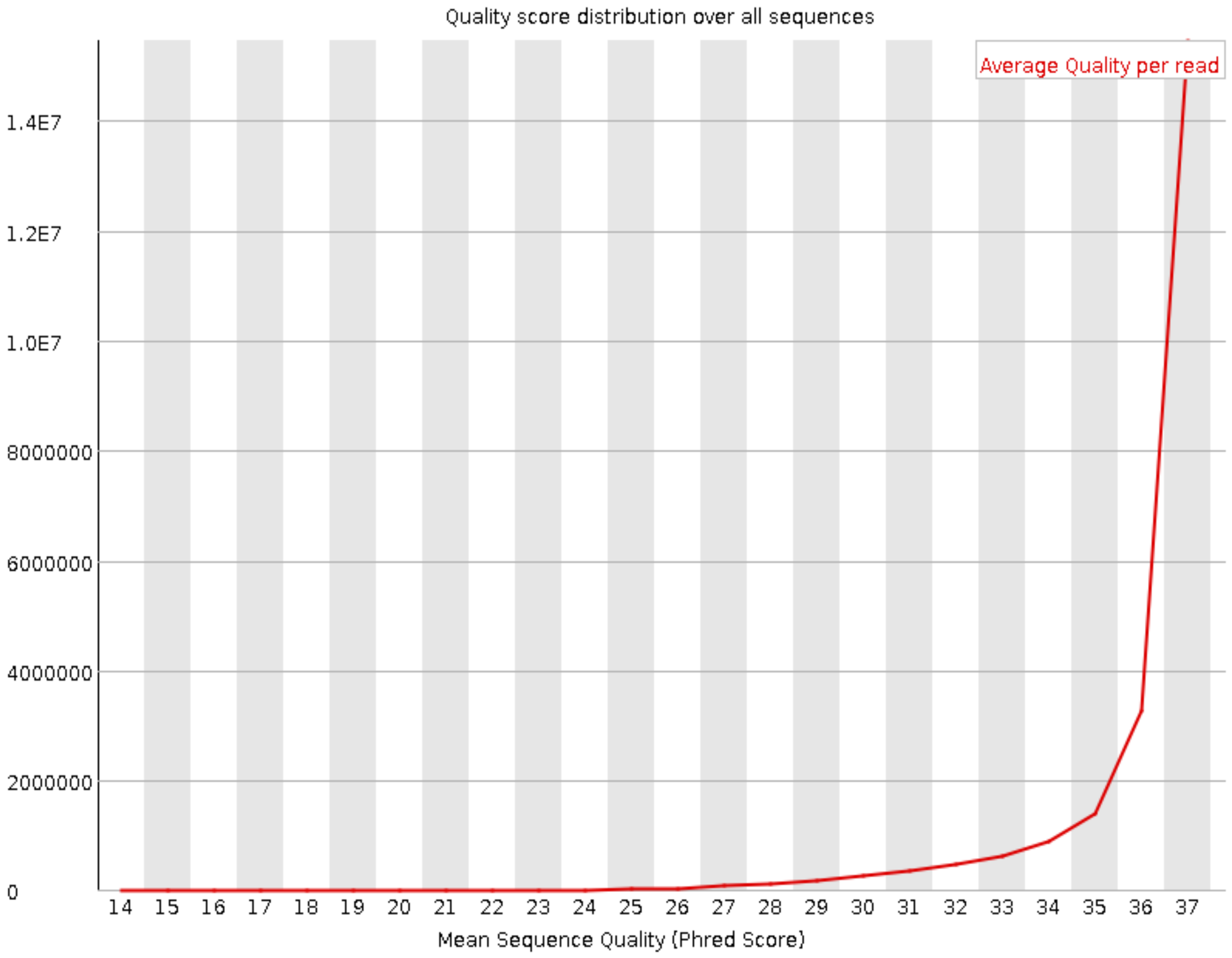

Per base sequence content

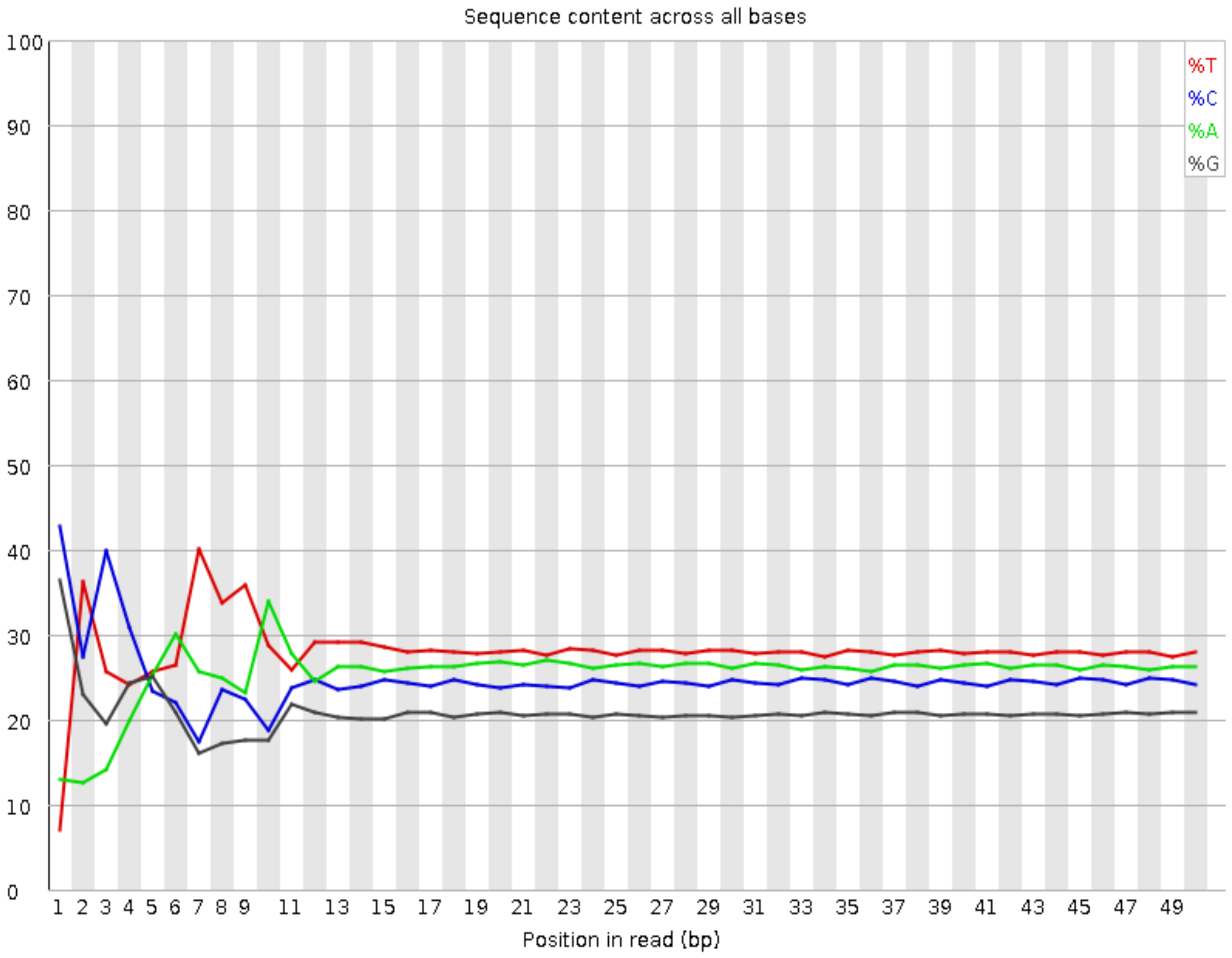

Per sequence GC content

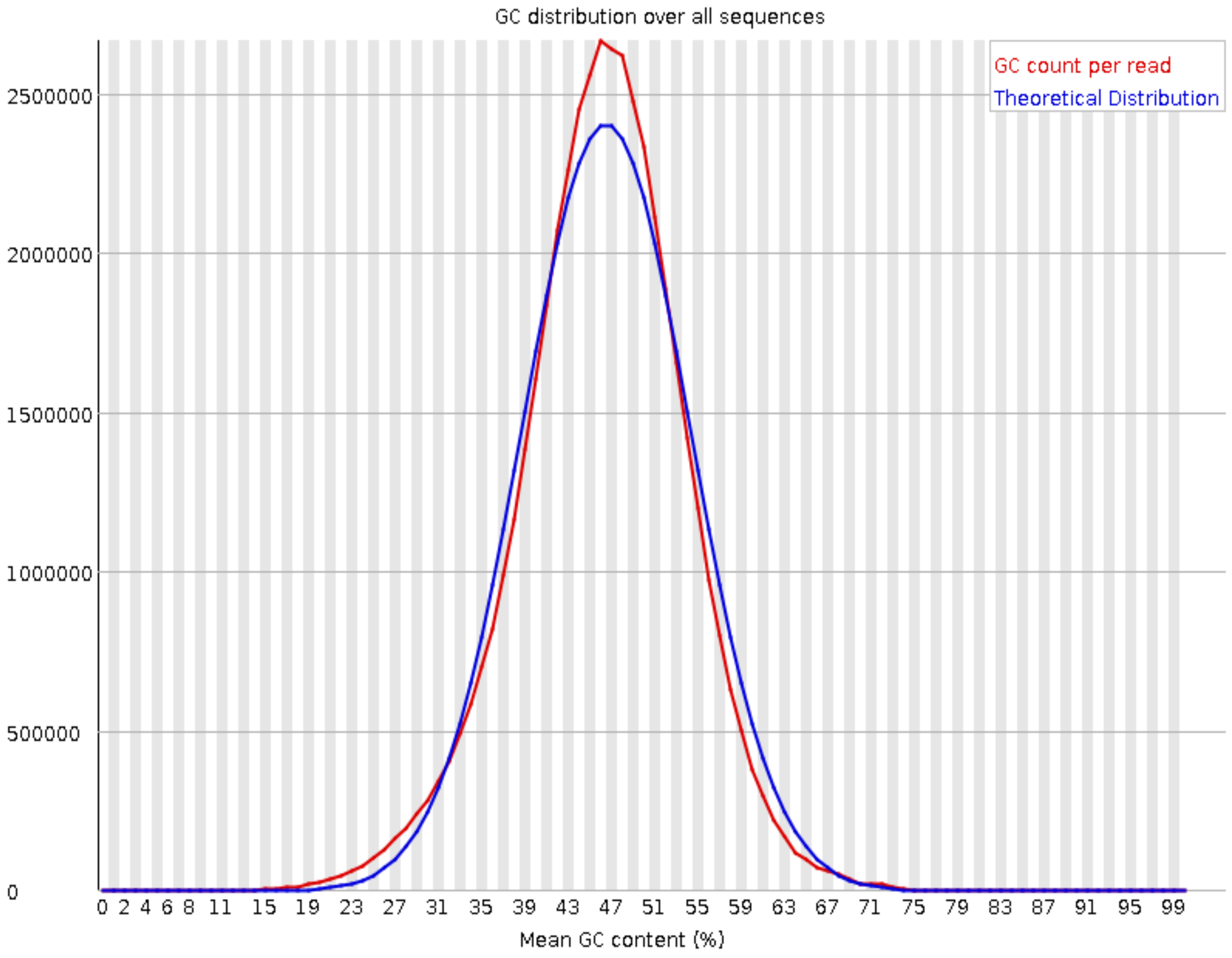

Per base N content

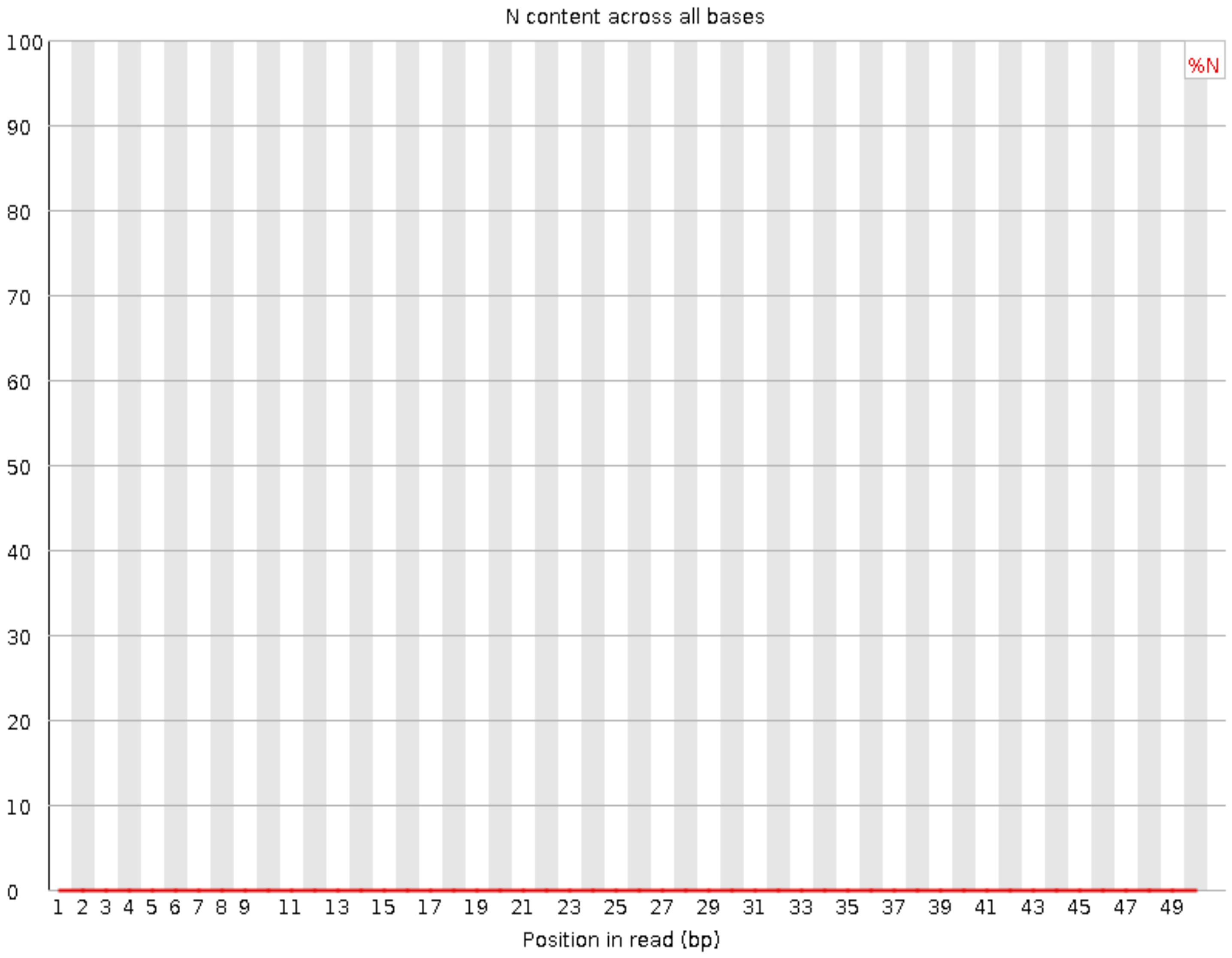

Sequence Length Distribution

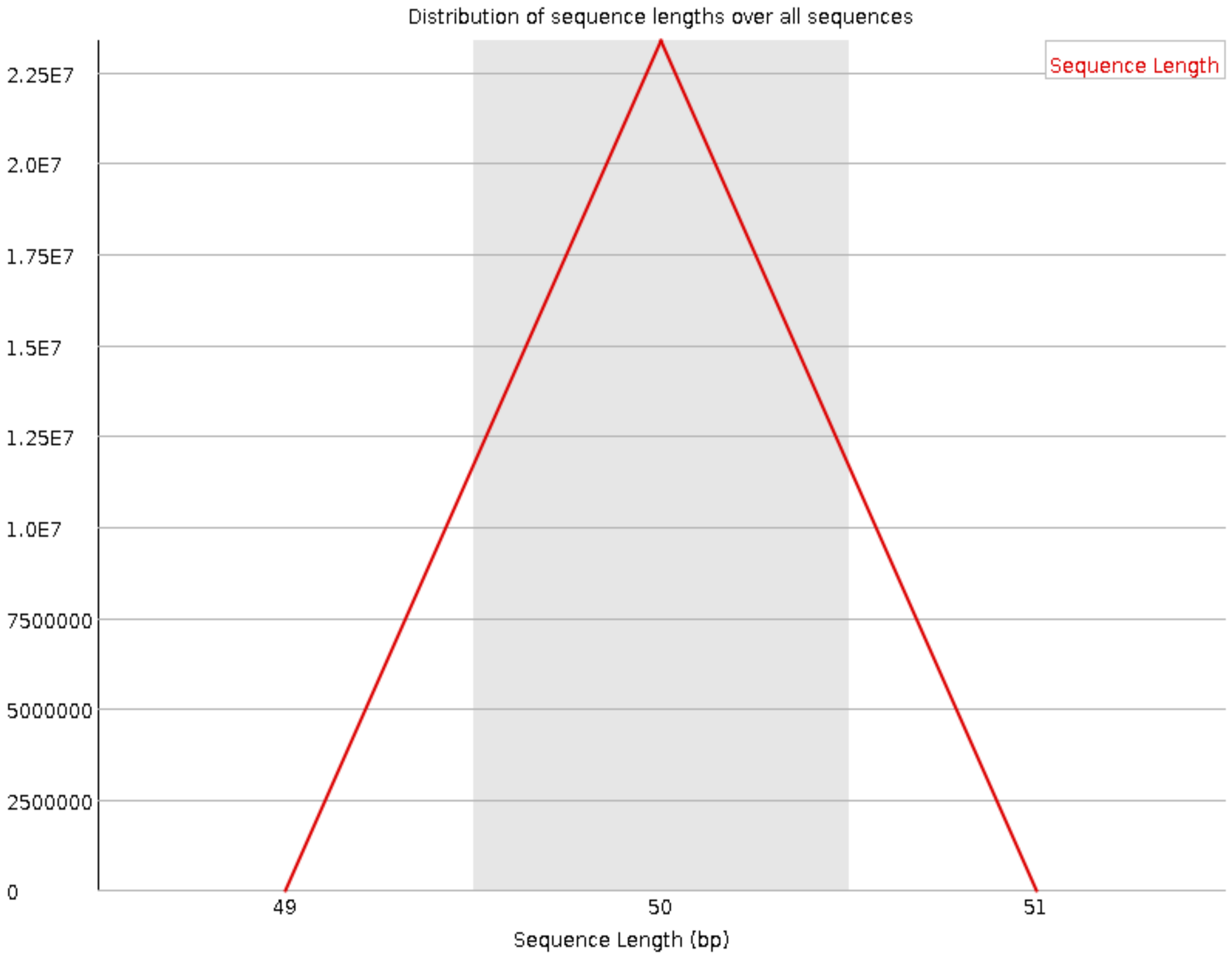

Sequence Duplication Levels

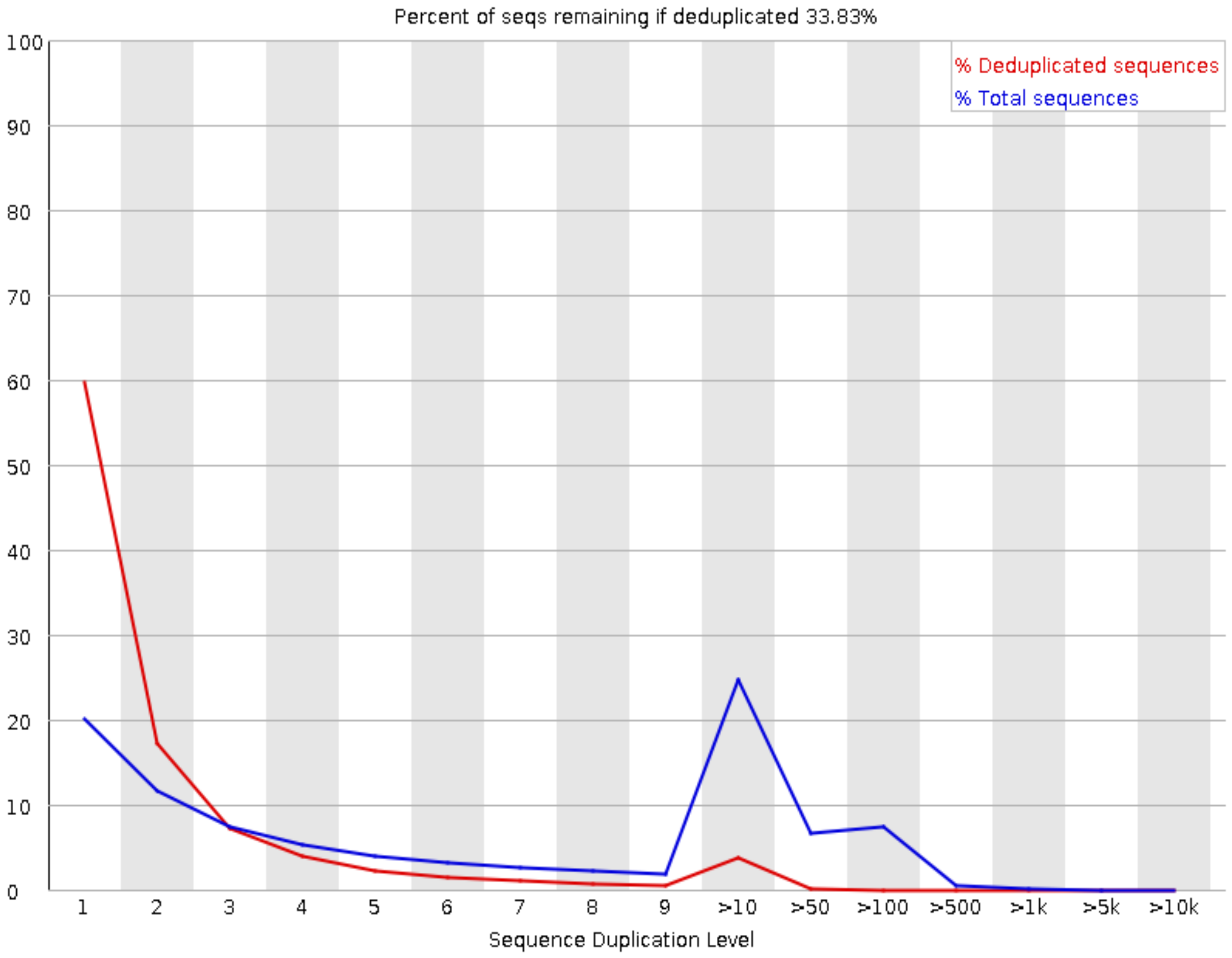

Overrepresented sequences

No overrepresented sequences

Adapter Content

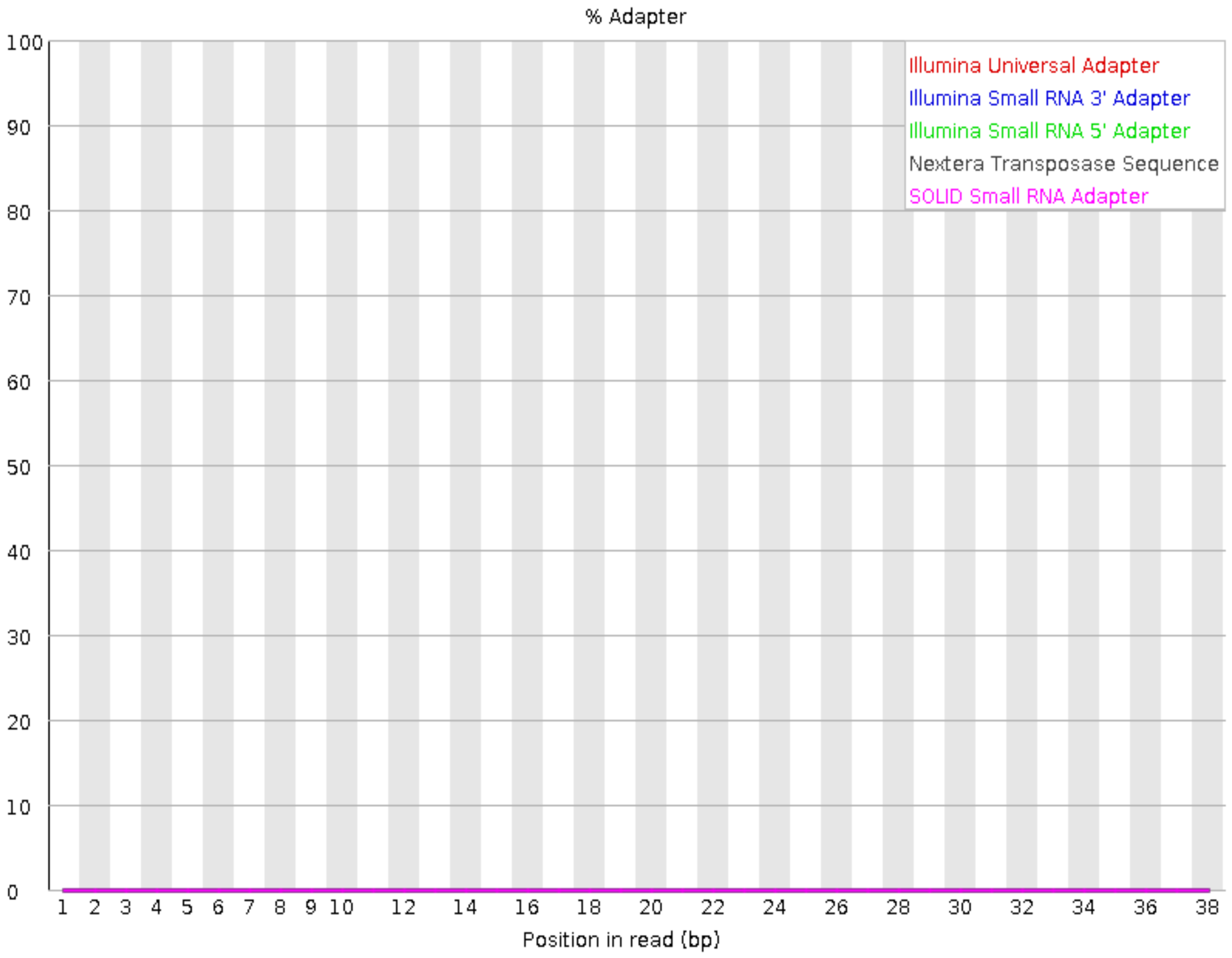

Kmer Content

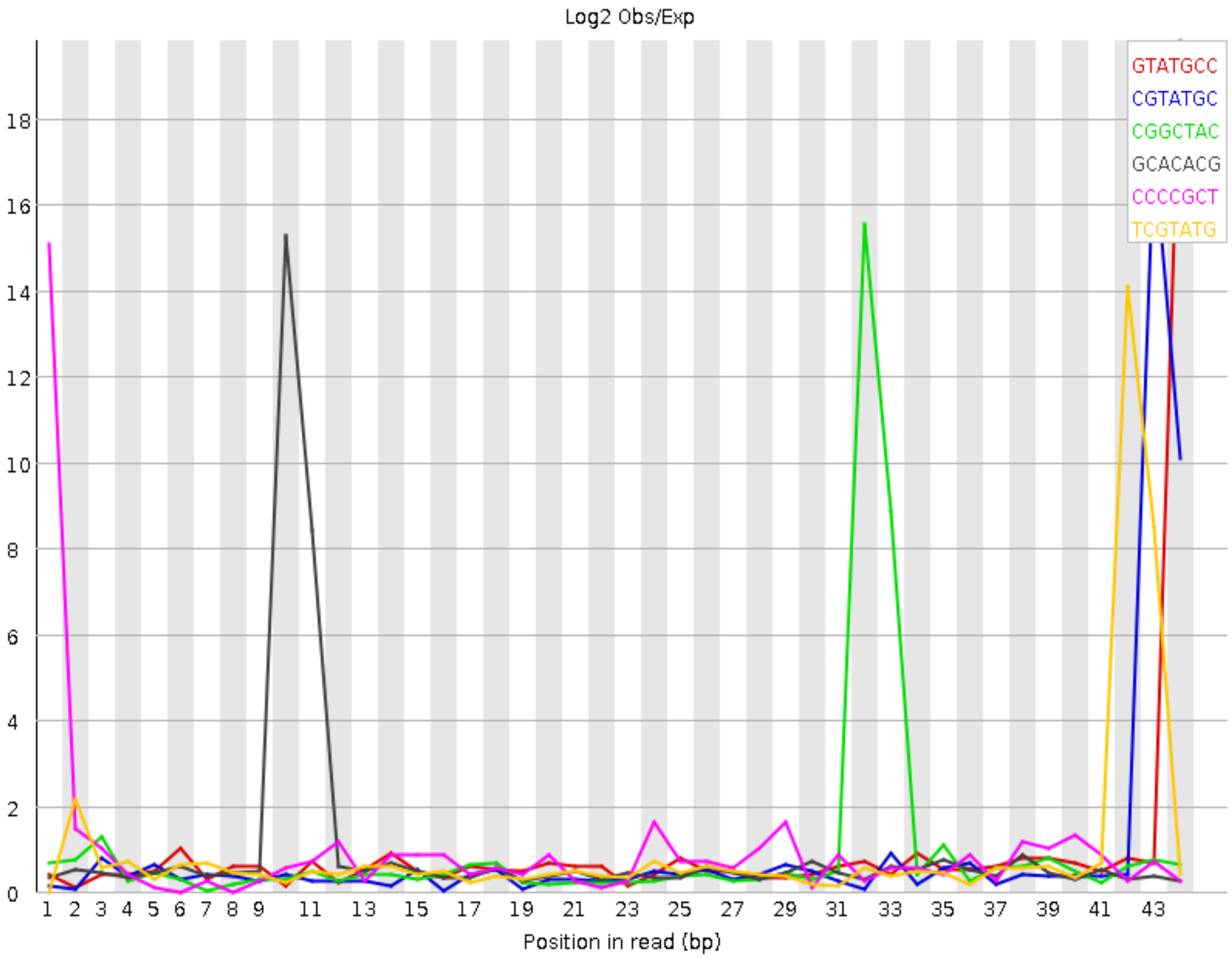

Sequence	Count	PValue	Obs/Exp Max	Max Obs/Exp Position
GTATGCC	3515	0.0	19.800291	44
CGTATGC	3985	0.0	17.354462	43
CGGCTAC	4580	0.0	15.55974	32
GCACACG	4765	0.0	15.2781315	10
CCCCGCT	1470	0.0	15.113368	1
TCGTATG	4965	0.0	14.106388	42
ACGTCTG	5400	0.0	13.848549	14
GGCTACA	5370	0.0	13.598362	33
ACGGCTA	5285	0.0	13.567363	31
CCCCCCT	820	0.0	12.876108	1
GTCCGCT	1705	0.0	12.514242	1
GGGGGCT	1570	0.0	12.469458	1
CACACGT	6025	0.0	12.338595	11

Sequence	Count	PValue	Obs/Exp Max	Max Obs/Exp Position
GGGGGCA	990	0.0	11.998191	1
GGGGCCT	890	0.0	11.86338	1
GGGGTAT	1255	0.0	11.74325	1
GCCCTAT	900	0.0	11.731565	1
GGGGGGG	150	7.808142E-4	11.7315645	1
GCCCCTT	2230	0.0	11.639501	1
CCCCCGT	780	0.0	11.56236	1

Produced by FastQC (version 0.11.5)

FastQC Report

Tue 12 Sep 2017
22008_CTTGTA_L001_R1.fastq.gz

Summary

- Basic Statistics
- Per base sequence quality
- Per tile sequence quality
- Per sequence quality scores
- Per base sequence content
- Per sequence GC content
- Per base N content
- Sequence Length Distribution
- Sequence Duplication Levels
- Overrepresented sequences
- Adapter Content
- Kmer Content

Basic Statistics

Measure	Value
Filename	22008_CTTGTA_L001_R1_001.fastq.gz
File type	Conventional base calls
Encoding	Sanger / Illumina 1.9
Total Sequences	23629366
Sequences flagged as poor quality	0
Sequence length	50
%GC	46

Per base sequence quality

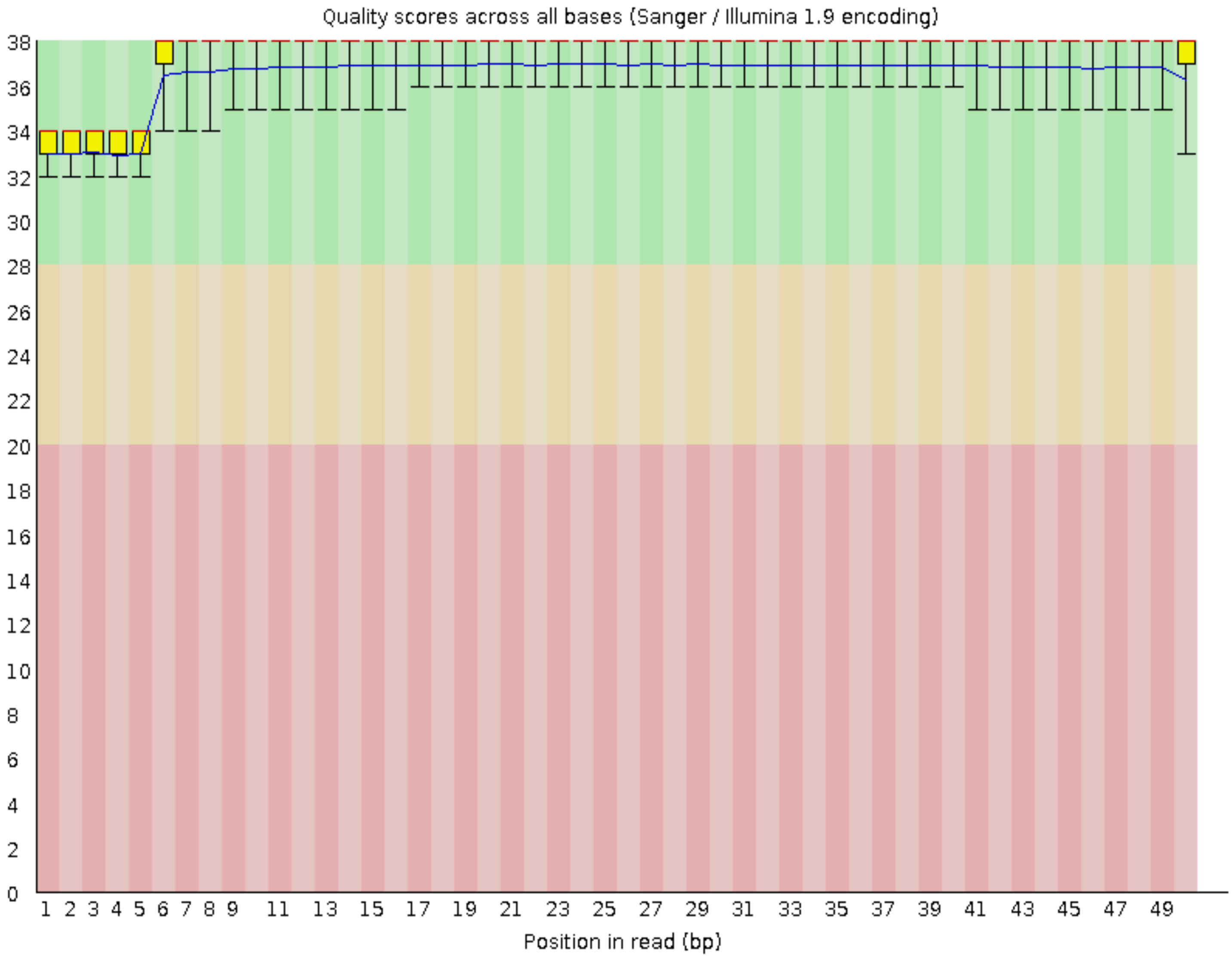

Per tile sequence quality

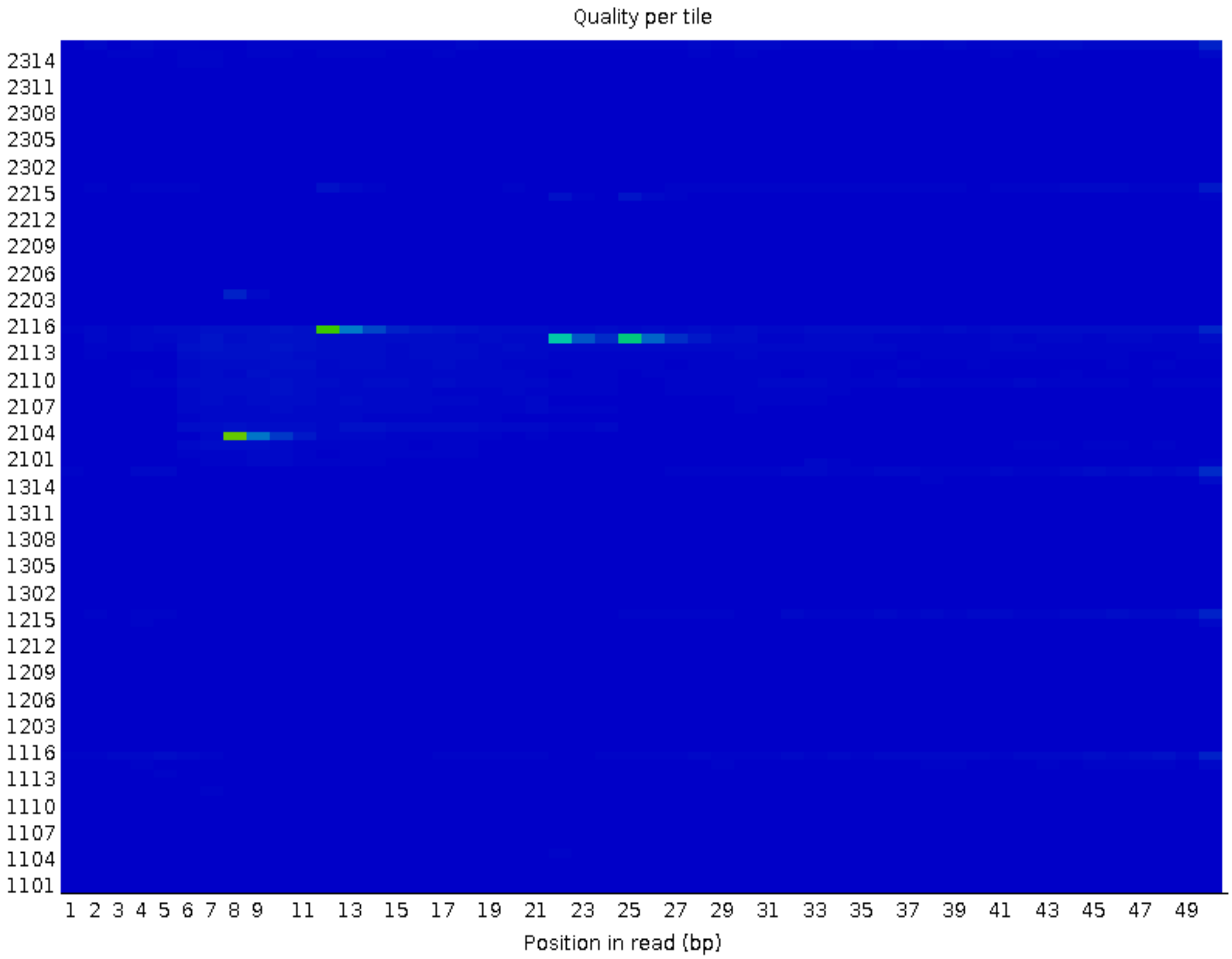

Per sequence quality scores

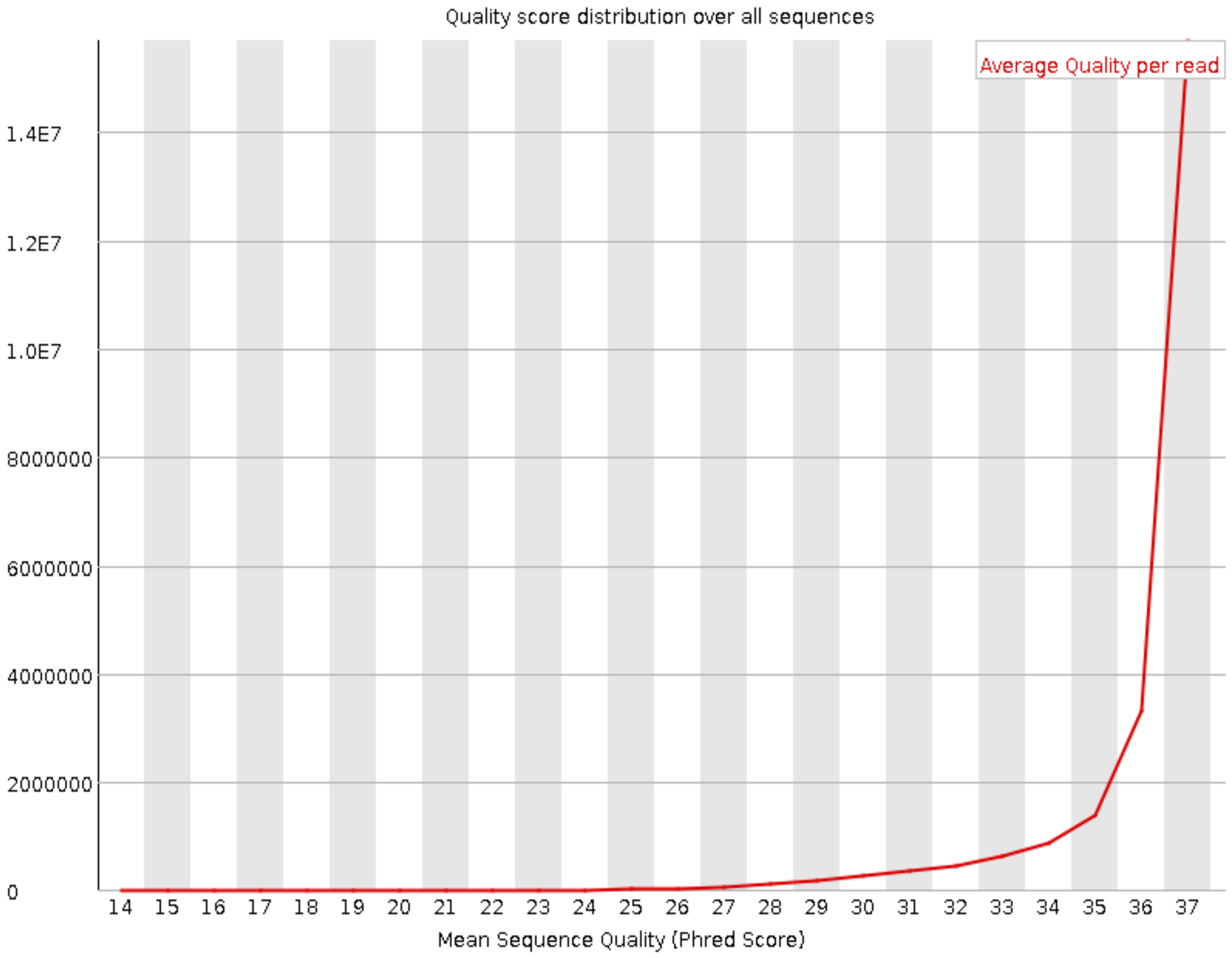

Per base sequence content

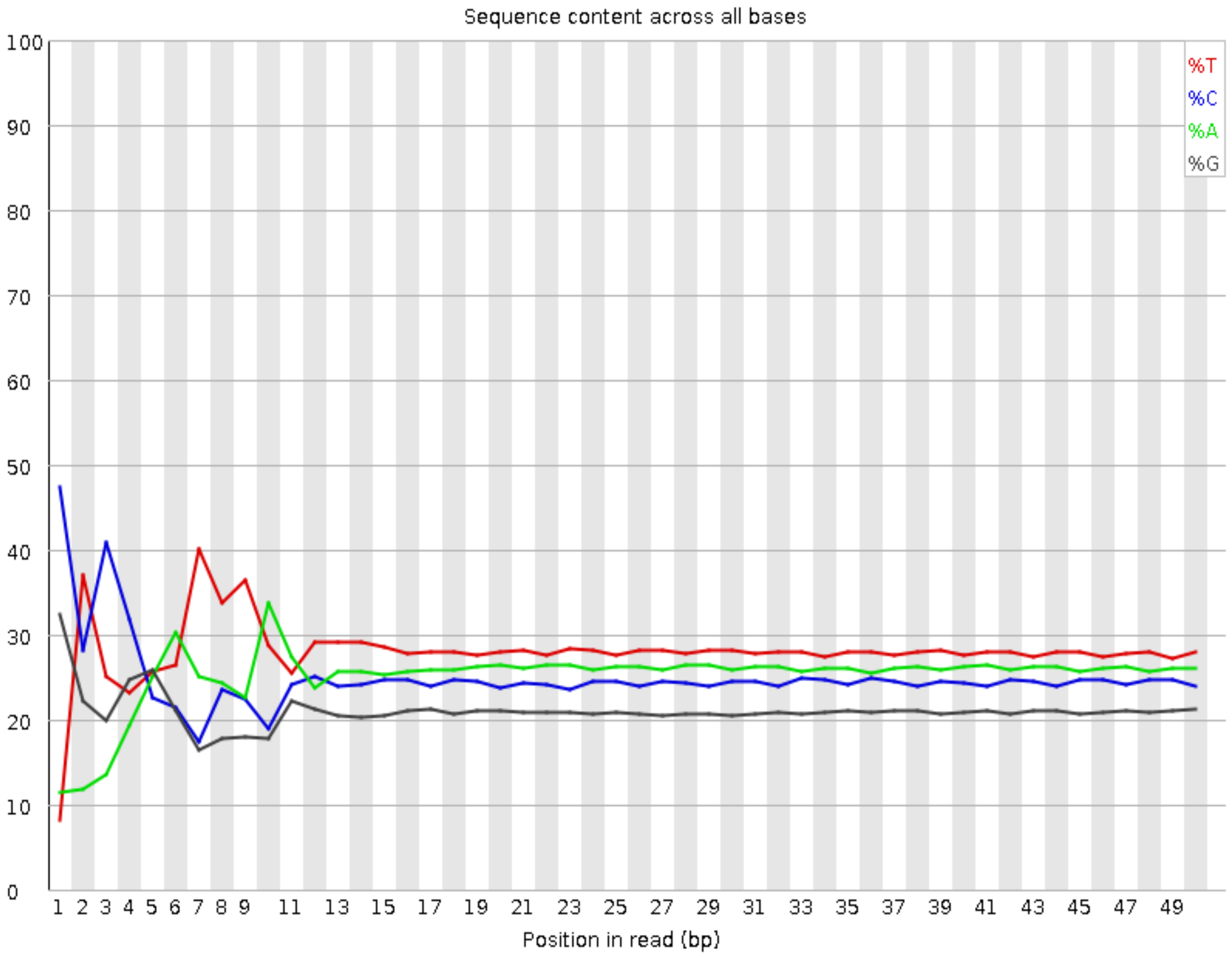

Per sequence GC content

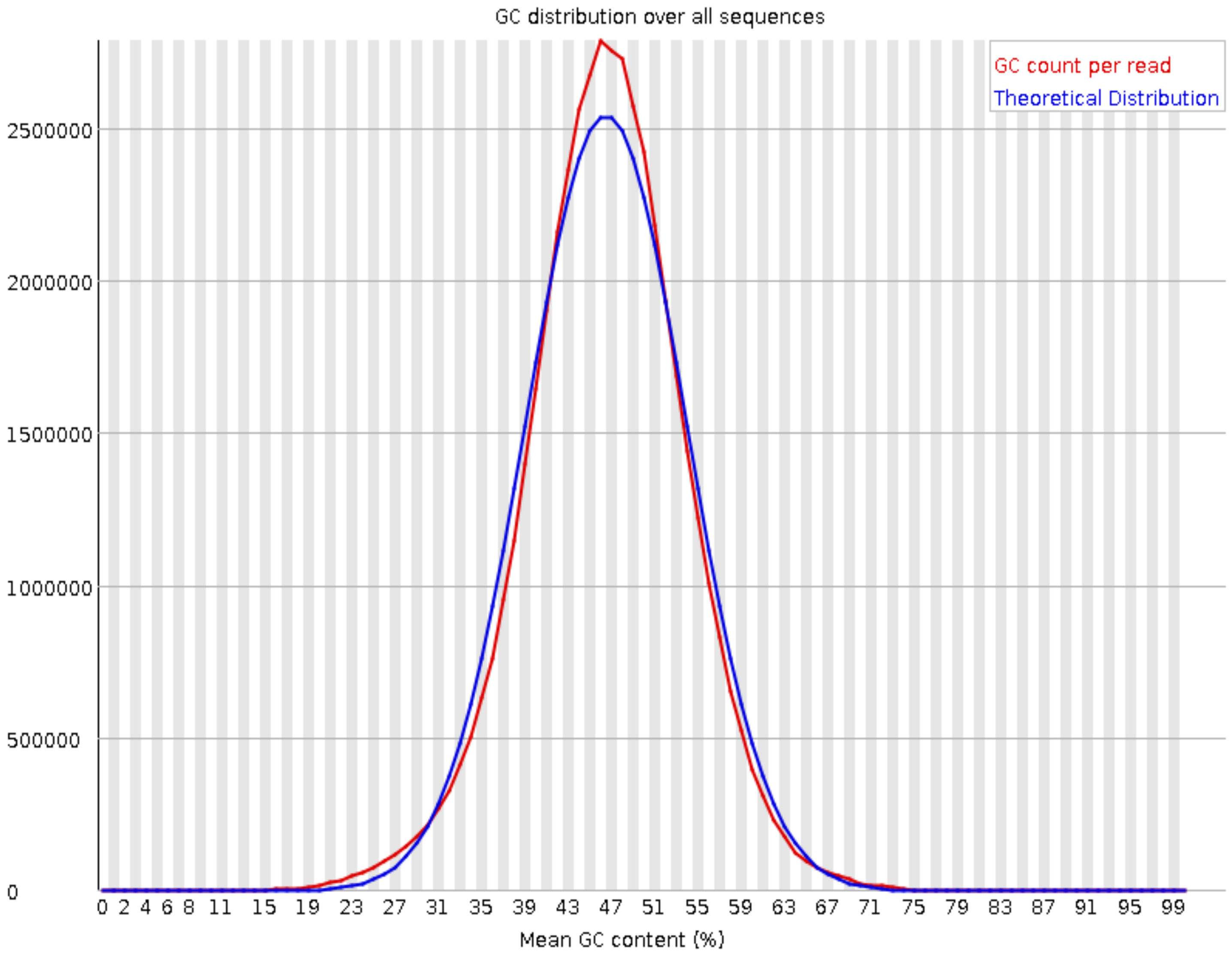

Per base N content

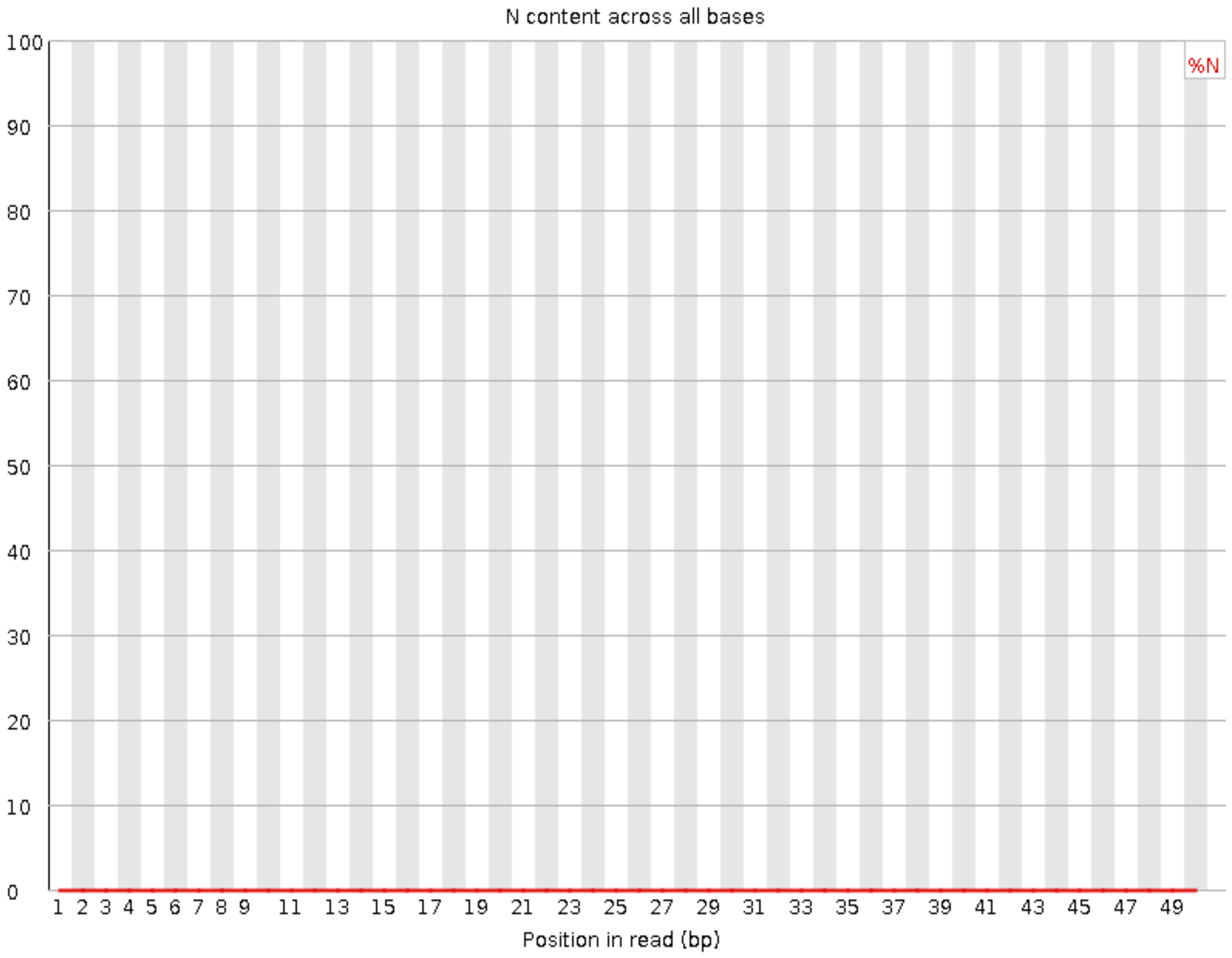

Sequence Length Distribution

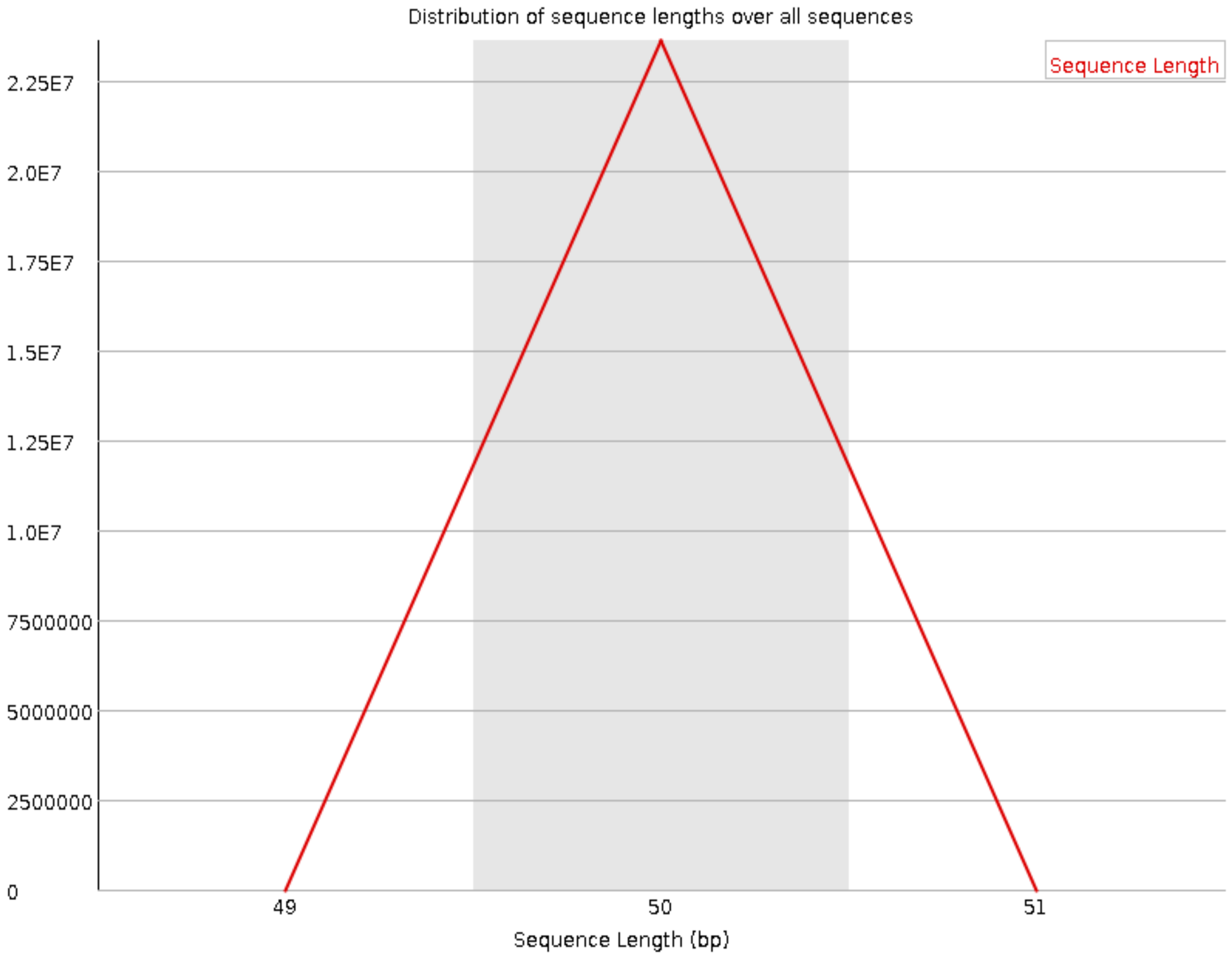

Sequence Duplication Levels

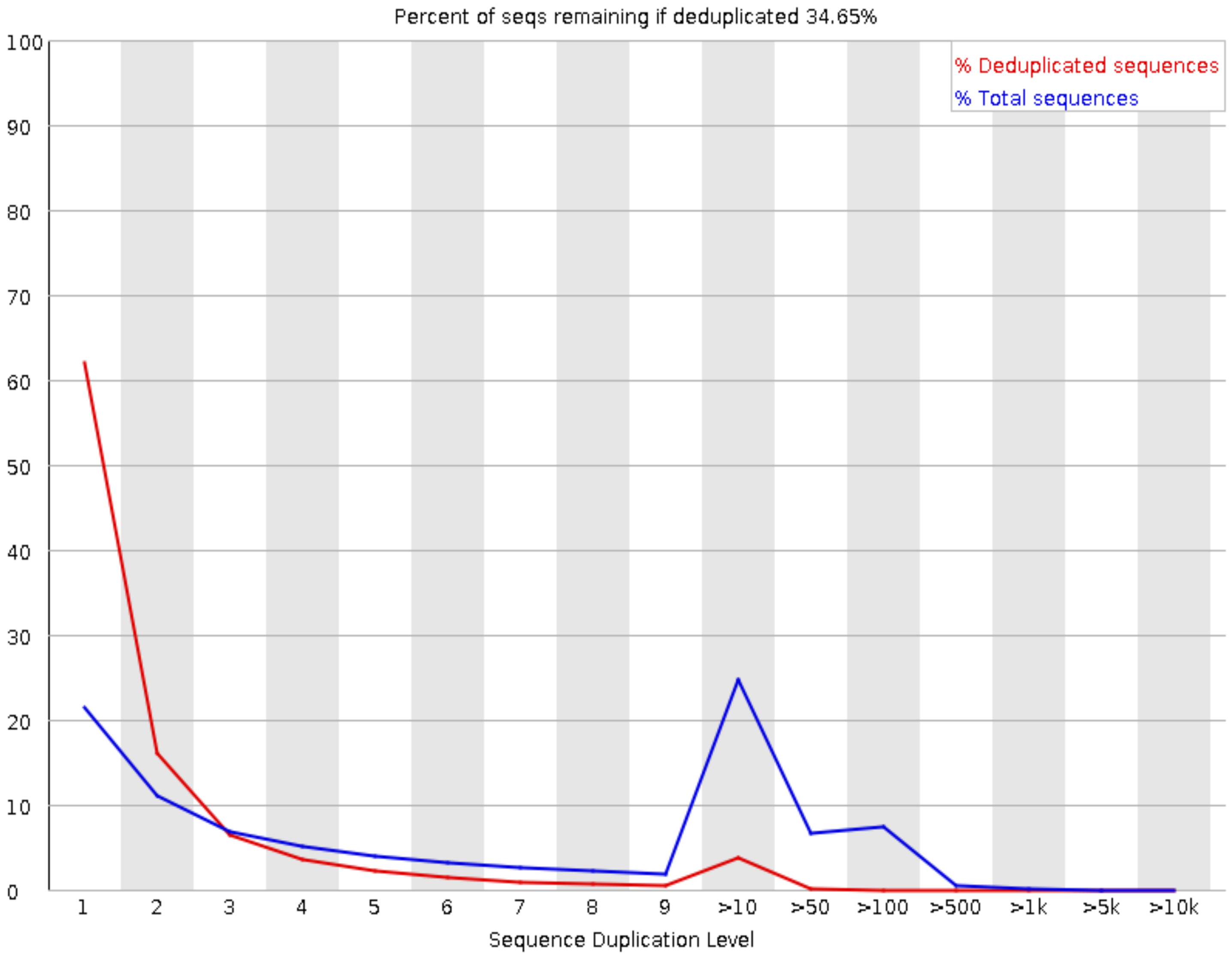

Overrepresented sequences

No overrepresented sequences

Adapter Content

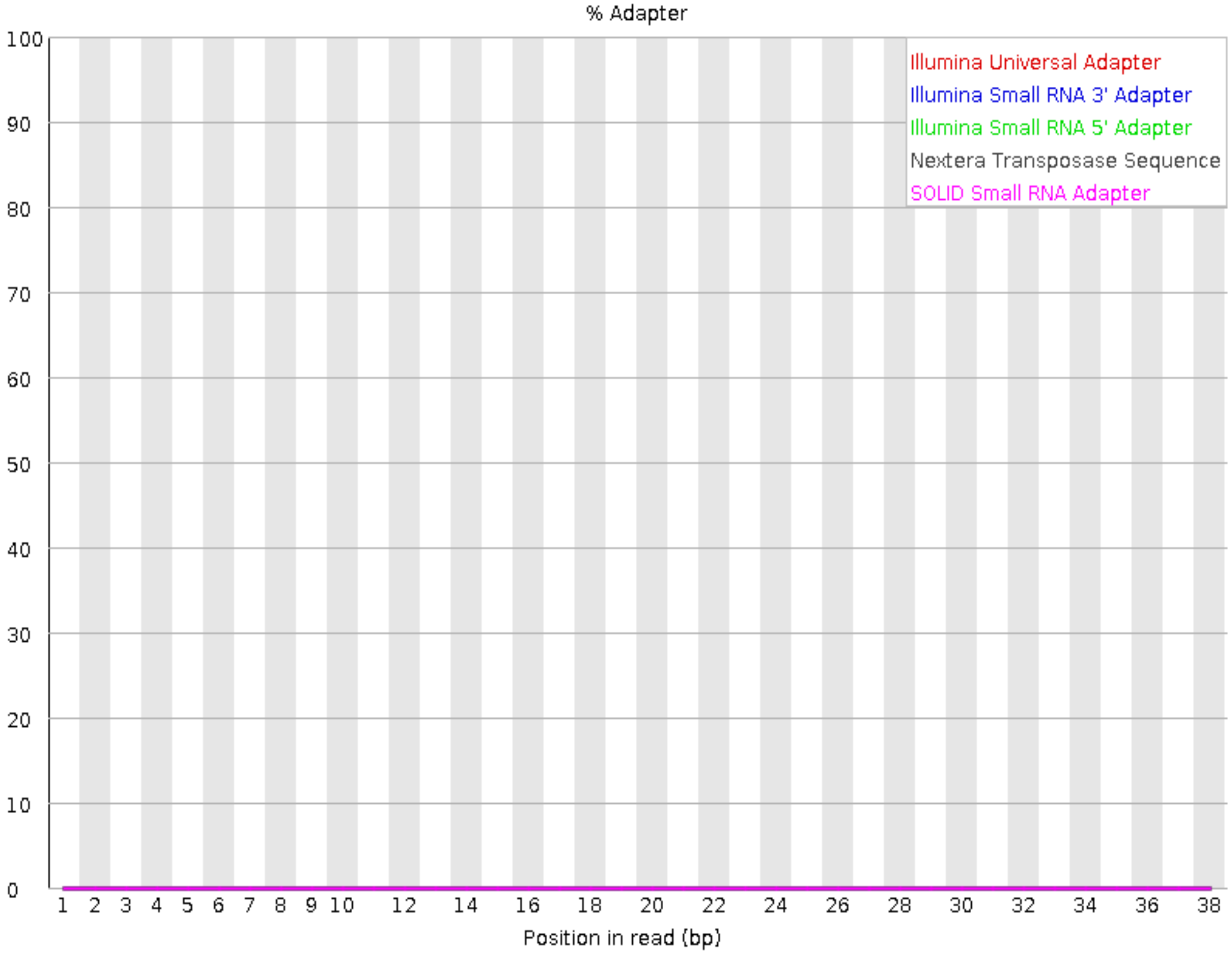

Kmer Content

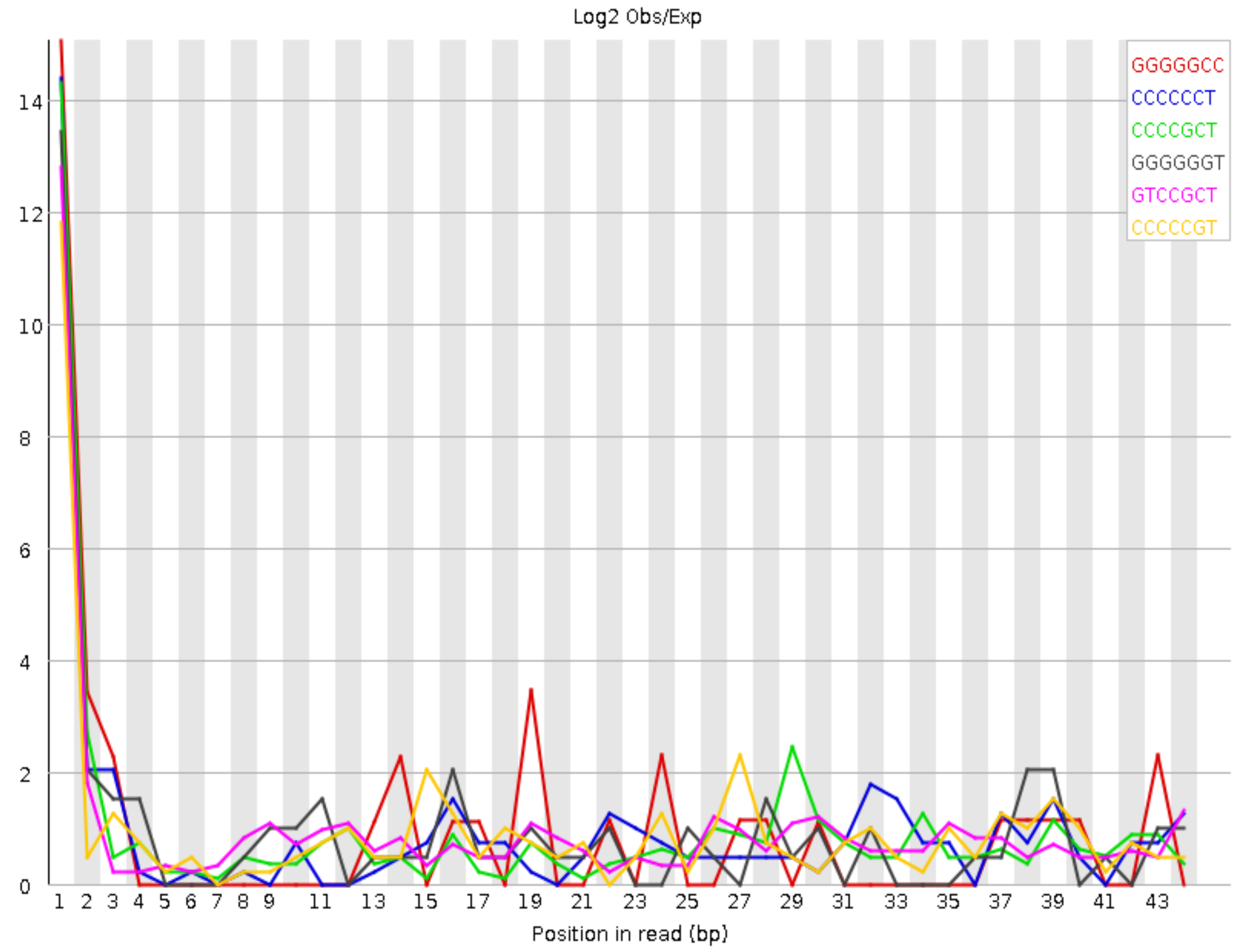

Sequence	Count	PValue	Obs/Exp Max	Max Obs/Exp Position
GGGGGCC	190	1.0815711E-8	15.0502	1
CCCCCCT	855	0.0	14.407029	1
CCCCGCT	1690	0.0	14.317214	1
GGGGGGT	425	0.0	13.45665	1
GTCCGCT	1785	0.0	12.815857	1
CCCCCGT	855	0.0	11.834346	1
GGGGGAT	1460	0.0	11.751526	1
GCCCCTT	2300	0.0	11.667681	1
CCCCGGT	1790	0.0	11.305436	1
CCCCTGT	3210	0.0	11.23806	1
GGCCCGT	725	0.0	11.225773	1
GTCCGAT	3775	0.0	11.129328	1
CCCCCAT	3435	0.0	11.078269	1

Sequence	Count	PValue	Obs/Exp Max	Max Obs/Exp Position
CCCCCTT	2190	0.0	10.948003	1
GGGGCCT	755	0.0	10.779716	1
GCCCTAT	980	0.0	10.773769	1
GGGGGGC	165	0.0017324059	10.664945	1
CGTATGC	2330	0.0	10.586415	44
GGGGGCT	1710	0.0	10.548003	1
GGGGGGG	230	2.2315782E-5	10.52004	1

Produced by FastQC (version 0.11.5)

FastQC Report

Tue 12 Sep 2017
22009_AGTCAA_L001_R1.fastq.gz

Summary

- Basic Statistics
- Per base sequence quality
- Per tile sequence quality
- Per sequence quality scores
- Per base sequence content
- Per sequence GC content
- Per base N content
- Sequence Length Distribution
- Sequence Duplication Levels
- Overrepresented sequences
- Adapter Content
- Kmer Content

Basic Statistics

Measure	Value
Filename	22009_AGTCAA_L001_R1_001.fastq.gz
File type	Conventional base calls
Encoding	Sanger / Illumina 1.9
Total Sequences	23464185
Sequences flagged as poor quality	0
Sequence length	50
%GC	46

Per base sequence quality

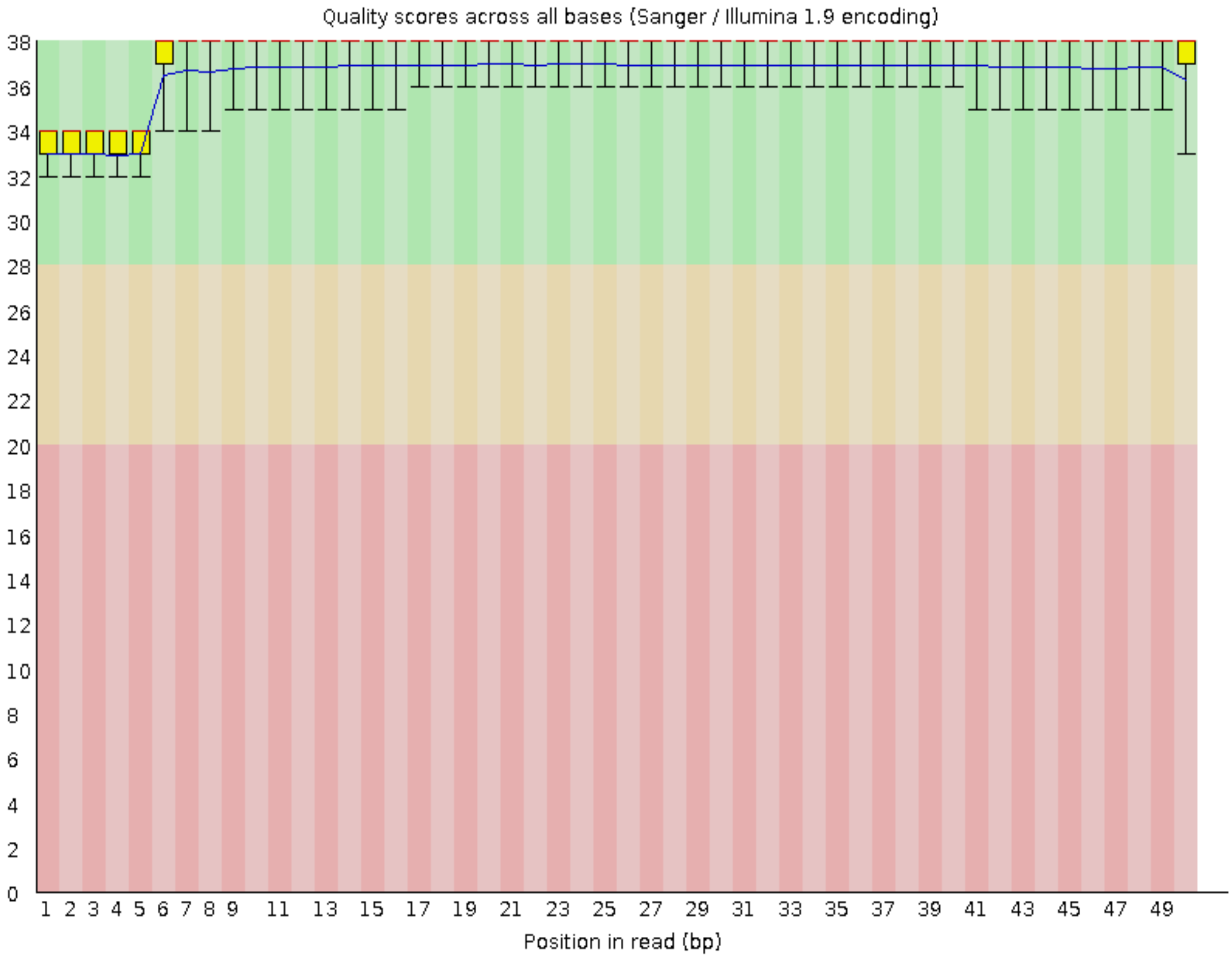

Per tile sequence quality

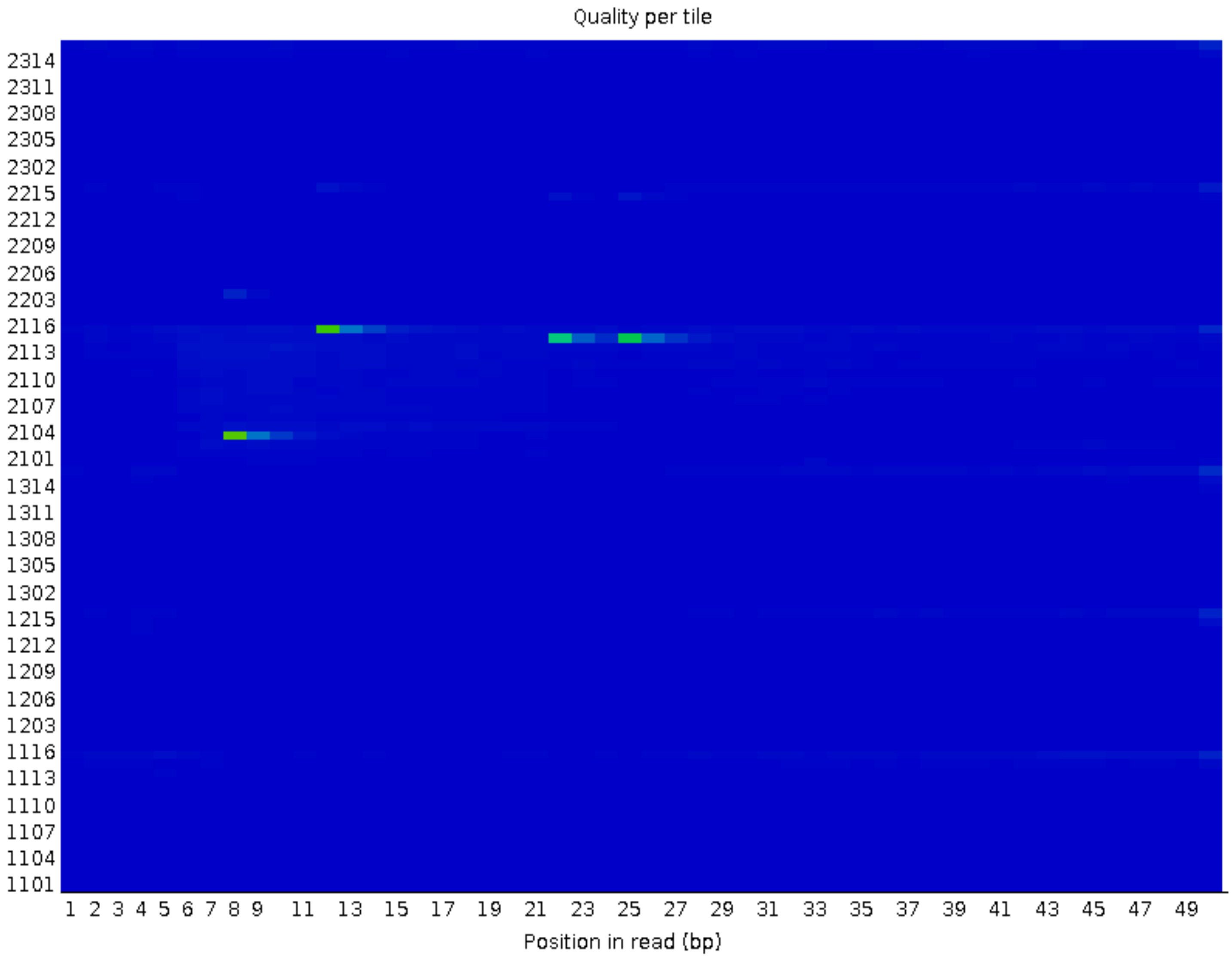

Per sequence quality scores

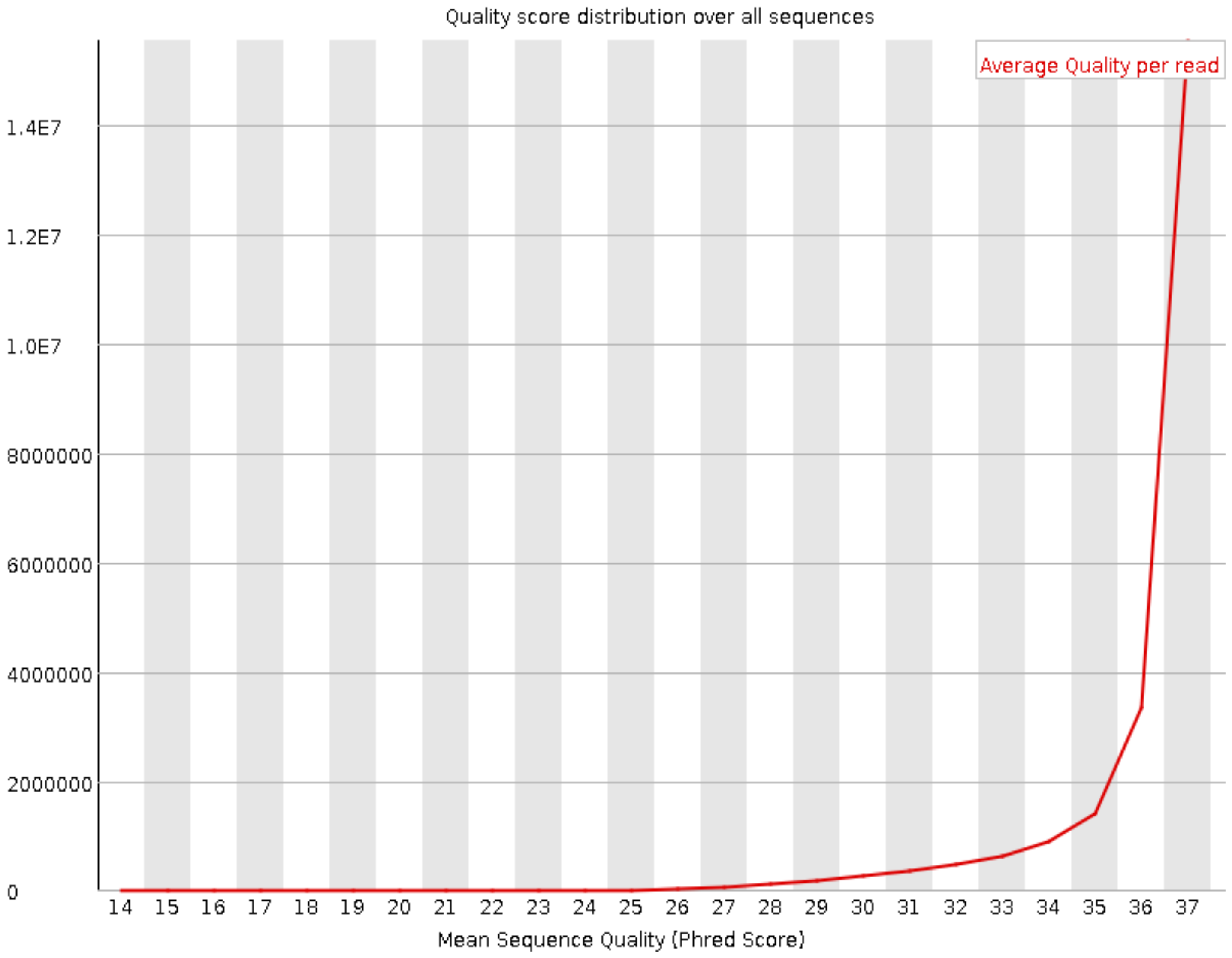

Per base sequence content

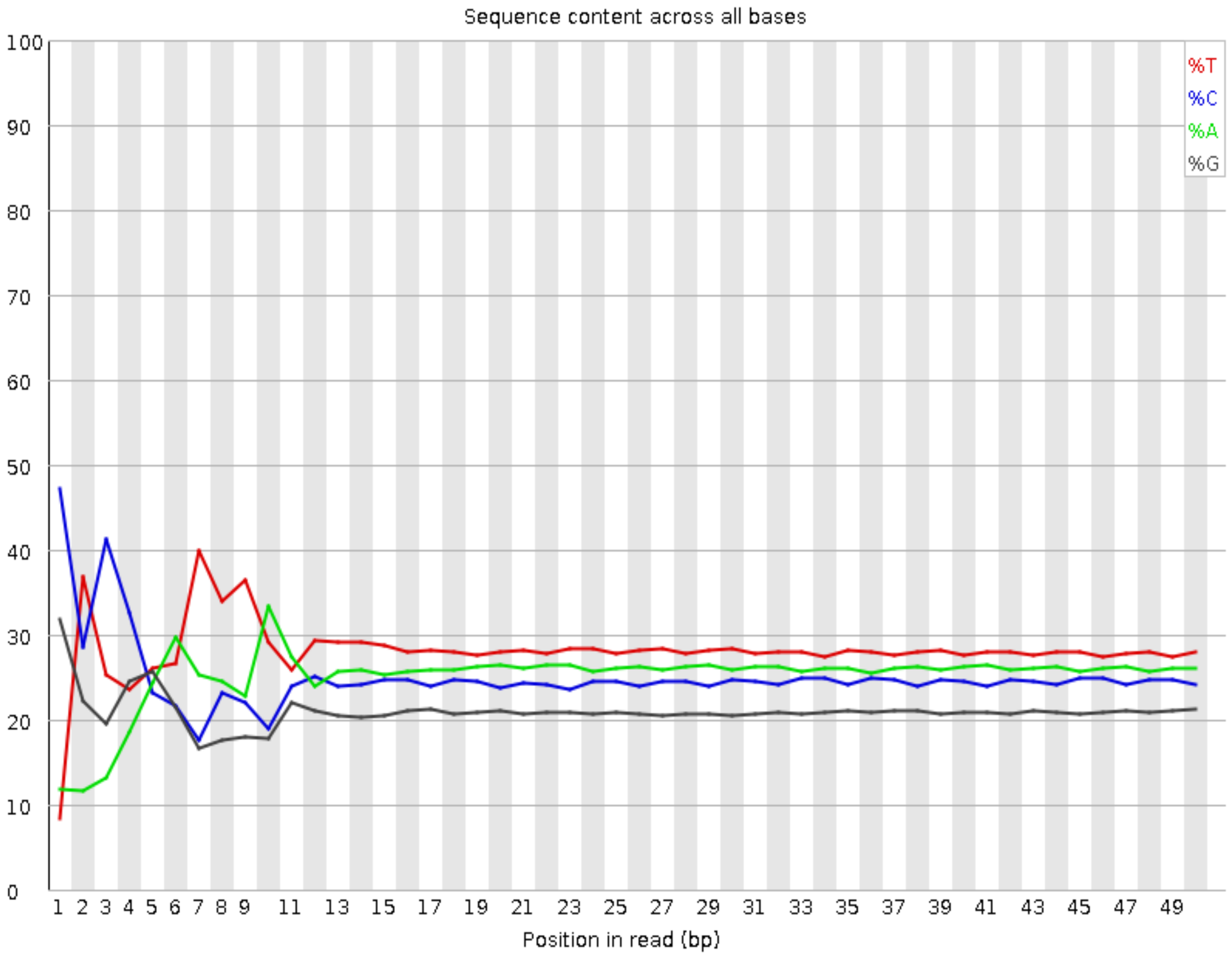

Per sequence GC content

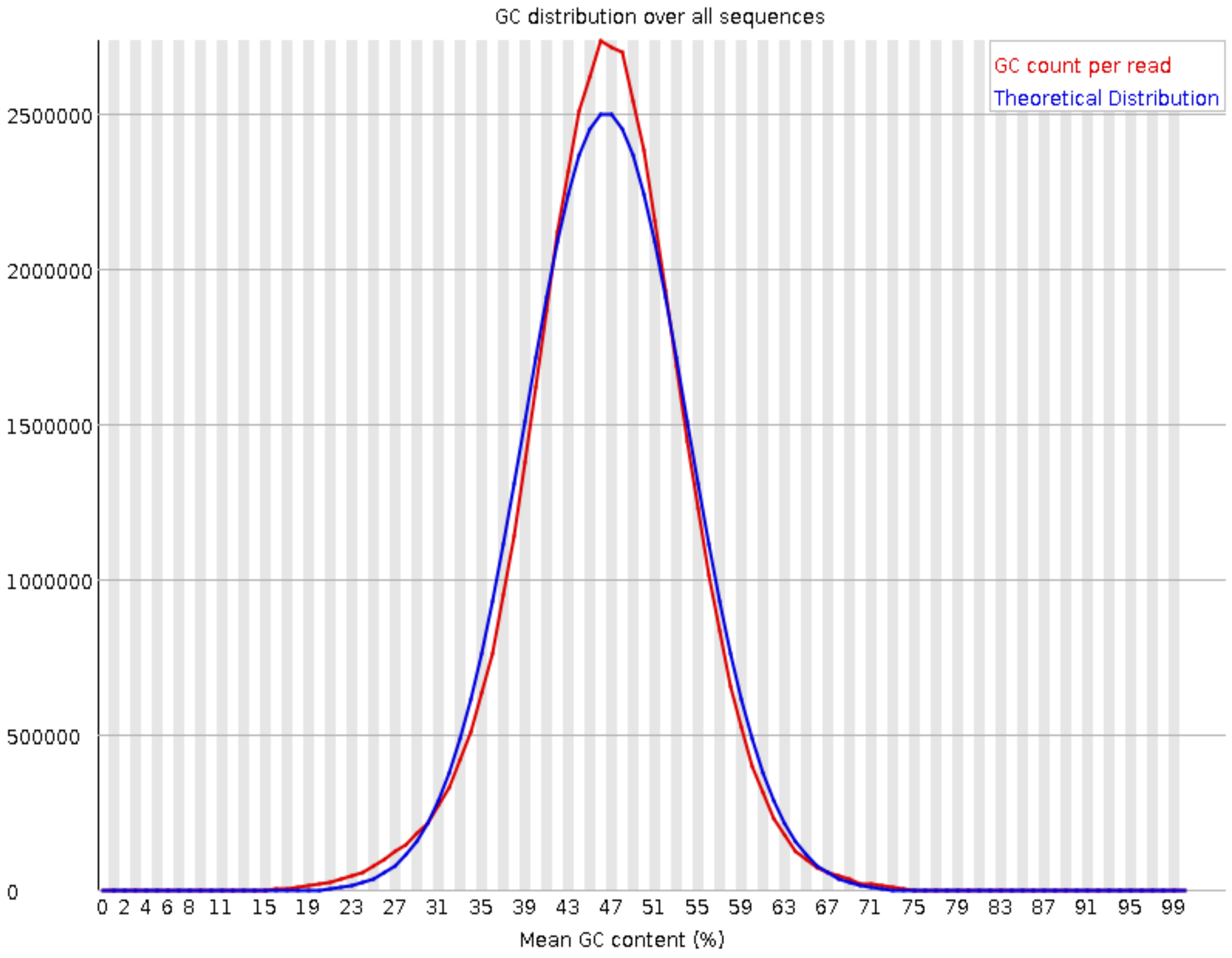

Per base N content

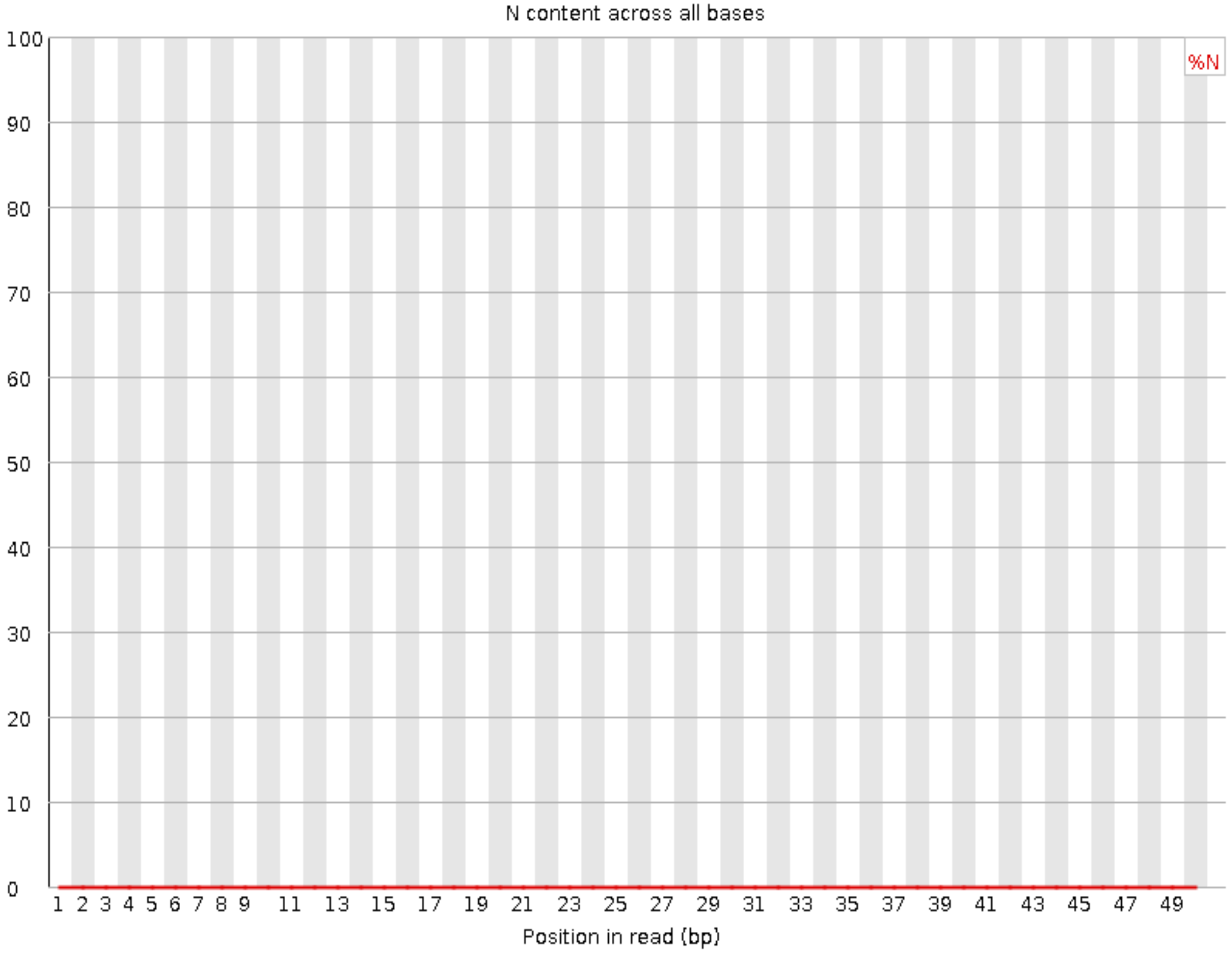

Sequence Length Distribution

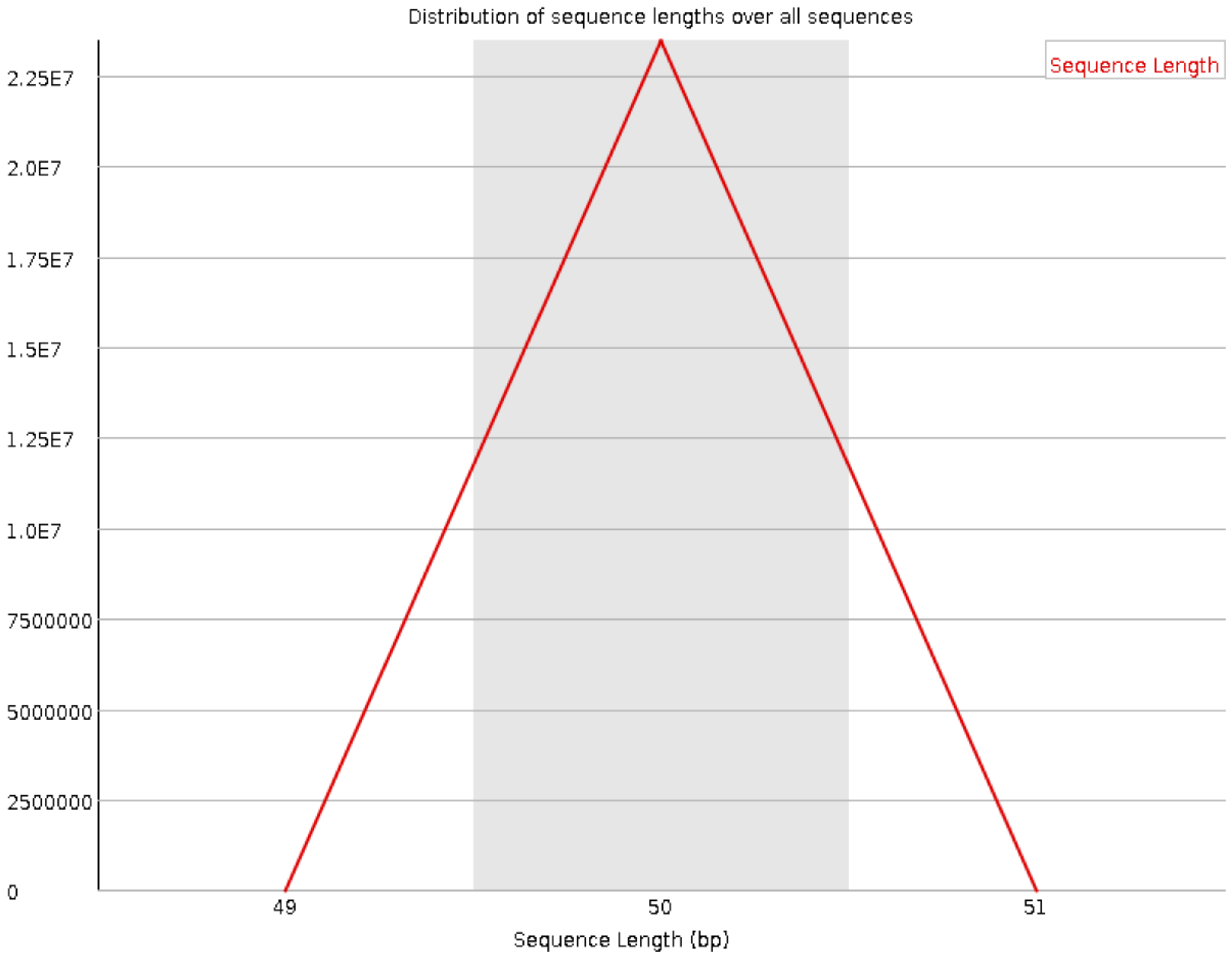

Sequence Duplication Levels

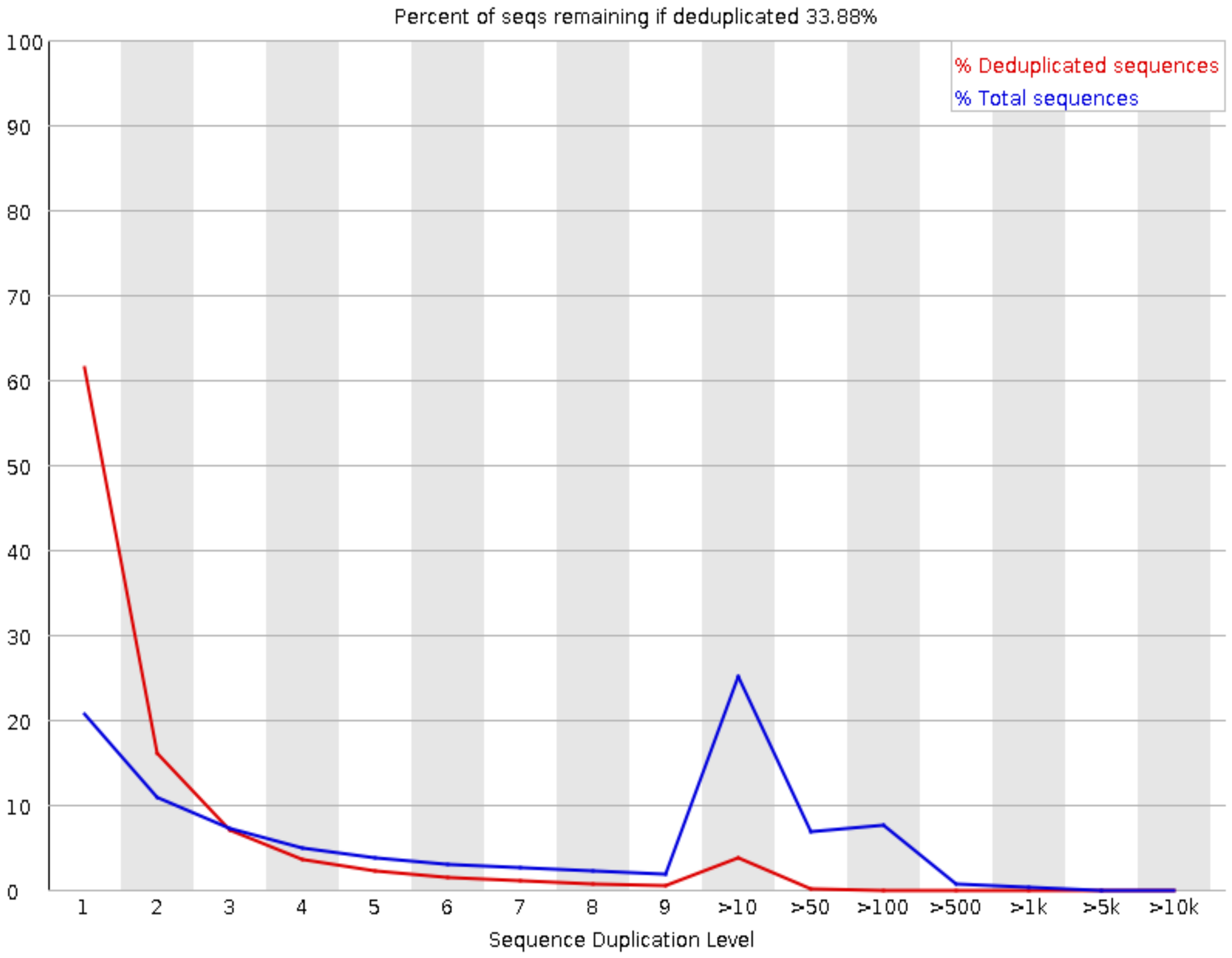

Overrepresented sequences

No overrepresented sequences

Adapter Content

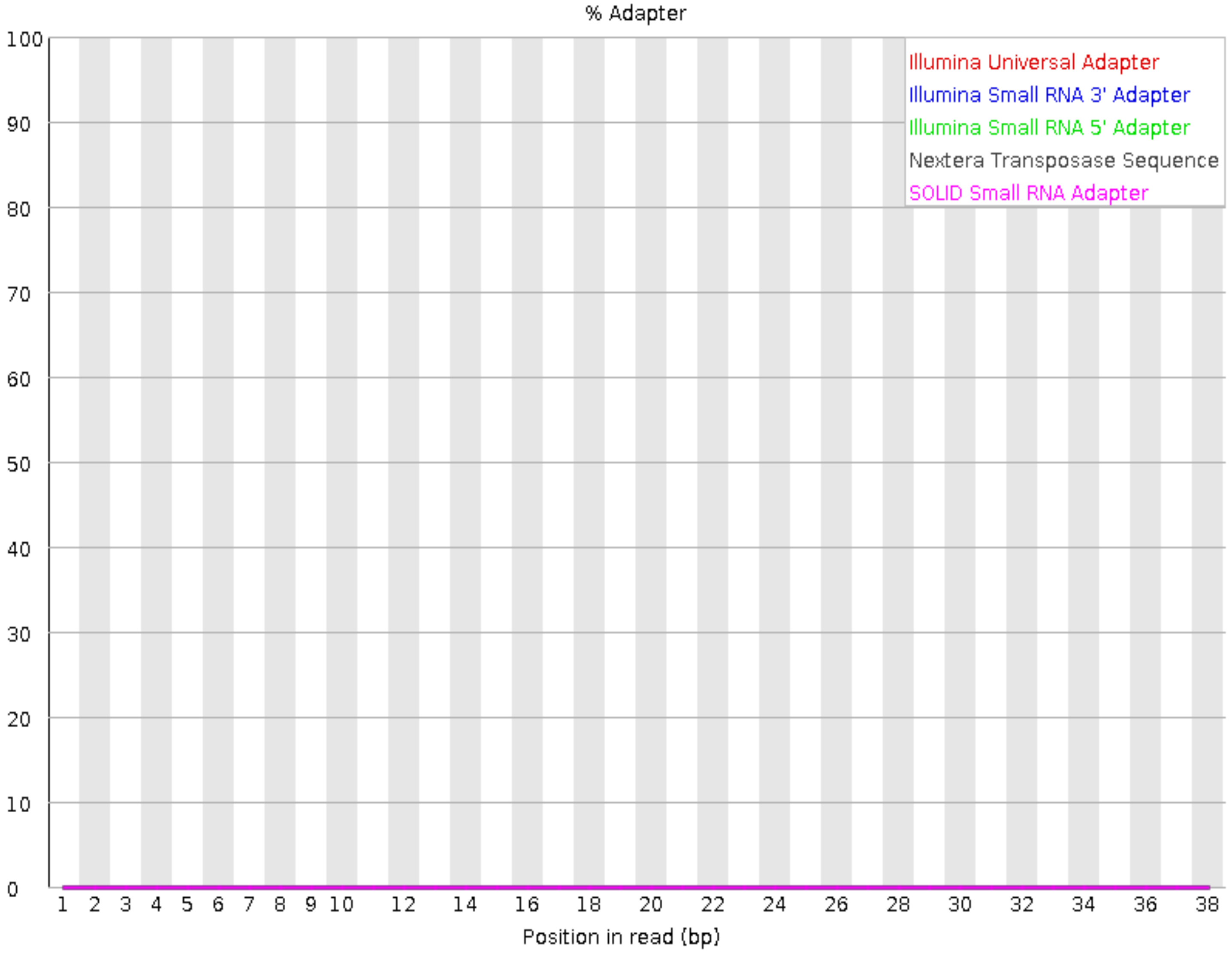

Kmer Content

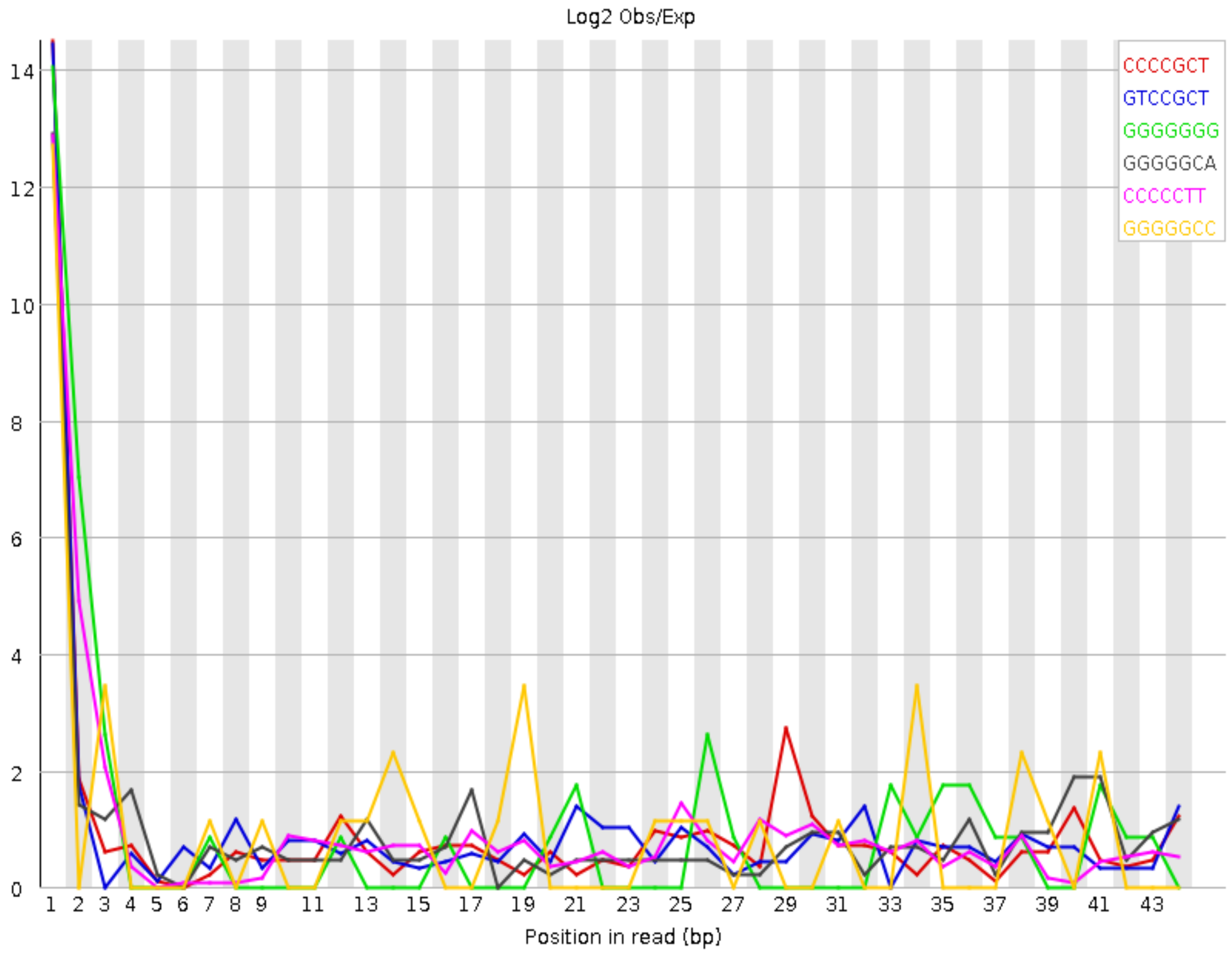

Sequence	Count	PValue	Obs/Exp Max	Max Obs/Exp Position
CCCCGCT	1760	0.0	14.4976015	1
GTCCGCT	1870	0.0	14.468196	1
GGGGGGG	250	1.382432E-10	14.077671	1
GGGGGCA	920	0.0	12.910908	1
CCCCCTT	2420	0.0	12.906956	1
GGGGGCC	190	2.663388E-6	12.734736	1
CCCCCCT	795	0.0	12.727454	1
CCCCCGT	875	0.0	12.317962	1
GGCCCGC	395	1.8189894E-12	11.694268	1
GCCCCTT	2185	0.0	11.677703	1
GCACACG	3835	0.0	11.355549	11
CCCCTGT	2965	0.0	11.350568	1
GCCCTAT	915	0.0	11.298678	1

Sequence	Count	PValue	Obs/Exp Max	Max Obs/Exp Position
CCCCGTT	1615	0.0	11.03223	1
TCGTATG	3645	0.0	10.815169	44
CGGGCAT	1635	0.0	10.762747	1
GGGGCCT	945	0.0	10.707224	1
GTCCGCA	1460	0.0	10.696861	1
CCCCCAT	3525	0.0	10.608174	1
CCCCTCT	4120	0.0	10.571068	1

Produced by FastQC (version 0.11.5)

FastQC Report

Tue 12 Sep 2017
22010_AGTTCC_L001_R1.fastq.gz

Summary

- Basic Statistics
- Per base sequence quality
- Per tile sequence quality
- Per sequence quality scores
- Per base sequence content
- Per sequence GC content
- Per base N content
- Sequence Length Distribution
- Sequence Duplication Levels
- Overrepresented sequences
- Adapter Content
- Kmer Content

Basic Statistics

Measure	Value
Filename	22010_AGTTCC_L001_R1_001.fastq.gz
File type	Conventional base calls
Encoding	Sanger / Illumina 1.9
Total Sequences	25902480
Sequences flagged as poor quality	0
Sequence length	50
%GC	46

Per base sequence quality

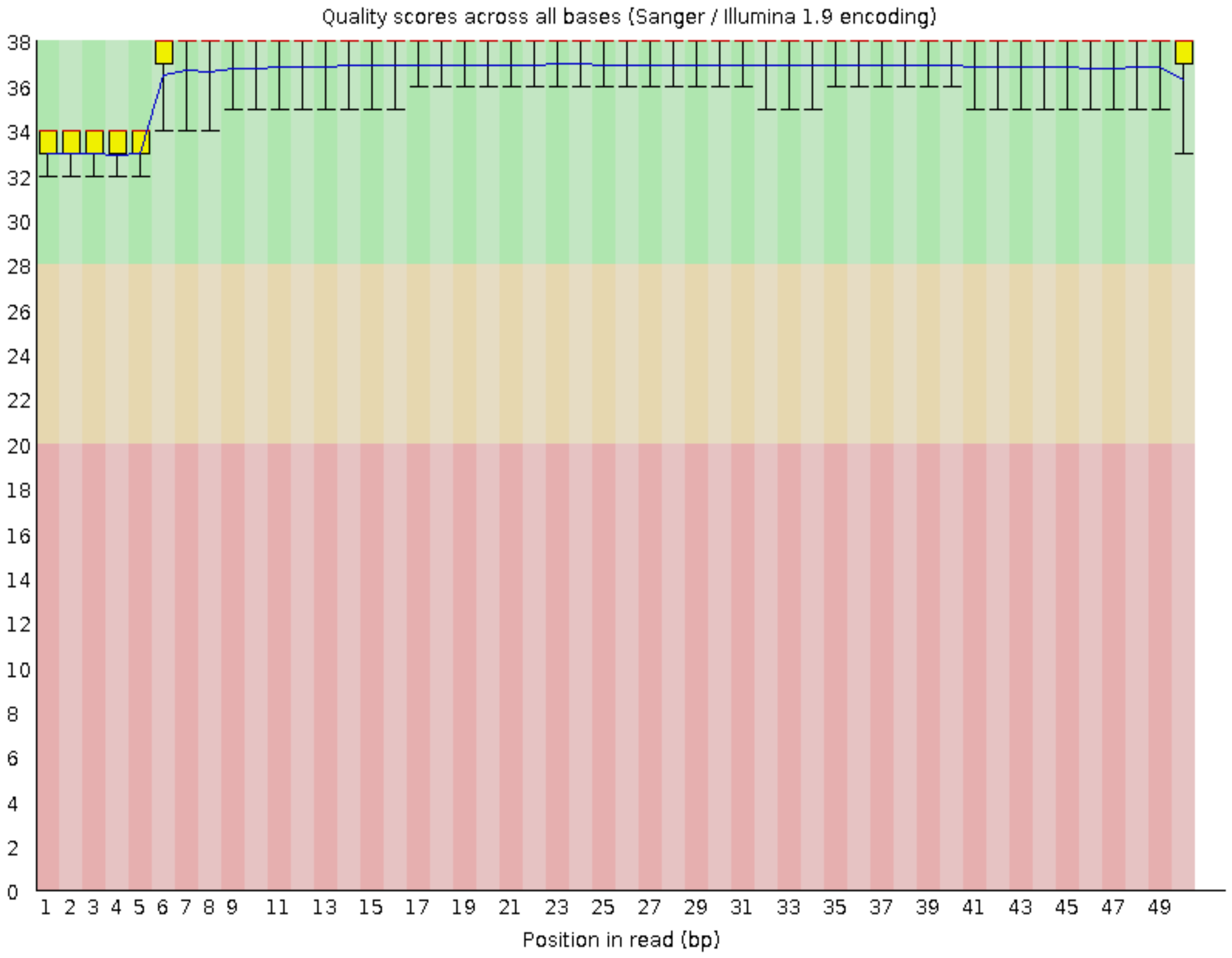

Per tile sequence quality

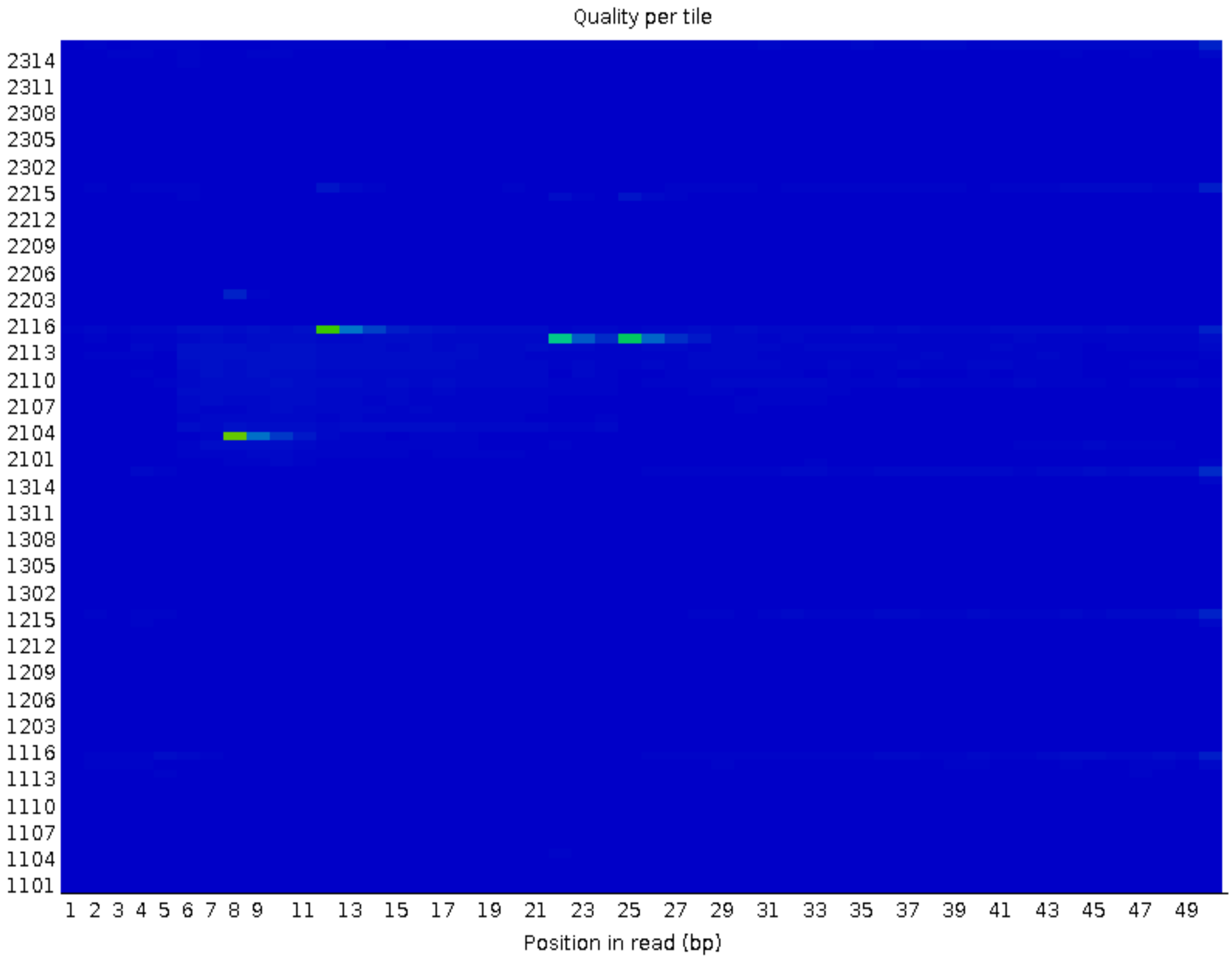

Per sequence quality scores

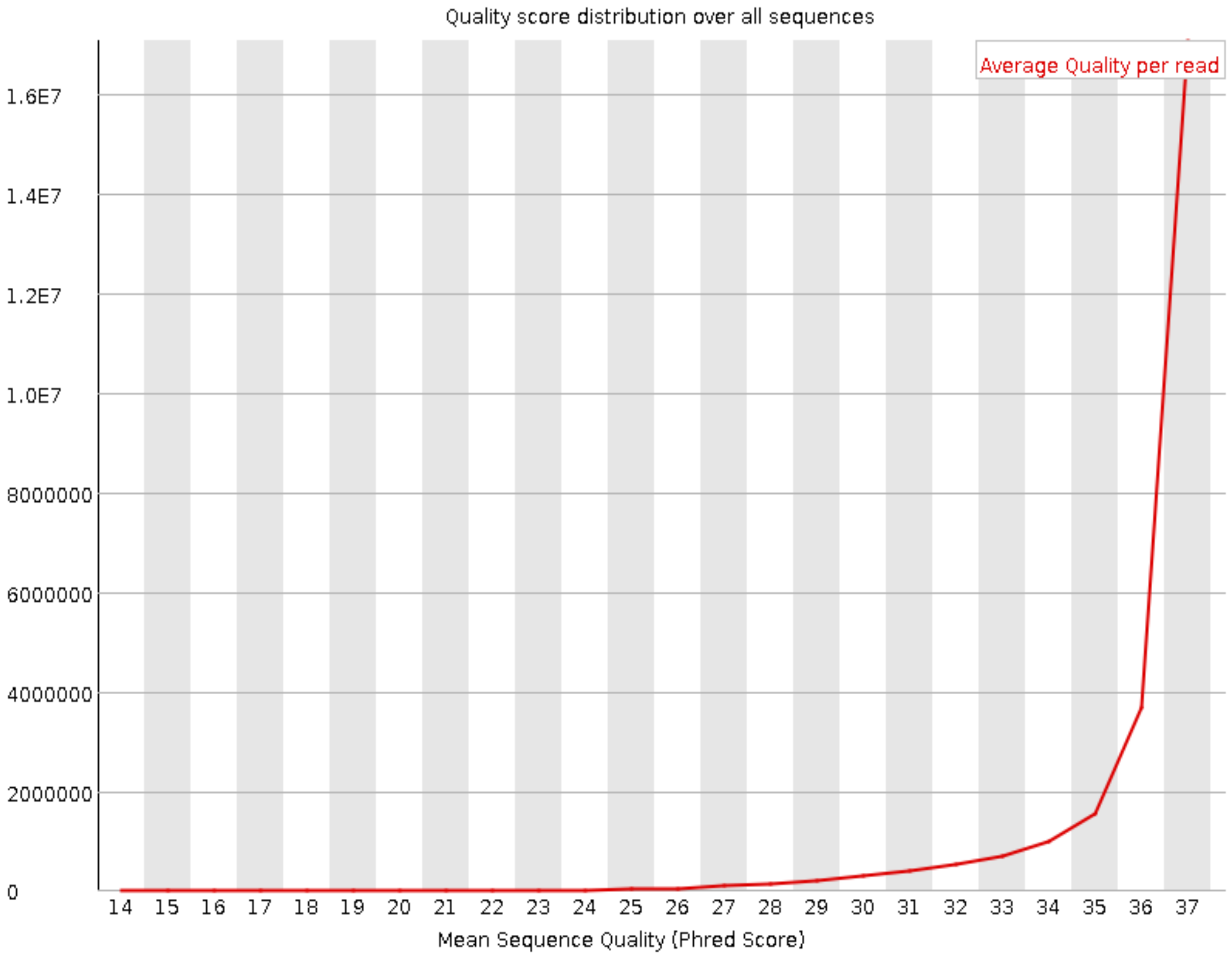

Per base sequence content

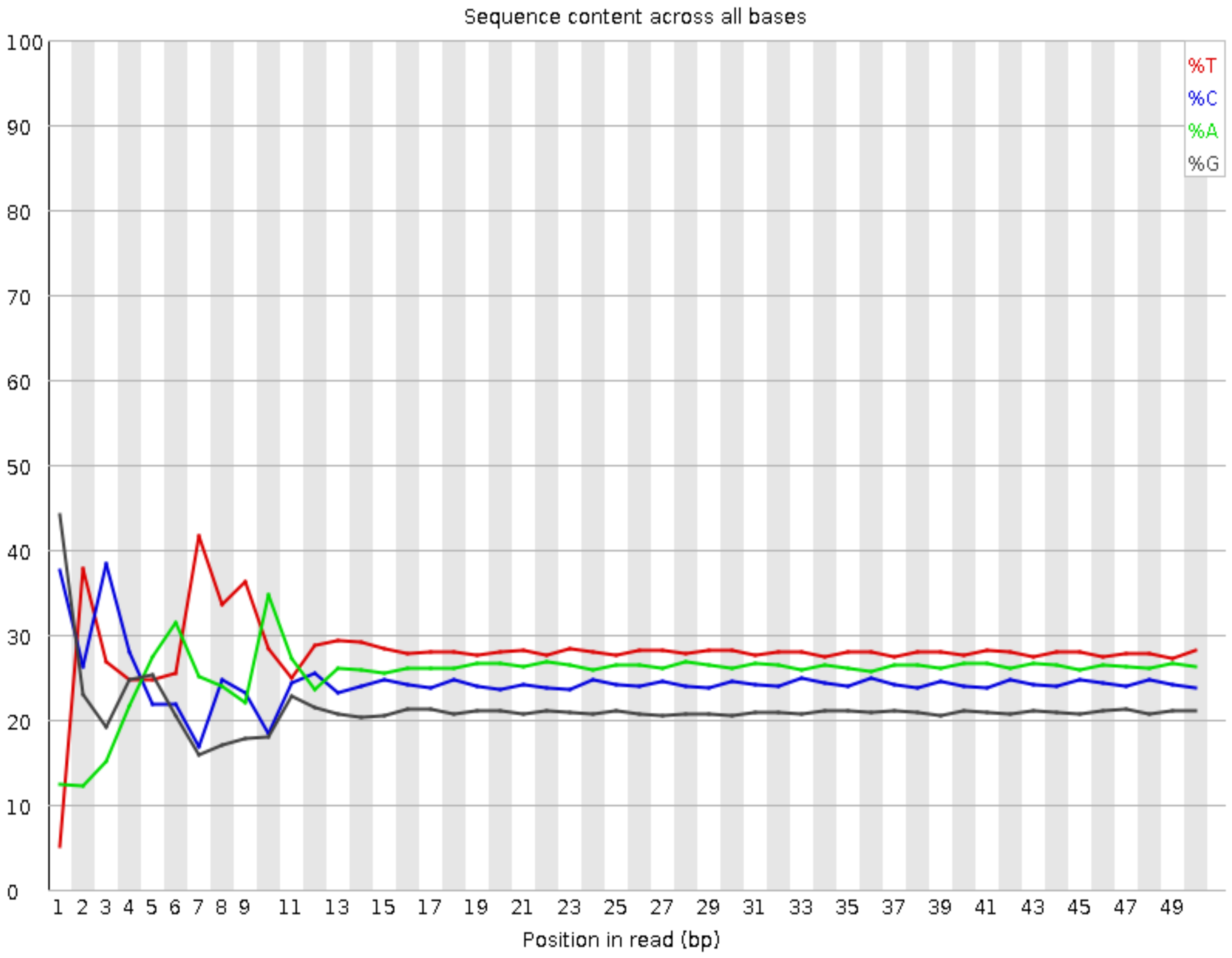

Per sequence GC content

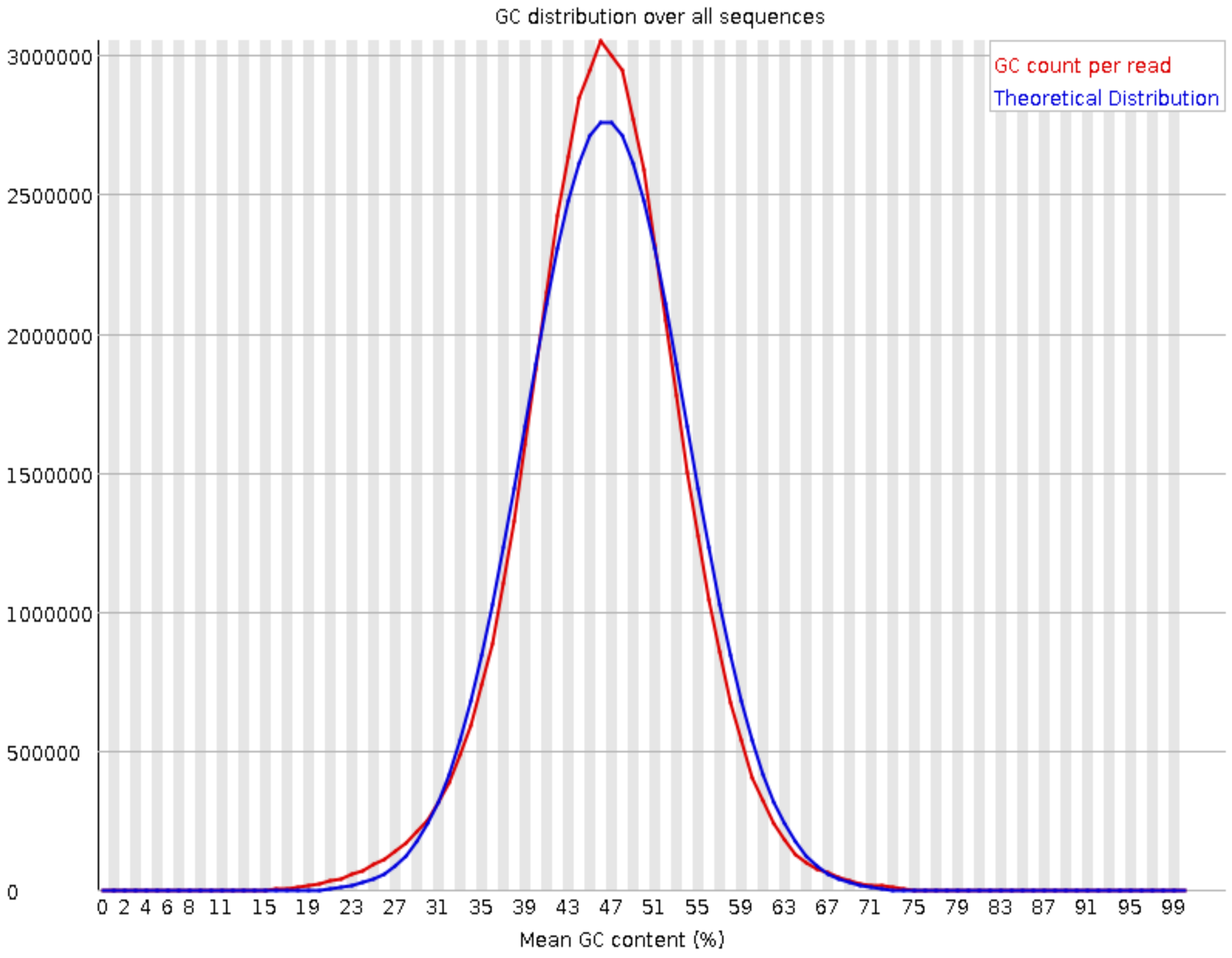

Per base N content

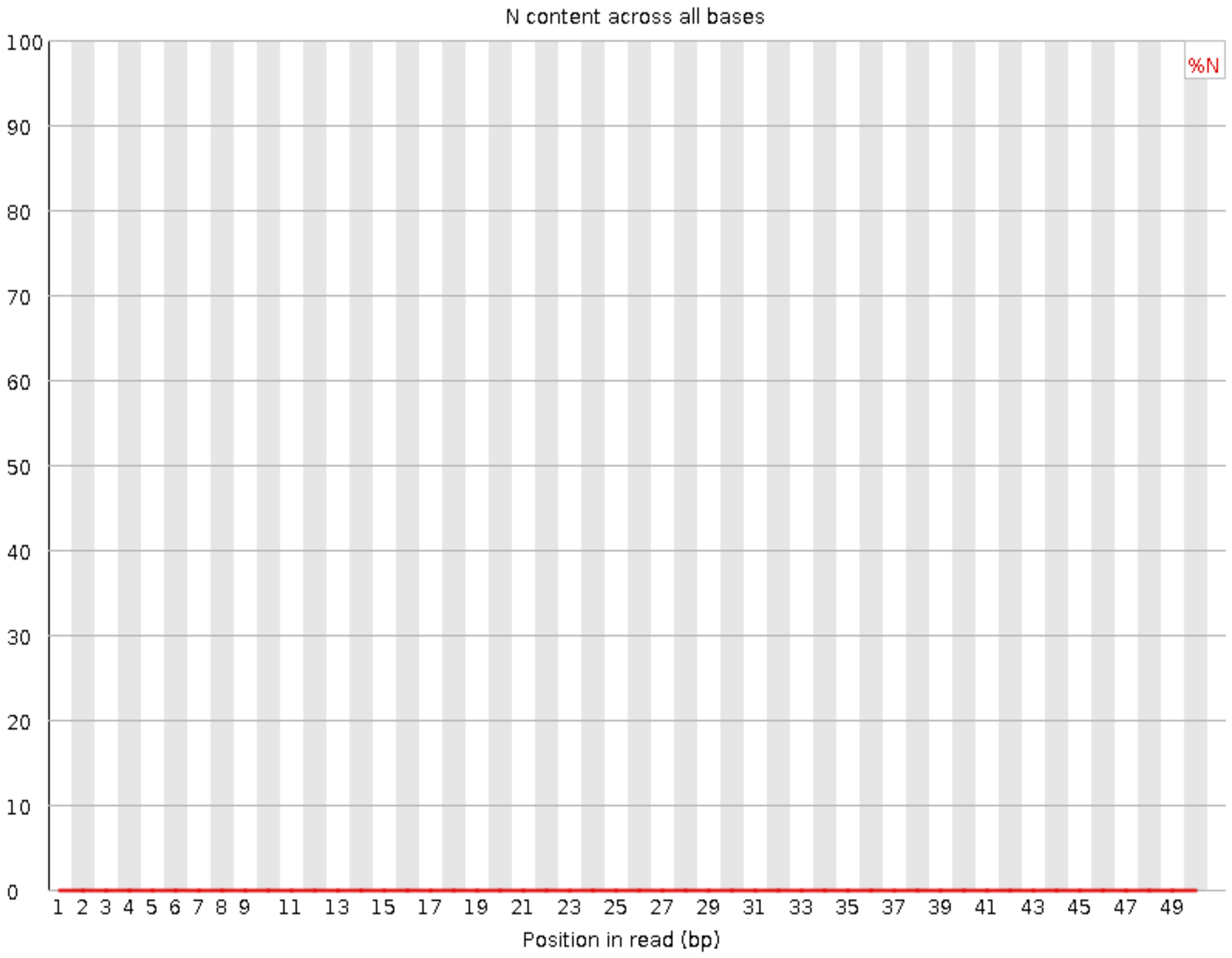

Sequence Length Distribution

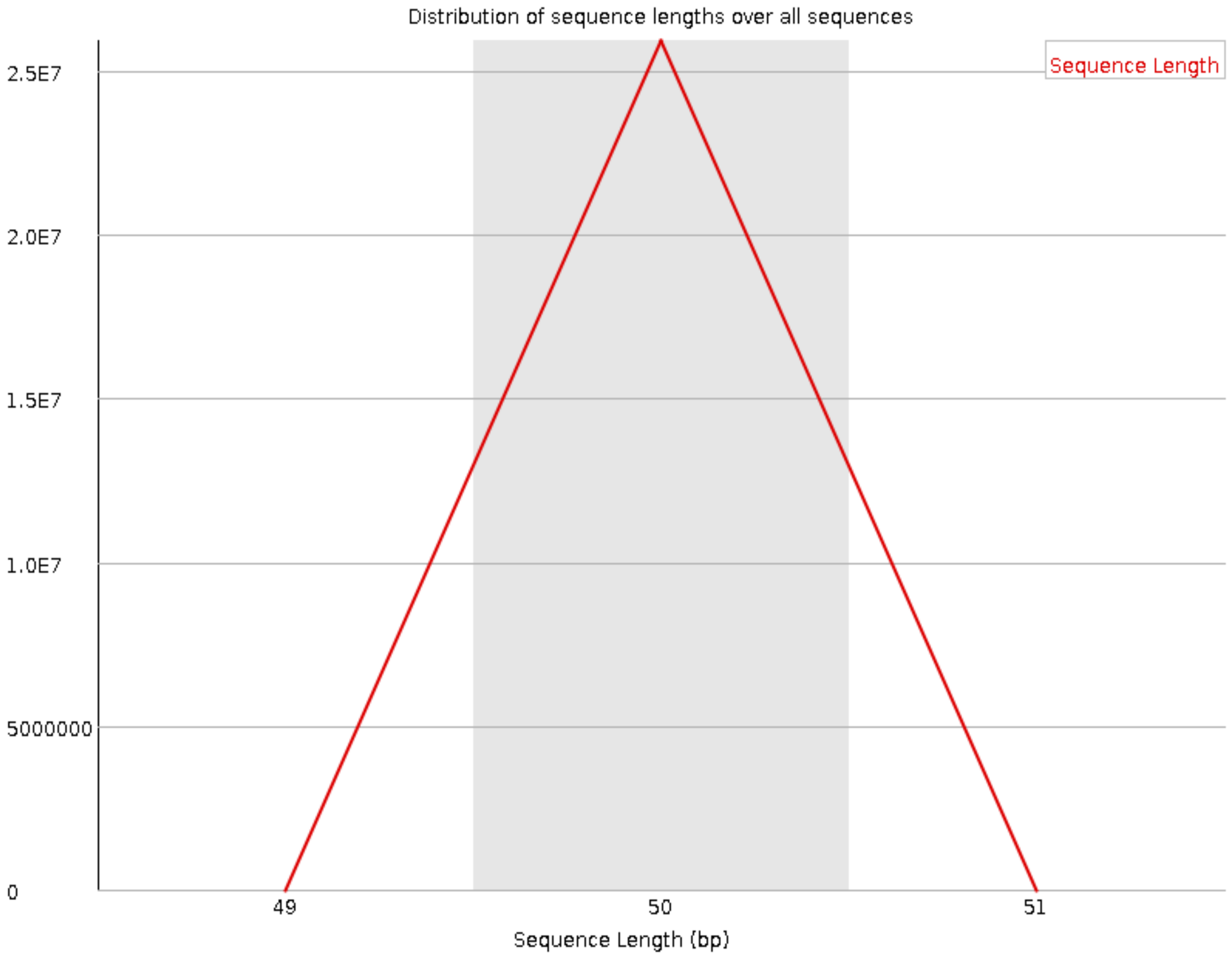

Sequence Duplication Levels

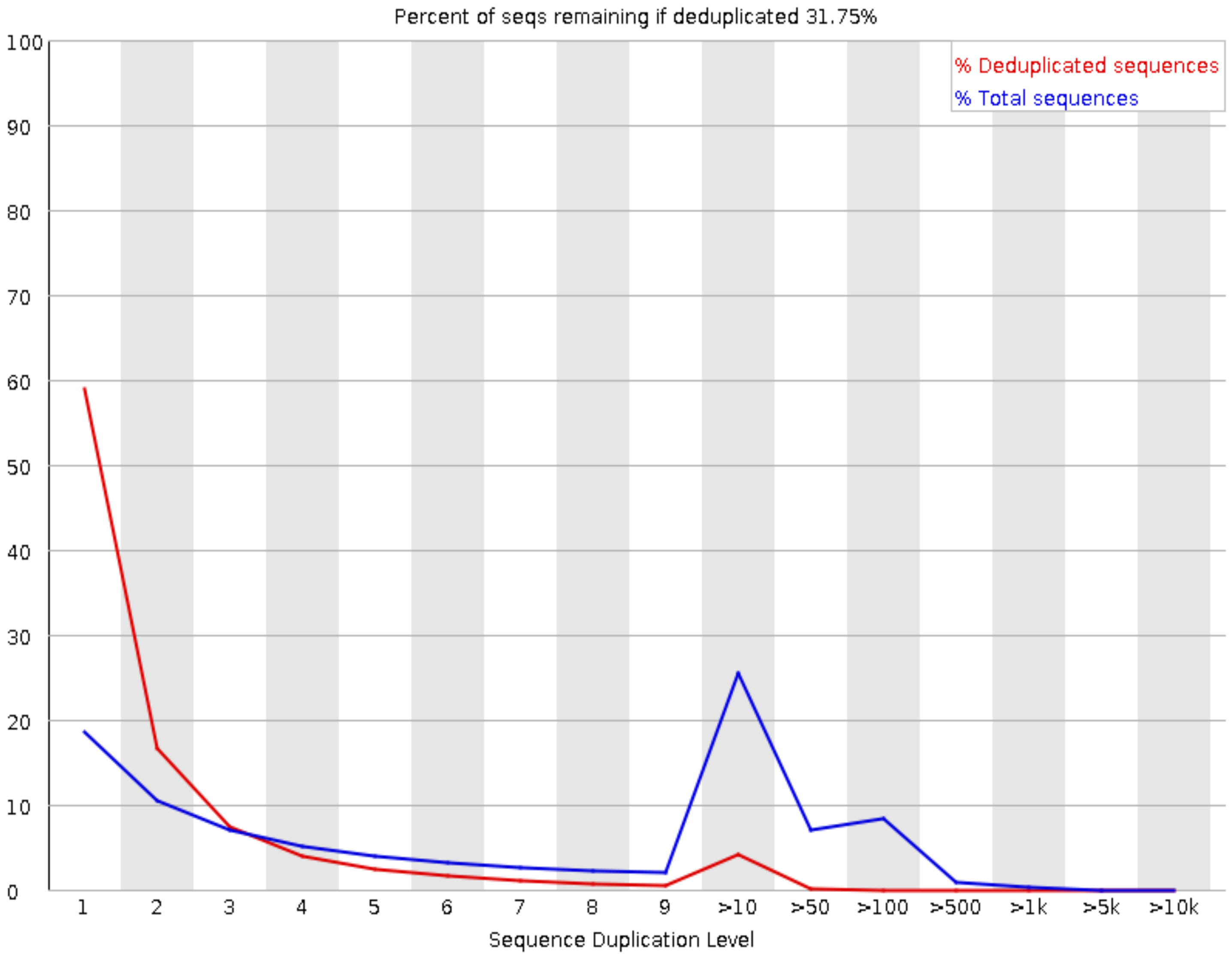

Overrepresented sequences

No overrepresented sequences

Adapter Content

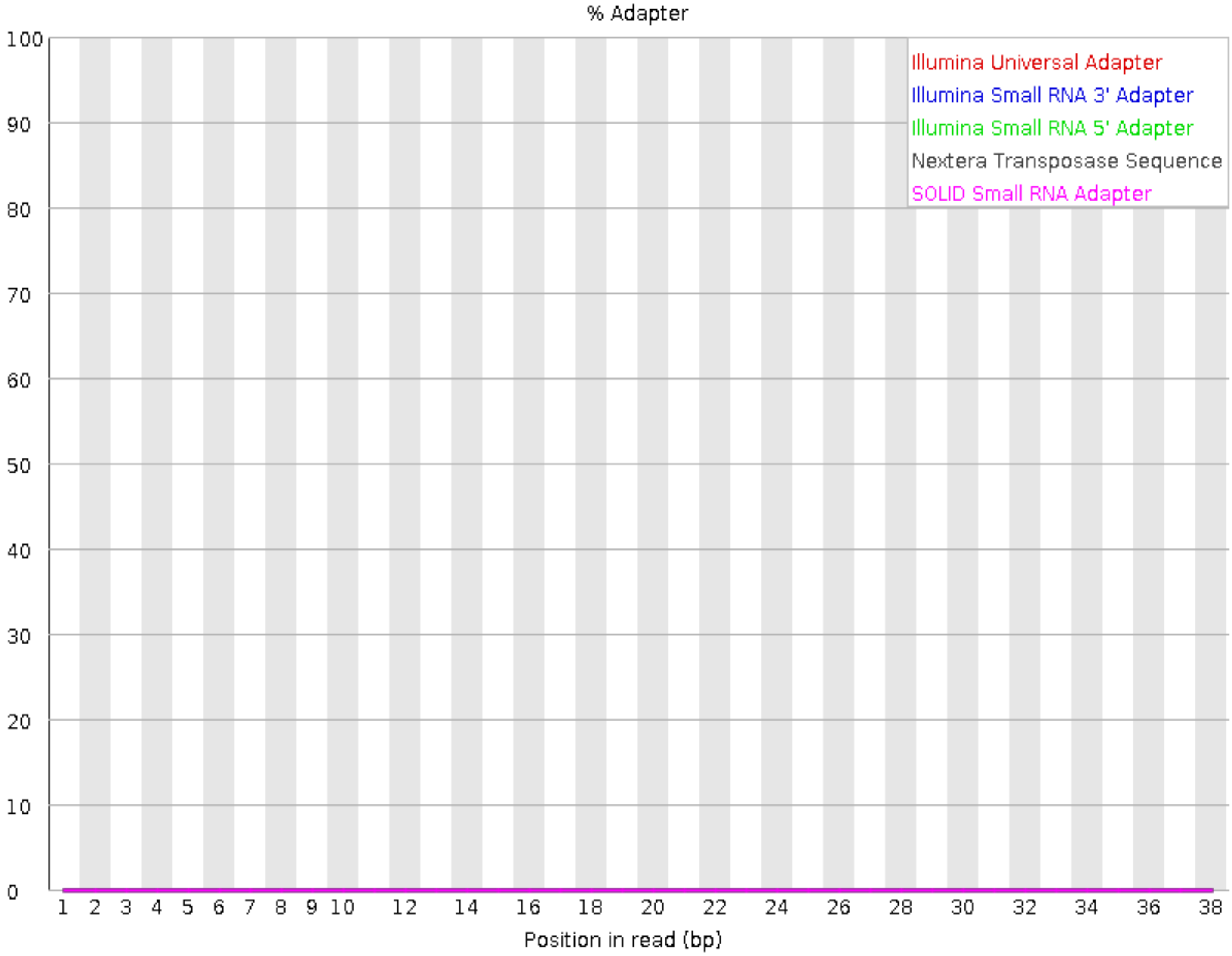

Kmer Content

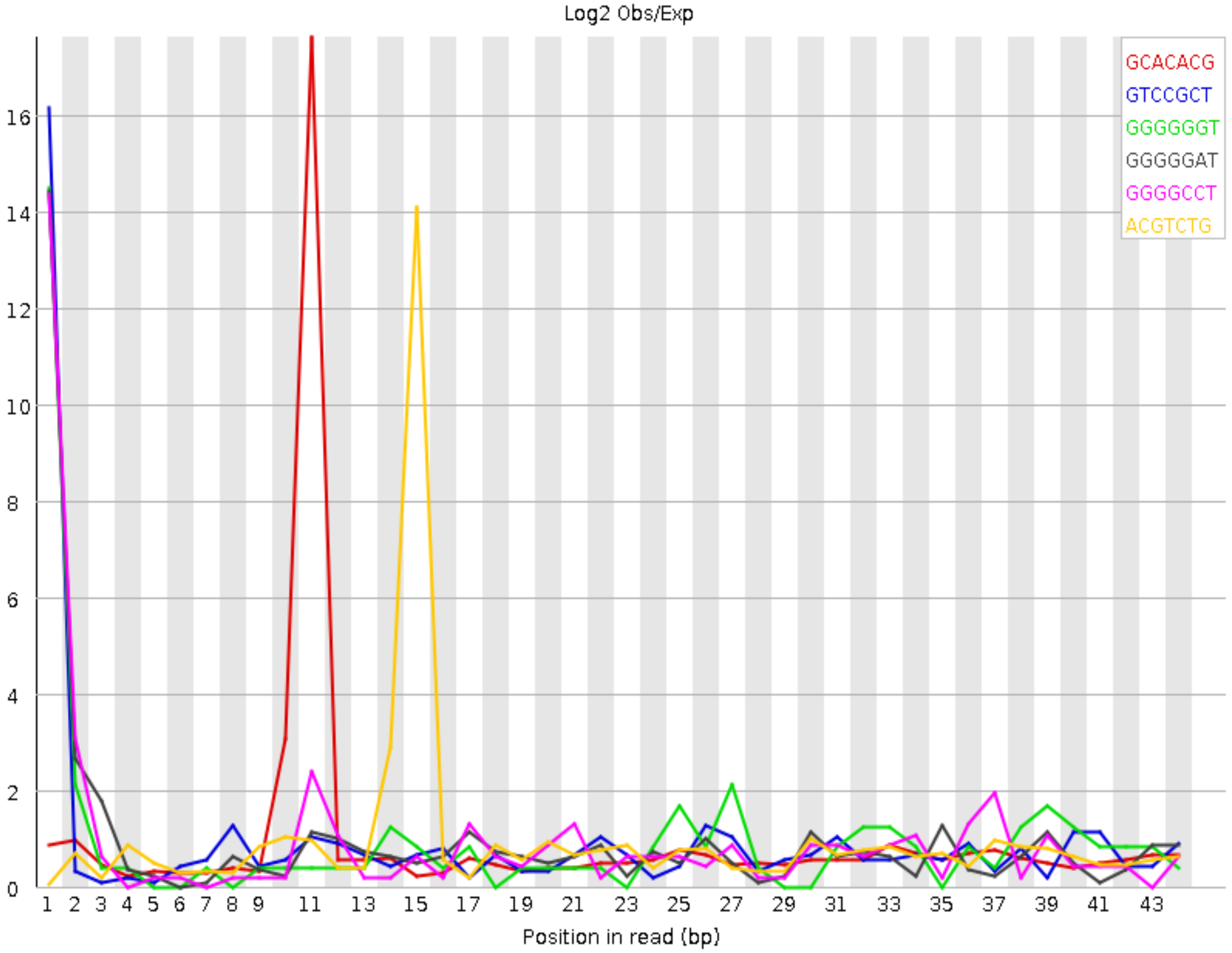

Sequence	Count	PValue	Obs/Exp Max	Max Obs/Exp Position
GCACACG	4170	0.0	17.61631	11
GTCCGCT	1875	0.0	16.18919	1
GGGGGGT	515	0.0	14.52175	1
GGGGGAT	1705	0.0	14.449104	1
GGGGCCT	995	0.0	14.369365	1
ACGTCTG	5315	0.0	14.111312	15
GGGGGCT	1825	0.0	13.981133	1
CACACGT	5410	0.0	13.619214	12
CCCCGCT	1485	0.0	13.331018	1
TATCTCG	5525	0.0	13.314099	41
GTCCGCA	1535	0.0	13.183379	1
GTCCGAT	3730	0.0	13.03259	1
GGGGGGA	850	0.0	12.93893	1

Sequence	Count	PValue	Obs/Exp Max	Max Obs/Exp Position
GCCCTAT	1085	0.0	12.569246	1
ACACGTC	6145	0.0	12.276538	13
CGTATCT	6310	0.0	12.070035	39
GTCCGTT	5425	0.0	11.920511	1
AGCACAC	6655	0.0	11.69925	10
CTCGTAT	6655	0.0	11.549881	44
GTCGGCT	3070	0.0	11.535457	1

Produced by FastQC (version 0.11.5)

FastQC Report

Tue 12 Sep 2017
22011_GTGAAA_L001_R1.fastq.gz

Summary

- Basic Statistics
- Per base sequence quality
- Per tile sequence quality
- Per sequence quality scores
- Per base sequence content
- Per sequence GC content
- Per base N content
- Sequence Length Distribution
- Sequence Duplication Levels
- Overrepresented sequences
- Adapter Content
- Kmer Content

Basic Statistics

Measure	Value
Filename	22011_GTGAAA_L001_R1_001.fastq.gz
File type	Conventional base calls
Encoding	Sanger / Illumina 1.9
Total Sequences	24878923
Sequences flagged as poor quality	0
Sequence length	50
%GC	46

Per base sequence quality

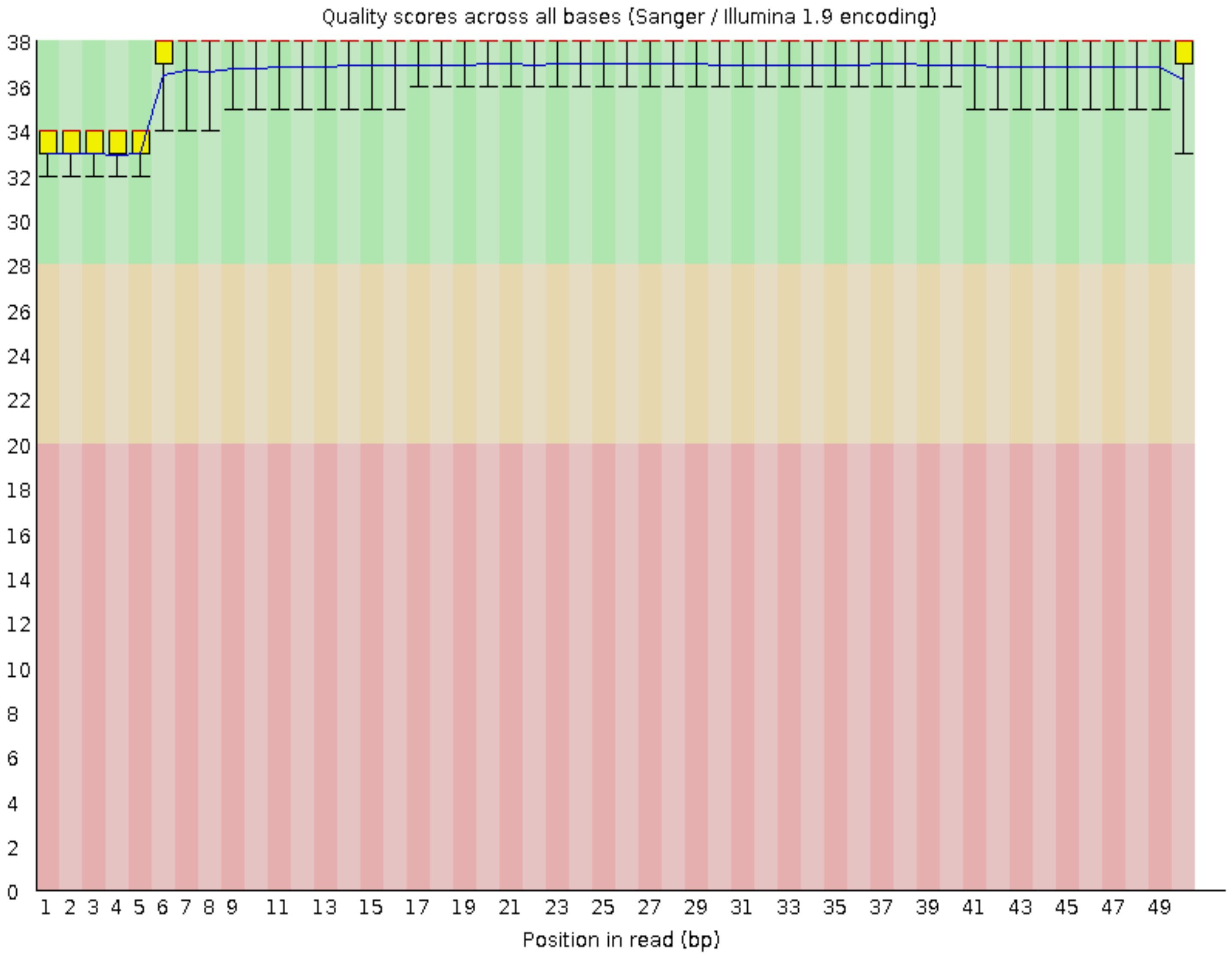

Per tile sequence quality

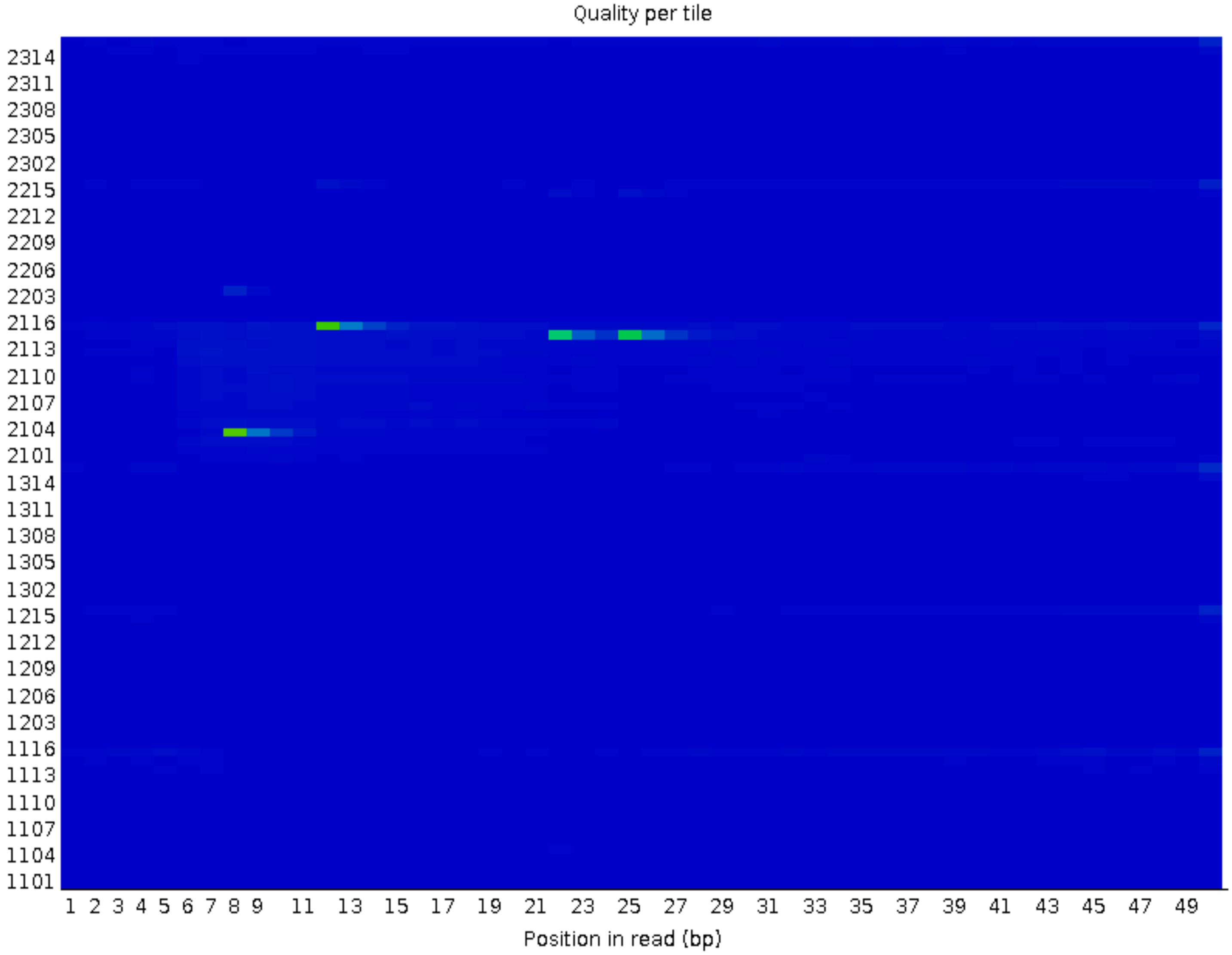

Per sequence quality scores

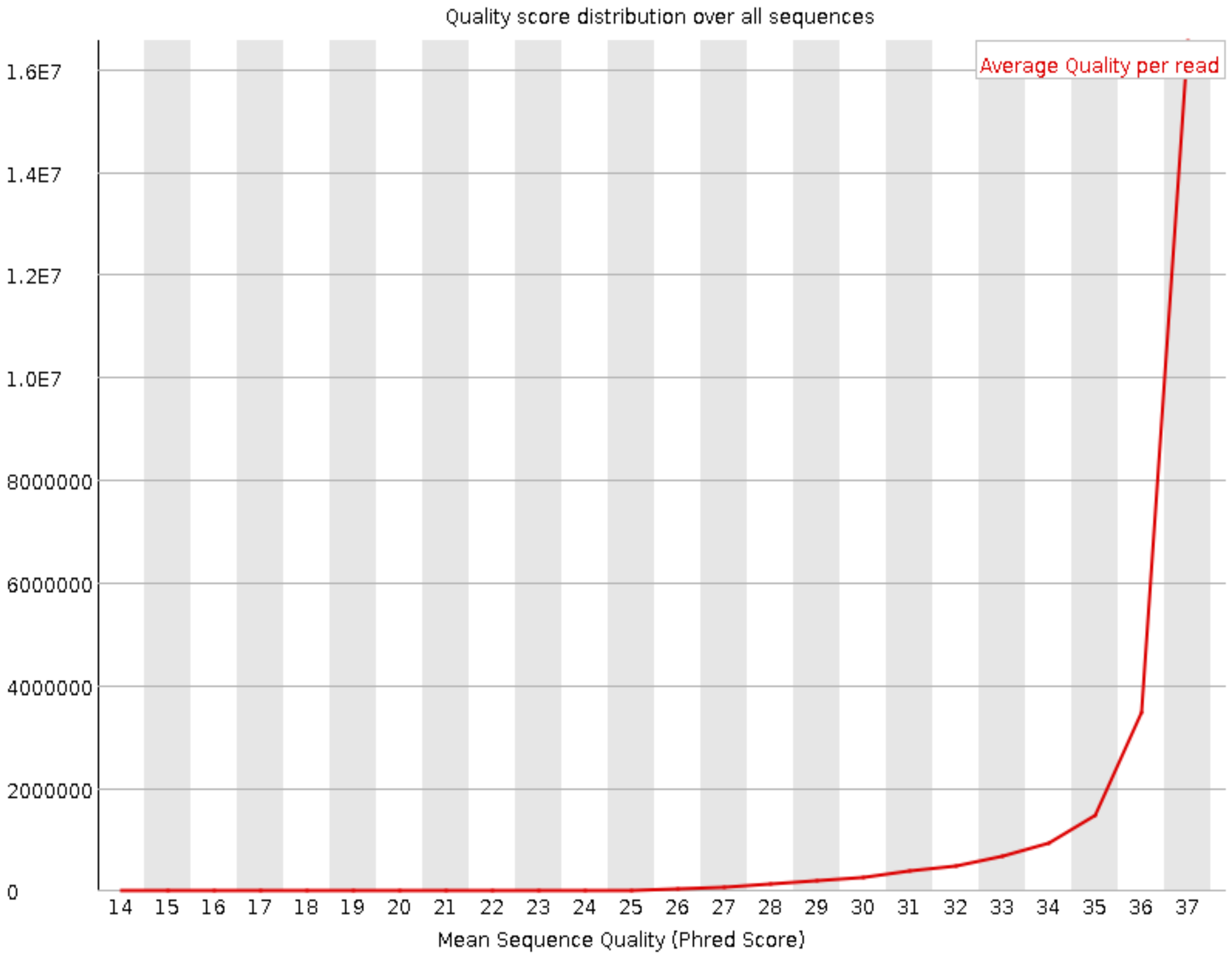

Per base sequence content

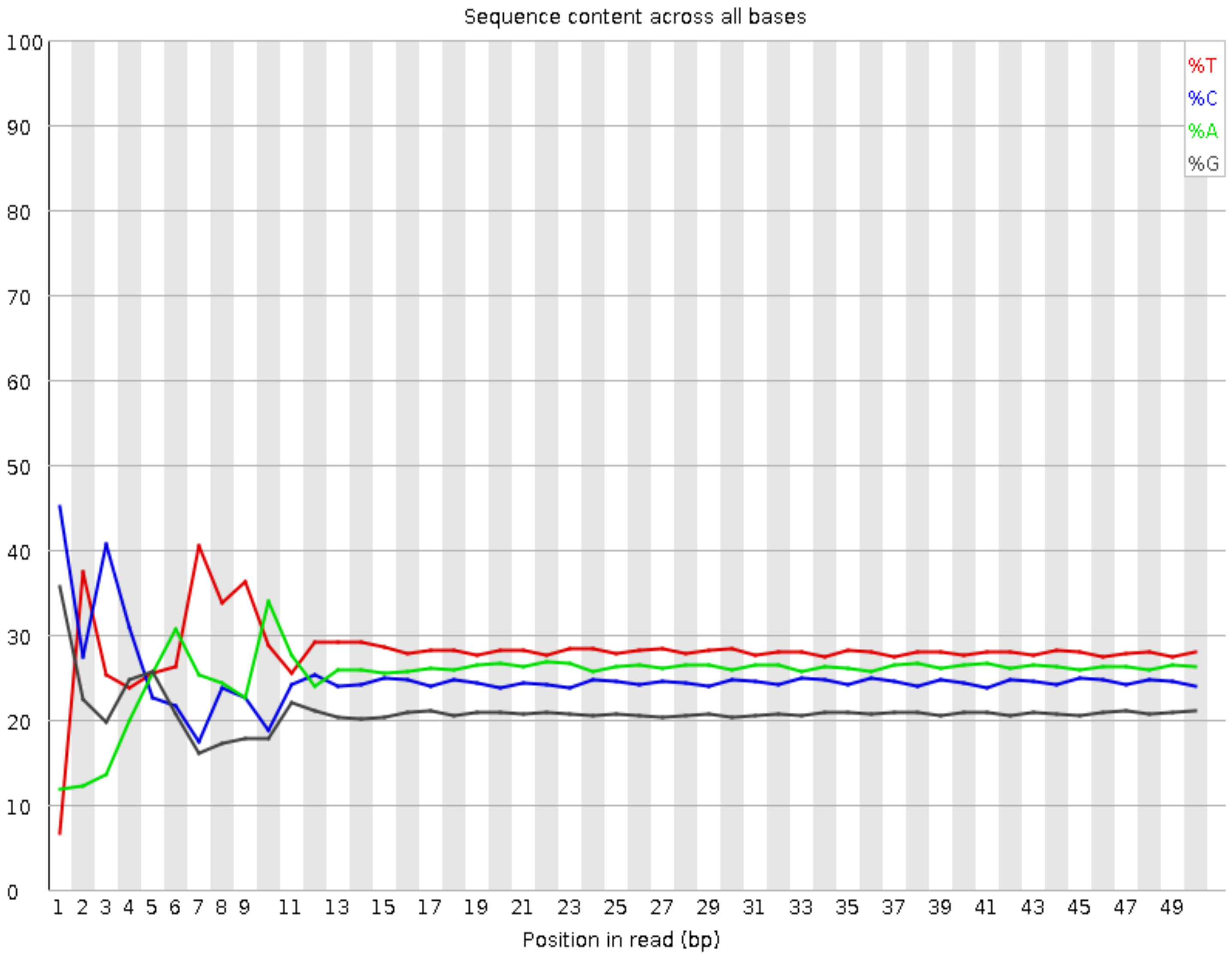

Per sequence GC content

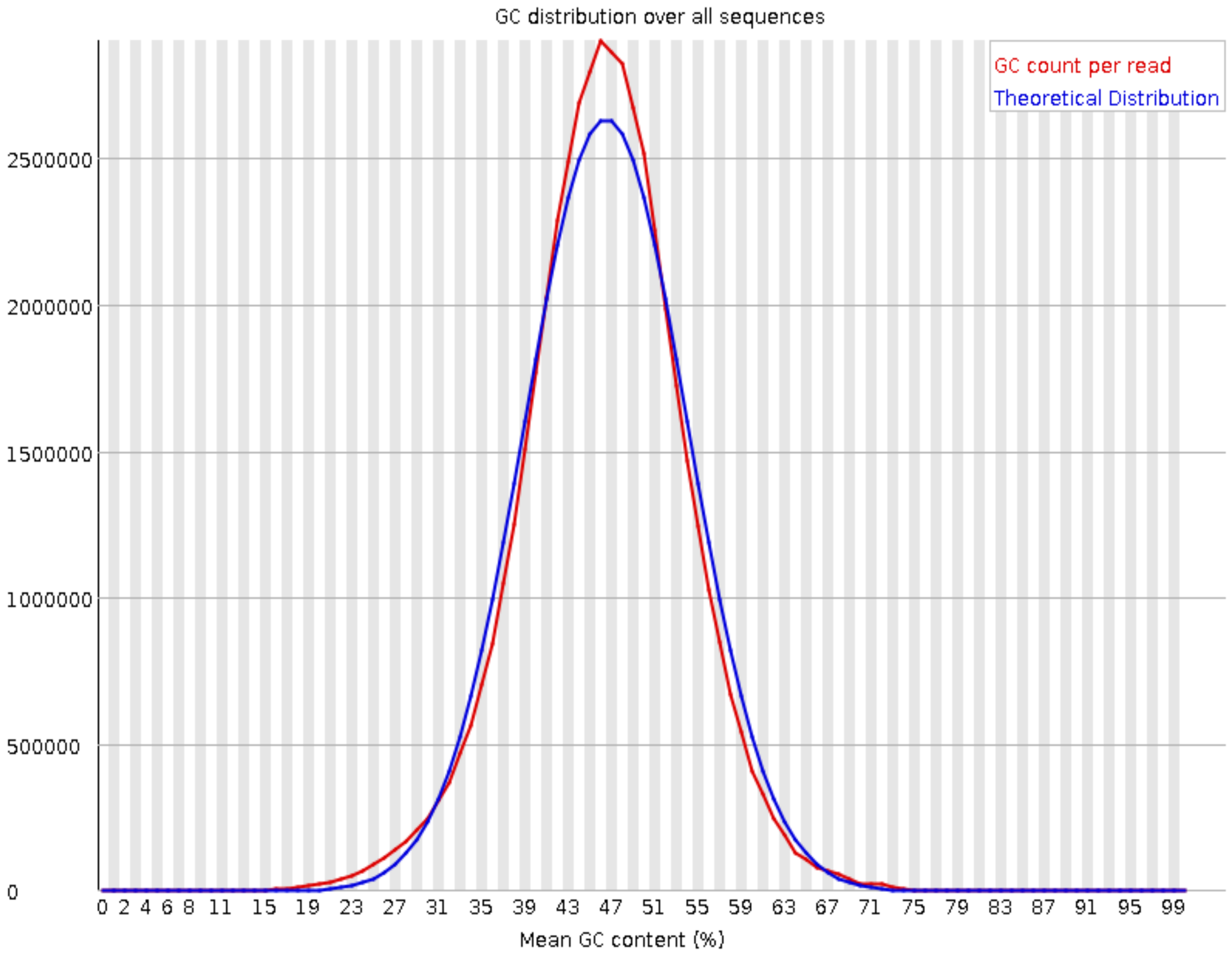

Per base N content

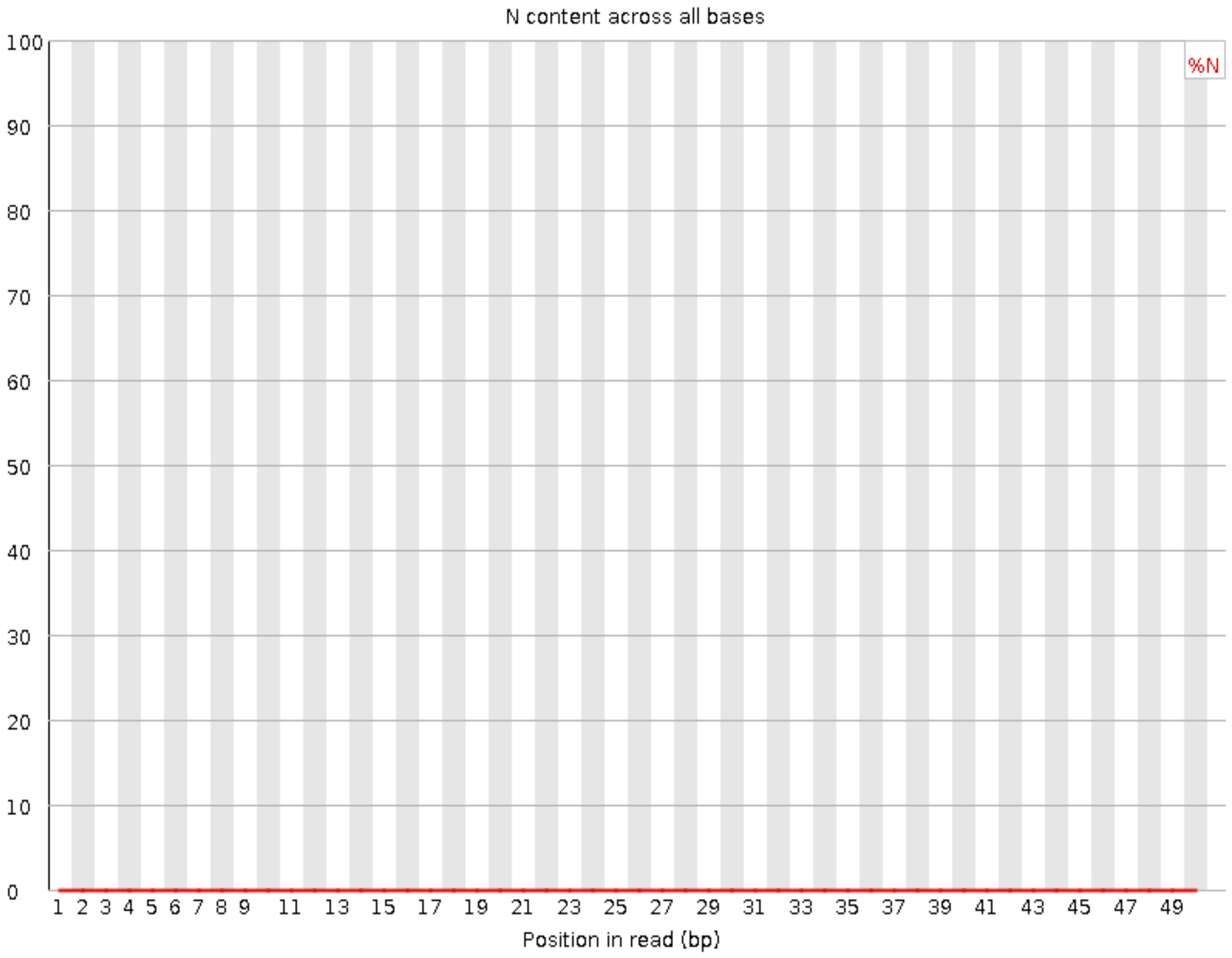

Sequence Length Distribution

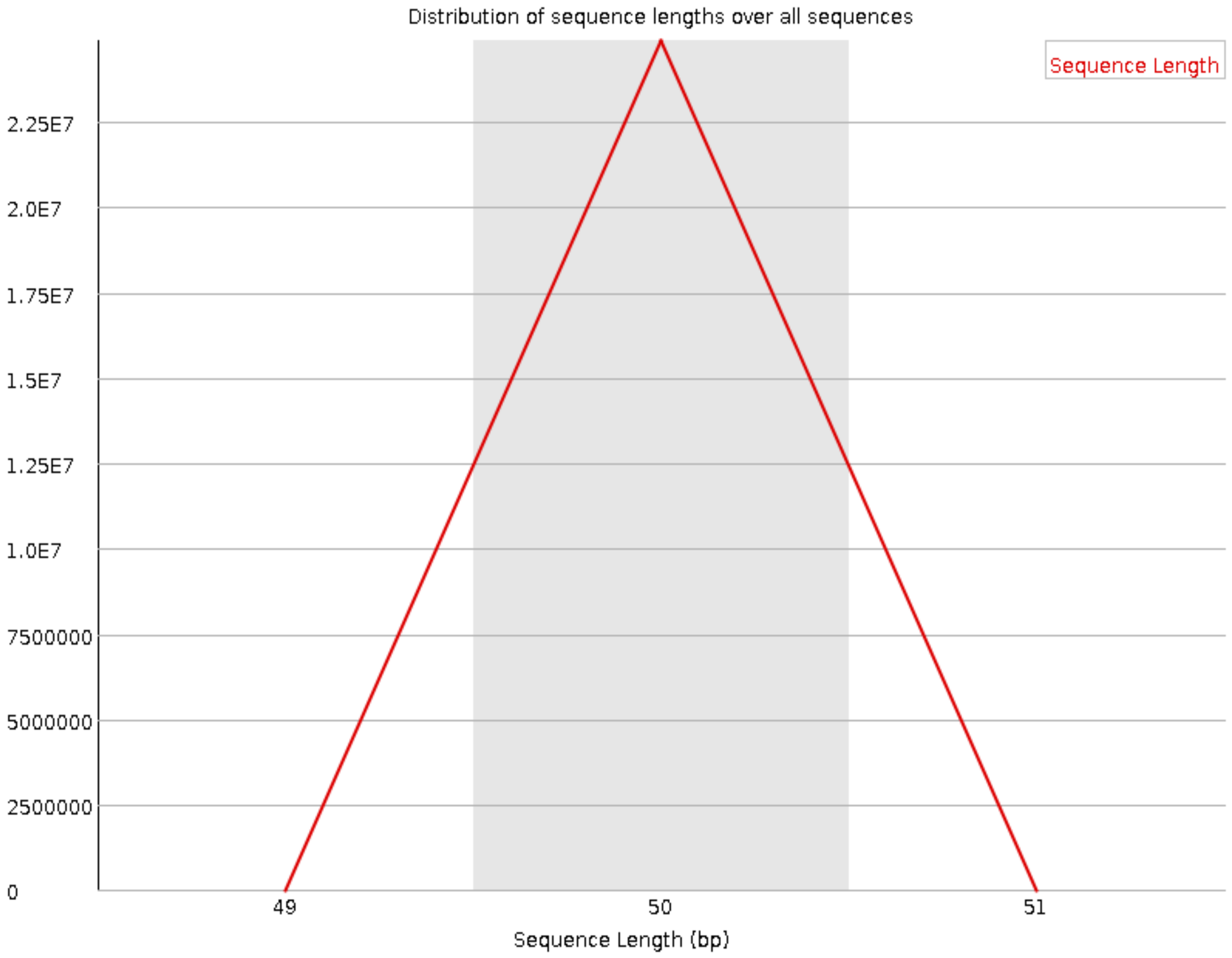

Sequence Duplication Levels

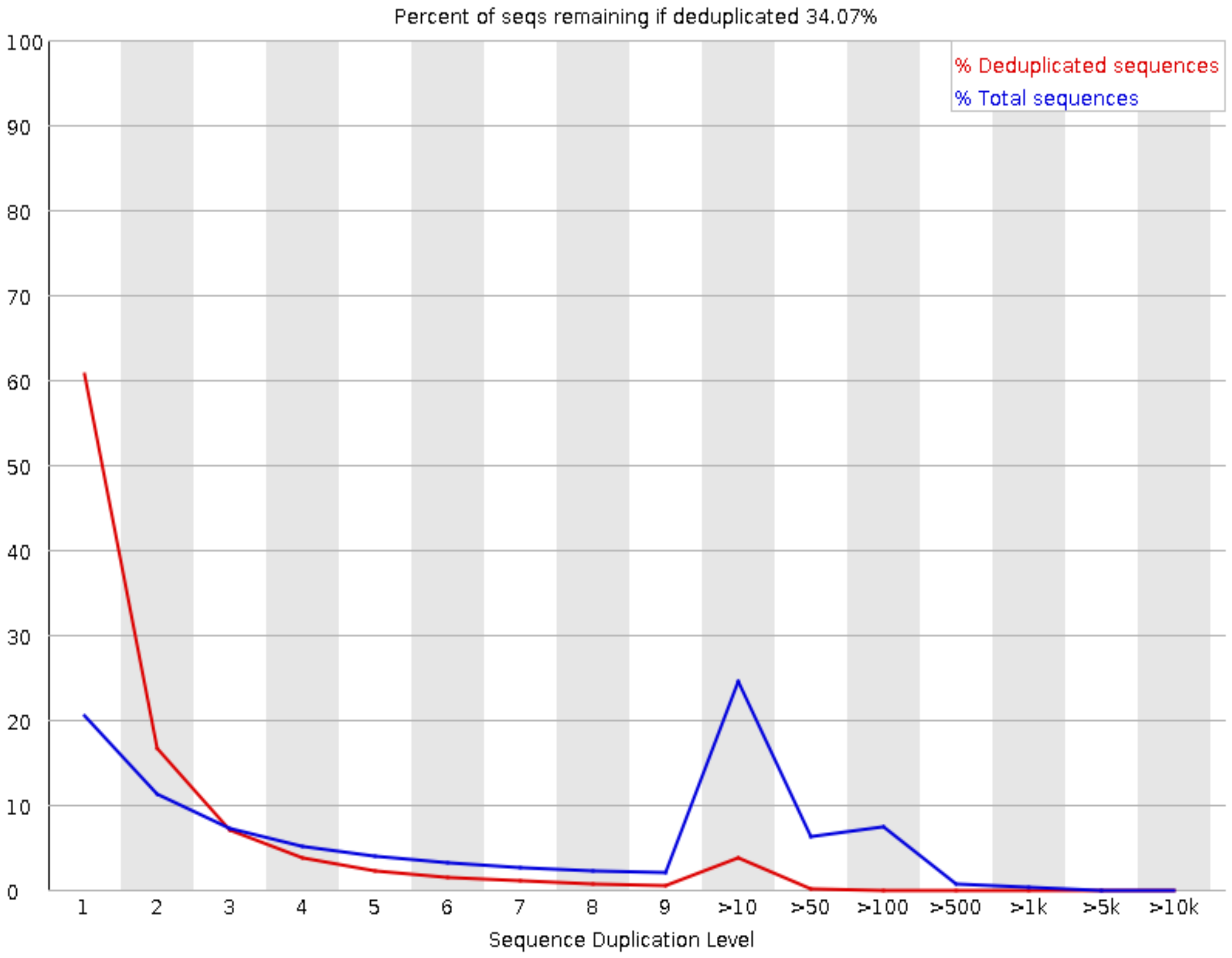

Overrepresented sequences

No overrepresented sequences

Adapter Content

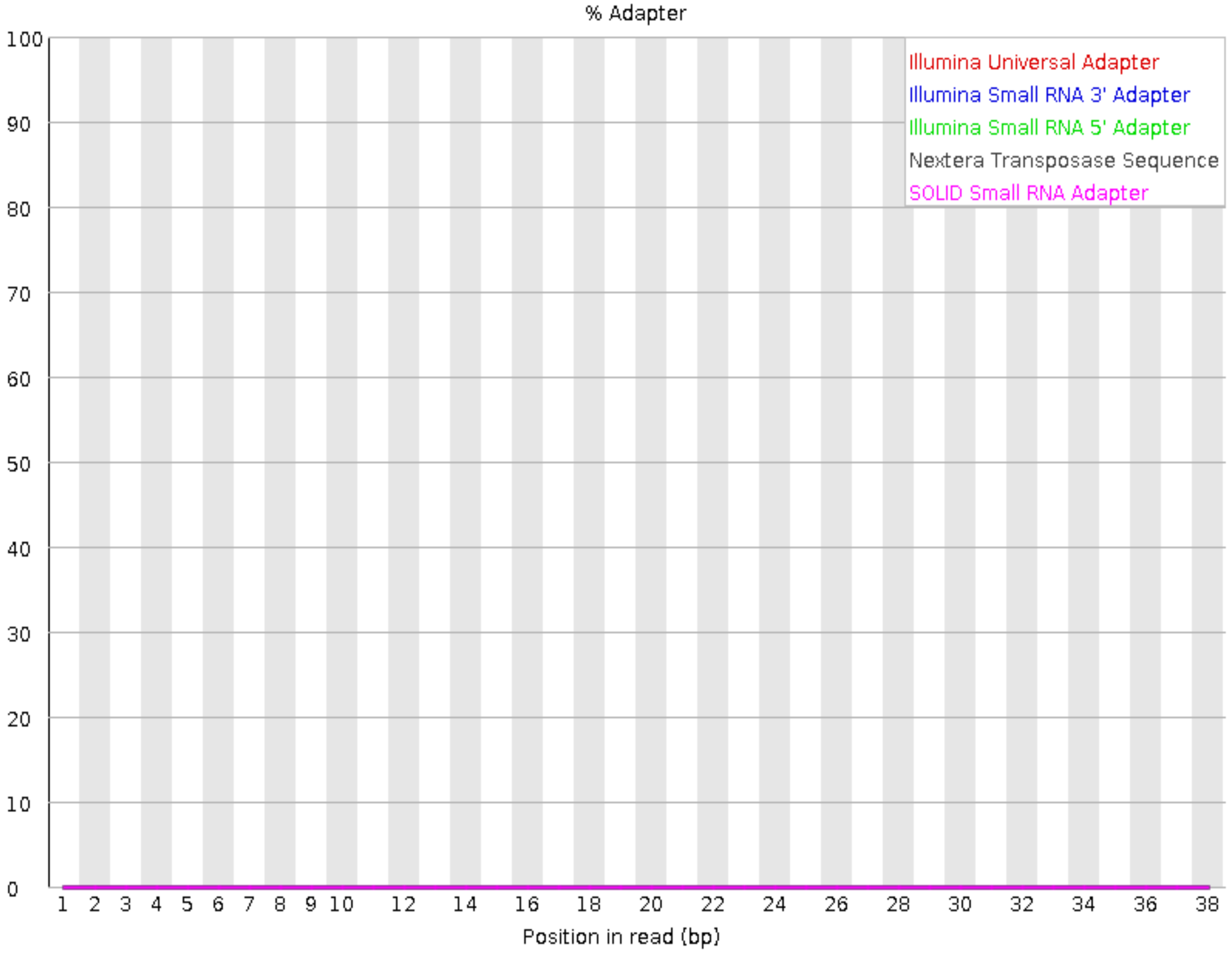

Kmer Content

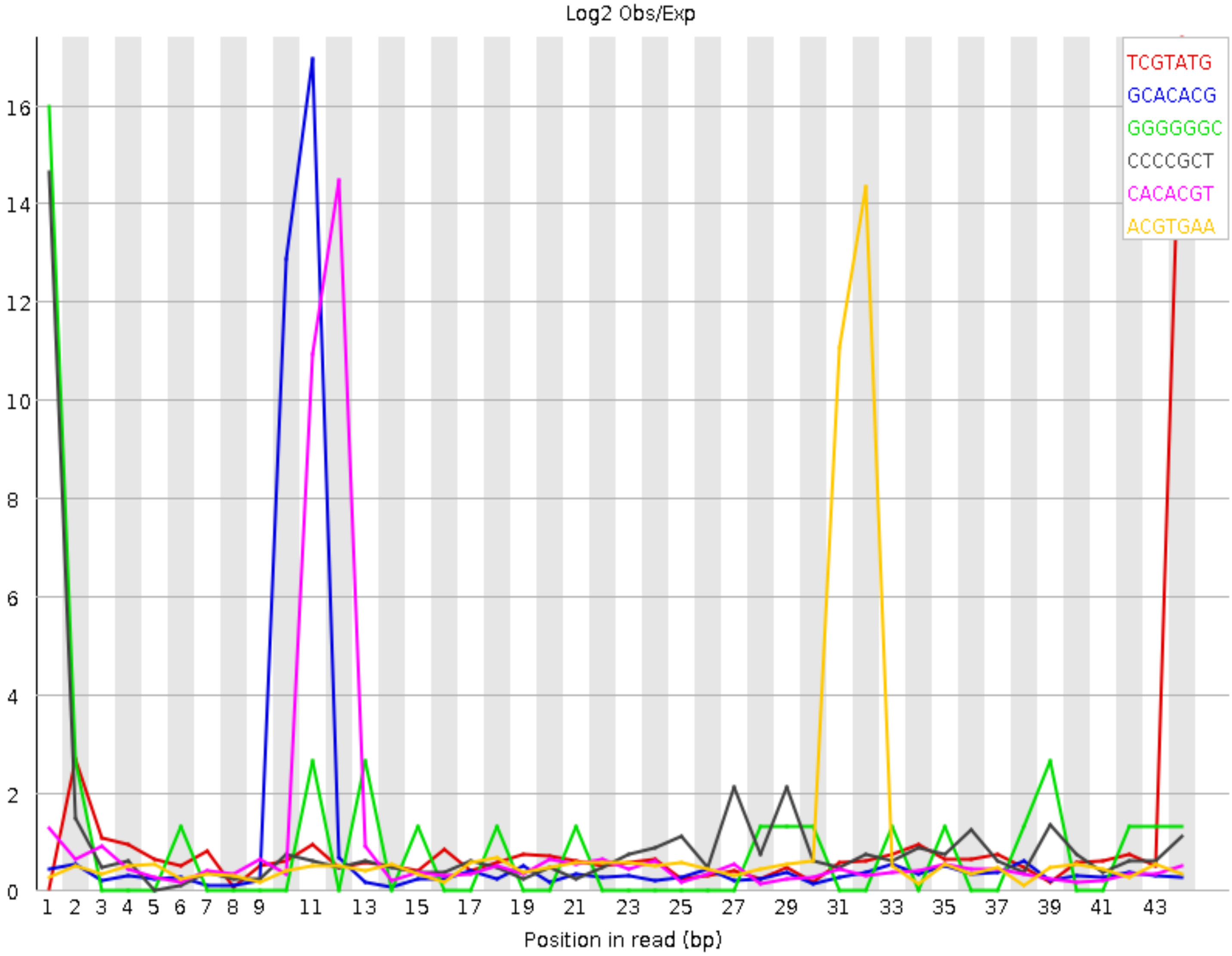

Sequence	Count	PValue	Obs/Exp Max	Max Obs/Exp Position
TCGTATG	4640	0.0	17.372032	44
GCACACG	6605	0.0	16.949297	11
GGGGGGC	165	3.124842E-8	15.997465	1
CCCCGCT	1770	0.0	14.664344	1
CACACGT	7850	0.0	14.485305	12
ACGTGAA	7865	0.0	14.346483	32
ACGTCTG	8005	0.0	14.067788	15
GTCACGT	8095	0.0	13.993233	29
GCCCTAT	1070	0.0	13.773519	1
CCCCGGT	1645	0.0	13.772894	1
GTCCGCT	1860	0.0	13.718255	1
ACACGTC	8455	0.0	13.526844	13
CACGTGA	8630	0.0	13.329618	31

Sequence	Count	PValue	Obs/Exp Max	Max Obs/Exp Position
AGCACAC	8645	0.0	13.153226	10
GGGGGCC	320	3.6379788E-12	13.060431	1
AGTCACG	8980	0.0	13.032103	28
TCACGTG	9150	0.0	12.596126	30
CTCGTAT	9335	0.0	12.456819	44
GAGCACA	9395	0.0	12.24367	9
CCCCCCT	755	0.0	12.236472	1

Produced by FastQC (version 0.11.5)

FastQC Report

Tue 12 Sep 2017
22012_GAGTGG_L001_R1.fastq.gz

Summary

- Basic Statistics
- Per base sequence quality
- Per tile sequence quality
- Per sequence quality scores
- Per base sequence content
- Per sequence GC content
- Per base N content
- Sequence Length Distribution
- Sequence Duplication Levels
- Overrepresented sequences
- Adapter Content
- Kmer Content

Basic Statistics

Measure	Value
Filename	22012_GAGTGG_L001_R1_001.fastq.gz
File type	Conventional base calls
Encoding	Sanger / Illumina 1.9
Total Sequences	25284412
Sequences flagged as poor quality	0
Sequence length	50
%GC	46

Per base sequence quality

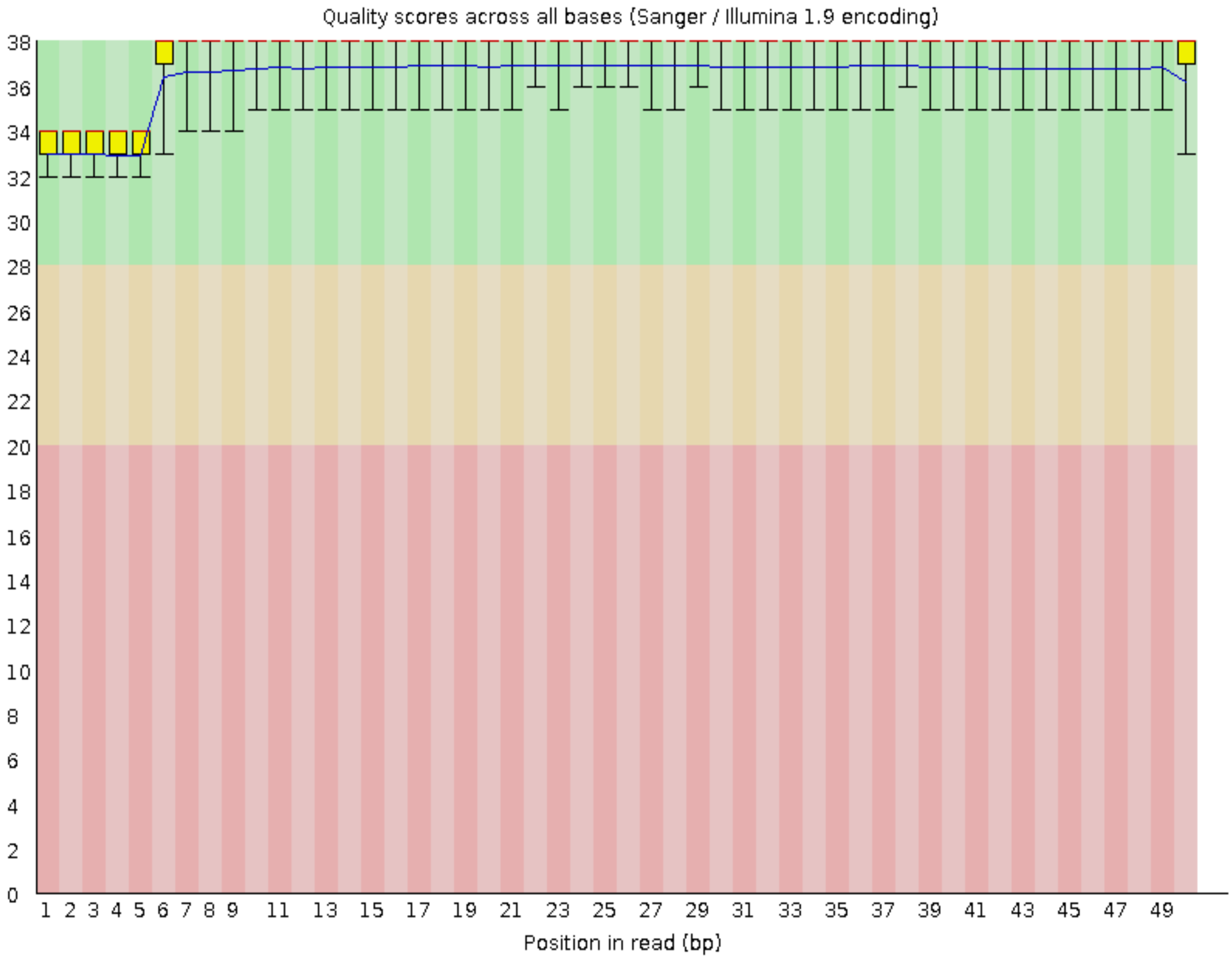

Per tile sequence quality

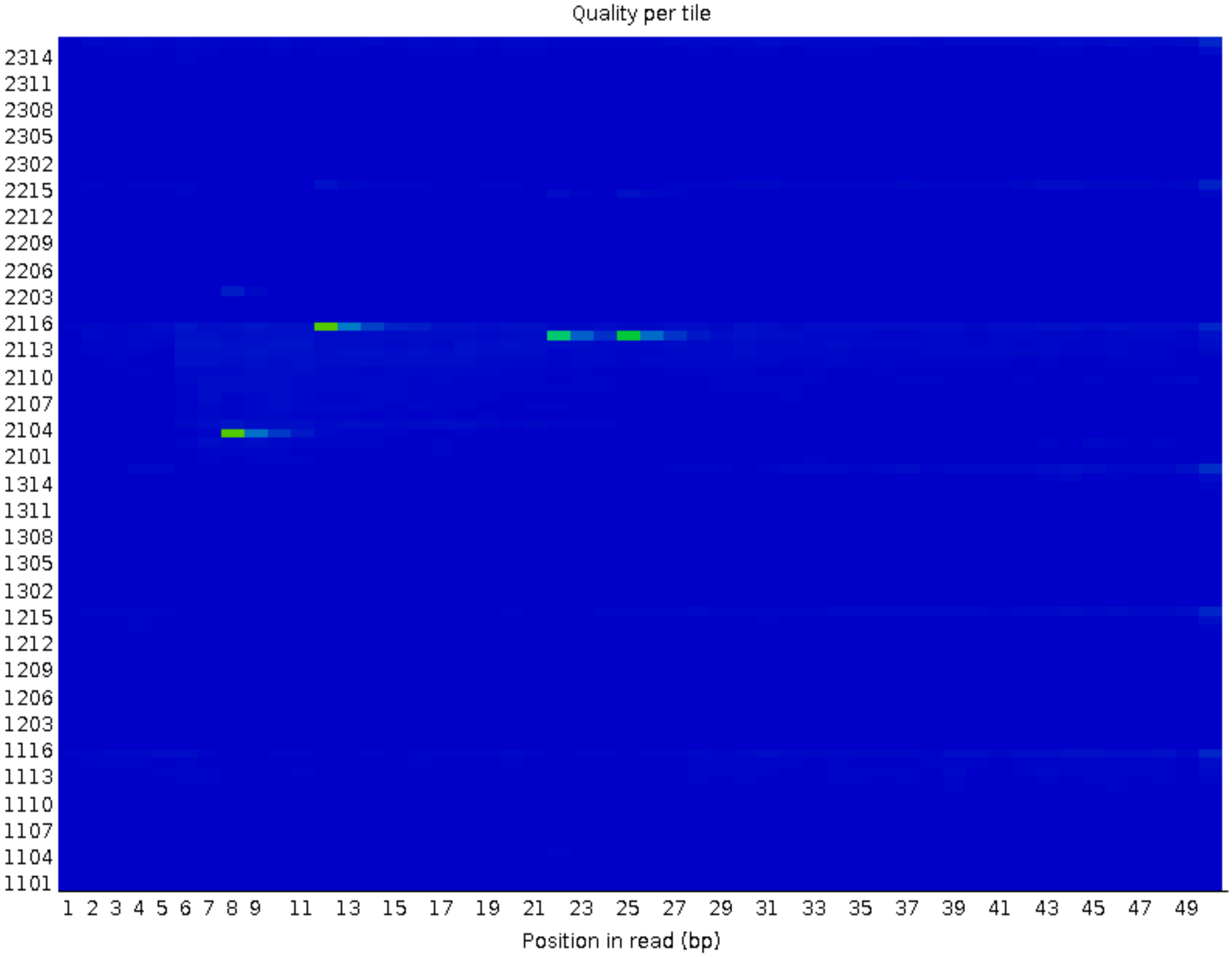

Per sequence quality scores

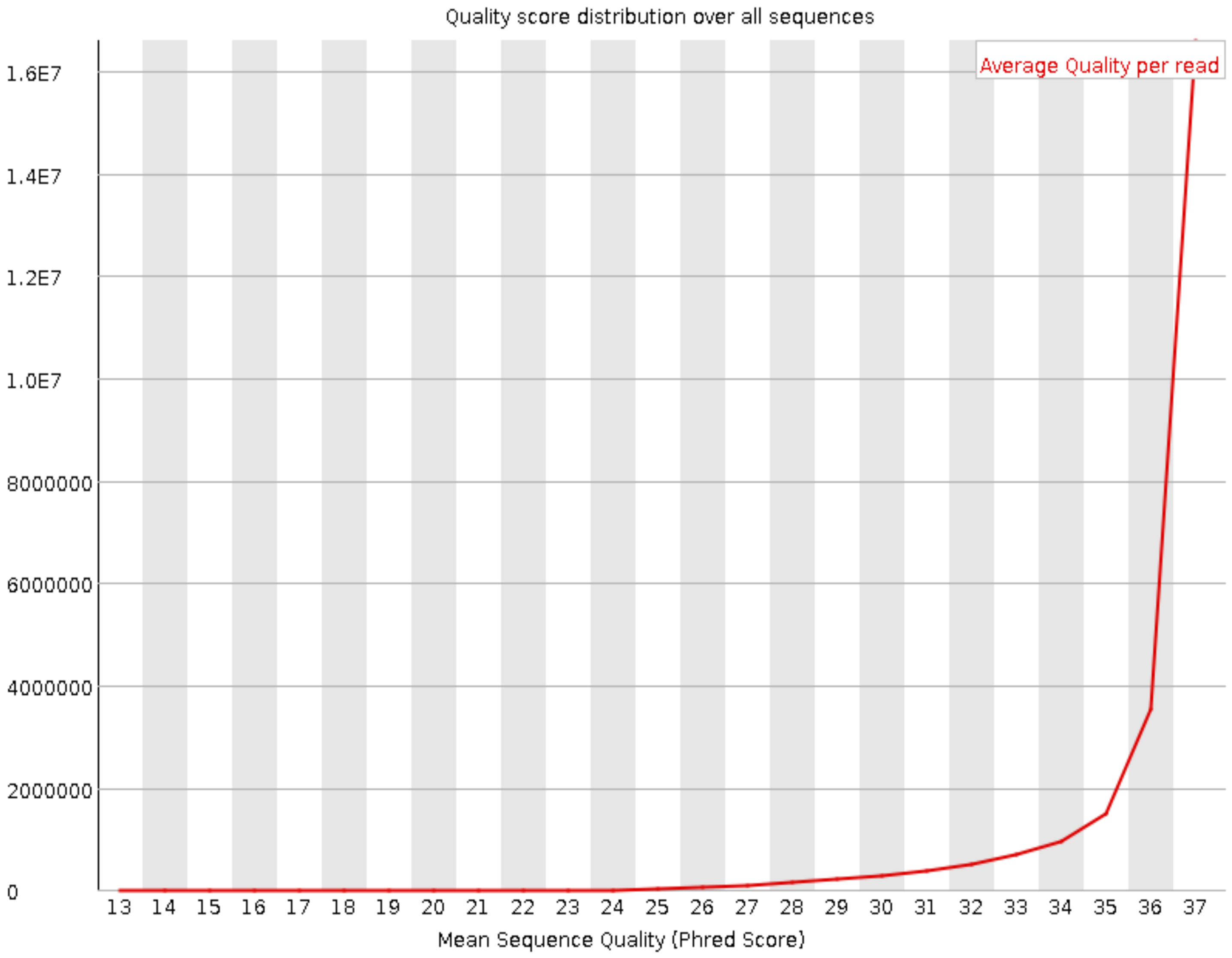

Per base sequence content

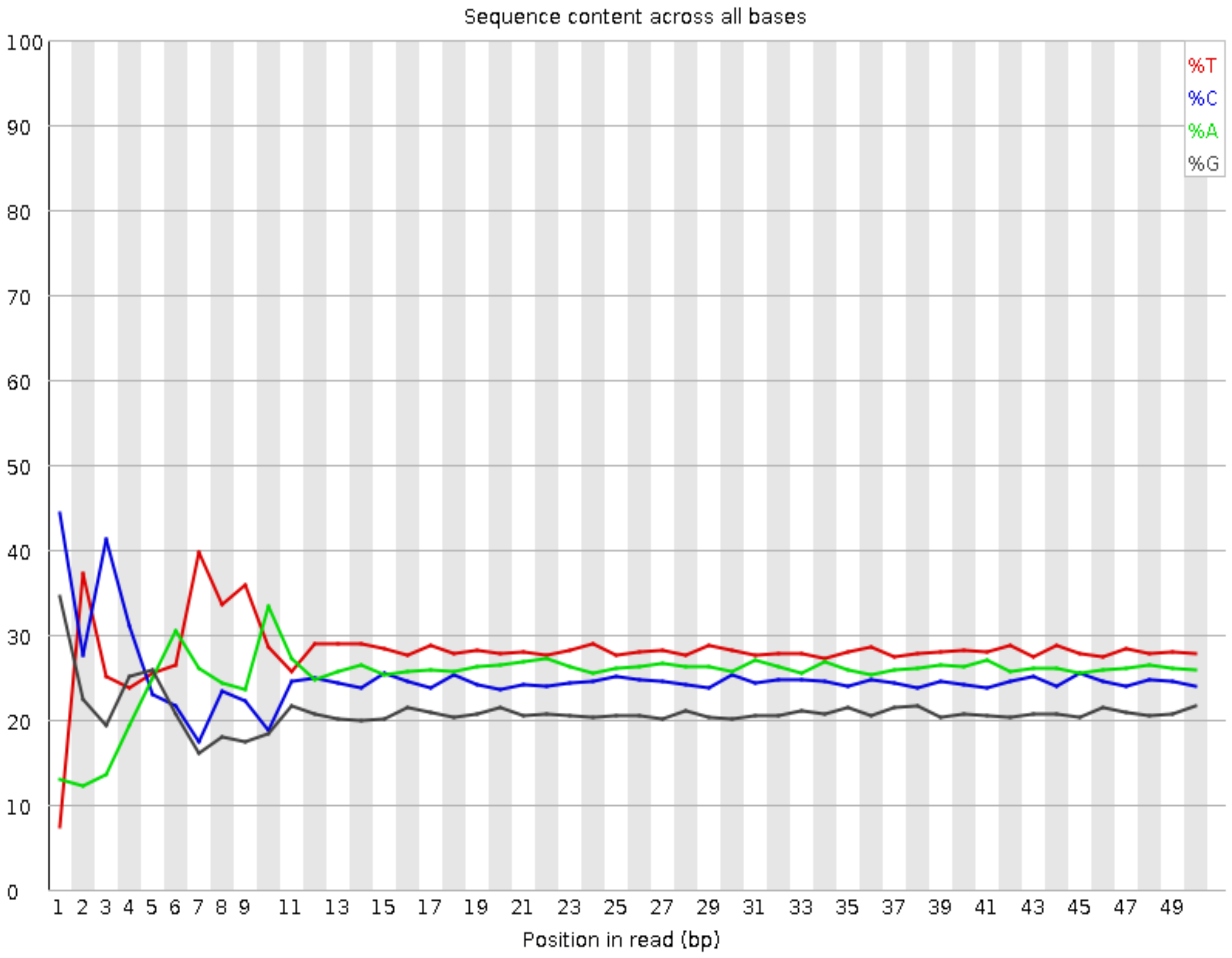

Per sequence GC content

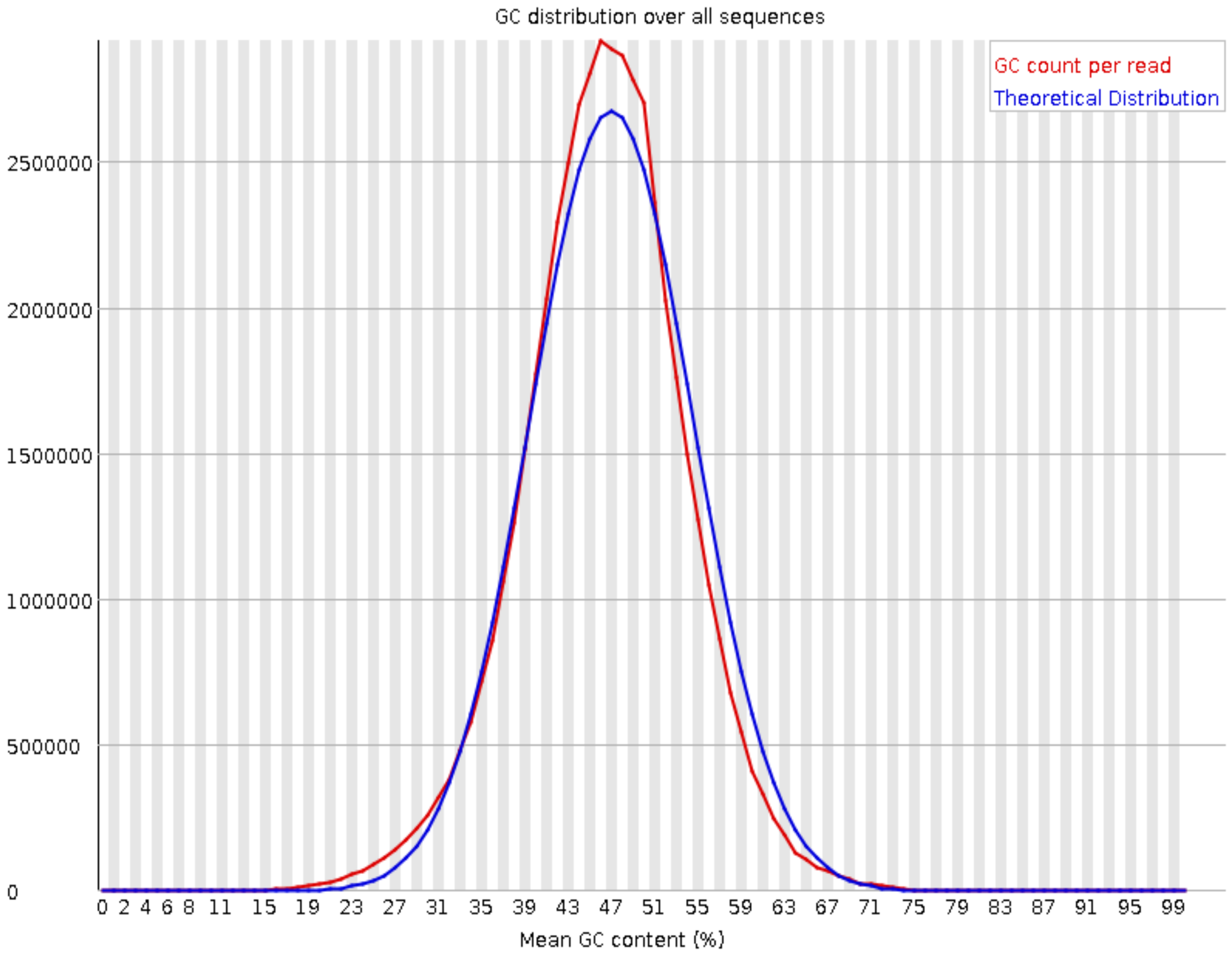

Per base N content

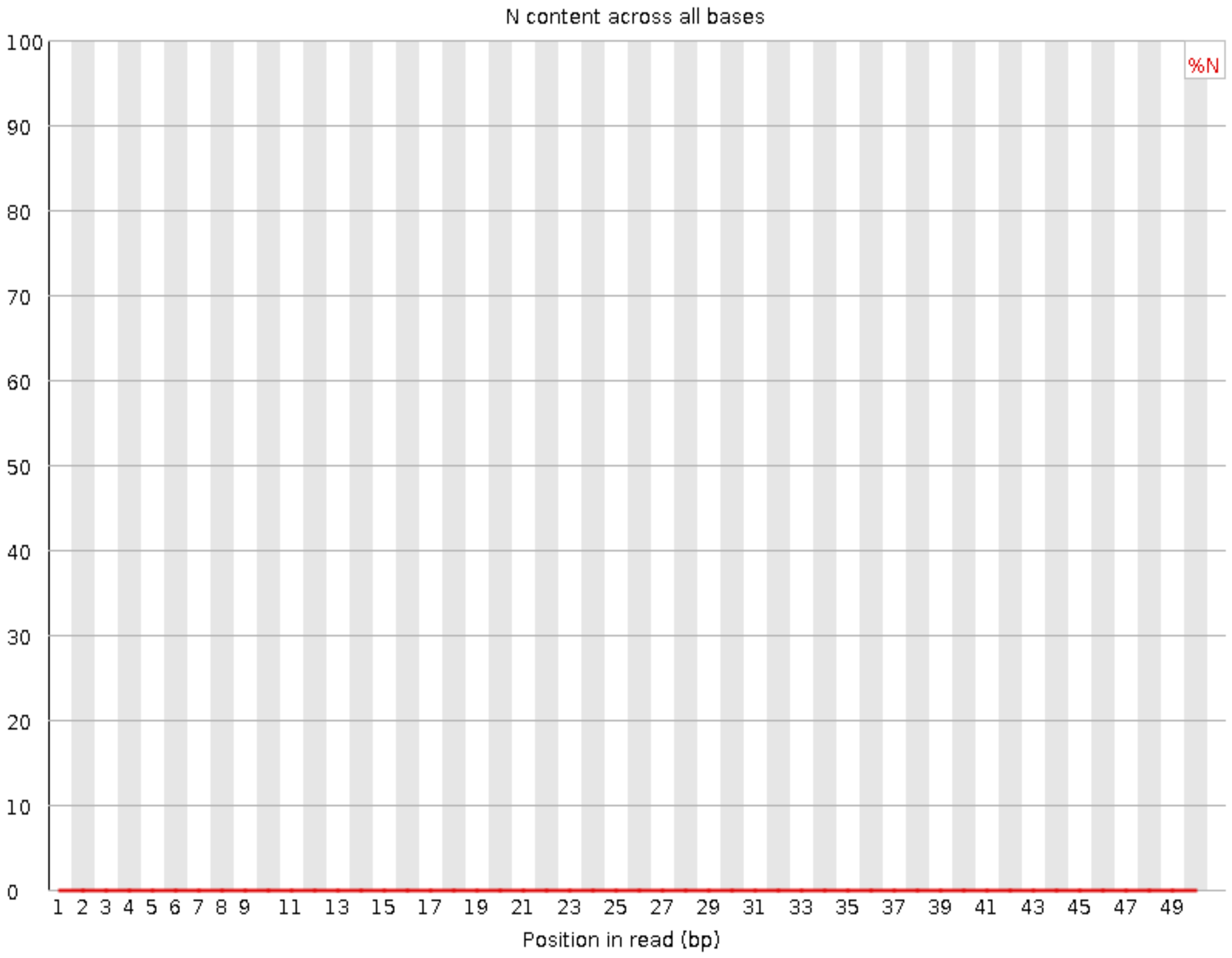

Sequence Length Distribution

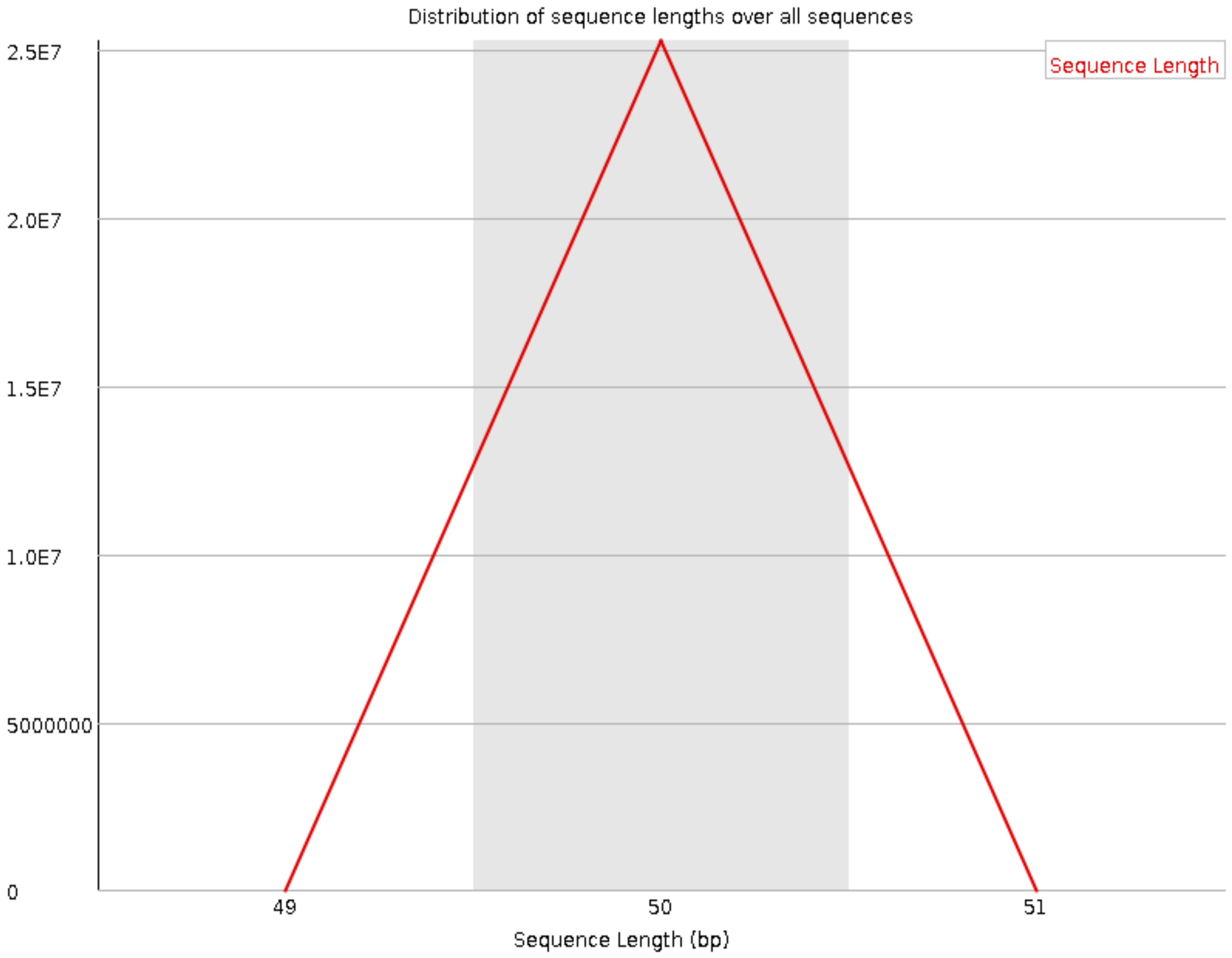

Sequence Duplication Levels

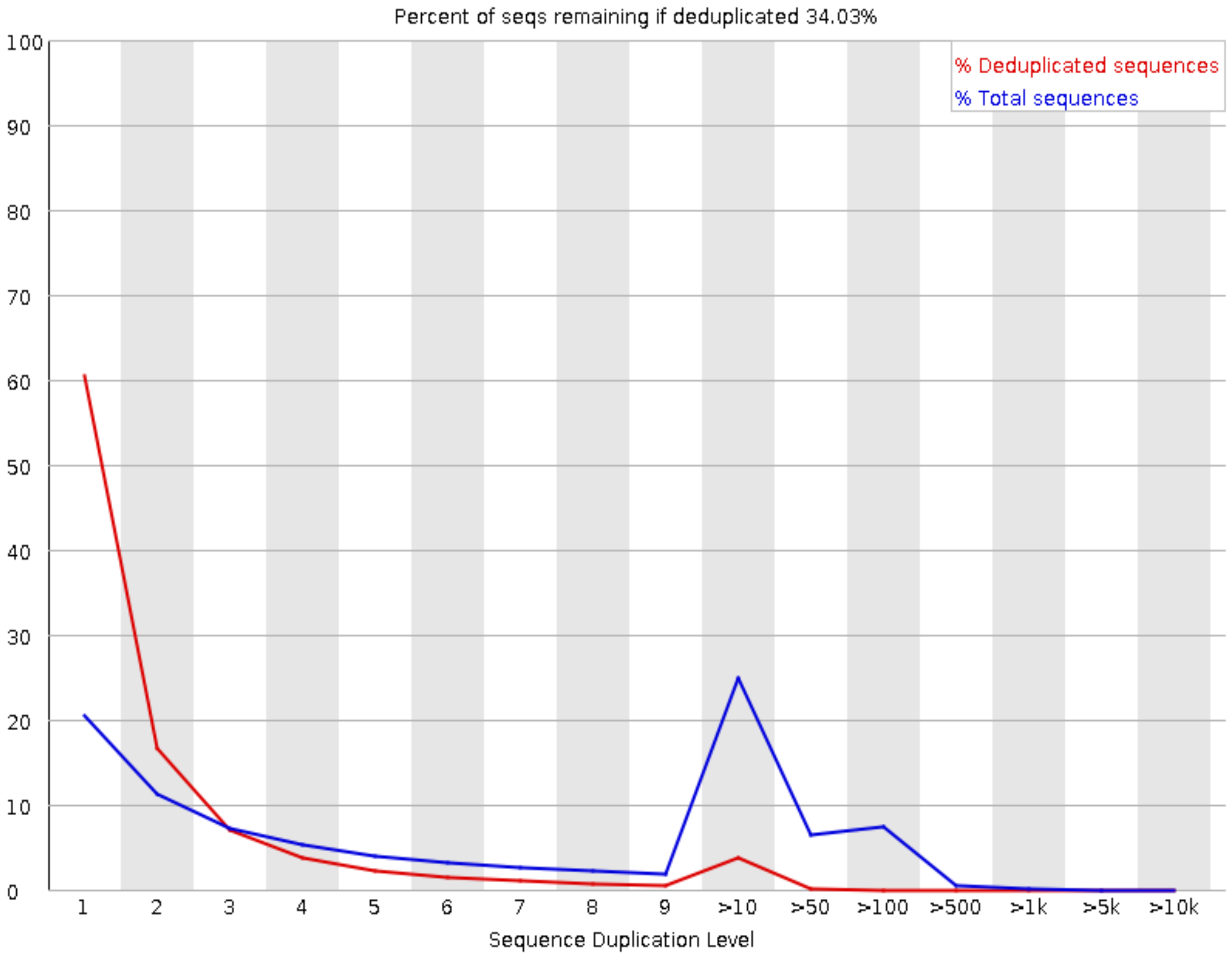

Overrepresented sequences

No overrepresented sequences

Adapter Content

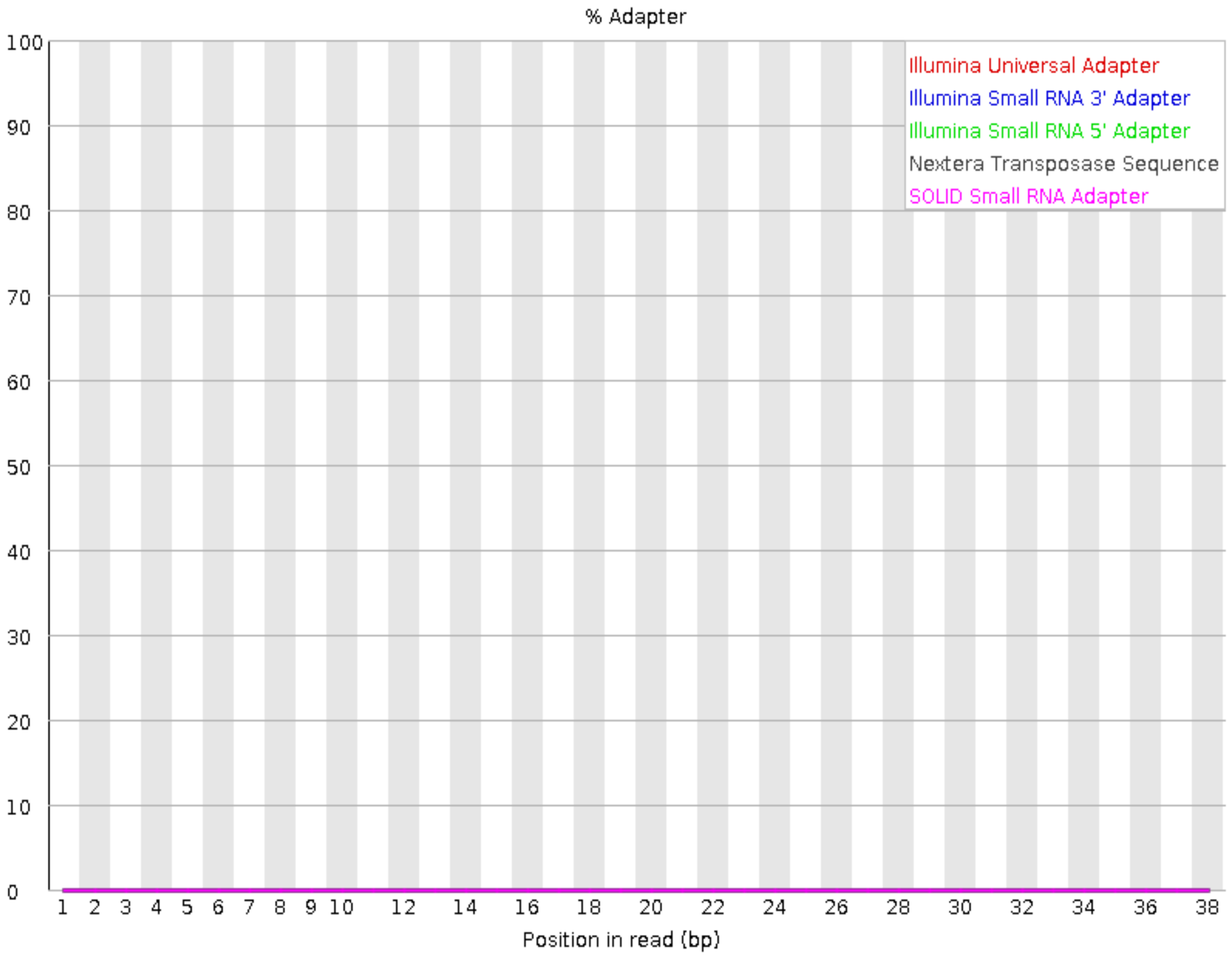

Kmer Content

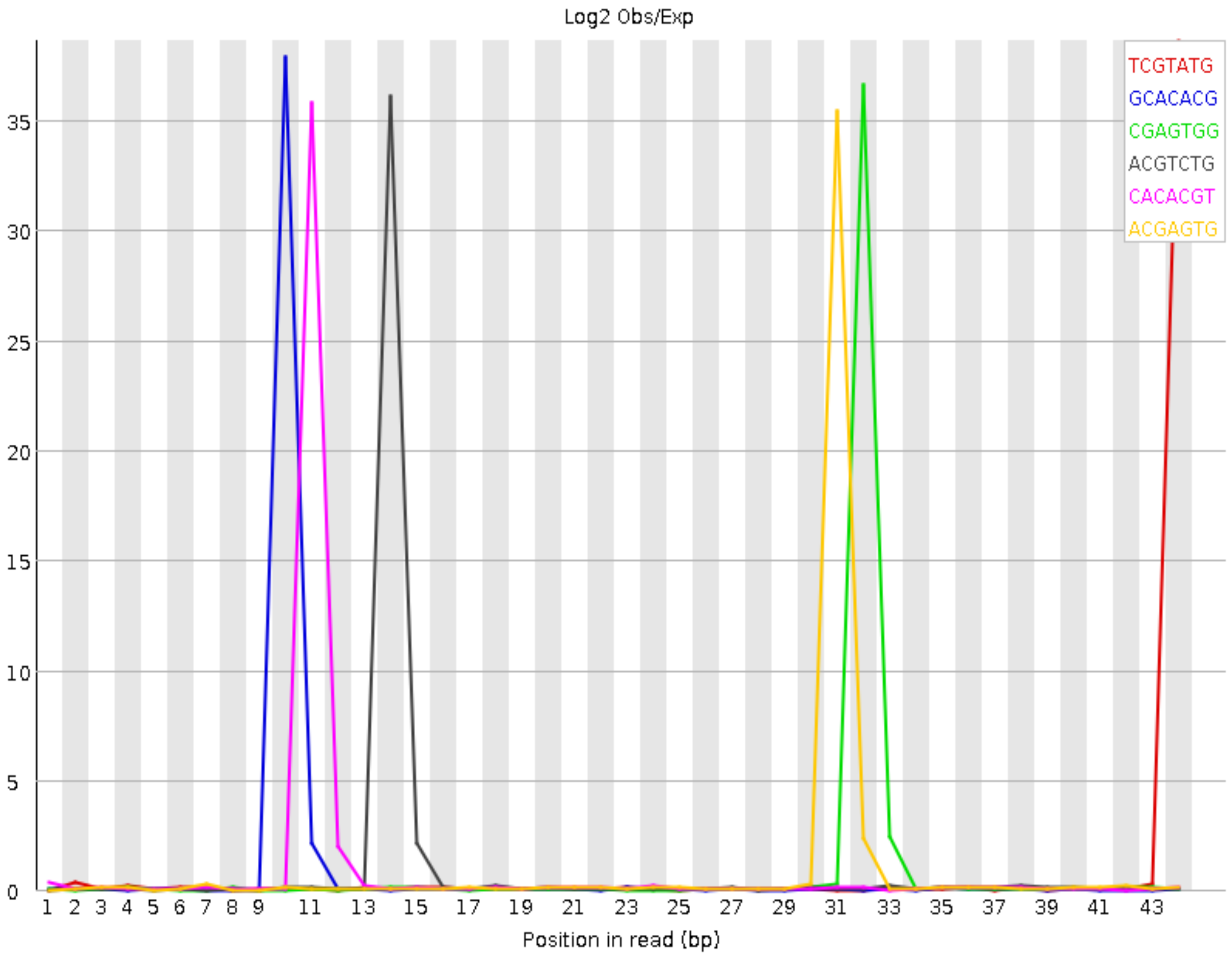

Sequence	Count	PValue	Obs/Exp Max	Max Obs/Exp Position
TCGTATG	22565	0.0	38.60924	44
GCACACG	24425	0.0	37.910328	10
CGAGTGG	25035	0.0	36.61914	32
ACGTCTG	25460	0.0	36.102253	14
CACACGT	25825	0.0	35.855167	11
ACGAGTG	25720	0.0	35.43007	31
ACACGTC	26155	0.0	35.335506	12
TATCTCG	25370	0.0	35.23794	40
AGCACAC	26770	0.0	34.696262	9
GAGCACA	27280	0.0	34.152428	8
AGTCACG	26695	0.0	34.032555	27
CACGAGT	26730	0.0	33.976135	30
TCGGAAG	27745	0.0	33.954906	2

Sequence	Count	PValue	Obs/Exp Max	Max Obs/Exp Position
AGTGGAT	27265	0.0	33.649868	34
GTCACGA	27065	0.0	33.633656	28
CTCGTAT	26735	0.0	33.60859	43
GTGGATA	27440	0.0	33.32791	35
CACGTCT	27990	0.0	33.03459	13
CGTCTGA	27835	0.0	33.013954	15
AGAGCAC	28275	0.0	32.95838	7

Produced by FastQC (version 0.11.5)
